# Identification of Lung-Cancer-Related Genes with the Shortest Path Approach in a Protein-Protein Interaction Network

**DOI:** 10.1155/2013/267375

**Published:** 2013-05-22

**Authors:** Bi-Qing Li, Jin You, Lei Chen, Jian Zhang, Ning Zhang, Hai-Peng Li, Tao Huang, Xiang-Yin Kong, Yu-Dong Cai

**Affiliations:** ^1^The Key Laboratory of Stem Cell Biology, Institute of Health Sciences, Shanghai Institutes for Biological Sciences, Chinese Academy of Sciences, Shanghai 200031, China; ^2^Key Laboratory of Systems Biology, Shanghai Institutes for Biological Sciences, Chinese Academy of Sciences, Shanghai 200031, China; ^3^Shanghai Jiao Tong University School of Medicine, Shanghai 200025, China; ^4^College of Information Engineering, Shanghai Maritime University, Shanghai 201306, China; ^5^Department of Ophthalmology, Shanghai First People's Hospital, Shanghai Jiaotong University, Shanghai 200080, China; ^6^Department of Biomedical Engineering Tianjin University, Tianjin Key Lab of BME Measurement, Tianjin 300072, China; ^7^CAS-MPG Partner Institute for Computational Biology, Shanghai Institutes for Biological Sciences, Chinese Academy of Sciences, Shanghai 200031, China; ^8^Department of Genetics and Genomic Sciences, Mount Sinai School of Medicine, New York, NY 10029, USA; ^9^Institute of Systems Biology, Shanghai University, Shanghai 200444, China

## Abstract

Lung cancer is one of the leading causes of cancer mortality worldwide. The main types of lung cancer are small cell lung cancer (SCLC) and nonsmall cell lung cancer (NSCLC). In this work, a computational method was proposed for identifying lung-cancer-related genes with a shortest path approach in a protein-protein interaction (PPI) network. Based on the PPI data from STRING, a weighted PPI network was constructed. 54 NSCLC- and 84 SCLC-related genes were retrieved from associated KEGG pathways. Then the shortest paths between each pair of these 54 NSCLC genes and 84 SCLC genes were obtained with Dijkstra's algorithm. Finally, all the genes on the shortest paths were extracted, and 25 and 38 shortest genes with a permutation *P* value less than 0.05 for NSCLC and SCLC were selected for further analysis. Some of the shortest path genes have been reported to be related to lung cancer. Intriguingly, the candidate genes we identified from the PPI network contained more cancer genes than those identified from the gene expression profiles. Furthermore, these genes possessed more functional similarity with the known cancer genes than those identified from the gene expression profiles. This study proved the efficiency of the proposed method and showed promising results.

## 1. Introduction

Lung cancer is one of the leading causes of cancer mortality worldwide [1]. Two main types of lung cancer are non-small cell lung cancer (NSCLC), which accounts for 80%–85%, and small cell lung cancer (SCLC), which accounts for around 20% of all cases. However, the SCLC has an extraordinarily high degree of metastasis and a strong association with smoking [2]. Diagnosis and treatment at the early stage of the disease process could reduce fatalities and increase the probability of disease-free survival. Therefore, it is meaningful to screen lung-cancer-related genes that could be used as prognostic factors or to help elucidate the mechanism of the disease.

Recently, as high-throughput biotechnologies develop rapidly, numerous biological data have been generated from processes such as protein complex, yeast two-hybrid systems, and gene expression profiles. These data are useful resources for understanding and deducing gene function. So far, protein-protein interaction (PPI) data has been widely utilized to annotate and predict the gene function assuming that interaction proteins possess the similar or identical functions and thus may participate in the same pathways. This so-called “guilt by association” rule was initially proposed by Nabieva et al. [3]. This rule could also be utilized to identify novel cancer-related genes.

Search Tool for the Retrieval of Interacting Genes (STRING) is an online database resource [4] that provides both predicted and experimental interaction information with a confidence score. It has been shown that proteins with short distances between each other in the PPI network tend to have the same biological functions [5–8], and interactive neighbors are prone to have the same biological functions as noninteractive ones [9, 10]. The possible reason is that the query protein and its interactive proteins might form a protein complex to exert a particular function or might participate in the same pathways.

Though great successes have been achieved for gene function prediction and identification of novel cancers-related genes with the application of the high-throughput data, yet high-throughput data is not error free. In this work, we proposed a computational method for identifying lung-cancer-related genes based on PPI network constructed from STRING. 54 NSCLC and 84 SCLC related genes were retrieved from associated KEGG pathways. Then, Dijkstra's algorithm [11] was employed to obtain the shortest paths between each pair of the 54 NSCLC and 84 SCLC genes. All the genes present on the shortest paths were extracted and analyzed. Several of these genes have been reported to be related to lung cancer. However, some of them were not previously reported. Therefore, there are probably novel lung-cancer-related genes and have the potential to be biomarkers for diagnosis of lung cancer.

## 2. Materials and Methods

### 2.1. Lung-Cancer-Related Gene List

We compiled all 54 genes existing in the human nonsmall cell lung cancer (NSCLC) pathway and 84 genes in the small cell lung cancer (SCLC) pathway from KEGG [12]. These two gene sets and corresponding Ensembl protein IDs are listed in Additional file S1 in Supplementary Matrial available online at doi: http://dx.doi.org/10.1155/2013/267375.

### 2.2. Lung Cancer Gene Expression Data

The gene expression profiling in Kastner et al.'s work was used in our study [13], which includes 8 SCLC, 16 NSCLC, and 14 normal lung tissue samples. It was retrieved from NCBI Gene Expression Omnibus (GEO) (Accession number: GSE40275). The gene expression profile was obtained by the Human Exon 1.0 ST Array with 56283 probes corresponding to 26410 genes. Signal intensity was first log2 transformed and then quantile normalized with “preprocessCore” package of R [14].

### 2.3. Identifying Differentially Expressed Genes

The “samr” package of R [15] was utilized to identify the differentially expressed genes between NSCLC, SCLC, and normal tissues separately with the criterion that false-discovery-rate- (FDR-) adjusted *P* value [16] was less than 0.01 and fold change was greater than 3 or less than 0.33.

### 2.4. Cancer-Related Gene List

A list of 742 cancer-related genes was compiled from three different sources [17]. First, a list of 457 cancer-related genes was collected from the Cancer Gene Census. Second, a list of cancer-related genes from the Atlas of Genetics and Cytogenetic in Oncology was retrieved [18]. We compiled the third list from the Human Protein Reference Database (HPRD) [19]. 

### 2.5. STRING PPI Data and Shortest Path Identification

 The initial weighted PPI network was constructed based on data from STRING (version 9.0) [4] (http://string.embl.de/). Each interaction in STRING is evaluated by an interaction confidence score in range from 1 to 999 to quantify the likelihood that an interaction may occur. We used Dijkstra's algorithm which has also been used in our previous works [20, 21] to identify the shortest path between each protein pair corresponding to the 54 NSCLC and 84 SCLC genes in the PPI network, respectively. Finally, all the proteins present on the shortest paths were ranked according to their betweenness. The Dijkstra's algorithm was implemented with R package “igraph” [22].

### 2.6. KEGG Pathway Enrichment Analysis

KEGG pathway enrichment analysis was performed with the functional annotation tool DAVID [23]. The enrichment *P* value was corrected with the Benjamin multiple testing correction method to control family-wide false discovery rate less than 0.05 [24]. All the protein-coding genes in human genome were taken as background during the enrichment analysis.

## 3. Results

### 3.1. Differentially Expressed Genes of the Gene Expression Profile

With the SAMR method, 1918 significantly upexpressed probes and 2243 downexpressed probes corresponding to 1825 genes were identified for NSCLC when compared with 14 normal lung tissues (for probes see additional file S2, and for gene symbols see additional file S3). For SCLC, 819 significantly up-expressed probes and 820 down-expressed probes corresponding to 1063 genes were identified (for probes see additional file S2, and for gene symbols see additional file S3). 

### 3.2. Shortest Path Genes and Enrichment Analysis

**Figure 1:**
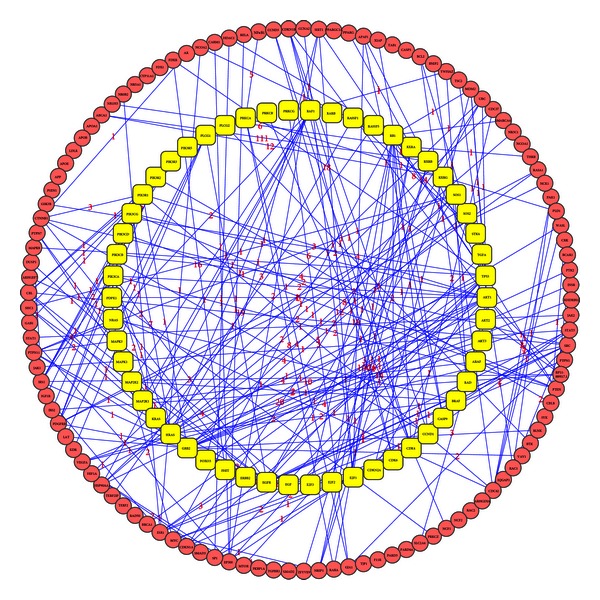
1711 shortest paths between 54 NSCLC genes. The 1171 shortest paths between 54 NSCLC genes were identified with Dijkstra's algorithm based on PPI data from STRING. Yellow round represents 54 NSCLC genes. Red round represents 114 genes existing on shortest paths. Numbers on edges represent the edge weight to quantify the interaction confidence. The smaller the number, the stronger the interaction between two nodes.

**Figure 2:**
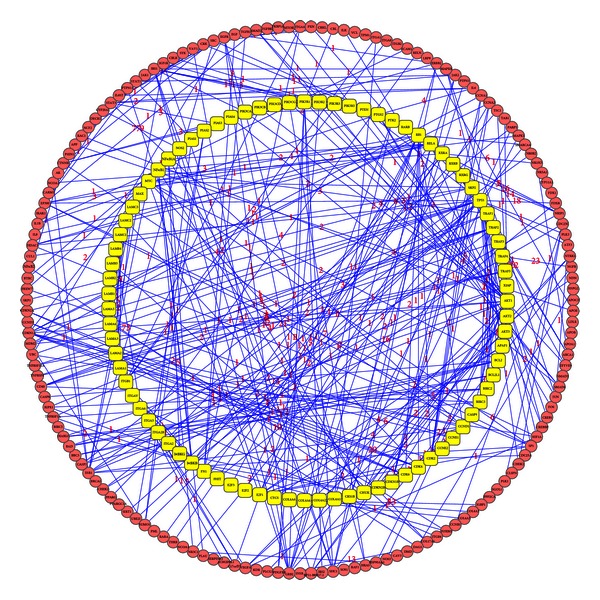
3916 shortest paths between 84 SCLC genes. The 3916 shortest paths between 84 SCLC genes were identified with Dijkstra's algorithm based on PPI data from STRING. Yellow round represents 84 SCLC genes. Red round represents 161 genes existing on shortest paths. Numbers on edges represent the edge weight to quantify the interaction confidence. The smaller the number, the stronger the interaction between two nodes.

An undirected graph was constructed with the PPI data from STRING. Subsequently, we repeatedly chose a pair of proteins corresponding to 54 NSCLC genes and the 84 SCLC genes respectively, and the shortest path between these two proteins was determined with Dijkstra's algorithm. A total of 1711 and 3916 shortest paths were obtained (see additional file S4) with lowest cost for NSCLC and SCLC containing 114 and 161 path genes, respectively. Shown in Figure 1 are the 1711 shortest paths between the 54 NSCLC genes. The weight was labeled on the edge between each of the interaction gene pairs. Shown in Figure 2 are the 3916 shortest paths between the 84 SCLC genes. To determine whether our 114 and 161 shortest path genes were also hubs in the background network, we performed a permutation to count the number of their occurrences on the shortest paths between 54 and 84 randomly selected genes only if they had a greater betweenness than that in our study. This process was repeated 2000 times, and the proportion of occurrences for the 114 and 161 shortest path genes was regarded as the *P* value. The detailed results thus obtained are given in additional file S5. Then we chose the 25 NSCLC and 38 SCLC shortest path genes with a *P* value less than 0.05 for further analysis (see additional file 5).

The GO enrichment analysis of 25 NSCLC shortest path genes indicated that they were significantly enriched in the regulation of intracellular signaling cascades and regulation of macromolecule metabolic processes (see additional file S6). These terms had been demonstrated to make great contributions to the survival and reproduction of cancer cells, while they also appeared in the enriched GO terms of 38 SCLC shortest path genes (see additional file S6). Besides these terms, the analysis result of SCLC shortest path genes showed that they were significantly enriched in cell adhesion processes, suggesting that genes in this term might play an important role in differentiating SCLC from NSCLC (see additional file S6). The KEGG pathway enrichment of these 38 SCLC shortest path genes indicated that they were enriched in canonical-cancer-related pathways such as the cell cycle and p53 signaling pathway (Table 1). 

### 3.3. Comparing the Overlap between Candidate Genes with 742 Cancer-Related Genes

The 25 and 38 shortest path genes were regarded as candidate genes for NSCLC and SCLC, respectively. We checked the overlap between 742 cancer genes and differentially expressed genes from the gene expression array as well as the overlap between the candidate genes identified in our study (Table 2). The entire 5-gene set can be found in additional file S3. From Table 2, we can see that both the lung cancer candidate genes identified from the gene expression array and those identified by our method had a significant overlap with the 742 cancer genes. However, the 25 NSCLC candidate genes identified with our method contained more cancer genes than those from the gene expression array (*P* value = 3.858*e* − 03) (Table 3). The 38 SCLC candidate genes had a higher percentage of cancer-related genes (0.1316) than those from expression array (0.0649) though the *P* value of Fisher's exact test was not significant (*P* value = 0.186). At least, the 38 SCLC candidate genes contained comparable cancer-related genes as those from gene expression array.

## 4. Discussion 

### 4.1. Shortest Path Genes in Nonsmall Cell Lung Cancer (NSCLC)

We identified 25 shortest path genes in NSCLC and 38 shortest path genes in SCLC with a permutation *P* value less than 0.05. Intriguingly the top five shortest path genes in NSCLC are also among the most significant genes in SCLC, while SCLC has several unique genes with large betweenness values. These may help to reveal the relationship between the two major types of lung cancer. 

As in NSCLC, HSP90AA1 [25–27] has been well documented to be relevant to lung cancer. We focus on candidate genes with large betweenness values and discuss the potential relationship between them and lung cancer.

Estrogen receptor 1 (ESR1) belongs to the nuclear steroid hormone receptor superfamily which acts as ligand-dependent, sequence-specific transcription factors and regulates the expression of genes involved in signal transduction, cell-cycle control, and cell survival [28]. Previous evidence showed that the proportion of never smokers among women with lung cancer is higher compared with men. Hypermethylation of ESR1 was reported to be detected only in lung tumors, but not in normal lung tissues, with a higher frequency being found in male patients than in female patients [29]. These all indicated ESR1 as a prognostic factor in lung cancer and as a potential target of hormone therapy.

ATP-binding cassette sub-family A member 1 (ABCA1) is a sulfonylurea-sensitive and cAMP-dependent anion transporter with critical impact on intracellular cholesterol transport. Cholesterol level increase has been found in cancers compared with normal tissue in many kinds of cancers [30], such as oral cancer [31]. Smith and Land demonstrated in colon cancer cells that ABCA1 had an anticancer activity in which deficiency allowed for increased mitochondrial cholesterol, inhibited release of mitochondrial cell death-promoting molecules, and facilitated cancer cell survival [32]. As abnormal metabolism is generally found in cancer, ABCA1 deserves further investigation with regard to its role in lung cancer.

Insulin receptor substrate 1 (IRS1) is an adaptor protein for insulin-like growth factor (IGF) signaling and is associated with IGF-stimulated proliferation [33]. It has been reported to be downregulated in NSCLC [34], and its degradation accelerates lung tumor growth by upgrading interaction between the potent mitogen platelet-derived growth factor receptor (PDGFR) and phosphatidylinositol 3 kinase (PI3 K) [35]. Correspondingly, our study shows that the shortest path of IRS1 is designated more than 100 and is significant in both NSCLC genes and SCLC genes, indicating that it may play a crucial part in lung cancer development.

FDXR (NADPH: adrenodoxin oxidoreductase) serves as the first electron transfer protein in the mitochondrial P450 systems. FDXR is identified to be target of the p53 family. It could be induced in a p53-dependent way by DNA damage in cells and participated in p53-mediated apoptosis via generating oxidative stress in mitochondria [36, 37]. Owing to the significance of p53 in apoptosis during tumorigenesis, the contribution of FDXR to lung cancer is worthy of further elucidation.

### 4.2. Shortest Path Genes in Small Cell Lung Cancer (SCLC)

The KEGG pathway enrichment analysis shows that there is a distinct group of shortest path genes in SCLC compared with NSCLC. These are the extracellular-matrix- (ECM-) related genes (Table 1). This coincides with the KEGG pathway enrichment analysis result of known SCLC pathway genes. ECM surrounds SCLC cells and includes collagen IV, tenascin, fibronectin, and laminin. Cell surface receptor integrins interact with ECM components and numerous signal transduction pathways which play important roles in cell cycle regulation, apoptosis, and so on and thus promote cancer cell proliferation [38]. Hodkinson et al. found that ECM can inhibit the caspase-3 activation and subsequent cell apoptosis induced by etoposide via stimulating phosphatidyl inositol 3-kinase- (PI3K-) signaling pathway in SCLC cells in a ITGB1/PI3K-dependent way [39]. Choi et al. demonstrated that downregulation of the phosphorylation activity of ILK (integrin-linked kinase) by single deletion of ILK protein itself or deletion of ITGB4/ILK complex could suppress the invasion of ovarian cancer [40]. Other studies also demonstrate that the intracellular signals activated by ECM components account for the high metastasis potential and drug resistance of SCLC [41]. In this work, we found that collagen IV members COL4A5 and COL4A3, integrin members ITGA1, ITGB4 and ITGA4, and linked kinase ILK all have a betweenness of more than 80 and a *P* value < 0.05, all of which may indicate their crucial roles in SCLC. 

Forkhead box protein M1 (FOXM1) is a transcription factor regulating cell proliferation and DNA damage repair [42, 43]. Research shows that it could be phosphorylated by MAPK (ERK) kinase [44] and then activate the expression of a number of cell-cycle-related genes which are crucial for DNA replication and mitotic division in the Ras-mitogen-activated protein-kinase- (MAPK-) signaling pathway, such as cyclin A2, cyclin B1, Aurora B kinase, Cdc25B phosphataseand Polo-like kinase1 [45]. Additionally, the protein level of FXOM1 has been found increased in prostate adenocarcinomas [46], infiltrating ductal breast carcinomas [47], basal cell carcinomas [48], intrahepatic cholangiocarcinomas [49], and in many other solid tumors [50]. A study by Kim et al. [51] showed that in human NSCLC Foxm1 protein is overexpressed and promotes tumor cells proliferation during the development of NSCLC. These all indicate that FXOM1 may play an import role in SCLC as well. 

Immunoglobulin-binding protein 1 (IGBP1) was formerly identified as a signal transduction molecule with a surface IgM receptor. More recently, it has been shown to regulate the phosphatase catalytic activity of protein phosphatase 2A (PP2A) [52]. PP2A is composed of a majority of cellular serine/threonine phosphatases [53] and regulates a number of important cellular processes, such as cell cycle transition, apoptosis, transcription, translation, autophagy [54], and cell transformation [55]. IGBP1 directly interacts with the catalytic subunit of PP2A [56], and this interaction leads to an antiapoptosis function. Recent studies show that, in carcinogen-transformed human cells and primary human cancers such as primary lung cancers, primary hepatocellular carcinomas and primary breast cancers, the expression level of IGBP1 is upregulated, [57]. Sakashita et al. found its overexpression in small cell adenocarcinomas [58], and Li et al. found that in a lung adenocarcinoma cell line the interaction of IGBP1 and Lactoferrin could induce cell apoptosis [59], implying IGBP1 to be a candidate target for SCLC therapy.

### 4.3. Functional Similarities between Candidate Genes and Known Cancer Genes

In order to compare the functional similarities between our candidate genes and the 742 known cancer genes, their functional profiles were constructed using the −log10 of the hypergeometric test *P* value on Gene Ontology (GO) terms [20, 21]. Then the Pearson correlation coefficient of their functional profiles was calculated [20, 21]. The functional similarities of five gene sets are shown in Table 4. All five gene sets can be found in additional file S3. Our 25 NSCLC (0.5554) and 38 SCLC (0.6919) candidate genes both had greater functional similarity with the cancer genes than the NSCLC (0.43139) and SCLC (0.48451) genes identified from gene expression profiles. It is suggested that our way is more efficient in identifying cancer-related genes.

## 5. Conclusion

In this study, we propose a computational method based on a protein-protein interaction network to identify cancer-related genes. We applied this method to lung cancer to find the shortest paths between 54 NSCLC and 84 SCLC genes in the protein-protein interaction network constructed based on STRING data and selected the 25 and 38 genes with a significant *P* value for NSCLC and SCLC, respectively. Analysis of these shortest path genes indicates that some of these genes, such as ESR1, FDXR, ABCA1, IRS1, HSP90AA1, FOXM1, and IGBP1 are related to lung cancer. In addition, the candidate genes of lung cancer identified in our study contain more cancer genes than those identified from gene expression profiles. Moreover, it is revealed that our candidate genes have greater functional similarity with the cancer genes than those identified from gene expression profiles. These candidate genes may be worth experiment validation and further research. It is expected that this method is useful in predicting novel cancer-related genes and has widespread use in cancer research.

## Supplementary Material

Additional file S1: This file contains two sheets. The first one shows 54 NSCLC related genes compiled from KEGG and corresponding Ensembl protein ID. The second one shows 84 SCLC related genes compiled from KEGG and corresponding Ensembl protein ID.
Additional file S2: This file contains four sheets. The first one shows the 1918 overexpressed probes in NSCLC compared with normal tissues identified from gene expression array GSE40275. The second one shows the 2243 downexpressed probes in NSCLC. The third one shows the 819 overexpressed probes in SCLC. The fourth one shows the 820 downexpressed probes in SCLC.
Additional file S3: This file contains five gene sets. The first one is the total 1825 differentially expressed NSCLC genes corresponding to the differentially expressed probes listed in Additional file S2. The second one is the total 1063 SCLC differentially expressed genes corresponding to the differentially expressed probes listed in Additional file S2. The third one is the 25 NSCLC shortest path genes. The fourth one is the 38 SCLC shortest path genes. The fifth one is the 742 cancer genes.
Additional file S4: 1711 shortest paths between 54 NSCLC genes and 3916 shortest paths between 84 SCLC genes.
Additional file S5: This file contains two sheets. The first one shows the 114 NSCLC shortest path genes with p-value. The second one shows the 161 SCLC shortest path genes with p-value.
Additional file S6: This file contains two sheets. The first one shows the GO enrichment result of 25 NSCLC shortest path genes. The second one shows the GO enrichment result of 38 SCLC shortest path genes.

## Figures and Tables

**Table 1:** KEGG enrichment analysis of 38 SCLC shortest path genes.

Term	Count^a^	Percentage^b^	*P* value	Benjamini adjusted *P* value
Focal adhesion	8	21.1	1.40*E* − 05	6.70*E* − 04
Regulation of actin cytoskeleton	7	18.4	2.20*E* − 04	5.40*E* − 03
Arrhythmogenic right ventricular cardiomyopathy (ARVC)	5	13.2	2.70*E* − 04	4.40*E* − 03
ECM-receptor interaction	5	13.2	4.00*E* − 04	4.80*E* − 03
Hypertrophic cardiomyopathy (HCM)	5	13.2	4.20*E* − 04	4.10*E* − 03
Dilated cardiomyopathy	5	13.2	5.70*E* − 04	4.60*E* − 03
Cell cycle	5	13.2	1.80*E* − 03	1.20*E* − 02
p53-signaling pathway	4	10.5	2.90*E* − 03	1.70*E* − 02

^a^The number of genes belonging to a certain pathway.

^b^The percentage of genes belonging to a certain pathway accounts for all the genes undergoing KEGG pathway analysis.

**Table 2:** Overlap between candidate genes and cancer-related genes.

Gene set	Number of candidate genes	Overlap with 742 cancer genes	*P* value
NSCLC from array	1825	93	6.698*e* − 04
SCLC from array	1063	69	2.218*e* − 06
NSCLC in our study	25	6	2.518*e* − 05
SCLC in our study	38	5	2.559*e* − 03

*P* value was calculated with the hypergeometric test assuming the total number of protein-coding genes was 20000.

**Table 3:** Comparing the overlap between candidate genes with cancer-related genes.

Gene set	Number of candidate genes	Overlap with 742 cancer genes	*P* value
NSCLC from array	1825	93	
NSCLC in our study	25	6	3.858*e* − 03
SCLC from array	1063	69	
SCLC in our study	38	5	0.186

*P* value was calculated with Fisher's exact test.

**Table 4:** The functional similarity between identified lung cancer genes and 742 cancer genes.

	742 cancer genes
1825 NSCLC genes from array	0.4314*
1063 SCLC genes from array	0.4845*
25 NSCLC genes from our study	0.5554*
38 SCLC genes from our study	0.6919*

*Pearson correlation coefficient of functional profiles.
