# Supplementary material for: Identification of Lung-Cancer-Related Genes with the Shortest Path Approach in a Protein-Protein Interaction Network
Source: Biomed Res Int. 2013 May 22;2013:267375. doi: 10.1155/2013/267375 (PMC3674655; doi:10.1155/2013/267375)
Supplement: Supplementary file 1 — Additional file S1: This file contains two sheets. The first one shows 54 NSCLC related genes compiled from KEGG and corresponding Ensembl protein ID. The second one shows 84 SCLC related genes compiled from KEGG and corresponding Ensembl protein ID. Additional file S2: This file contains four sheets. The first one shows the 1918 overexpressed probes in NSCLC compared with normal tissues identified from gene expression array GSE40275. The second one shows the 2243 downexpressed probes in NSCLC. The third one shows the 819 overexpressed probes in SCLC. The fourth one shows the 820 downexpressed probes in SCLC. Additional file S3: This file contains five gene sets. The first one is the total 1825 differentially expressed NSCLC genes corresponding to the differentially expressed probes listed in Additional file S2. The second one is the total 1063 SCLC differentially expressed genes corresponding to the differentially expressed probes listed in Additional file S2. The third one is the 25 NSCLC shortest path genes. The fourth one is the 38 SCLC shortest path genes. The fifth one is the 742 cancer genes. Additional file S4: 1711 shortest paths between 54 NSCLC genes and 3916 shortest paths between 84 SCLC genes. Additional file S5: This file contains two sheets. The first one shows the 114 NSCLC shortest path genes with p-value. The second one shows the 161 SCLC shortest path genes with p-value. Additional file S6: This file contains two sheets. The first one shows the GO enrichment result of 25 NSCLC shortest path genes. The second one shows the GO enrichment result of 38 SCLC shortest path genes. [file 267375.f1.zip › Additional file/Additional file S4.docx]

**Small cell lung cancer shortest path with cost**

1.ENSP00000007722->ENSP00000364094->ENSP00000386896->ENSP00000205386 75

2.ENSP00000007722->ENSP00000364094->ENSP00000380227->ENSP00000228307->ENSP00000341189->ENSP00000350941->ENSP00000264657->ENSP00000263253->ENSP00000384273->ENSP00000216797 9

3.ENSP00000007722->ENSP00000364094->ENSP00000380227->ENSP00000228307->ENSP00000300574->ENSP00000264122->ENSP00000222254 6

4.ENSP00000007722->ENSP00000364094->ENSP00000386896->ENSP00000200181->ENSP00000340937->ENSP00000264144->ENSP00000252999->ENSP00000222399 24

5.ENSP00000007722->ENSP00000364094->ENSP00000367316->ENSP00000303242->ENSP00000264832->ENSP00000226574 9

6.ENSP00000007722->ENSP00000364094->ENSP00000380227->ENSP00000228307->ENSP00000341189->ENSP00000350941->ENSP00000264657->ENSP00000227507 8

7.ENSP00000007722->ENSP00000364094->ENSP00000380227->ENSP00000228307->ENSP00000300574->ENSP00000275493->ENSP00000344818->ENSP00000259808->ENSP00000227758 10

8.ENSP00000007722->ENSP00000364094->ENSP00000380227->ENSP00000228307->ENSP00000299421->ENSP00000270202->ENSP00000228872 7

9.ENSP00000007722->ENSP00000364094->ENSP00000380227->ENSP00000228307->ENSP00000299421->ENSP00000270202->ENSP00000228872->ENSP00000255465->ENSP00000306043->ENSP00000342307->ENSP00000230538 21

10.ENSP00000007722->ENSP00000364094->ENSP00000380227->ENSP00000228307->ENSP00000300574->ENSP00000275493->ENSP00000344818->ENSP00000259808->ENSP00000247668 10

11.ENSP00000007722->ENSP00000364094->ENSP00000380227->ENSP00000228307->ENSP00000341189->ENSP00000339007->ENSP00000371067->ENSP00000354394->ENSP00000249636 8

12.ENSP00000007722->ENSP00000364094->ENSP00000386896->ENSP00000200181->ENSP00000340937->ENSP00000264144->ENSP00000252999 19

13.ENSP00000007722->ENSP00000364094->ENSP00000380227->ENSP00000228307->ENSP00000299421->ENSP00000270202->ENSP00000228872->ENSP00000257904 8

14.ENSP00000007722->ENSP00000364094->ENSP00000386896->ENSP00000200181->ENSP00000340937->ENSP00000264144->ENSP00000252999->ENSP00000258341 21

15.ENSP00000007722->ENSP00000364094->ENSP00000261023 2

16.ENSP00000007722->ENSP00000364094->ENSP00000380227->ENSP00000228307->ENSP00000300574->ENSP00000275493->ENSP00000344818->ENSP00000316840->ENSP00000269485->ENSP00000261464 12

17.ENSP00000007722->ENSP00000364094->ENSP00000380227->ENSP00000228307->ENSP00000341189->ENSP00000339007->ENSP00000401303->ENSP00000172229->ENSP00000262395 31

18.ENSP00000007722->ENSP00000364094->ENSP00000261023->ENSP00000262017->ENSP00000262407 4

19.ENSP00000007722->ENSP00000364094->ENSP00000380227->ENSP00000228307->ENSP00000299421->ENSP00000270202->ENSP00000228872->ENSP00000262643 8

20.ENSP00000007722->ENSP00000364094->ENSP00000380227->ENSP00000228307->ENSP00000341189->ENSP00000339007->ENSP00000304895->ENSP00000262741 9

21.ENSP00000007722->ENSP00000364094->ENSP00000380227->ENSP00000228307->ENSP00000341189->ENSP00000361021->ENSP00000269305->ENSP00000267163->ENSP00000262904 9

22.ENSP00000007722->ENSP00000346839->ENSP00000221930->ENSP00000364133->ENSP00000341551->ENSP00000262971 11

23.ENSP00000007722->ENSP00000364094->ENSP00000380227->ENSP00000228307->ENSP00000300574->ENSP00000275493->ENSP00000344818->ENSP00000316840->ENSP00000361359->ENSP00000263464 11

24.ENSP00000007722->ENSP00000364094->ENSP00000261023->ENSP00000361125->ENSP00000263826 27

25.ENSP00000007722->ENSP00000364094->ENSP00000380227->ENSP00000228307->ENSP00000341189->ENSP00000339007->ENSP00000274335->ENSP00000263967 7

26.ENSP00000007722->ENSP00000364094->ENSP00000386896->ENSP00000200181->ENSP00000340937->ENSP00000264144 17

27.ENSP00000007722->ENSP00000364094->ENSP00000380227->ENSP00000228307->ENSP00000299421->ENSP00000270202->ENSP00000228872->ENSP00000265734 8

28.ENSP00000007722->ENSP00000364094->ENSP00000380227->ENSP00000228307->ENSP00000299421->ENSP00000270202->ENSP00000228872->ENSP00000266970 8

29.ENSP00000007722->ENSP00000364094->ENSP00000380227->ENSP00000228307->ENSP00000341189->ENSP00000361021->ENSP00000269305->ENSP00000267163 8

30.ENSP00000007722->ENSP00000364094->ENSP00000380227->ENSP00000228307->ENSP00000299421->ENSP00000270202->ENSP00000352121->ENSP00000269300 15

31.ENSP00000007722->ENSP00000364094->ENSP00000380227->ENSP00000228307->ENSP00000341189->ENSP00000361021->ENSP00000269305 7

32.ENSP00000007722->ENSP00000364094->ENSP00000380227->ENSP00000228307->ENSP00000299421->ENSP00000270202 6

33.ENSP00000007722->ENSP00000364094->ENSP00000380227->ENSP00000228307->ENSP00000299421->ENSP00000270202->ENSP00000228872->ENSP00000274255 8

34.ENSP00000007722->ENSP00000364094->ENSP00000380227->ENSP00000228307->ENSP00000341189->ENSP00000339007->ENSP00000274335 6

35.ENSP00000007722->ENSP00000364094->ENSP00000380227->ENSP00000228307->ENSP00000299421->ENSP00000270202->ENSP00000228872->ENSP00000257904->ENSP00000276925 9

36.ENSP00000007722->ENSP00000364094->ENSP00000380227->ENSP00000228307->ENSP00000341189->ENSP00000339007->ENSP00000274335->ENSP00000289153 10

37.ENSP00000007722->ENSP00000364094->ENSP00000296585 2

38.ENSP00000007722->ENSP00000364094->ENSP00000380227->ENSP00000228307->ENSP00000299421->ENSP00000270202->ENSP00000309103->ENSP00000302564 8

39.ENSP00000007722->ENSP00000364094->ENSP00000380227->ENSP00000228307->ENSP00000300574->ENSP00000381107->ENSP00000348461->ENSP00000356505->ENSP00000220764->ENSP00000348380->ENSP00000305638 1023

40.ENSP00000007722->ENSP00000364094->ENSP00000386896->ENSP00000200181->ENSP00000340937->ENSP00000264144->ENSP00000252999->ENSP00000307156 24

41.ENSP00000007722->ENSP00000364094->ENSP00000380227->ENSP00000228307->ENSP00000341189->ENSP00000361021->ENSP00000269305->ENSP00000353059->ENSP00000307786 15

42.ENSP00000007722->ENSP00000364094->ENSP00000380227->ENSP00000228307->ENSP00000299421->ENSP00000270202->ENSP00000228872->ENSP00000266970->ENSP00000309181 9

43.ENSP00000007722->ENSP00000364094->ENSP00000380227->ENSP00000228307->ENSP00000299421->ENSP00000270202->ENSP00000228872->ENSP00000266970->ENSP00000311083 9

44.ENSP00000007722->ENSP00000364094->ENSP00000386896->ENSP00000200181->ENSP00000340937->ENSP00000348384->ENSP00000324532 12

45.ENSP00000007722->ENSP00000364094->ENSP00000367316->ENSP00000303242->ENSP00000264832->ENSP00000226574->ENSP00000327251 28

46.ENSP00000007722->ENSP00000364094->ENSP00000380227->ENSP00000228307->ENSP00000341189->ENSP00000361021->ENSP00000269305->ENSP00000329623 8

47.ENSP00000007722->ENSP00000364094->ENSP00000380227->ENSP00000228307->ENSP00000341189->ENSP00000361021->ENSP00000269305->ENSP00000353059->ENSP00000330237 10

48.ENSP00000007722->ENSP00000364094->ENSP00000380227->ENSP00000228307->ENSP00000341189->ENSP00000350941->ENSP00000206249->ENSP00000399968->ENSP00000419692->ENSP00000332296 24

49.ENSP00000007722->ENSP00000364094->ENSP00000380227->ENSP00000228307->ENSP00000300574->ENSP00000275493->ENSP00000344818->ENSP00000316840->ENSP00000361359->ENSP00000332468 10

50.ENSP00000007722->ENSP00000364094->ENSP00000380227->ENSP00000228307->ENSP00000341189->ENSP00000350941->ENSP00000264657->ENSP00000263253->ENSP00000384273->ENSP00000339151 9

51.ENSP00000007722->ENSP00000364094->ENSP00000380227->ENSP00000228307->ENSP00000341189 4

52.ENSP00000007722->ENSP00000364094->ENSP00000380227->ENSP00000228307->ENSP00000341189->ENSP00000361021->ENSP00000269305->ENSP00000293195->ENSP00000342087 41

53.ENSP00000007722->ENSP00000364094->ENSP00000380227->ENSP00000228307->ENSP00000341189->ENSP00000350941->ENSP00000206249->ENSP00000329357->ENSP00000345571 8

54.ENSP00000007722->ENSP00000346839 2

55.ENSP00000007722->ENSP00000364094->ENSP00000380227->ENSP00000228307->ENSP00000300574->ENSP00000275493->ENSP00000344818->ENSP00000316840->ENSP00000216160->ENSP00000347858 10

56.ENSP00000007722->ENSP00000364094->ENSP00000386896->ENSP00000200181->ENSP00000340937->ENSP00000348384 11

57.ENSP00000007722->ENSP00000364094->ENSP00000380227->ENSP00000228307->ENSP00000341189->ENSP00000339007->ENSP00000215832->ENSP00000367207->ENSP00000351490 9

58.ENSP00000007722->ENSP00000364094->ENSP00000380227->ENSP00000228307->ENSP00000299421->ENSP00000270202->ENSP00000352121 12

59.ENSP00000007722->ENSP00000364094->ENSP00000380227->ENSP00000228307->ENSP00000341189->ENSP00000350941->ENSP00000206249->ENSP00000327213->ENSP00000352900 21

60.ENSP00000007722->ENSP00000364094->ENSP00000380227->ENSP00000228307->ENSP00000341189->ENSP00000361021->ENSP00000269305->ENSP00000353059 9

61.ENSP00000007722->ENSP00000364094->ENSP00000282588->ENSP00000364979->ENSP00000353654 14

62.ENSP00000007722->ENSP00000364094->ENSP00000380227->ENSP00000228307->ENSP00000341189->ENSP00000339007->ENSP00000312435->ENSP00000400365->ENSP00000354360 20

63.ENSP00000007722->ENSP00000364094->ENSP00000380227->ENSP00000228307->ENSP00000341189->ENSP00000361021->ENSP00000269305->ENSP00000267163->ENSP00000355249 9

64.ENSP00000007722->ENSP00000364094->ENSP00000380227->ENSP00000228307->ENSP00000341189->ENSP00000350941->ENSP00000264657->ENSP00000258743->ENSP00000356438 11

65.ENSP00000007722->ENSP00000364094->ENSP00000380227->ENSP00000228307->ENSP00000300574->ENSP00000275493->ENSP00000344818->ENSP00000316840->ENSP00000358622 9

66.ENSP00000007722->ENSP00000364094->ENSP00000380227->ENSP00000228307->ENSP00000341189->ENSP00000350941->ENSP00000264657->ENSP00000263253->ENSP00000384273->ENSP00000359424 9

67.ENSP00000007722->ENSP00000364094->ENSP00000380227->ENSP00000228307->ENSP00000341189->ENSP00000361021 6

68.ENSP00000007722->ENSP00000364094->ENSP00000380227->ENSP00000228307->ENSP00000341189->ENSP00000361021->ENSP00000269305->ENSP00000278916->ENSP00000321656->ENSP00000300093->ENSP00000263753->ENSP00000418447->ENSP00000348784->ENSP00000379866->ENSP00000379823->ENSP00000331902->ENSP00000361290 51

69.ENSP00000007722->ENSP00000364094->ENSP00000380227->ENSP00000228307->ENSP00000300574->ENSP00000275493->ENSP00000344818->ENSP00000316840->ENSP00000361359->ENSP00000362994 10

70.ENSP00000007722->ENSP00000364094->ENSP00000380227->ENSP00000228307->ENSP00000341189->ENSP00000350941->ENSP00000206249->ENSP00000327213->ENSP00000363812 33

71.ENSP00000007722->ENSP00000364094 1

72.ENSP00000007722->ENSP00000364094->ENSP00000282588->ENSP00000364979 13

73.ENSP00000007722->ENSP00000364094->ENSP00000380227->ENSP00000228307->ENSP00000341189->ENSP00000361021->ENSP00000366563 18

74.ENSP00000007722->ENSP00000364094->ENSP00000380227->ENSP00000228307->ENSP00000341189->ENSP00000339007->ENSP00000215832->ENSP00000367207 8

75.ENSP00000007722->ENSP00000392423->ENSP00000303634->ENSP00000252486->ENSP00000252444->ENSP00000233242->ENSP00000236850->ENSP00000363868->ENSP00000372703 125

76.ENSP00000007722->ENSP00000364094->ENSP00000386896->ENSP00000200181->ENSP00000340937->ENSP00000264144->ENSP00000252999->ENSP00000258341->ENSP00000374309 26

77.ENSP00000007722->ENSP00000364094->ENSP00000380227->ENSP00000228307->ENSP00000341189->ENSP00000339007->ENSP00000304895->ENSP00000348986->ENSP00000375892 12

78.ENSP00000007722->ENSP00000364094->ENSP00000380227->ENSP00000228307->ENSP00000341189->ENSP00000350941->ENSP00000264657->ENSP00000376765 9

79.ENSP00000007722->ENSP00000364094->ENSP00000380227->ENSP00000228307->ENSP00000341189->ENSP00000361021->ENSP00000269305->ENSP00000278916->ENSP00000321656->ENSP00000300093->ENSP00000263753->ENSP00000418447->ENSP00000348784->ENSP00000379866 48

80.ENSP00000007722->ENSP00000364094->ENSP00000380227->ENSP00000228307->ENSP00000341189->ENSP00000361021->ENSP00000269305->ENSP00000324897->ENSP00000381648 9

81.ENSP00000007722->ENSP00000364094->ENSP00000380227->ENSP00000228307->ENSP00000341189->ENSP00000350941->ENSP00000264657->ENSP00000263253->ENSP00000384273 8

82.ENSP00000007722->ENSP00000364094->ENSP00000386896 2

83.ENSP00000007722->ENSP00000392423->ENSP00000303634->ENSP00000252486->ENSP00000252444->ENSP00000233242->ENSP00000236850->ENSP00000363868->ENSP00000393286 125

84.ENSP00000007722->ENSP00000392423->ENSP00000303634->ENSP00000252486->ENSP00000252444->ENSP00000233242->ENSP00000236850->ENSP00000363868->ENSP00000393870 125

85.ENSP00000007722->ENSP00000346839->ENSP00000221930->ENSP00000262160->ENSP00000287727->ENSP00000400104 107

86.ENSP00000007722->ENSP00000364094->ENSP00000380227->ENSP00000228307->ENSP00000341189->ENSP00000339007->ENSP00000312435->ENSP00000400365 19

87.ENSP00000007722->ENSP00000392423->ENSP00000303634->ENSP00000252486->ENSP00000252444->ENSP00000233242->ENSP00000236850->ENSP00000363868->ENSP00000402590 125

88.ENSP00000007722->ENSP00000364094->ENSP00000380227->ENSP00000228307->ENSP00000341189->ENSP00000350941->ENSP00000206249->ENSP00000399968->ENSP00000419692 8

89.ENSP00000205386->ENSP00000386896->ENSP00000364094->ENSP00000380227->ENSP00000228307->ENSP00000341189->ENSP00000350941->ENSP00000344456->ENSP00000359206->ENSP00000216797 82

90.ENSP00000205386->ENSP00000386896->ENSP00000364094->ENSP00000380227->ENSP00000228307->ENSP00000300574->ENSP00000264122->ENSP00000222254 79

91.ENSP00000205386->ENSP00000230538->ENSP00000222399 91

92.ENSP00000205386->ENSP00000386896->ENSP00000364094->ENSP00000367316->ENSP00000303242->ENSP00000264832->ENSP00000226574 82

93.ENSP00000205386->ENSP00000386896->ENSP00000364094->ENSP00000380227->ENSP00000228307->ENSP00000341189->ENSP00000350941->ENSP00000264657->ENSP00000227507 81

94.ENSP00000205386->ENSP00000386896->ENSP00000364094->ENSP00000380227->ENSP00000228307->ENSP00000300574->ENSP00000275493->ENSP00000344818->ENSP00000259808->ENSP00000227758 83

95.ENSP00000205386->ENSP00000386896->ENSP00000364094->ENSP00000380227->ENSP00000228307->ENSP00000299421->ENSP00000270202->ENSP00000228872 80

96.ENSP00000205386->ENSP00000230538 87

97.ENSP00000205386->ENSP00000386896->ENSP00000364094->ENSP00000380227->ENSP00000228307->ENSP00000300574->ENSP00000275493->ENSP00000344818->ENSP00000259808->ENSP00000247668 83

98.ENSP00000205386->ENSP00000386896->ENSP00000364094->ENSP00000380227->ENSP00000228307->ENSP00000341189->ENSP00000339007->ENSP00000371067->ENSP00000354394->ENSP00000249636 81

99.ENSP00000205386->ENSP00000386896->ENSP00000200181->ENSP00000340937->ENSP00000264144->ENSP00000252999 90

100.ENSP00000205386->ENSP00000386896->ENSP00000364094->ENSP00000380227->ENSP00000228307->ENSP00000299421->ENSP00000270202->ENSP00000228872->ENSP00000257904 81

101.ENSP00000205386->ENSP00000386896->ENSP00000200181->ENSP00000340937->ENSP00000264144->ENSP00000252999->ENSP00000258341 92

102.ENSP00000205386->ENSP00000386896->ENSP00000364094->ENSP00000261023 75

103.ENSP00000205386->ENSP00000386896->ENSP00000364094->ENSP00000380227->ENSP00000228307->ENSP00000300574->ENSP00000275493->ENSP00000344818->ENSP00000316840->ENSP00000269485->ENSP00000261464 85

104.ENSP00000205386->ENSP00000386896->ENSP00000364094->ENSP00000380227->ENSP00000228307->ENSP00000341189->ENSP00000339007->ENSP00000401303->ENSP00000172229->ENSP00000262395 104

105.ENSP00000205386->ENSP00000386896->ENSP00000364094->ENSP00000261023->ENSP00000262017->ENSP00000262407 77

106.ENSP00000205386->ENSP00000386896->ENSP00000364094->ENSP00000380227->ENSP00000228307->ENSP00000299421->ENSP00000270202->ENSP00000228872->ENSP00000262643 81

107.ENSP00000205386->ENSP00000386896->ENSP00000364094->ENSP00000380227->ENSP00000228307->ENSP00000341189->ENSP00000339007->ENSP00000304895->ENSP00000262741 82

108.ENSP00000205386->ENSP00000386896->ENSP00000364094->ENSP00000380227->ENSP00000228307->ENSP00000341189->ENSP00000361021->ENSP00000269305->ENSP00000267163->ENSP00000262904 82

109.ENSP00000205386->ENSP00000386896->ENSP00000364094->ENSP00000346839->ENSP00000221930->ENSP00000364133->ENSP00000341551->ENSP00000262971 84

110.ENSP00000205386->ENSP00000386896->ENSP00000364094->ENSP00000380227->ENSP00000228307->ENSP00000300574->ENSP00000275493->ENSP00000344818->ENSP00000316840->ENSP00000361359->ENSP00000263464 84

111.ENSP00000205386->ENSP00000386896->ENSP00000364094->ENSP00000261023->ENSP00000361125->ENSP00000263826 100

112.ENSP00000205386->ENSP00000386896->ENSP00000364094->ENSP00000380227->ENSP00000228307->ENSP00000341189->ENSP00000339007->ENSP00000274335->ENSP00000263967 80

113.ENSP00000205386->ENSP00000386896->ENSP00000200181->ENSP00000340937->ENSP00000264144 88

114.ENSP00000205386->ENSP00000386896->ENSP00000364094->ENSP00000380227->ENSP00000228307->ENSP00000299421->ENSP00000270202->ENSP00000228872->ENSP00000265734 81

115.ENSP00000205386->ENSP00000386896->ENSP00000364094->ENSP00000380227->ENSP00000228307->ENSP00000341189->ENSP00000361021->ENSP00000269305->ENSP00000266970 81

116.ENSP00000205386->ENSP00000386896->ENSP00000364094->ENSP00000380227->ENSP00000228307->ENSP00000341189->ENSP00000361021->ENSP00000269305->ENSP00000267163 81

117.ENSP00000205386->ENSP00000386896->ENSP00000364094->ENSP00000380227->ENSP00000228307->ENSP00000299421->ENSP00000270202->ENSP00000352121->ENSP00000269300 88

118.ENSP00000205386->ENSP00000386896->ENSP00000364094->ENSP00000380227->ENSP00000228307->ENSP00000341189->ENSP00000361021->ENSP00000269305 80

119.ENSP00000205386->ENSP00000386896->ENSP00000364094->ENSP00000380227->ENSP00000228307->ENSP00000299421->ENSP00000270202 79

120.ENSP00000205386->ENSP00000386896->ENSP00000364094->ENSP00000380227->ENSP00000228307->ENSP00000299421->ENSP00000270202->ENSP00000228872->ENSP00000274255 81

121.ENSP00000205386->ENSP00000386896->ENSP00000364094->ENSP00000380227->ENSP00000228307->ENSP00000341189->ENSP00000339007->ENSP00000274335 79

122.ENSP00000205386->ENSP00000386896->ENSP00000364094->ENSP00000380227->ENSP00000228307->ENSP00000299421->ENSP00000270202->ENSP00000228872->ENSP00000257904->ENSP00000276925 82

123.ENSP00000205386->ENSP00000386896->ENSP00000364094->ENSP00000380227->ENSP00000228307->ENSP00000341189->ENSP00000339007->ENSP00000274335->ENSP00000289153 83

124.ENSP00000205386->ENSP00000386896->ENSP00000364094->ENSP00000296585 75

125.ENSP00000205386->ENSP00000386896->ENSP00000364094->ENSP00000380227->ENSP00000228307->ENSP00000299421->ENSP00000270202->ENSP00000309103->ENSP00000302564 81

126.ENSP00000205386->ENSP00000386896->ENSP00000364094->ENSP00000380227->ENSP00000228307->ENSP00000300574->ENSP00000381107->ENSP00000348461->ENSP00000356505->ENSP00000220764->ENSP00000348380->ENSP00000305638 1096

127.ENSP00000205386->ENSP00000386896->ENSP00000200181->ENSP00000340937->ENSP00000264144->ENSP00000252999->ENSP00000307156 95

128.ENSP00000205386->ENSP00000386896->ENSP00000364094->ENSP00000380227->ENSP00000228307->ENSP00000341189->ENSP00000361021->ENSP00000269305->ENSP00000353059->ENSP00000307786 88

129.ENSP00000205386->ENSP00000386896->ENSP00000364094->ENSP00000380227->ENSP00000228307->ENSP00000341189->ENSP00000361021->ENSP00000269305->ENSP00000266970->ENSP00000309181 82

130.ENSP00000205386->ENSP00000386896->ENSP00000364094->ENSP00000380227->ENSP00000228307->ENSP00000341189->ENSP00000361021->ENSP00000269305->ENSP00000266970->ENSP00000311083 82

131.ENSP00000205386->ENSP00000386896->ENSP00000200181->ENSP00000340937->ENSP00000348384->ENSP00000324532 83

132.ENSP00000205386->ENSP00000386896->ENSP00000364094->ENSP00000367316->ENSP00000303242->ENSP00000264832->ENSP00000226574->ENSP00000327251 101

133.ENSP00000205386->ENSP00000386896->ENSP00000364094->ENSP00000380227->ENSP00000228307->ENSP00000341189->ENSP00000361021->ENSP00000269305->ENSP00000329623 81

134.ENSP00000205386->ENSP00000386896->ENSP00000364094->ENSP00000380227->ENSP00000228307->ENSP00000341189->ENSP00000361021->ENSP00000269305->ENSP00000353059->ENSP00000330237 83

135.ENSP00000205386->ENSP00000386896->ENSP00000364094->ENSP00000380227->ENSP00000228307->ENSP00000341189->ENSP00000350941->ENSP00000206249->ENSP00000399968->ENSP00000419692->ENSP00000332296 97

136.ENSP00000205386->ENSP00000386896->ENSP00000364094->ENSP00000380227->ENSP00000228307->ENSP00000300574->ENSP00000275493->ENSP00000344818->ENSP00000316840->ENSP00000361359->ENSP00000332468 83

137.ENSP00000205386->ENSP00000386896->ENSP00000364094->ENSP00000346839->ENSP00000221930->ENSP00000262160->ENSP00000263253->ENSP00000384273->ENSP00000339151 82

138.ENSP00000205386->ENSP00000386896->ENSP00000364094->ENSP00000380227->ENSP00000228307->ENSP00000341189 77

139.ENSP00000205386->ENSP00000386896->ENSP00000364094->ENSP00000380227->ENSP00000228307->ENSP00000341189->ENSP00000361021->ENSP00000269305->ENSP00000293195->ENSP00000342087 114

140.ENSP00000205386->ENSP00000386896->ENSP00000364094->ENSP00000380227->ENSP00000228307->ENSP00000341189->ENSP00000350941->ENSP00000206249->ENSP00000329357->ENSP00000345571 81

141.ENSP00000205386->ENSP00000386896->ENSP00000364094->ENSP00000346839 75

142.ENSP00000205386->ENSP00000386896->ENSP00000364094->ENSP00000380227->ENSP00000228307->ENSP00000300574->ENSP00000275493->ENSP00000344818->ENSP00000316840->ENSP00000216160->ENSP00000347858 83

143.ENSP00000205386->ENSP00000386896->ENSP00000200181->ENSP00000340937->ENSP00000348384 82

144.ENSP00000205386->ENSP00000386896->ENSP00000364094->ENSP00000380227->ENSP00000228307->ENSP00000341189->ENSP00000350941->ENSP00000206249->ENSP00000367207->ENSP00000351490 82

145.ENSP00000205386->ENSP00000386896->ENSP00000364094->ENSP00000380227->ENSP00000228307->ENSP00000299421->ENSP00000270202->ENSP00000352121 85

146.ENSP00000205386->ENSP00000386896->ENSP00000364094->ENSP00000380227->ENSP00000228307->ENSP00000341189->ENSP00000350941->ENSP00000206249->ENSP00000327213->ENSP00000352900 94

147.ENSP00000205386->ENSP00000386896->ENSP00000364094->ENSP00000380227->ENSP00000228307->ENSP00000341189->ENSP00000361021->ENSP00000269305->ENSP00000353059 82

148.ENSP00000205386->ENSP00000386896->ENSP00000364094->ENSP00000282588->ENSP00000364979->ENSP00000353654 87

149.ENSP00000205386->ENSP00000230538->ENSP00000354360 88

150.ENSP00000205386->ENSP00000386896->ENSP00000364094->ENSP00000380227->ENSP00000228307->ENSP00000341189->ENSP00000361021->ENSP00000269305->ENSP00000267163->ENSP00000355249 82

151.ENSP00000205386->ENSP00000386896->ENSP00000364094->ENSP00000380227->ENSP00000228307->ENSP00000341189->ENSP00000350941->ENSP00000264657->ENSP00000258743->ENSP00000356438 84

152.ENSP00000205386->ENSP00000386896->ENSP00000364094->ENSP00000380227->ENSP00000228307->ENSP00000300574->ENSP00000275493->ENSP00000344818->ENSP00000316840->ENSP00000358622 82

153.ENSP00000205386->ENSP00000386896->ENSP00000364094->ENSP00000346839->ENSP00000221930->ENSP00000262160->ENSP00000263253->ENSP00000384273->ENSP00000359424 82

154.ENSP00000205386->ENSP00000386896->ENSP00000364094->ENSP00000380227->ENSP00000228307->ENSP00000341189->ENSP00000361021 79

155.ENSP00000205386->ENSP00000386896->ENSP00000364094->ENSP00000380227->ENSP00000228307->ENSP00000341189->ENSP00000361021->ENSP00000269305->ENSP00000278916->ENSP00000312995->ENSP00000300093->ENSP00000263753->ENSP00000418447->ENSP00000348784->ENSP00000379866->ENSP00000379823->ENSP00000331902->ENSP00000361290 124

156.ENSP00000205386->ENSP00000386896->ENSP00000364094->ENSP00000380227->ENSP00000228307->ENSP00000300574->ENSP00000275493->ENSP00000344818->ENSP00000316840->ENSP00000361359->ENSP00000362994 83

157.ENSP00000205386->ENSP00000386896->ENSP00000364094->ENSP00000380227->ENSP00000228307->ENSP00000341189->ENSP00000350941->ENSP00000206249->ENSP00000327213->ENSP00000363812 106

158.ENSP00000205386->ENSP00000386896->ENSP00000364094 74

159.ENSP00000205386->ENSP00000386896->ENSP00000364094->ENSP00000282588->ENSP00000364979 86

160.ENSP00000205386->ENSP00000386896->ENSP00000364094->ENSP00000380227->ENSP00000228307->ENSP00000341189->ENSP00000361021->ENSP00000366563 91

161.ENSP00000205386->ENSP00000386896->ENSP00000364094->ENSP00000380227->ENSP00000228307->ENSP00000341189->ENSP00000350941->ENSP00000206249->ENSP00000367207 81

162.ENSP00000205386->ENSP00000386896->ENSP00000364094->ENSP00000380227->ENSP00000228307->ENSP00000341189->ENSP00000350941->ENSP00000206249->ENSP00000254227->ENSP00000342470->ENSP00000363868->ENSP00000372703 199

163.ENSP00000205386->ENSP00000374309 92

164.ENSP00000205386->ENSP00000386896->ENSP00000364094->ENSP00000380227->ENSP00000228307->ENSP00000341189->ENSP00000339007->ENSP00000304895->ENSP00000348986->ENSP00000375892 85

165.ENSP00000205386->ENSP00000386896->ENSP00000364094->ENSP00000380227->ENSP00000228307->ENSP00000341189->ENSP00000350941->ENSP00000264657->ENSP00000376765 82

166.ENSP00000205386->ENSP00000386896->ENSP00000364094->ENSP00000380227->ENSP00000228307->ENSP00000341189->ENSP00000361021->ENSP00000269305->ENSP00000278916->ENSP00000312995->ENSP00000300093->ENSP00000263753->ENSP00000418447->ENSP00000348784->ENSP00000379866 121

167.ENSP00000205386->ENSP00000386896->ENSP00000364094->ENSP00000380227->ENSP00000228307->ENSP00000341189->ENSP00000361021->ENSP00000269305->ENSP00000324897->ENSP00000381648 82

168.ENSP00000205386->ENSP00000386896->ENSP00000364094->ENSP00000346839->ENSP00000221930->ENSP00000262160->ENSP00000263253->ENSP00000384273 81

169.ENSP00000205386->ENSP00000386896 73

170.ENSP00000205386->ENSP00000386896->ENSP00000364094->ENSP00000380227->ENSP00000228307->ENSP00000341189->ENSP00000350941->ENSP00000206249->ENSP00000254227->ENSP00000342470->ENSP00000363868->ENSP00000393286 199

171.ENSP00000205386->ENSP00000386896->ENSP00000364094->ENSP00000380227->ENSP00000228307->ENSP00000341189->ENSP00000350941->ENSP00000206249->ENSP00000254227->ENSP00000342470->ENSP00000363868->ENSP00000393870 199

172.ENSP00000205386->ENSP00000386896->ENSP00000364094->ENSP00000346839->ENSP00000221930->ENSP00000262160->ENSP00000287727->ENSP00000400104 180

173.ENSP00000205386->ENSP00000230538->ENSP00000354360->ENSP00000400365 89

174.ENSP00000205386->ENSP00000386896->ENSP00000364094->ENSP00000380227->ENSP00000228307->ENSP00000341189->ENSP00000350941->ENSP00000206249->ENSP00000254227->ENSP00000342470->ENSP00000363868->ENSP00000402590 199

175.ENSP00000205386->ENSP00000386896->ENSP00000364094->ENSP00000380227->ENSP00000228307->ENSP00000341189->ENSP00000350941->ENSP00000206249->ENSP00000399968->ENSP00000419692 81

176.ENSP00000216797->ENSP00000384273->ENSP00000262367->ENSP00000269305->ENSP00000353483->ENSP00000304895->ENSP00000222254 6

177.ENSP00000216797->ENSP00000384273->ENSP00000262367->ENSP00000269305->ENSP00000278916->ENSP00000321656->ENSP00000256442->ENSP00000342307->ENSP00000230538->ENSP00000222399 21

178.ENSP00000216797->ENSP00000226574 1

179.ENSP00000216797->ENSP00000359206->ENSP00000344456->ENSP00000227507 3

180.ENSP00000216797->ENSP00000359424->ENSP00000247668->ENSP00000227758 3

181.ENSP00000216797->ENSP00000359206->ENSP00000326804->ENSP00000274255->ENSP00000228872 4

182.ENSP00000216797->ENSP00000384273->ENSP00000262367->ENSP00000269305->ENSP00000278916->ENSP00000321656->ENSP00000256442->ENSP00000342307->ENSP00000230538 17

183.ENSP00000216797->ENSP00000359424->ENSP00000247668 2

184.ENSP00000216797->ENSP00000384273->ENSP00000263253->ENSP00000354394->ENSP00000249636 4

185.ENSP00000216797->ENSP00000384273->ENSP00000262367->ENSP00000269305->ENSP00000278916->ENSP00000321656->ENSP00000256442->ENSP00000342307->ENSP00000230538->ENSP00000354360->ENSP00000252999 23

186.ENSP00000216797->ENSP00000384273->ENSP00000362649->ENSP00000267163->ENSP00000257904 4

187.ENSP00000216797->ENSP00000359206->ENSP00000344456->ENSP00000269571->ENSP00000339007->ENSP00000312435->ENSP00000400365->ENSP00000258341 23

188.ENSP00000216797->ENSP00000384273->ENSP00000263253->ENSP00000262160->ENSP00000221930->ENSP00000346839->ENSP00000261023 8

189.ENSP00000216797->ENSP00000359424->ENSP00000247668->ENSP00000263932->ENSP00000261464 5

190.ENSP00000216797->ENSP00000359206->ENSP00000344456->ENSP00000269571->ENSP00000401303->ENSP00000172229->ENSP00000262395 30

191.ENSP00000216797->ENSP00000384273->ENSP00000263253->ENSP00000262160->ENSP00000221930->ENSP00000346839->ENSP00000261023->ENSP00000262017->ENSP00000262407 10

192.ENSP00000216797->ENSP00000359206->ENSP00000231487->ENSP00000281708->ENSP00000262643 4

193.ENSP00000216797->ENSP00000384273->ENSP00000262367->ENSP00000269305->ENSP00000353483->ENSP00000304895->ENSP00000262741 8

194.ENSP00000216797->ENSP00000384273->ENSP00000362649->ENSP00000267163->ENSP00000262904 4

195.ENSP00000216797->ENSP00000384273->ENSP00000263253->ENSP00000262160->ENSP00000341551->ENSP00000262971 8

196.ENSP00000216797->ENSP00000359424->ENSP00000247668->ENSP00000263464 3

197.ENSP00000216797->ENSP00000384273->ENSP00000262367->ENSP00000338018->ENSP00000361125->ENSP00000263826 24

198.ENSP00000216797->ENSP00000384273->ENSP00000262367->ENSP00000269305->ENSP00000361021->ENSP00000263967 6

199.ENSP00000216797->ENSP00000359206->ENSP00000344456->ENSP00000269571->ENSP00000339007->ENSP00000341189->ENSP00000228307->ENSP00000380227->ENSP00000364094->ENSP00000386896->ENSP00000200181->ENSP00000340937->ENSP00000264144 24

200.ENSP00000216797->ENSP00000359206->ENSP00000344456->ENSP00000227507->ENSP00000265734 4

201.ENSP00000216797->ENSP00000384273->ENSP00000262367->ENSP00000269305->ENSP00000266970 4

202.ENSP00000216797->ENSP00000384273->ENSP00000362649->ENSP00000267163 3

203.ENSP00000216797->ENSP00000384273->ENSP00000263253->ENSP00000206249->ENSP00000270202->ENSP00000352121->ENSP00000269300 14

204.ENSP00000216797->ENSP00000384273->ENSP00000262367->ENSP00000269305 3

205.ENSP00000216797->ENSP00000384273->ENSP00000263253->ENSP00000206249->ENSP00000270202 5

206.ENSP00000216797->ENSP00000359206->ENSP00000326804->ENSP00000274255 3

207.ENSP00000216797->ENSP00000359206->ENSP00000344456->ENSP00000274335 5

208.ENSP00000216797->ENSP00000384273->ENSP00000362649->ENSP00000267163->ENSP00000257904->ENSP00000276925 5

209.ENSP00000216797->ENSP00000384273->ENSP00000263253->ENSP00000206249->ENSP00000270202->ENSP00000289153 9

210.ENSP00000216797->ENSP00000359206->ENSP00000344456->ENSP00000269571->ENSP00000339007->ENSP00000341189->ENSP00000228307->ENSP00000380227->ENSP00000364094->ENSP00000296585 9

211.ENSP00000216797->ENSP00000384273->ENSP00000262367->ENSP00000269305->ENSP00000302564 5

212.ENSP00000216797->ENSP00000359206->ENSP00000344456->ENSP00000348461->ENSP00000356505->ENSP00000220764->ENSP00000348380->ENSP00000305638 1022

213.ENSP00000216797->ENSP00000384273->ENSP00000262367->ENSP00000269305->ENSP00000278916->ENSP00000321656->ENSP00000256442->ENSP00000342307->ENSP00000230538->ENSP00000354360->ENSP00000252999->ENSP00000307156 28

214.ENSP00000216797->ENSP00000384273->ENSP00000262367->ENSP00000269305->ENSP00000353059->ENSP00000307786 11

215.ENSP00000216797->ENSP00000384273->ENSP00000262367->ENSP00000269305->ENSP00000266970->ENSP00000309181 5

216.ENSP00000216797->ENSP00000359206->ENSP00000326804->ENSP00000274255->ENSP00000311083 4

217.ENSP00000216797->ENSP00000359206->ENSP00000344456->ENSP00000269571->ENSP00000339007->ENSP00000341189->ENSP00000228307->ENSP00000380227->ENSP00000364094->ENSP00000386896->ENSP00000200181->ENSP00000340937->ENSP00000348384->ENSP00000324532 19

218.ENSP00000216797->ENSP00000226574->ENSP00000327251 20

219.ENSP00000216797->ENSP00000384273->ENSP00000262367->ENSP00000269305->ENSP00000329623 4

220.ENSP00000216797->ENSP00000358622->ENSP00000316840->ENSP00000216160->ENSP00000347858->ENSP00000330237 5

221.ENSP00000216797->ENSP00000384273->ENSP00000263253->ENSP00000206249->ENSP00000399968->ENSP00000419692->ENSP00000332296 21

222.ENSP00000216797->ENSP00000359424->ENSP00000247668->ENSP00000227758->ENSP00000332468 4

223.ENSP00000216797->ENSP00000339151 1

224.ENSP00000216797->ENSP00000359206->ENSP00000344456->ENSP00000269571->ENSP00000339007->ENSP00000341189 5

225.ENSP00000216797->ENSP00000384273->ENSP00000262367->ENSP00000269305->ENSP00000293195->ENSP00000342087 37

226.ENSP00000216797->ENSP00000384273->ENSP00000263253->ENSP00000329357->ENSP00000345571 4

227.ENSP00000216797->ENSP00000384273->ENSP00000263253->ENSP00000262160->ENSP00000221930->ENSP00000346839 7

228.ENSP00000216797->ENSP00000358622->ENSP00000316840->ENSP00000216160->ENSP00000347858 4

229.ENSP00000216797->ENSP00000359206->ENSP00000344456->ENSP00000269571->ENSP00000339007->ENSP00000341189->ENSP00000228307->ENSP00000380227->ENSP00000364094->ENSP00000386896->ENSP00000200181->ENSP00000340937->ENSP00000348384 18

230.ENSP00000216797->ENSP00000384273->ENSP00000263253->ENSP00000367207->ENSP00000351490 4

231.ENSP00000216797->ENSP00000384273->ENSP00000263253->ENSP00000206249->ENSP00000270202->ENSP00000352121 11

232.ENSP00000216797->ENSP00000384273->ENSP00000263253->ENSP00000206249->ENSP00000327213->ENSP00000352900 18

233.ENSP00000216797->ENSP00000384273->ENSP00000262367->ENSP00000269305->ENSP00000353059 5

234.ENSP00000216797->ENSP00000359206->ENSP00000344456->ENSP00000269571->ENSP00000339007->ENSP00000341189->ENSP00000228307->ENSP00000380227->ENSP00000364094->ENSP00000282588->ENSP00000364979->ENSP00000353654 21

235.ENSP00000216797->ENSP00000384273->ENSP00000262367->ENSP00000269305->ENSP00000278916->ENSP00000321656->ENSP00000256442->ENSP00000342307->ENSP00000230538->ENSP00000354360 18

236.ENSP00000216797->ENSP00000384273->ENSP00000362649->ENSP00000267163->ENSP00000355249 4

237.ENSP00000216797->ENSP00000226574->ENSP00000263341->ENSP00000356438 7

238.ENSP00000216797->ENSP00000358622 1

239.ENSP00000216797->ENSP00000359424 1

240.ENSP00000216797->ENSP00000384273->ENSP00000262367->ENSP00000269305->ENSP00000361021 4

241.ENSP00000216797->ENSP00000384273->ENSP00000262367->ENSP00000269305->ENSP00000278916->ENSP00000312995->ENSP00000300093->ENSP00000263753->ENSP00000418447->ENSP00000348784->ENSP00000379866->ENSP00000379823->ENSP00000331902->ENSP00000361290 47

242.ENSP00000216797->ENSP00000226574->ENSP00000362994 4

243.ENSP00000216797->ENSP00000384273->ENSP00000263253->ENSP00000206249->ENSP00000327213->ENSP00000363812 30

244.ENSP00000216797->ENSP00000359206->ENSP00000344456->ENSP00000269571->ENSP00000339007->ENSP00000341189->ENSP00000228307->ENSP00000380227->ENSP00000364094 8

245.ENSP00000216797->ENSP00000359206->ENSP00000344456->ENSP00000269571->ENSP00000339007->ENSP00000341189->ENSP00000228307->ENSP00000380227->ENSP00000364094->ENSP00000282588->ENSP00000364979 20

246.ENSP00000216797->ENSP00000384273->ENSP00000262367->ENSP00000269305->ENSP00000361021->ENSP00000366563 16

247.ENSP00000216797->ENSP00000384273->ENSP00000263253->ENSP00000367207 3

248.ENSP00000216797->ENSP00000359206->ENSP00000344456->ENSP00000326366->ENSP00000284981->ENSP00000252486->ENSP00000252444->ENSP00000233242->ENSP00000236850->ENSP00000363868->ENSP00000372703 123

249.ENSP00000216797->ENSP00000384273->ENSP00000262367->ENSP00000269305->ENSP00000278916->ENSP00000321656->ENSP00000256442->ENSP00000342307->ENSP00000230538->ENSP00000222399->ENSP00000374309 26

250.ENSP00000216797->ENSP00000384273->ENSP00000263253->ENSP00000206249->ENSP00000270202->ENSP00000348986->ENSP00000375892 11

251.ENSP00000216797->ENSP00000384273->ENSP00000263253->ENSP00000264657->ENSP00000376765 6

252.ENSP00000216797->ENSP00000384273->ENSP00000262367->ENSP00000269305->ENSP00000278916->ENSP00000312995->ENSP00000300093->ENSP00000263753->ENSP00000418447->ENSP00000348784->ENSP00000379866 44

253.ENSP00000216797->ENSP00000384273->ENSP00000262367->ENSP00000269305->ENSP00000324897->ENSP00000381648 5

254.ENSP00000216797->ENSP00000384273 1

255.ENSP00000216797->ENSP00000359206->ENSP00000344456->ENSP00000269571->ENSP00000339007->ENSP00000341189->ENSP00000228307->ENSP00000380227->ENSP00000364094->ENSP00000386896 9

256.ENSP00000216797->ENSP00000359206->ENSP00000344456->ENSP00000326366->ENSP00000284981->ENSP00000252486->ENSP00000252444->ENSP00000233242->ENSP00000236850->ENSP00000363868->ENSP00000393286 123

257.ENSP00000216797->ENSP00000359206->ENSP00000344456->ENSP00000326366->ENSP00000284981->ENSP00000252486->ENSP00000252444->ENSP00000233242->ENSP00000236850->ENSP00000363868->ENSP00000393870 123

258.ENSP00000216797->ENSP00000384273->ENSP00000263253->ENSP00000262160->ENSP00000287727->ENSP00000400104 104

259.ENSP00000216797->ENSP00000359206->ENSP00000344456->ENSP00000269571->ENSP00000339007->ENSP00000312435->ENSP00000400365 18

260.ENSP00000216797->ENSP00000359206->ENSP00000344456->ENSP00000326366->ENSP00000284981->ENSP00000252486->ENSP00000252444->ENSP00000233242->ENSP00000236850->ENSP00000363868->ENSP00000402590 123

261.ENSP00000216797->ENSP00000384273->ENSP00000263253->ENSP00000206249->ENSP00000399968->ENSP00000419692 5

262.ENSP00000222254->ENSP00000304895->ENSP00000339007->ENSP00000312435->ENSP00000400365->ENSP00000222399 21

263.ENSP00000222254->ENSP00000304895->ENSP00000353483->ENSP00000269305->ENSP00000263253->ENSP00000384273->ENSP00000226574 6

264.ENSP00000222254->ENSP00000304895->ENSP00000268035->ENSP00000206249->ENSP00000227507 5

265.ENSP00000222254->ENSP00000304895->ENSP00000353483->ENSP00000269305->ENSP00000344818->ENSP00000259808->ENSP00000227758 7

266.ENSP00000222254->ENSP00000304895->ENSP00000348986->ENSP00000270202->ENSP00000228872 4

267.ENSP00000222254->ENSP00000304895->ENSP00000353483->ENSP00000269305->ENSP00000278916->ENSP00000321656->ENSP00000256442->ENSP00000342307->ENSP00000230538 17

268.ENSP00000222254->ENSP00000304895->ENSP00000353483->ENSP00000269305->ENSP00000344818->ENSP00000259808->ENSP00000247668 7

269.ENSP00000222254->ENSP00000304895->ENSP00000340944->ENSP00000354394->ENSP00000249636 5

270.ENSP00000222254->ENSP00000304895->ENSP00000339007->ENSP00000312435->ENSP00000400365->ENSP00000354360->ENSP00000252999 22

271.ENSP00000222254->ENSP00000304895->ENSP00000353483->ENSP00000269305->ENSP00000267163->ENSP00000257904 5

272.ENSP00000222254->ENSP00000304895->ENSP00000339007->ENSP00000312435->ENSP00000400365->ENSP00000258341 21

273.ENSP00000222254->ENSP00000264122->ENSP00000300574->ENSP00000228307->ENSP00000380227->ENSP00000346839->ENSP00000261023 6

274.ENSP00000222254->ENSP00000304895->ENSP00000353483->ENSP00000316840->ENSP00000269485->ENSP00000261464 9

275.ENSP00000222254->ENSP00000304895->ENSP00000401303->ENSP00000172229->ENSP00000262395 28

276.ENSP00000222254->ENSP00000264122->ENSP00000300574->ENSP00000228307->ENSP00000380227->ENSP00000346839->ENSP00000261023->ENSP00000262017->ENSP00000262407 8

277.ENSP00000222254->ENSP00000304895->ENSP00000353483->ENSP00000269305->ENSP00000266970->ENSP00000262643 5

278.ENSP00000222254->ENSP00000304895->ENSP00000262741 4

279.ENSP00000222254->ENSP00000304895->ENSP00000353483->ENSP00000269305->ENSP00000267163->ENSP00000262904 5

280.ENSP00000222254->ENSP00000304895->ENSP00000353483->ENSP00000360266->ENSP00000341551->ENSP00000262971 10

281.ENSP00000222254->ENSP00000304895->ENSP00000353483->ENSP00000316840->ENSP00000361359->ENSP00000263464 8

282.ENSP00000222254->ENSP00000261799->ENSP00000244007->ENSP00000263923->ENSP00000361125->ENSP00000263826 24

283.ENSP00000222254->ENSP00000304895->ENSP00000263967 2

284.ENSP00000222254->ENSP00000264122->ENSP00000300574->ENSP00000228307->ENSP00000380227->ENSP00000364094->ENSP00000386896->ENSP00000200181->ENSP00000340937->ENSP00000264144 21

285.ENSP00000222254->ENSP00000304895->ENSP00000353483->ENSP00000269305->ENSP00000355153->ENSP00000265734 5

286.ENSP00000222254->ENSP00000304895->ENSP00000353483->ENSP00000269305->ENSP00000266970 4

287.ENSP00000222254->ENSP00000304895->ENSP00000353483->ENSP00000269305->ENSP00000267163 4

288.ENSP00000222254->ENSP00000304895->ENSP00000348986->ENSP00000270202->ENSP00000352121->ENSP00000269300 12

289.ENSP00000222254->ENSP00000304895->ENSP00000353483->ENSP00000269305 3

290.ENSP00000222254->ENSP00000304895->ENSP00000348986->ENSP00000270202 3

291.ENSP00000222254->ENSP00000304895->ENSP00000348986->ENSP00000270202->ENSP00000228872->ENSP00000274255 5

292.ENSP00000222254->ENSP00000304895->ENSP00000274335 2

293.ENSP00000222254->ENSP00000304895->ENSP00000353483->ENSP00000269305->ENSP00000267163->ENSP00000257904->ENSP00000276925 6

294.ENSP00000222254->ENSP00000304895->ENSP00000274335->ENSP00000289153 6

295.ENSP00000222254->ENSP00000264122->ENSP00000300574->ENSP00000228307->ENSP00000380227->ENSP00000364094->ENSP00000296585 6

296.ENSP00000222254->ENSP00000304895->ENSP00000353483->ENSP00000269305->ENSP00000302564 5

297.ENSP00000222254->ENSP00000264122->ENSP00000364898->ENSP00000302269->ENSP00000348461->ENSP00000356505->ENSP00000220764->ENSP00000348380->ENSP00000305638 1021

298.ENSP00000222254->ENSP00000304895->ENSP00000339007->ENSP00000312435->ENSP00000400365->ENSP00000354360->ENSP00000252999->ENSP00000307156 27

299.ENSP00000222254->ENSP00000304895->ENSP00000353483->ENSP00000269305->ENSP00000353059->ENSP00000307786 11

300.ENSP00000222254->ENSP00000304895->ENSP00000353483->ENSP00000269305->ENSP00000266970->ENSP00000309181 5

301.ENSP00000222254->ENSP00000304895->ENSP00000353483->ENSP00000269305->ENSP00000266970->ENSP00000311083 5

302.ENSP00000222254->ENSP00000264122->ENSP00000300574->ENSP00000228307->ENSP00000380227->ENSP00000364094->ENSP00000386896->ENSP00000200181->ENSP00000340937->ENSP00000348384->ENSP00000324532 16

303.ENSP00000222254->ENSP00000304895->ENSP00000353483->ENSP00000269305->ENSP00000263253->ENSP00000384273->ENSP00000226574->ENSP00000327251 25

304.ENSP00000222254->ENSP00000304895->ENSP00000353483->ENSP00000269305->ENSP00000329623 4

305.ENSP00000222254->ENSP00000304895->ENSP00000353483->ENSP00000269305->ENSP00000353059->ENSP00000330237 6

306.ENSP00000222254->ENSP00000304895->ENSP00000268035->ENSP00000206249->ENSP00000399968->ENSP00000419692->ENSP00000332296 21

307.ENSP00000222254->ENSP00000304895->ENSP00000353483->ENSP00000316840->ENSP00000361359->ENSP00000332468 7

308.ENSP00000222254->ENSP00000304895->ENSP00000353483->ENSP00000269305->ENSP00000263253->ENSP00000384273->ENSP00000339151 6

309.ENSP00000222254->ENSP00000264122->ENSP00000300574->ENSP00000341189 3

310.ENSP00000222254->ENSP00000304895->ENSP00000353483->ENSP00000269305->ENSP00000293195->ENSP00000342087 37

311.ENSP00000222254->ENSP00000304895->ENSP00000353483->ENSP00000269305->ENSP00000267163->ENSP00000345571 5

312.ENSP00000222254->ENSP00000264122->ENSP00000300574->ENSP00000228307->ENSP00000380227->ENSP00000346839 5

313.ENSP00000222254->ENSP00000304895->ENSP00000353483->ENSP00000269305->ENSP00000355759->ENSP00000311032->ENSP00000347858 7

314.ENSP00000222254->ENSP00000264122->ENSP00000300574->ENSP00000228307->ENSP00000380227->ENSP00000364094->ENSP00000386896->ENSP00000200181->ENSP00000340937->ENSP00000348384 15

315.ENSP00000222254->ENSP00000304895->ENSP00000339007->ENSP00000215832->ENSP00000367207->ENSP00000351490 6

316.ENSP00000222254->ENSP00000304895->ENSP00000348986->ENSP00000270202->ENSP00000352121 9

317.ENSP00000222254->ENSP00000304895->ENSP00000268035->ENSP00000206249->ENSP00000327213->ENSP00000352900 18

318.ENSP00000222254->ENSP00000304895->ENSP00000353483->ENSP00000269305->ENSP00000353059 5

319.ENSP00000222254->ENSP00000264122->ENSP00000300574->ENSP00000228307->ENSP00000380227->ENSP00000364094->ENSP00000282588->ENSP00000364979->ENSP00000353654 18

320.ENSP00000222254->ENSP00000304895->ENSP00000339007->ENSP00000312435->ENSP00000400365->ENSP00000354360 17

321.ENSP00000222254->ENSP00000304895->ENSP00000353483->ENSP00000269305->ENSP00000267163->ENSP00000355249 5

322.ENSP00000222254->ENSP00000304895->ENSP00000353483->ENSP00000360266->ENSP00000356438 8

323.ENSP00000222254->ENSP00000304895->ENSP00000353483->ENSP00000316840->ENSP00000358622 6

324.ENSP00000222254->ENSP00000304895->ENSP00000353483->ENSP00000269305->ENSP00000263253->ENSP00000384273->ENSP00000359424 6

325.ENSP00000222254->ENSP00000304895->ENSP00000263967->ENSP00000361021 4

326.ENSP00000222254->ENSP00000304895->ENSP00000353483->ENSP00000269305->ENSP00000278916->ENSP00000312995->ENSP00000300093->ENSP00000263753->ENSP00000418447->ENSP00000348784->ENSP00000379866->ENSP00000379823->ENSP00000331902->ENSP00000361290 47

327.ENSP00000222254->ENSP00000304895->ENSP00000353483->ENSP00000316840->ENSP00000361359->ENSP00000362994 7

328.ENSP00000222254->ENSP00000304895->ENSP00000268035->ENSP00000206249->ENSP00000327213->ENSP00000363812 30

329.ENSP00000222254->ENSP00000264122->ENSP00000300574->ENSP00000228307->ENSP00000380227->ENSP00000364094 5

330.ENSP00000222254->ENSP00000264122->ENSP00000300574->ENSP00000228307->ENSP00000380227->ENSP00000364094->ENSP00000282588->ENSP00000364979 17

331.ENSP00000222254->ENSP00000366563 16

332.ENSP00000222254->ENSP00000304895->ENSP00000339007->ENSP00000215832->ENSP00000367207 5

333.ENSP00000222254->ENSP00000304895->ENSP00000268035->ENSP00000206249->ENSP00000254227->ENSP00000342470->ENSP00000363868->ENSP00000372703 123

334.ENSP00000222254->ENSP00000304895->ENSP00000339007->ENSP00000312435->ENSP00000400365->ENSP00000258341->ENSP00000374309 26

335.ENSP00000222254->ENSP00000304895->ENSP00000348986->ENSP00000375892 7

336.ENSP00000222254->ENSP00000304895->ENSP00000343204->ENSP00000264657->ENSP00000376765 6

337.ENSP00000222254->ENSP00000304895->ENSP00000353483->ENSP00000269305->ENSP00000278916->ENSP00000312995->ENSP00000300093->ENSP00000263753->ENSP00000418447->ENSP00000348784->ENSP00000379866 44

338.ENSP00000222254->ENSP00000304895->ENSP00000353483->ENSP00000269305->ENSP00000324897->ENSP00000381648 5

339.ENSP00000222254->ENSP00000304895->ENSP00000353483->ENSP00000269305->ENSP00000263253->ENSP00000384273 5

340.ENSP00000222254->ENSP00000264122->ENSP00000300574->ENSP00000228307->ENSP00000380227->ENSP00000364094->ENSP00000386896 6

341.ENSP00000222254->ENSP00000304895->ENSP00000268035->ENSP00000206249->ENSP00000254227->ENSP00000342470->ENSP00000363868->ENSP00000393286 123

342.ENSP00000222254->ENSP00000304895->ENSP00000268035->ENSP00000206249->ENSP00000254227->ENSP00000342470->ENSP00000363868->ENSP00000393870 123

343.ENSP00000222254->ENSP00000304895->ENSP00000353483->ENSP00000269305->ENSP00000263253->ENSP00000262160->ENSP00000287727->ENSP00000400104 106

344.ENSP00000222254->ENSP00000304895->ENSP00000339007->ENSP00000312435->ENSP00000400365 16

345.ENSP00000222254->ENSP00000304895->ENSP00000268035->ENSP00000206249->ENSP00000254227->ENSP00000342470->ENSP00000363868->ENSP00000402590 123

346.ENSP00000222254->ENSP00000304895->ENSP00000268035->ENSP00000206249->ENSP00000399968->ENSP00000419692 5

347.ENSP00000222399->ENSP00000230538->ENSP00000342307->ENSP00000256442->ENSP00000321656->ENSP00000278916->ENSP00000269305->ENSP00000362649->ENSP00000384273->ENSP00000226574 21

348.ENSP00000222399->ENSP00000230538->ENSP00000342307->ENSP00000306043->ENSP00000274026->ENSP00000228872->ENSP00000227507 19

349.ENSP00000222399->ENSP00000230538->ENSP00000342307->ENSP00000256442->ENSP00000321656->ENSP00000278916->ENSP00000269305->ENSP00000344818->ENSP00000259808->ENSP00000227758 22

350.ENSP00000222399->ENSP00000230538->ENSP00000342307->ENSP00000306043->ENSP00000274026->ENSP00000228872 18

351.ENSP00000222399->ENSP00000230538 4

352.ENSP00000222399->ENSP00000230538->ENSP00000342307->ENSP00000256442->ENSP00000321656->ENSP00000278916->ENSP00000269305->ENSP00000344818->ENSP00000259808->ENSP00000247668 22

353.ENSP00000222399->ENSP00000230538->ENSP00000342307->ENSP00000256442->ENSP00000321656->ENSP00000278916->ENSP00000269305->ENSP00000376076->ENSP00000249636 21

354.ENSP00000222399->ENSP00000252999 5

355.ENSP00000222399->ENSP00000230538->ENSP00000342307->ENSP00000306043->ENSP00000274026->ENSP00000228872->ENSP00000257904 19

356.ENSP00000222399->ENSP00000252999->ENSP00000258341 7

357.ENSP00000222399->ENSP00000400365->ENSP00000312435->ENSP00000339007->ENSP00000341189->ENSP00000228307->ENSP00000380227->ENSP00000346839->ENSP00000261023 24

358.ENSP00000222399->ENSP00000230538->ENSP00000342307->ENSP00000256442->ENSP00000321656->ENSP00000278916->ENSP00000269305->ENSP00000344818->ENSP00000316840->ENSP00000269485->ENSP00000261464 24

359.ENSP00000222399->ENSP00000230538->ENSP00000342307->ENSP00000306043->ENSP00000274026->ENSP00000345571->ENSP00000332643->ENSP00000172229->ENSP00000262395 45

360.ENSP00000222399->ENSP00000400365->ENSP00000312435->ENSP00000339007->ENSP00000341189->ENSP00000262017->ENSP00000262407 26

361.ENSP00000222399->ENSP00000230538->ENSP00000342307->ENSP00000306043->ENSP00000274026->ENSP00000228872->ENSP00000262643 19

362.ENSP00000222399->ENSP00000400365->ENSP00000312435->ENSP00000339007->ENSP00000304895->ENSP00000262741 23

363.ENSP00000222399->ENSP00000230538->ENSP00000342307->ENSP00000256442->ENSP00000321656->ENSP00000278916->ENSP00000269305->ENSP00000267163->ENSP00000262904 20

364.ENSP00000222399->ENSP00000230538->ENSP00000342307->ENSP00000256442->ENSP00000321656->ENSP00000278916->ENSP00000269305->ENSP00000329357->ENSP00000332973->ENSP00000341551->ENSP00000262971 25

365.ENSP00000222399->ENSP00000230538->ENSP00000342307->ENSP00000256442->ENSP00000321656->ENSP00000278916->ENSP00000269305->ENSP00000344818->ENSP00000316840->ENSP00000361359->ENSP00000263464 23

366.ENSP00000222399->ENSP00000230538->ENSP00000342307->ENSP00000256442->ENSP00000321656->ENSP00000278916->ENSP00000269305->ENSP00000338018->ENSP00000361125->ENSP00000263826 40

367.ENSP00000222399->ENSP00000230538->ENSP00000342307->ENSP00000256442->ENSP00000321656->ENSP00000278916->ENSP00000269305->ENSP00000361021->ENSP00000263967 21

368.ENSP00000222399->ENSP00000252999->ENSP00000264144 7

369.ENSP00000222399->ENSP00000230538->ENSP00000342307->ENSP00000306043->ENSP00000274026->ENSP00000228872->ENSP00000265734 19

370.ENSP00000222399->ENSP00000230538->ENSP00000342307->ENSP00000306043->ENSP00000274026->ENSP00000266970 18

371.ENSP00000222399->ENSP00000230538->ENSP00000342307->ENSP00000256442->ENSP00000321656->ENSP00000278916->ENSP00000269305->ENSP00000267163 19

372.ENSP00000222399->ENSP00000230538->ENSP00000342307->ENSP00000306043->ENSP00000274026->ENSP00000228872->ENSP00000270202->ENSP00000352121->ENSP00000269300 28

373.ENSP00000222399->ENSP00000230538->ENSP00000342307->ENSP00000256442->ENSP00000321656->ENSP00000278916->ENSP00000269305 18

374.ENSP00000222399->ENSP00000230538->ENSP00000342307->ENSP00000306043->ENSP00000274026->ENSP00000228872->ENSP00000270202 19

375.ENSP00000222399->ENSP00000230538->ENSP00000342307->ENSP00000306043->ENSP00000274026->ENSP00000228872->ENSP00000274255 19

376.ENSP00000222399->ENSP00000400365->ENSP00000312435->ENSP00000339007->ENSP00000274335 20

377.ENSP00000222399->ENSP00000230538->ENSP00000342307->ENSP00000306043->ENSP00000274026->ENSP00000228872->ENSP00000257904->ENSP00000276925 20

378.ENSP00000222399->ENSP00000230538->ENSP00000342307->ENSP00000306043->ENSP00000274026->ENSP00000228872->ENSP00000270202->ENSP00000289153 23

379.ENSP00000222399->ENSP00000400365->ENSP00000312435->ENSP00000339007->ENSP00000341189->ENSP00000228307->ENSP00000380227->ENSP00000364094->ENSP00000296585 24

380.ENSP00000222399->ENSP00000230538->ENSP00000342307->ENSP00000256442->ENSP00000321656->ENSP00000278916->ENSP00000269305->ENSP00000302564 20

381.ENSP00000222399->ENSP00000230538->ENSP00000342307->ENSP00000306043->ENSP00000274026->ENSP00000228872->ENSP00000270202->ENSP00000348461->ENSP00000356505->ENSP00000220764->ENSP00000348380->ENSP00000305638 1037

382.ENSP00000222399->ENSP00000252999->ENSP00000307156 10

383.ENSP00000222399->ENSP00000230538->ENSP00000342307->ENSP00000256442->ENSP00000321656->ENSP00000278916->ENSP00000269305->ENSP00000353059->ENSP00000307786 26

384.ENSP00000222399->ENSP00000230538->ENSP00000342307->ENSP00000306043->ENSP00000274026->ENSP00000266970->ENSP00000309181 19

385.ENSP00000222399->ENSP00000230538->ENSP00000342307->ENSP00000306043->ENSP00000274026->ENSP00000266970->ENSP00000311083 19

386.ENSP00000222399->ENSP00000252999->ENSP00000264144->ENSP00000324532 22

387.ENSP00000222399->ENSP00000230538->ENSP00000342307->ENSP00000256442->ENSP00000321656->ENSP00000278916->ENSP00000269305->ENSP00000362649->ENSP00000384273->ENSP00000226574->ENSP00000327251 40

388.ENSP00000222399->ENSP00000230538->ENSP00000342307->ENSP00000256442->ENSP00000321656->ENSP00000278916->ENSP00000269305->ENSP00000329623 19

389.ENSP00000222399->ENSP00000230538->ENSP00000342307->ENSP00000256442->ENSP00000321656->ENSP00000278916->ENSP00000269305->ENSP00000353059->ENSP00000330237 21

390.ENSP00000222399->ENSP00000230538->ENSP00000342307->ENSP00000256442->ENSP00000321656->ENSP00000372023->ENSP00000350283->ENSP00000206249->ENSP00000399968->ENSP00000419692->ENSP00000332296 37

391.ENSP00000222399->ENSP00000230538->ENSP00000342307->ENSP00000256442->ENSP00000321656->ENSP00000278916->ENSP00000269305->ENSP00000344818->ENSP00000316840->ENSP00000361359->ENSP00000332468 22

392.ENSP00000222399->ENSP00000230538->ENSP00000342307->ENSP00000256442->ENSP00000321656->ENSP00000278916->ENSP00000269305->ENSP00000362649->ENSP00000384273->ENSP00000339151 21

393.ENSP00000222399->ENSP00000400365->ENSP00000312435->ENSP00000339007->ENSP00000341189 20

394.ENSP00000222399->ENSP00000230538->ENSP00000342307->ENSP00000256442->ENSP00000321656->ENSP00000278916->ENSP00000269305->ENSP00000293195->ENSP00000342087 52

395.ENSP00000222399->ENSP00000230538->ENSP00000342307->ENSP00000306043->ENSP00000274026->ENSP00000345571 18

396.ENSP00000222399->ENSP00000400365->ENSP00000312435->ENSP00000339007->ENSP00000341189->ENSP00000228307->ENSP00000380227->ENSP00000346839 23

397.ENSP00000222399->ENSP00000230538->ENSP00000342307->ENSP00000256442->ENSP00000321656->ENSP00000278916->ENSP00000269305->ENSP00000329623->ENSP00000311032->ENSP00000347858 22

398.ENSP00000222399->ENSP00000252999->ENSP00000264144->ENSP00000324532->ENSP00000348384 23

399.ENSP00000222399->ENSP00000230538->ENSP00000342307->ENSP00000306043->ENSP00000274026->ENSP00000244741->ENSP00000367207->ENSP00000351490 21

400.ENSP00000222399->ENSP00000230538->ENSP00000342307->ENSP00000306043->ENSP00000274026->ENSP00000228872->ENSP00000270202->ENSP00000352121 25

401.ENSP00000222399->ENSP00000230538->ENSP00000342307->ENSP00000256442->ENSP00000321656->ENSP00000372023->ENSP00000350283->ENSP00000206249->ENSP00000327213->ENSP00000352900 34

402.ENSP00000222399->ENSP00000230538->ENSP00000342307->ENSP00000256442->ENSP00000321656->ENSP00000278916->ENSP00000269305->ENSP00000353059 20

403.ENSP00000222399->ENSP00000400365->ENSP00000312435->ENSP00000339007->ENSP00000341189->ENSP00000228307->ENSP00000380227->ENSP00000364094->ENSP00000282588->ENSP00000364979->ENSP00000353654 36

404.ENSP00000222399->ENSP00000230538->ENSP00000354360 5

405.ENSP00000222399->ENSP00000230538->ENSP00000342307->ENSP00000256442->ENSP00000321656->ENSP00000278916->ENSP00000269305->ENSP00000267163->ENSP00000355249 20

406.ENSP00000222399->ENSP00000230538->ENSP00000342307->ENSP00000256442->ENSP00000321656->ENSP00000278916->ENSP00000269305->ENSP00000344352->ENSP00000360266->ENSP00000356438 25

407.ENSP00000222399->ENSP00000230538->ENSP00000342307->ENSP00000256442->ENSP00000321656->ENSP00000278916->ENSP00000269305->ENSP00000344818->ENSP00000316840->ENSP00000358622 21

408.ENSP00000222399->ENSP00000230538->ENSP00000342307->ENSP00000256442->ENSP00000321656->ENSP00000278916->ENSP00000269305->ENSP00000362649->ENSP00000384273->ENSP00000359424 21

409.ENSP00000222399->ENSP00000230538->ENSP00000342307->ENSP00000256442->ENSP00000321656->ENSP00000278916->ENSP00000269305->ENSP00000361021 19

410.ENSP00000222399->ENSP00000230538->ENSP00000342307->ENSP00000256442->ENSP00000300093->ENSP00000263753->ENSP00000418447->ENSP00000348784->ENSP00000379866->ENSP00000379823->ENSP00000331902->ENSP00000361290 57

411.ENSP00000222399->ENSP00000230538->ENSP00000342307->ENSP00000256442->ENSP00000321656->ENSP00000278916->ENSP00000269305->ENSP00000344818->ENSP00000316840->ENSP00000361359->ENSP00000362994 22

412.ENSP00000222399->ENSP00000230538->ENSP00000342307->ENSP00000256442->ENSP00000321656->ENSP00000372023->ENSP00000350283->ENSP00000206249->ENSP00000327213->ENSP00000363812 46

413.ENSP00000222399->ENSP00000400365->ENSP00000312435->ENSP00000339007->ENSP00000341189->ENSP00000228307->ENSP00000380227->ENSP00000364094 23

414.ENSP00000222399->ENSP00000400365->ENSP00000312435->ENSP00000339007->ENSP00000341189->ENSP00000228307->ENSP00000380227->ENSP00000364094->ENSP00000282588->ENSP00000364979 35

415.ENSP00000222399->ENSP00000230538->ENSP00000342307->ENSP00000256442->ENSP00000321656->ENSP00000278916->ENSP00000269305->ENSP00000361021->ENSP00000366563 31

416.ENSP00000222399->ENSP00000230538->ENSP00000342307->ENSP00000306043->ENSP00000274026->ENSP00000244741->ENSP00000367207 20

417.ENSP00000222399->ENSP00000400365->ENSP00000312435->ENSP00000363827->ENSP00000252486->ENSP00000252444->ENSP00000233242->ENSP00000236850->ENSP00000363868->ENSP00000372703 139

418.ENSP00000222399->ENSP00000374309 5

419.ENSP00000222399->ENSP00000230538->ENSP00000342307->ENSP00000306043->ENSP00000274026->ENSP00000228872->ENSP00000270202->ENSP00000348986->ENSP00000375892 25

420.ENSP00000222399->ENSP00000230538->ENSP00000342307->ENSP00000256442->ENSP00000321656->ENSP00000278916->ENSP00000269305->ENSP00000263253->ENSP00000264657->ENSP00000376765 23

421.ENSP00000222399->ENSP00000230538->ENSP00000342307->ENSP00000256442->ENSP00000300093->ENSP00000263753->ENSP00000418447->ENSP00000348784->ENSP00000379866 54

422.ENSP00000222399->ENSP00000230538->ENSP00000342307->ENSP00000256442->ENSP00000321656->ENSP00000278916->ENSP00000269305->ENSP00000324897->ENSP00000381648 20

423.ENSP00000222399->ENSP00000230538->ENSP00000342307->ENSP00000256442->ENSP00000321656->ENSP00000278916->ENSP00000269305->ENSP00000362649->ENSP00000384273 20

424.ENSP00000222399->ENSP00000252999->ENSP00000264144->ENSP00000340937->ENSP00000200181->ENSP00000386896 22

425.ENSP00000222399->ENSP00000400365->ENSP00000312435->ENSP00000363827->ENSP00000252486->ENSP00000252444->ENSP00000233242->ENSP00000236850->ENSP00000363868->ENSP00000393286 139

426.ENSP00000222399->ENSP00000400365->ENSP00000312435->ENSP00000363827->ENSP00000252486->ENSP00000252444->ENSP00000233242->ENSP00000236850->ENSP00000363868->ENSP00000393870 139

427.ENSP00000222399->ENSP00000230538->ENSP00000342307->ENSP00000256442->ENSP00000321656->ENSP00000278916->ENSP00000269305->ENSP00000329357->ENSP00000332973->ENSP00000287727->ENSP00000400104 121

428.ENSP00000222399->ENSP00000400365 5

429.ENSP00000222399->ENSP00000400365->ENSP00000312435->ENSP00000363827->ENSP00000252486->ENSP00000252444->ENSP00000233242->ENSP00000236850->ENSP00000363868->ENSP00000402590 139

430.ENSP00000222399->ENSP00000230538->ENSP00000342307->ENSP00000256442->ENSP00000321656->ENSP00000372023->ENSP00000350283->ENSP00000206249->ENSP00000399968->ENSP00000419692 21

431.ENSP00000226574->ENSP00000216797->ENSP00000359206->ENSP00000344456->ENSP00000227507 4

432.ENSP00000226574->ENSP00000339151->ENSP00000162749->ENSP00000227758 3

433.ENSP00000226574->ENSP00000216797->ENSP00000359206->ENSP00000326804->ENSP00000274255->ENSP00000228872 5

434.ENSP00000226574->ENSP00000384273->ENSP00000262367->ENSP00000269305->ENSP00000278916->ENSP00000321656->ENSP00000256442->ENSP00000342307->ENSP00000230538 17

435.ENSP00000226574->ENSP00000359424->ENSP00000247668 2

436.ENSP00000226574->ENSP00000384273->ENSP00000263253->ENSP00000354394->ENSP00000249636 4

437.ENSP00000226574->ENSP00000384273->ENSP00000262367->ENSP00000269305->ENSP00000278916->ENSP00000321656->ENSP00000256442->ENSP00000342307->ENSP00000230538->ENSP00000354360->ENSP00000252999 23

438.ENSP00000226574->ENSP00000384273->ENSP00000362649->ENSP00000267163->ENSP00000257904 4

439.ENSP00000226574->ENSP00000384273->ENSP00000262367->ENSP00000269305->ENSP00000278916->ENSP00000321656->ENSP00000256442->ENSP00000342307->ENSP00000230538->ENSP00000354360->ENSP00000400365->ENSP00000258341 24

440.ENSP00000226574->ENSP00000361850->ENSP00000223095->ENSP00000226218->ENSP00000261023 8

441.ENSP00000226574->ENSP00000359424->ENSP00000247668->ENSP00000263932->ENSP00000261464 5

442.ENSP00000226574->ENSP00000384273->ENSP00000263253->ENSP00000206249->ENSP00000268035->ENSP00000401303->ENSP00000172229->ENSP00000262395 30

443.ENSP00000226574->ENSP00000361850->ENSP00000223095->ENSP00000226218->ENSP00000262017->ENSP00000262407 10

444.ENSP00000226574->ENSP00000384273->ENSP00000262367->ENSP00000269305->ENSP00000244741->ENSP00000262643 5

445.ENSP00000226574->ENSP00000384273->ENSP00000262367->ENSP00000269305->ENSP00000353483->ENSP00000304895->ENSP00000262741 8

446.ENSP00000226574->ENSP00000384273->ENSP00000362649->ENSP00000267163->ENSP00000262904 4

447.ENSP00000226574->ENSP00000384273->ENSP00000263253->ENSP00000262160->ENSP00000341551->ENSP00000262971 8

448.ENSP00000226574->ENSP00000359424->ENSP00000247668->ENSP00000263464 3

449.ENSP00000226574->ENSP00000384273->ENSP00000262367->ENSP00000338018->ENSP00000361125->ENSP00000263826 24

450.ENSP00000226574->ENSP00000384273->ENSP00000262367->ENSP00000269305->ENSP00000361021->ENSP00000263967 6

451.ENSP00000226574->ENSP00000264832->ENSP00000303242->ENSP00000367316->ENSP00000364094->ENSP00000386896->ENSP00000200181->ENSP00000340937->ENSP00000264144 24

452.ENSP00000226574->ENSP00000384273->ENSP00000362649->ENSP00000267163->ENSP00000265734 5

453.ENSP00000226574->ENSP00000384273->ENSP00000262367->ENSP00000269305->ENSP00000266970 4

454.ENSP00000226574->ENSP00000384273->ENSP00000362649->ENSP00000267163 3

455.ENSP00000226574->ENSP00000231509->ENSP00000335153->ENSP00000270202->ENSP00000352121->ENSP00000269300 14

456.ENSP00000226574->ENSP00000384273->ENSP00000262367->ENSP00000269305 3

457.ENSP00000226574->ENSP00000231509->ENSP00000335153->ENSP00000270202 5

458.ENSP00000226574->ENSP00000216797->ENSP00000359206->ENSP00000326804->ENSP00000274255 4

459.ENSP00000226574->ENSP00000216797->ENSP00000359206->ENSP00000344456->ENSP00000274335 6

460.ENSP00000226574->ENSP00000384273->ENSP00000362649->ENSP00000267163->ENSP00000257904->ENSP00000276925 5

461.ENSP00000226574->ENSP00000231509->ENSP00000335153->ENSP00000270202->ENSP00000289153 9

462.ENSP00000226574->ENSP00000264832->ENSP00000303242->ENSP00000367316->ENSP00000364094->ENSP00000296585 9

463.ENSP00000226574->ENSP00000384273->ENSP00000262367->ENSP00000269305->ENSP00000302564 5

464.ENSP00000226574->ENSP00000384273->ENSP00000263253->ENSP00000264657->ENSP00000348461->ENSP00000356505->ENSP00000220764->ENSP00000348380->ENSP00000305638 1022

465.ENSP00000226574->ENSP00000384273->ENSP00000262367->ENSP00000269305->ENSP00000278916->ENSP00000321656->ENSP00000256442->ENSP00000342307->ENSP00000230538->ENSP00000354360->ENSP00000252999->ENSP00000307156 28

466.ENSP00000226574->ENSP00000384273->ENSP00000262367->ENSP00000269305->ENSP00000353059->ENSP00000307786 11

467.ENSP00000226574->ENSP00000384273->ENSP00000262367->ENSP00000269305->ENSP00000266970->ENSP00000309181 5

468.ENSP00000226574->ENSP00000216797->ENSP00000359206->ENSP00000326804->ENSP00000274255->ENSP00000311083 5

469.ENSP00000226574->ENSP00000264832->ENSP00000303242->ENSP00000367316->ENSP00000364094->ENSP00000386896->ENSP00000200181->ENSP00000340937->ENSP00000348384->ENSP00000324532 19

470.ENSP00000226574->ENSP00000327251 19

471.ENSP00000226574->ENSP00000384273->ENSP00000262367->ENSP00000269305->ENSP00000329623 4

472.ENSP00000226574->ENSP00000384273->ENSP00000262367->ENSP00000269305->ENSP00000353059->ENSP00000330237 6

473.ENSP00000226574->ENSP00000231509->ENSP00000320940->ENSP00000348827->ENSP00000419692->ENSP00000332296 21

474.ENSP00000226574->ENSP00000339151->ENSP00000162749->ENSP00000227758->ENSP00000332468 4

475.ENSP00000226574->ENSP00000339151 1

476.ENSP00000226574->ENSP00000384273->ENSP00000263253->ENSP00000264657->ENSP00000350941->ENSP00000341189 5

477.ENSP00000226574->ENSP00000384273->ENSP00000262367->ENSP00000269305->ENSP00000293195->ENSP00000342087 37

478.ENSP00000226574->ENSP00000384273->ENSP00000362649->ENSP00000329357->ENSP00000345571 4

479.ENSP00000226574->ENSP00000384273->ENSP00000263253->ENSP00000262160->ENSP00000221930->ENSP00000346839 7

480.ENSP00000226574->ENSP00000359424->ENSP00000358622->ENSP00000316840->ENSP00000216160->ENSP00000347858 5

481.ENSP00000226574->ENSP00000264832->ENSP00000303242->ENSP00000367316->ENSP00000364094->ENSP00000386896->ENSP00000200181->ENSP00000340937->ENSP00000348384 18

482.ENSP00000226574->ENSP00000384273->ENSP00000263253->ENSP00000367207->ENSP00000351490 4

483.ENSP00000226574->ENSP00000231509->ENSP00000335153->ENSP00000270202->ENSP00000352121 11

484.ENSP00000226574->ENSP00000384273->ENSP00000263253->ENSP00000206249->ENSP00000327213->ENSP00000352900 18

485.ENSP00000226574->ENSP00000384273->ENSP00000262367->ENSP00000269305->ENSP00000353059 5

486.ENSP00000226574->ENSP00000264832->ENSP00000303242->ENSP00000367316->ENSP00000364094->ENSP00000282588->ENSP00000364979->ENSP00000353654 21

487.ENSP00000226574->ENSP00000384273->ENSP00000262367->ENSP00000269305->ENSP00000278916->ENSP00000321656->ENSP00000256442->ENSP00000342307->ENSP00000230538->ENSP00000354360 18

488.ENSP00000226574->ENSP00000384273->ENSP00000362649->ENSP00000267163->ENSP00000355249 4

489.ENSP00000226574->ENSP00000263341->ENSP00000356438 6

490.ENSP00000226574->ENSP00000359424->ENSP00000358622 2

491.ENSP00000226574->ENSP00000359424 1

492.ENSP00000226574->ENSP00000384273->ENSP00000262367->ENSP00000269305->ENSP00000361021 4

493.ENSP00000226574->ENSP00000384273->ENSP00000262367->ENSP00000269305->ENSP00000278916->ENSP00000321656->ENSP00000300093->ENSP00000263753->ENSP00000418447->ENSP00000348784->ENSP00000379866->ENSP00000379823->ENSP00000331902->ENSP00000361290 47

494.ENSP00000226574->ENSP00000362994 3

495.ENSP00000226574->ENSP00000384273->ENSP00000263253->ENSP00000206249->ENSP00000327213->ENSP00000363812 30

496.ENSP00000226574->ENSP00000264832->ENSP00000303242->ENSP00000367316->ENSP00000364094 8

497.ENSP00000226574->ENSP00000264832->ENSP00000303242->ENSP00000367316->ENSP00000364094->ENSP00000282588->ENSP00000364979 20

498.ENSP00000226574->ENSP00000384273->ENSP00000262367->ENSP00000269305->ENSP00000361021->ENSP00000366563 16

499.ENSP00000226574->ENSP00000384273->ENSP00000263253->ENSP00000367207 3

500.ENSP00000226574->ENSP00000384273->ENSP00000263253->ENSP00000206249->ENSP00000254227->ENSP00000342470->ENSP00000363868->ENSP00000372703 123

501.ENSP00000226574->ENSP00000384273->ENSP00000262367->ENSP00000269305->ENSP00000278916->ENSP00000321656->ENSP00000256442->ENSP00000342307->ENSP00000230538->ENSP00000222399->ENSP00000374309 26

502.ENSP00000226574->ENSP00000231509->ENSP00000335153->ENSP00000270202->ENSP00000348986->ENSP00000375892 11

503.ENSP00000226574->ENSP00000384273->ENSP00000263253->ENSP00000264657->ENSP00000376765 6

504.ENSP00000226574->ENSP00000384273->ENSP00000262367->ENSP00000269305->ENSP00000278916->ENSP00000321656->ENSP00000300093->ENSP00000263753->ENSP00000418447->ENSP00000348784->ENSP00000379866 44

505.ENSP00000226574->ENSP00000384273->ENSP00000262367->ENSP00000269305->ENSP00000324897->ENSP00000381648 5

506.ENSP00000226574->ENSP00000384273 1

507.ENSP00000226574->ENSP00000264832->ENSP00000303242->ENSP00000367316->ENSP00000364094->ENSP00000386896 9

508.ENSP00000226574->ENSP00000384273->ENSP00000263253->ENSP00000206249->ENSP00000254227->ENSP00000342470->ENSP00000363868->ENSP00000393286 123

509.ENSP00000226574->ENSP00000384273->ENSP00000263253->ENSP00000206249->ENSP00000254227->ENSP00000342470->ENSP00000363868->ENSP00000393870 123

510.ENSP00000226574->ENSP00000384273->ENSP00000263253->ENSP00000262160->ENSP00000287727->ENSP00000400104 104

511.ENSP00000226574->ENSP00000384273->ENSP00000262367->ENSP00000269305->ENSP00000278916->ENSP00000321656->ENSP00000256442->ENSP00000342307->ENSP00000230538->ENSP00000354360->ENSP00000400365 19

512.ENSP00000226574->ENSP00000384273->ENSP00000263253->ENSP00000206249->ENSP00000254227->ENSP00000342470->ENSP00000363868->ENSP00000402590 123

513.ENSP00000226574->ENSP00000231509->ENSP00000320940->ENSP00000348827->ENSP00000419692 5

514.ENSP00000227507->ENSP00000244741->ENSP00000344818->ENSP00000259808->ENSP00000227758 6

515.ENSP00000227507->ENSP00000228872 1

516.ENSP00000227507->ENSP00000345571->ENSP00000274026->ENSP00000306043->ENSP00000342307->ENSP00000230538 15

517.ENSP00000227507->ENSP00000344456->ENSP00000359206->ENSP00000189444->ENSP00000359424->ENSP00000247668 5

518.ENSP00000227507->ENSP00000264657->ENSP00000338799->ENSP00000354394->ENSP00000249636 5

519.ENSP00000227507->ENSP00000345571->ENSP00000274026->ENSP00000306043->ENSP00000342307->ENSP00000230538->ENSP00000354360->ENSP00000252999 21

520.ENSP00000227507->ENSP00000257904 1

521.ENSP00000227507->ENSP00000345571->ENSP00000274026->ENSP00000306043->ENSP00000342307->ENSP00000230538->ENSP00000354360->ENSP00000400365->ENSP00000258341 22

522.ENSP00000227507->ENSP00000228872->ENSP00000270202->ENSP00000299421->ENSP00000228307->ENSP00000380227->ENSP00000364094->ENSP00000261023 8

523.ENSP00000227507->ENSP00000244741->ENSP00000344818->ENSP00000316840->ENSP00000269485->ENSP00000261464 8

524.ENSP00000227507->ENSP00000345571->ENSP00000332643->ENSP00000172229->ENSP00000262395 28

525.ENSP00000227507->ENSP00000344456->ENSP00000269571->ENSP00000339007->ENSP00000341189->ENSP00000262017->ENSP00000262407 10

526.ENSP00000227507->ENSP00000244741->ENSP00000262643 2

527.ENSP00000227507->ENSP00000344456->ENSP00000269571->ENSP00000339007->ENSP00000304895->ENSP00000262741 7

528.ENSP00000227507->ENSP00000267163->ENSP00000262904 2

529.ENSP00000227507->ENSP00000345571->ENSP00000329357->ENSP00000332973->ENSP00000341551->ENSP00000262971 8

530.ENSP00000227507->ENSP00000344456->ENSP00000359206->ENSP00000189444->ENSP00000359424->ENSP00000247668->ENSP00000263464 6

531.ENSP00000227507->ENSP00000267163->ENSP00000269305->ENSP00000338018->ENSP00000361125->ENSP00000263826 24

532.ENSP00000227507->ENSP00000228872->ENSP00000270202->ENSP00000263967 4

533.ENSP00000227507->ENSP00000228872->ENSP00000270202->ENSP00000299421->ENSP00000228307->ENSP00000380227->ENSP00000364094->ENSP00000386896->ENSP00000200181->ENSP00000340937->ENSP00000264144 23

534.ENSP00000227507->ENSP00000265734 1

535.ENSP00000227507->ENSP00000266970 1

536.ENSP00000227507->ENSP00000267163 1

537.ENSP00000227507->ENSP00000228872->ENSP00000270202->ENSP00000352121->ENSP00000269300 11

538.ENSP00000227507->ENSP00000267163->ENSP00000269305 2

539.ENSP00000227507->ENSP00000228872->ENSP00000270202 2

540.ENSP00000227507->ENSP00000228872->ENSP00000274255 2

541.ENSP00000227507->ENSP00000344456->ENSP00000274335 4

542.ENSP00000227507->ENSP00000257904->ENSP00000276925 2

543.ENSP00000227507->ENSP00000228872->ENSP00000270202->ENSP00000289153 6

544.ENSP00000227507->ENSP00000228872->ENSP00000270202->ENSP00000299421->ENSP00000228307->ENSP00000380227->ENSP00000364094->ENSP00000296585 8

545.ENSP00000227507->ENSP00000267163->ENSP00000269305->ENSP00000302564 4

546.ENSP00000227507->ENSP00000228872->ENSP00000270202->ENSP00000348461->ENSP00000356505->ENSP00000220764->ENSP00000348380->ENSP00000305638 1020

547.ENSP00000227507->ENSP00000345571->ENSP00000274026->ENSP00000306043->ENSP00000342307->ENSP00000230538->ENSP00000354360->ENSP00000252999->ENSP00000307156 26

548.ENSP00000227507->ENSP00000267163->ENSP00000269305->ENSP00000353059->ENSP00000307786 10

549.ENSP00000227507->ENSP00000266970->ENSP00000309181 2

550.ENSP00000227507->ENSP00000266970->ENSP00000311083 2

551.ENSP00000227507->ENSP00000228872->ENSP00000270202->ENSP00000299421->ENSP00000228307->ENSP00000380227->ENSP00000364094->ENSP00000386896->ENSP00000200181->ENSP00000340937->ENSP00000348384->ENSP00000324532 18

552.ENSP00000227507->ENSP00000344456->ENSP00000359206->ENSP00000216797->ENSP00000226574->ENSP00000327251 23

553.ENSP00000227507->ENSP00000267163->ENSP00000269305->ENSP00000329623 3

554.ENSP00000227507->ENSP00000267163->ENSP00000269305->ENSP00000353059->ENSP00000330237 5

555.ENSP00000227507->ENSP00000206249->ENSP00000399968->ENSP00000419692->ENSP00000332296 20

556.ENSP00000227507->ENSP00000244741->ENSP00000344818->ENSP00000316840->ENSP00000361359->ENSP00000332468 6

557.ENSP00000227507->ENSP00000344456->ENSP00000359206->ENSP00000216797->ENSP00000339151 4

558.ENSP00000227507->ENSP00000344456->ENSP00000269571->ENSP00000339007->ENSP00000341189 4

559.ENSP00000227507->ENSP00000267163->ENSP00000269305->ENSP00000293195->ENSP00000342087 36

560.ENSP00000227507->ENSP00000345571 1

561.ENSP00000227507->ENSP00000344456->ENSP00000269571->ENSP00000265171->ENSP00000346839 7

562.ENSP00000227507->ENSP00000267163->ENSP00000269305->ENSP00000329623->ENSP00000311032->ENSP00000347858 6

563.ENSP00000227507->ENSP00000228872->ENSP00000270202->ENSP00000299421->ENSP00000228307->ENSP00000380227->ENSP00000364094->ENSP00000386896->ENSP00000200181->ENSP00000340937->ENSP00000348384 17

564.ENSP00000227507->ENSP00000345571->ENSP00000367207->ENSP00000351490 4

565.ENSP00000227507->ENSP00000228872->ENSP00000270202->ENSP00000352121 8

566.ENSP00000227507->ENSP00000206249->ENSP00000327213->ENSP00000352900 17

567.ENSP00000227507->ENSP00000267163->ENSP00000269305->ENSP00000353059 4

568.ENSP00000227507->ENSP00000228872->ENSP00000270202->ENSP00000299421->ENSP00000228307->ENSP00000380227->ENSP00000364094->ENSP00000282588->ENSP00000364979->ENSP00000353654 20

569.ENSP00000227507->ENSP00000345571->ENSP00000274026->ENSP00000306043->ENSP00000342307->ENSP00000230538->ENSP00000354360 16

570.ENSP00000227507->ENSP00000267163->ENSP00000355249 2

571.ENSP00000227507->ENSP00000264657->ENSP00000258743->ENSP00000356438 7

572.ENSP00000227507->ENSP00000344456->ENSP00000359206->ENSP00000216797->ENSP00000358622 4

573.ENSP00000227507->ENSP00000344456->ENSP00000359206->ENSP00000189444->ENSP00000359424 4

574.ENSP00000227507->ENSP00000267163->ENSP00000269305->ENSP00000361021 3

575.ENSP00000227507->ENSP00000345571->ENSP00000274026->ENSP00000306043->ENSP00000256442->ENSP00000300093->ENSP00000263753->ENSP00000418447->ENSP00000348784->ENSP00000379866->ENSP00000379823->ENSP00000331902->ENSP00000361290 46

576.ENSP00000227507->ENSP00000244741->ENSP00000344818->ENSP00000316840->ENSP00000361359->ENSP00000362994 6

577.ENSP00000227507->ENSP00000206249->ENSP00000327213->ENSP00000363812 29

578.ENSP00000227507->ENSP00000228872->ENSP00000270202->ENSP00000299421->ENSP00000228307->ENSP00000380227->ENSP00000364094 7

579.ENSP00000227507->ENSP00000228872->ENSP00000270202->ENSP00000299421->ENSP00000228307->ENSP00000380227->ENSP00000364094->ENSP00000282588->ENSP00000364979 19

580.ENSP00000227507->ENSP00000267163->ENSP00000269305->ENSP00000361021->ENSP00000366563 15

581.ENSP00000227507->ENSP00000345571->ENSP00000367207 3

582.ENSP00000227507->ENSP00000344456->ENSP00000326366->ENSP00000284981->ENSP00000252486->ENSP00000252444->ENSP00000233242->ENSP00000236850->ENSP00000363868->ENSP00000372703 122

583.ENSP00000227507->ENSP00000345571->ENSP00000274026->ENSP00000306043->ENSP00000342307->ENSP00000230538->ENSP00000222399->ENSP00000374309 24

584.ENSP00000227507->ENSP00000228872->ENSP00000270202->ENSP00000348986->ENSP00000375892 8

585.ENSP00000227507->ENSP00000264657->ENSP00000376765 5

586.ENSP00000227507->ENSP00000345571->ENSP00000274026->ENSP00000306043->ENSP00000256442->ENSP00000300093->ENSP00000263753->ENSP00000418447->ENSP00000348784->ENSP00000379866 43

587.ENSP00000227507->ENSP00000267163->ENSP00000269305->ENSP00000324897->ENSP00000381648 4

588.ENSP00000227507->ENSP00000267163->ENSP00000362649->ENSP00000384273 3

589.ENSP00000227507->ENSP00000228872->ENSP00000270202->ENSP00000299421->ENSP00000228307->ENSP00000380227->ENSP00000364094->ENSP00000386896 8

590.ENSP00000227507->ENSP00000344456->ENSP00000326366->ENSP00000284981->ENSP00000252486->ENSP00000252444->ENSP00000233242->ENSP00000236850->ENSP00000363868->ENSP00000393286 122

591.ENSP00000227507->ENSP00000344456->ENSP00000326366->ENSP00000284981->ENSP00000252486->ENSP00000252444->ENSP00000233242->ENSP00000236850->ENSP00000363868->ENSP00000393870 122

592.ENSP00000227507->ENSP00000345571->ENSP00000329357->ENSP00000332973->ENSP00000287727->ENSP00000400104 104

593.ENSP00000227507->ENSP00000345571->ENSP00000274026->ENSP00000306043->ENSP00000342307->ENSP00000230538->ENSP00000354360->ENSP00000400365 17

594.ENSP00000227507->ENSP00000344456->ENSP00000326366->ENSP00000284981->ENSP00000252486->ENSP00000252444->ENSP00000233242->ENSP00000236850->ENSP00000363868->ENSP00000402590 122

595.ENSP00000227507->ENSP00000206249->ENSP00000399968->ENSP00000419692 4

596.ENSP00000227758->ENSP00000259808->ENSP00000344818->ENSP00000269305->ENSP00000266970->ENSP00000228872 6

597.ENSP00000227758->ENSP00000259808->ENSP00000344818->ENSP00000269305->ENSP00000278916->ENSP00000321656->ENSP00000256442->ENSP00000342307->ENSP00000230538 18

598.ENSP00000227758->ENSP00000247668 1

599.ENSP00000227758->ENSP00000162749->ENSP00000339151->ENSP00000384273->ENSP00000263253->ENSP00000354394->ENSP00000249636 6

600.ENSP00000227758->ENSP00000259808->ENSP00000344818->ENSP00000269305->ENSP00000278916->ENSP00000321656->ENSP00000256442->ENSP00000342307->ENSP00000230538->ENSP00000354360->ENSP00000252999 24

601.ENSP00000227758->ENSP00000259808->ENSP00000344818->ENSP00000269305->ENSP00000267163->ENSP00000257904 6

602.ENSP00000227758->ENSP00000259808->ENSP00000344818->ENSP00000275493->ENSP00000339007->ENSP00000312435->ENSP00000400365->ENSP00000258341 24

603.ENSP00000227758->ENSP00000259808->ENSP00000344818->ENSP00000338018->ENSP00000361125->ENSP00000261023 10

604.ENSP00000227758->ENSP00000247668->ENSP00000263932->ENSP00000261464 4

605.ENSP00000227758->ENSP00000259808->ENSP00000344818->ENSP00000275493->ENSP00000401303->ENSP00000172229->ENSP00000262395 30

606.ENSP00000227758->ENSP00000259808->ENSP00000344818->ENSP00000275493->ENSP00000350941->ENSP00000341189->ENSP00000262017->ENSP00000262407 12

607.ENSP00000227758->ENSP00000259808->ENSP00000344818->ENSP00000269305->ENSP00000266970->ENSP00000262643 6

608.ENSP00000227758->ENSP00000259808->ENSP00000344818->ENSP00000275493->ENSP00000340944->ENSP00000304895->ENSP00000262741 9

609.ENSP00000227758->ENSP00000259808->ENSP00000344818->ENSP00000269305->ENSP00000267163->ENSP00000262904 6

610.ENSP00000227758->ENSP00000162749->ENSP00000339151->ENSP00000384273->ENSP00000263253->ENSP00000262160->ENSP00000341551->ENSP00000262971 10

611.ENSP00000227758->ENSP00000362994->ENSP00000263464 2

612.ENSP00000227758->ENSP00000259808->ENSP00000344818->ENSP00000338018->ENSP00000361125->ENSP00000263826 25

613.ENSP00000227758->ENSP00000259808->ENSP00000344818->ENSP00000275493->ENSP00000263967 7

614.ENSP00000227758->ENSP00000259808->ENSP00000344818->ENSP00000275493->ENSP00000300574->ENSP00000228307->ENSP00000380227->ENSP00000364094->ENSP00000386896->ENSP00000200181->ENSP00000340937->ENSP00000264144 25

615.ENSP00000227758->ENSP00000259808->ENSP00000344818->ENSP00000269305->ENSP00000355153->ENSP00000265734 6

616.ENSP00000227758->ENSP00000259808->ENSP00000344818->ENSP00000269305->ENSP00000266970 5

617.ENSP00000227758->ENSP00000259808->ENSP00000344818->ENSP00000269305->ENSP00000267163 5

618.ENSP00000227758->ENSP00000259808->ENSP00000344818->ENSP00000417281->ENSP00000270202->ENSP00000352121->ENSP00000269300 14

619.ENSP00000227758->ENSP00000259808->ENSP00000344818->ENSP00000269305 4

620.ENSP00000227758->ENSP00000259808->ENSP00000344818->ENSP00000417281->ENSP00000270202 5

621.ENSP00000227758->ENSP00000162749->ENSP00000339151->ENSP00000216797->ENSP00000359206->ENSP00000326804->ENSP00000274255 6

622.ENSP00000227758->ENSP00000259808->ENSP00000344818->ENSP00000275493->ENSP00000264033->ENSP00000274335 6

623.ENSP00000227758->ENSP00000259808->ENSP00000344818->ENSP00000269305->ENSP00000267163->ENSP00000257904->ENSP00000276925 7

624.ENSP00000227758->ENSP00000259808->ENSP00000344818->ENSP00000417281->ENSP00000270202->ENSP00000289153 9

625.ENSP00000227758->ENSP00000259808->ENSP00000344818->ENSP00000275493->ENSP00000300574->ENSP00000228307->ENSP00000380227->ENSP00000364094->ENSP00000296585 10

626.ENSP00000227758->ENSP00000259808->ENSP00000344818->ENSP00000269305->ENSP00000302564 6

627.ENSP00000227758->ENSP00000259808->ENSP00000344818->ENSP00000417281->ENSP00000270202->ENSP00000348461->ENSP00000356505->ENSP00000220764->ENSP00000348380->ENSP00000305638 1023

628.ENSP00000227758->ENSP00000259808->ENSP00000344818->ENSP00000269305->ENSP00000278916->ENSP00000321656->ENSP00000256442->ENSP00000342307->ENSP00000230538->ENSP00000354360->ENSP00000252999->ENSP00000307156 29

629.ENSP00000227758->ENSP00000267169->ENSP00000347858->ENSP00000330237->ENSP00000353059->ENSP00000307786 10

630.ENSP00000227758->ENSP00000259808->ENSP00000344818->ENSP00000269305->ENSP00000266970->ENSP00000309181 6

631.ENSP00000227758->ENSP00000259808->ENSP00000344818->ENSP00000269305->ENSP00000266970->ENSP00000311083 6

632.ENSP00000227758->ENSP00000259808->ENSP00000344818->ENSP00000275493->ENSP00000300574->ENSP00000228307->ENSP00000380227->ENSP00000364094->ENSP00000386896->ENSP00000200181->ENSP00000340937->ENSP00000348384->ENSP00000324532 20

633.ENSP00000227758->ENSP00000162749->ENSP00000339151->ENSP00000226574->ENSP00000327251 22

634.ENSP00000227758->ENSP00000259808->ENSP00000351273->ENSP00000329623 4

635.ENSP00000227758->ENSP00000267169->ENSP00000347858->ENSP00000330237 3

636.ENSP00000227758->ENSP00000162749->ENSP00000339151->ENSP00000384273->ENSP00000263253->ENSP00000206249->ENSP00000399968->ENSP00000419692->ENSP00000332296 23

637.ENSP00000227758->ENSP00000332468 1

638.ENSP00000227758->ENSP00000162749->ENSP00000339151 2

639.ENSP00000227758->ENSP00000259808->ENSP00000344818->ENSP00000275493->ENSP00000350941->ENSP00000341189 6

640.ENSP00000227758->ENSP00000259808->ENSP00000344818->ENSP00000269305->ENSP00000293195->ENSP00000342087 38

641.ENSP00000227758->ENSP00000259808->ENSP00000344818->ENSP00000269305->ENSP00000267163->ENSP00000345571 6

642.ENSP00000227758->ENSP00000259808->ENSP00000344818->ENSP00000275493->ENSP00000265171->ENSP00000346839 9

643.ENSP00000227758->ENSP00000267169->ENSP00000347858 2

644.ENSP00000227758->ENSP00000259808->ENSP00000344818->ENSP00000275493->ENSP00000300574->ENSP00000228307->ENSP00000380227->ENSP00000364094->ENSP00000386896->ENSP00000200181->ENSP00000340937->ENSP00000348384 19

645.ENSP00000227758->ENSP00000162749->ENSP00000339151->ENSP00000384273->ENSP00000263253->ENSP00000367207->ENSP00000351490 6

646.ENSP00000227758->ENSP00000259808->ENSP00000344818->ENSP00000417281->ENSP00000270202->ENSP00000352121 11

647.ENSP00000227758->ENSP00000162749->ENSP00000339151->ENSP00000384273->ENSP00000263253->ENSP00000206249->ENSP00000327213->ENSP00000352900 20

648.ENSP00000227758->ENSP00000267169->ENSP00000347858->ENSP00000330237->ENSP00000353059 4

649.ENSP00000227758->ENSP00000259808->ENSP00000344818->ENSP00000275493->ENSP00000300574->ENSP00000228307->ENSP00000380227->ENSP00000364094->ENSP00000282588->ENSP00000364979->ENSP00000353654 22

650.ENSP00000227758->ENSP00000259808->ENSP00000344818->ENSP00000269305->ENSP00000278916->ENSP00000321656->ENSP00000256442->ENSP00000342307->ENSP00000230538->ENSP00000354360 19

651.ENSP00000227758->ENSP00000259808->ENSP00000344818->ENSP00000269305->ENSP00000267163->ENSP00000355249 6

652.ENSP00000227758->ENSP00000247668->ENSP00000269485->ENSP00000316840->ENSP00000358997->ENSP00000263341->ENSP00000356438 8

653.ENSP00000227758->ENSP00000259808->ENSP00000358622 2

654.ENSP00000227758->ENSP00000247668->ENSP00000359424 2

655.ENSP00000227758->ENSP00000259808->ENSP00000344818->ENSP00000269305->ENSP00000361021 5

656.ENSP00000227758->ENSP00000259808->ENSP00000344818->ENSP00000269305->ENSP00000278916->ENSP00000312995->ENSP00000300093->ENSP00000263753->ENSP00000418447->ENSP00000348784->ENSP00000379866->ENSP00000379823->ENSP00000331902->ENSP00000361290 48

657.ENSP00000227758->ENSP00000362994 1

658.ENSP00000227758->ENSP00000162749->ENSP00000339151->ENSP00000384273->ENSP00000263253->ENSP00000206249->ENSP00000327213->ENSP00000363812 32

659.ENSP00000227758->ENSP00000259808->ENSP00000344818->ENSP00000275493->ENSP00000300574->ENSP00000228307->ENSP00000380227->ENSP00000364094 9

660.ENSP00000227758->ENSP00000259808->ENSP00000344818->ENSP00000275493->ENSP00000300574->ENSP00000228307->ENSP00000380227->ENSP00000364094->ENSP00000282588->ENSP00000364979 21

661.ENSP00000227758->ENSP00000259808->ENSP00000344818->ENSP00000269305->ENSP00000361021->ENSP00000366563 17

662.ENSP00000227758->ENSP00000162749->ENSP00000339151->ENSP00000384273->ENSP00000263253->ENSP00000367207 5

663.ENSP00000227758->ENSP00000162749->ENSP00000339151->ENSP00000384273->ENSP00000263253->ENSP00000206249->ENSP00000254227->ENSP00000342470->ENSP00000363868->ENSP00000372703 125

664.ENSP00000227758->ENSP00000259808->ENSP00000344818->ENSP00000269305->ENSP00000278916->ENSP00000321656->ENSP00000256442->ENSP00000342307->ENSP00000230538->ENSP00000222399->ENSP00000374309 27

665.ENSP00000227758->ENSP00000259808->ENSP00000344818->ENSP00000417281->ENSP00000270202->ENSP00000348986->ENSP00000375892 11

666.ENSP00000227758->ENSP00000259808->ENSP00000344818->ENSP00000275493->ENSP00000264657->ENSP00000376765 8

667.ENSP00000227758->ENSP00000259808->ENSP00000344818->ENSP00000269305->ENSP00000278916->ENSP00000312995->ENSP00000300093->ENSP00000263753->ENSP00000418447->ENSP00000348784->ENSP00000379866 45

668.ENSP00000227758->ENSP00000259808->ENSP00000344818->ENSP00000269305->ENSP00000324897->ENSP00000381648 6

669.ENSP00000227758->ENSP00000162749->ENSP00000339151->ENSP00000384273 3

670.ENSP00000227758->ENSP00000259808->ENSP00000344818->ENSP00000275493->ENSP00000300574->ENSP00000228307->ENSP00000380227->ENSP00000364094->ENSP00000386896 10

671.ENSP00000227758->ENSP00000162749->ENSP00000339151->ENSP00000384273->ENSP00000263253->ENSP00000206249->ENSP00000254227->ENSP00000342470->ENSP00000363868->ENSP00000393286 125

672.ENSP00000227758->ENSP00000162749->ENSP00000339151->ENSP00000384273->ENSP00000263253->ENSP00000206249->ENSP00000254227->ENSP00000342470->ENSP00000363868->ENSP00000393870 125

673.ENSP00000227758->ENSP00000162749->ENSP00000339151->ENSP00000384273->ENSP00000263253->ENSP00000262160->ENSP00000287727->ENSP00000400104 106

674.ENSP00000227758->ENSP00000259808->ENSP00000344818->ENSP00000275493->ENSP00000339007->ENSP00000312435->ENSP00000400365 19

675.ENSP00000227758->ENSP00000162749->ENSP00000339151->ENSP00000384273->ENSP00000263253->ENSP00000206249->ENSP00000254227->ENSP00000342470->ENSP00000363868->ENSP00000402590 125

676.ENSP00000227758->ENSP00000162749->ENSP00000339151->ENSP00000384273->ENSP00000263253->ENSP00000206249->ENSP00000399968->ENSP00000419692 7

677.ENSP00000228872->ENSP00000274026->ENSP00000306043->ENSP00000342307->ENSP00000230538 14

678.ENSP00000228872->ENSP00000266970->ENSP00000269305->ENSP00000344818->ENSP00000259808->ENSP00000247668 6

679.ENSP00000228872->ENSP00000266970->ENSP00000269305->ENSP00000376076->ENSP00000249636 5

680.ENSP00000228872->ENSP00000274026->ENSP00000306043->ENSP00000342307->ENSP00000230538->ENSP00000354360->ENSP00000252999 20

681.ENSP00000228872->ENSP00000257904 1

682.ENSP00000228872->ENSP00000274026->ENSP00000306043->ENSP00000342307->ENSP00000230538->ENSP00000354360->ENSP00000400365->ENSP00000258341 21

683.ENSP00000228872->ENSP00000270202->ENSP00000299421->ENSP00000228307->ENSP00000380227->ENSP00000364094->ENSP00000261023 7

684.ENSP00000228872->ENSP00000266970->ENSP00000269305->ENSP00000344818->ENSP00000316840->ENSP00000269485->ENSP00000261464 8

685.ENSP00000228872->ENSP00000270202->ENSP00000348986->ENSP00000303830->ENSP00000401303->ENSP00000172229->ENSP00000262395 29

686.ENSP00000228872->ENSP00000270202->ENSP00000299421->ENSP00000228307->ENSP00000380227->ENSP00000364094->ENSP00000261023->ENSP00000262017->ENSP00000262407 9

687.ENSP00000228872->ENSP00000262643 1

688.ENSP00000228872->ENSP00000270202->ENSP00000348986->ENSP00000304895->ENSP00000262741 6

689.ENSP00000228872->ENSP00000266970->ENSP00000267163->ENSP00000262904 3

690.ENSP00000228872->ENSP00000266970->ENSP00000269305->ENSP00000263253->ENSP00000262160->ENSP00000341551->ENSP00000262971 9

691.ENSP00000228872->ENSP00000266970->ENSP00000269305->ENSP00000344818->ENSP00000316840->ENSP00000361359->ENSP00000263464 7

692.ENSP00000228872->ENSP00000266970->ENSP00000269305->ENSP00000338018->ENSP00000361125->ENSP00000263826 24

693.ENSP00000228872->ENSP00000270202->ENSP00000263967 3

694.ENSP00000228872->ENSP00000270202->ENSP00000299421->ENSP00000228307->ENSP00000380227->ENSP00000364094->ENSP00000386896->ENSP00000200181->ENSP00000340937->ENSP00000264144 22

695.ENSP00000228872->ENSP00000265734 1

696.ENSP00000228872->ENSP00000266970 1

697.ENSP00000228872->ENSP00000266970->ENSP00000267163 2

698.ENSP00000228872->ENSP00000270202->ENSP00000352121->ENSP00000269300 10

699.ENSP00000228872->ENSP00000266970->ENSP00000269305 2

700.ENSP00000228872->ENSP00000270202 1

701.ENSP00000228872->ENSP00000274255 1

702.ENSP00000228872->ENSP00000270202->ENSP00000274335 4

703.ENSP00000228872->ENSP00000257904->ENSP00000276925 2

704.ENSP00000228872->ENSP00000270202->ENSP00000289153 5

705.ENSP00000228872->ENSP00000270202->ENSP00000299421->ENSP00000228307->ENSP00000380227->ENSP00000364094->ENSP00000296585 7

706.ENSP00000228872->ENSP00000270202->ENSP00000309103->ENSP00000302564 3

707.ENSP00000228872->ENSP00000270202->ENSP00000348461->ENSP00000356505->ENSP00000220764->ENSP00000348380->ENSP00000305638 1019

708.ENSP00000228872->ENSP00000274026->ENSP00000306043->ENSP00000342307->ENSP00000230538->ENSP00000354360->ENSP00000252999->ENSP00000307156 25

709.ENSP00000228872->ENSP00000266970->ENSP00000269305->ENSP00000353059->ENSP00000307786 10

710.ENSP00000228872->ENSP00000266970->ENSP00000309181 2

711.ENSP00000228872->ENSP00000274255->ENSP00000311083 2

712.ENSP00000228872->ENSP00000270202->ENSP00000299421->ENSP00000228307->ENSP00000380227->ENSP00000364094->ENSP00000386896->ENSP00000200181->ENSP00000340937->ENSP00000348384->ENSP00000324532 17

713.ENSP00000228872->ENSP00000266970->ENSP00000269305->ENSP00000263253->ENSP00000384273->ENSP00000226574->ENSP00000327251 24

714.ENSP00000228872->ENSP00000270202->ENSP00000309103->ENSP00000329623 3

715.ENSP00000228872->ENSP00000266970->ENSP00000269305->ENSP00000353059->ENSP00000330237 5

716.ENSP00000228872->ENSP00000270202->ENSP00000206249->ENSP00000399968->ENSP00000419692->ENSP00000332296 21

717.ENSP00000228872->ENSP00000266970->ENSP00000269305->ENSP00000344818->ENSP00000316840->ENSP00000361359->ENSP00000332468 6

718.ENSP00000228872->ENSP00000270202->ENSP00000298552->ENSP00000339151 5

719.ENSP00000228872->ENSP00000266970->ENSP00000269305->ENSP00000361021->ENSP00000341189 5

720.ENSP00000228872->ENSP00000266970->ENSP00000269305->ENSP00000293195->ENSP00000342087 36

721.ENSP00000228872->ENSP00000274026->ENSP00000345571 2

722.ENSP00000228872->ENSP00000270202->ENSP00000299421->ENSP00000228307->ENSP00000380227->ENSP00000346839 6

723.ENSP00000228872->ENSP00000266970->ENSP00000269305->ENSP00000355759->ENSP00000311032->ENSP00000347858 6

724.ENSP00000228872->ENSP00000270202->ENSP00000299421->ENSP00000228307->ENSP00000380227->ENSP00000364094->ENSP00000386896->ENSP00000200181->ENSP00000340937->ENSP00000348384 16

725.ENSP00000228872->ENSP00000274255->ENSP00000367207->ENSP00000351490 4

726.ENSP00000228872->ENSP00000270202->ENSP00000352121 7

727.ENSP00000228872->ENSP00000270202->ENSP00000206249->ENSP00000327213->ENSP00000352900 18

728.ENSP00000228872->ENSP00000266970->ENSP00000269305->ENSP00000353059 4

729.ENSP00000228872->ENSP00000270202->ENSP00000299421->ENSP00000228307->ENSP00000380227->ENSP00000364094->ENSP00000282588->ENSP00000364979->ENSP00000353654 19

730.ENSP00000228872->ENSP00000274026->ENSP00000306043->ENSP00000342307->ENSP00000230538->ENSP00000354360 15

731.ENSP00000228872->ENSP00000266970->ENSP00000267163->ENSP00000355249 3

732.ENSP00000228872->ENSP00000227507->ENSP00000264657->ENSP00000258743->ENSP00000356438 8

733.ENSP00000228872->ENSP00000266970->ENSP00000269305->ENSP00000344818->ENSP00000316840->ENSP00000358622 5

734.ENSP00000228872->ENSP00000266970->ENSP00000269305->ENSP00000263253->ENSP00000384273->ENSP00000359424 5

735.ENSP00000228872->ENSP00000266970->ENSP00000269305->ENSP00000361021 3

736.ENSP00000228872->ENSP00000274026->ENSP00000306043->ENSP00000256442->ENSP00000300093->ENSP00000263753->ENSP00000418447->ENSP00000348784->ENSP00000379866->ENSP00000379823->ENSP00000331902->ENSP00000361290 45

737.ENSP00000228872->ENSP00000266970->ENSP00000269305->ENSP00000344818->ENSP00000316840->ENSP00000361359->ENSP00000362994 6

738.ENSP00000228872->ENSP00000270202->ENSP00000206249->ENSP00000327213->ENSP00000363812 30

739.ENSP00000228872->ENSP00000270202->ENSP00000299421->ENSP00000228307->ENSP00000380227->ENSP00000364094 6

740.ENSP00000228872->ENSP00000270202->ENSP00000299421->ENSP00000228307->ENSP00000380227->ENSP00000364094->ENSP00000282588->ENSP00000364979 18

741.ENSP00000228872->ENSP00000266970->ENSP00000269305->ENSP00000361021->ENSP00000366563 15

742.ENSP00000228872->ENSP00000274255->ENSP00000367207 3

743.ENSP00000228872->ENSP00000227507->ENSP00000344456->ENSP00000326366->ENSP00000284981->ENSP00000252486->ENSP00000252444->ENSP00000233242->ENSP00000236850->ENSP00000363868->ENSP00000372703 123

744.ENSP00000228872->ENSP00000274026->ENSP00000306043->ENSP00000342307->ENSP00000230538->ENSP00000222399->ENSP00000374309 23

745.ENSP00000228872->ENSP00000270202->ENSP00000348986->ENSP00000375892 7

746.ENSP00000228872->ENSP00000227507->ENSP00000264657->ENSP00000376765 6

747.ENSP00000228872->ENSP00000274026->ENSP00000306043->ENSP00000256442->ENSP00000300093->ENSP00000263753->ENSP00000418447->ENSP00000348784->ENSP00000379866 42

748.ENSP00000228872->ENSP00000266970->ENSP00000269305->ENSP00000324897->ENSP00000381648 4

749.ENSP00000228872->ENSP00000266970->ENSP00000269305->ENSP00000263253->ENSP00000384273 4

750.ENSP00000228872->ENSP00000270202->ENSP00000299421->ENSP00000228307->ENSP00000380227->ENSP00000364094->ENSP00000386896 7

751.ENSP00000228872->ENSP00000227507->ENSP00000344456->ENSP00000326366->ENSP00000284981->ENSP00000252486->ENSP00000252444->ENSP00000233242->ENSP00000236850->ENSP00000363868->ENSP00000393286 123

752.ENSP00000228872->ENSP00000227507->ENSP00000344456->ENSP00000326366->ENSP00000284981->ENSP00000252486->ENSP00000252444->ENSP00000233242->ENSP00000236850->ENSP00000363868->ENSP00000393870 123

753.ENSP00000228872->ENSP00000266970->ENSP00000269305->ENSP00000263253->ENSP00000262160->ENSP00000287727->ENSP00000400104 105

754.ENSP00000228872->ENSP00000274026->ENSP00000306043->ENSP00000342307->ENSP00000230538->ENSP00000354360->ENSP00000400365 16

755.ENSP00000228872->ENSP00000227507->ENSP00000344456->ENSP00000326366->ENSP00000284981->ENSP00000252486->ENSP00000252444->ENSP00000233242->ENSP00000236850->ENSP00000363868->ENSP00000402590 123

756.ENSP00000228872->ENSP00000270202->ENSP00000206249->ENSP00000399968->ENSP00000419692 5

757.ENSP00000230538->ENSP00000342307->ENSP00000256442->ENSP00000321656->ENSP00000278916->ENSP00000269305->ENSP00000344818->ENSP00000259808->ENSP00000247668 18

758.ENSP00000230538->ENSP00000342307->ENSP00000256442->ENSP00000321656->ENSP00000278916->ENSP00000269305->ENSP00000376076->ENSP00000249636 17

759.ENSP00000230538->ENSP00000354360->ENSP00000252999 6

760.ENSP00000230538->ENSP00000342307->ENSP00000306043->ENSP00000255465->ENSP00000228872->ENSP00000257904 15

761.ENSP00000230538->ENSP00000354360->ENSP00000400365->ENSP00000258341 7

762.ENSP00000230538->ENSP00000342307->ENSP00000256442->ENSP00000321656->ENSP00000278916->ENSP00000269305->ENSP00000338018->ENSP00000361125->ENSP00000261023 21

763.ENSP00000230538->ENSP00000342307->ENSP00000256442->ENSP00000321656->ENSP00000278916->ENSP00000269305->ENSP00000344818->ENSP00000316840->ENSP00000269485->ENSP00000261464 20

764.ENSP00000230538->ENSP00000342307->ENSP00000306043->ENSP00000274026->ENSP00000345571->ENSP00000332643->ENSP00000172229->ENSP00000262395 41

765.ENSP00000230538->ENSP00000342307->ENSP00000256442->ENSP00000321656->ENSP00000278916->ENSP00000269305->ENSP00000361021->ENSP00000341189->ENSP00000262017->ENSP00000262407 23

766.ENSP00000230538->ENSP00000342307->ENSP00000306043->ENSP00000255465->ENSP00000228872->ENSP00000262643 15

767.ENSP00000230538->ENSP00000342307->ENSP00000256442->ENSP00000321656->ENSP00000278916->ENSP00000269305->ENSP00000353483->ENSP00000304895->ENSP00000262741 19

768.ENSP00000230538->ENSP00000342307->ENSP00000306043->ENSP00000274026->ENSP00000345571->ENSP00000267163->ENSP00000262904 16

769.ENSP00000230538->ENSP00000342307->ENSP00000256442->ENSP00000321656->ENSP00000278916->ENSP00000269305->ENSP00000263253->ENSP00000262160->ENSP00000341551->ENSP00000262971 21

770.ENSP00000230538->ENSP00000342307->ENSP00000256442->ENSP00000321656->ENSP00000278916->ENSP00000269305->ENSP00000344818->ENSP00000316840->ENSP00000361359->ENSP00000263464 19

771.ENSP00000230538->ENSP00000342307->ENSP00000256442->ENSP00000321656->ENSP00000278916->ENSP00000269305->ENSP00000338018->ENSP00000361125->ENSP00000263826 36

772.ENSP00000230538->ENSP00000342307->ENSP00000256442->ENSP00000321656->ENSP00000278916->ENSP00000269305->ENSP00000361021->ENSP00000263967 17

773.ENSP00000230538->ENSP00000354360->ENSP00000252999->ENSP00000264144 8

774.ENSP00000230538->ENSP00000342307->ENSP00000306043->ENSP00000255465->ENSP00000228872->ENSP00000265734 15

775.ENSP00000230538->ENSP00000342307->ENSP00000306043->ENSP00000255465->ENSP00000266970 14

776.ENSP00000230538->ENSP00000342307->ENSP00000306043->ENSP00000274026->ENSP00000345571->ENSP00000267163 15

777.ENSP00000230538->ENSP00000342307->ENSP00000306043->ENSP00000255465->ENSP00000228872->ENSP00000270202->ENSP00000352121->ENSP00000269300 24

778.ENSP00000230538->ENSP00000342307->ENSP00000256442->ENSP00000321656->ENSP00000278916->ENSP00000269305 14

779.ENSP00000230538->ENSP00000342307->ENSP00000306043->ENSP00000255465->ENSP00000228872->ENSP00000270202 15

780.ENSP00000230538->ENSP00000342307->ENSP00000306043->ENSP00000255465->ENSP00000228872->ENSP00000274255 15

781.ENSP00000230538->ENSP00000342307->ENSP00000256442->ENSP00000321656->ENSP00000278916->ENSP00000269305->ENSP00000353483->ENSP00000304895->ENSP00000274335 17

782.ENSP00000230538->ENSP00000342307->ENSP00000306043->ENSP00000255465->ENSP00000228872->ENSP00000257904->ENSP00000276925 16

783.ENSP00000230538->ENSP00000342307->ENSP00000306043->ENSP00000255465->ENSP00000228872->ENSP00000270202->ENSP00000289153 19

784.ENSP00000230538->ENSP00000342307->ENSP00000256442->ENSP00000321656->ENSP00000278916->ENSP00000269305->ENSP00000361021->ENSP00000341189->ENSP00000228307->ENSP00000380227->ENSP00000364094->ENSP00000296585 21

785.ENSP00000230538->ENSP00000342307->ENSP00000256442->ENSP00000321656->ENSP00000278916->ENSP00000269305->ENSP00000302564 16

786.ENSP00000230538->ENSP00000342307->ENSP00000306043->ENSP00000255465->ENSP00000228872->ENSP00000270202->ENSP00000348461->ENSP00000356505->ENSP00000220764->ENSP00000348380->ENSP00000305638 1033

787.ENSP00000230538->ENSP00000354360->ENSP00000252999->ENSP00000307156 11

788.ENSP00000230538->ENSP00000342307->ENSP00000256442->ENSP00000321656->ENSP00000278916->ENSP00000269305->ENSP00000353059->ENSP00000307786 22

789.ENSP00000230538->ENSP00000342307->ENSP00000306043->ENSP00000255465->ENSP00000266970->ENSP00000309181 15

790.ENSP00000230538->ENSP00000342307->ENSP00000306043->ENSP00000255465->ENSP00000266970->ENSP00000311083 15

791.ENSP00000230538->ENSP00000354360->ENSP00000252999->ENSP00000264144->ENSP00000324532 23

792.ENSP00000230538->ENSP00000342307->ENSP00000256442->ENSP00000321656->ENSP00000278916->ENSP00000269305->ENSP00000262367->ENSP00000384273->ENSP00000226574->ENSP00000327251 36

793.ENSP00000230538->ENSP00000342307->ENSP00000256442->ENSP00000321656->ENSP00000278916->ENSP00000269305->ENSP00000329623 15

794.ENSP00000230538->ENSP00000342307->ENSP00000256442->ENSP00000321656->ENSP00000278916->ENSP00000269305->ENSP00000353059->ENSP00000330237 17

795.ENSP00000230538->ENSP00000342307->ENSP00000256442->ENSP00000321656->ENSP00000372023->ENSP00000350283->ENSP00000206249->ENSP00000399968->ENSP00000419692->ENSP00000332296 33

796.ENSP00000230538->ENSP00000342307->ENSP00000256442->ENSP00000321656->ENSP00000278916->ENSP00000269305->ENSP00000344818->ENSP00000316840->ENSP00000361359->ENSP00000332468 18

797.ENSP00000230538->ENSP00000342307->ENSP00000256442->ENSP00000321656->ENSP00000278916->ENSP00000269305->ENSP00000262367->ENSP00000384273->ENSP00000339151 17

798.ENSP00000230538->ENSP00000342307->ENSP00000256442->ENSP00000321656->ENSP00000278916->ENSP00000269305->ENSP00000361021->ENSP00000341189 17

799.ENSP00000230538->ENSP00000342307->ENSP00000256442->ENSP00000321656->ENSP00000278916->ENSP00000269305->ENSP00000293195->ENSP00000342087 48

800.ENSP00000230538->ENSP00000342307->ENSP00000306043->ENSP00000274026->ENSP00000345571 14

801.ENSP00000230538->ENSP00000342307->ENSP00000256442->ENSP00000321656->ENSP00000278916->ENSP00000269305->ENSP00000263253->ENSP00000262160->ENSP00000221930->ENSP00000346839 20

802.ENSP00000230538->ENSP00000342307->ENSP00000256442->ENSP00000321656->ENSP00000278916->ENSP00000269305->ENSP00000329623->ENSP00000311032->ENSP00000347858 18

803.ENSP00000230538->ENSP00000354360->ENSP00000252999->ENSP00000264144->ENSP00000324532->ENSP00000348384 24

804.ENSP00000230538->ENSP00000342307->ENSP00000306043->ENSP00000274026->ENSP00000345571->ENSP00000367207->ENSP00000351490 17

805.ENSP00000230538->ENSP00000342307->ENSP00000306043->ENSP00000255465->ENSP00000228872->ENSP00000270202->ENSP00000352121 21

806.ENSP00000230538->ENSP00000342307->ENSP00000256442->ENSP00000321656->ENSP00000372023->ENSP00000350283->ENSP00000206249->ENSP00000327213->ENSP00000352900 30

807.ENSP00000230538->ENSP00000342307->ENSP00000256442->ENSP00000321656->ENSP00000278916->ENSP00000269305->ENSP00000353059 16

808.ENSP00000230538->ENSP00000342307->ENSP00000256442->ENSP00000321656->ENSP00000278916->ENSP00000269305->ENSP00000361021->ENSP00000341189->ENSP00000228307->ENSP00000380227->ENSP00000364094->ENSP00000282588->ENSP00000364979->ENSP00000353654 33

809.ENSP00000230538->ENSP00000354360 1

810.ENSP00000230538->ENSP00000342307->ENSP00000306043->ENSP00000274026->ENSP00000345571->ENSP00000267163->ENSP00000355249 16

811.ENSP00000230538->ENSP00000342307->ENSP00000256442->ENSP00000321656->ENSP00000278916->ENSP00000269305->ENSP00000353483->ENSP00000360266->ENSP00000356438 21

812.ENSP00000230538->ENSP00000342307->ENSP00000256442->ENSP00000321656->ENSP00000278916->ENSP00000269305->ENSP00000344818->ENSP00000316840->ENSP00000358622 17

813.ENSP00000230538->ENSP00000342307->ENSP00000256442->ENSP00000321656->ENSP00000278916->ENSP00000269305->ENSP00000262367->ENSP00000384273->ENSP00000359424 17

814.ENSP00000230538->ENSP00000342307->ENSP00000256442->ENSP00000321656->ENSP00000278916->ENSP00000269305->ENSP00000361021 15

815.ENSP00000230538->ENSP00000342307->ENSP00000256442->ENSP00000300093->ENSP00000263753->ENSP00000418447->ENSP00000348784->ENSP00000379866->ENSP00000379823->ENSP00000331902->ENSP00000361290 53

816.ENSP00000230538->ENSP00000342307->ENSP00000256442->ENSP00000321656->ENSP00000278916->ENSP00000269305->ENSP00000344818->ENSP00000316840->ENSP00000361359->ENSP00000362994 18

817.ENSP00000230538->ENSP00000342307->ENSP00000256442->ENSP00000321656->ENSP00000372023->ENSP00000350283->ENSP00000206249->ENSP00000327213->ENSP00000363812 42

818.ENSP00000230538->ENSP00000342307->ENSP00000256442->ENSP00000321656->ENSP00000278916->ENSP00000269305->ENSP00000361021->ENSP00000341189->ENSP00000228307->ENSP00000380227->ENSP00000364094 20

819.ENSP00000230538->ENSP00000342307->ENSP00000256442->ENSP00000321656->ENSP00000278916->ENSP00000269305->ENSP00000361021->ENSP00000341189->ENSP00000228307->ENSP00000380227->ENSP00000364094->ENSP00000282588->ENSP00000364979 32

820.ENSP00000230538->ENSP00000342307->ENSP00000256442->ENSP00000321656->ENSP00000278916->ENSP00000269305->ENSP00000361021->ENSP00000366563 27

821.ENSP00000230538->ENSP00000342307->ENSP00000306043->ENSP00000274026->ENSP00000345571->ENSP00000367207 16

822.ENSP00000230538->ENSP00000342307->ENSP00000256442->ENSP00000321656->ENSP00000372023->ENSP00000350283->ENSP00000206249->ENSP00000254227->ENSP00000342470->ENSP00000363868->ENSP00000372703 135

823.ENSP00000230538->ENSP00000222399->ENSP00000374309 9

824.ENSP00000230538->ENSP00000342307->ENSP00000306043->ENSP00000255465->ENSP00000228872->ENSP00000270202->ENSP00000348986->ENSP00000375892 21

825.ENSP00000230538->ENSP00000342307->ENSP00000256442->ENSP00000321656->ENSP00000278916->ENSP00000269305->ENSP00000263253->ENSP00000264657->ENSP00000376765 19

826.ENSP00000230538->ENSP00000342307->ENSP00000256442->ENSP00000300093->ENSP00000263753->ENSP00000418447->ENSP00000348784->ENSP00000379866 50

827.ENSP00000230538->ENSP00000342307->ENSP00000256442->ENSP00000321656->ENSP00000278916->ENSP00000269305->ENSP00000324897->ENSP00000381648 16

828.ENSP00000230538->ENSP00000342307->ENSP00000256442->ENSP00000321656->ENSP00000278916->ENSP00000269305->ENSP00000262367->ENSP00000384273 16

829.ENSP00000230538->ENSP00000386896 21

830.ENSP00000230538->ENSP00000342307->ENSP00000256442->ENSP00000321656->ENSP00000372023->ENSP00000350283->ENSP00000206249->ENSP00000254227->ENSP00000342470->ENSP00000363868->ENSP00000393286 135

831.ENSP00000230538->ENSP00000342307->ENSP00000256442->ENSP00000321656->ENSP00000372023->ENSP00000350283->ENSP00000206249->ENSP00000254227->ENSP00000342470->ENSP00000363868->ENSP00000393870 135

832.ENSP00000230538->ENSP00000342307->ENSP00000256442->ENSP00000321656->ENSP00000278916->ENSP00000269305->ENSP00000263253->ENSP00000262160->ENSP00000287727->ENSP00000400104 117

833.ENSP00000230538->ENSP00000354360->ENSP00000400365 2

834.ENSP00000230538->ENSP00000342307->ENSP00000256442->ENSP00000321656->ENSP00000372023->ENSP00000350283->ENSP00000206249->ENSP00000254227->ENSP00000342470->ENSP00000363868->ENSP00000402590 135

835.ENSP00000230538->ENSP00000342307->ENSP00000256442->ENSP00000321656->ENSP00000372023->ENSP00000350283->ENSP00000206249->ENSP00000399968->ENSP00000419692 17

836.ENSP00000247668->ENSP00000359424->ENSP00000384273->ENSP00000263253->ENSP00000354394->ENSP00000249636 5

837.ENSP00000247668->ENSP00000259808->ENSP00000344818->ENSP00000269305->ENSP00000278916->ENSP00000321656->ENSP00000256442->ENSP00000342307->ENSP00000230538->ENSP00000354360->ENSP00000252999 24

838.ENSP00000247668->ENSP00000359424->ENSP00000384273->ENSP00000362649->ENSP00000267163->ENSP00000257904 5

839.ENSP00000247668->ENSP00000259808->ENSP00000344818->ENSP00000275493->ENSP00000339007->ENSP00000312435->ENSP00000400365->ENSP00000258341 24

840.ENSP00000247668->ENSP00000359424->ENSP00000384273->ENSP00000263253->ENSP00000262160->ENSP00000221930->ENSP00000346839->ENSP00000261023 9

841.ENSP00000247668->ENSP00000263932->ENSP00000261464 3

842.ENSP00000247668->ENSP00000259808->ENSP00000344818->ENSP00000275493->ENSP00000401303->ENSP00000172229->ENSP00000262395 30

843.ENSP00000247668->ENSP00000359424->ENSP00000384273->ENSP00000263253->ENSP00000262160->ENSP00000221930->ENSP00000346839->ENSP00000261023->ENSP00000262017->ENSP00000262407 11

844.ENSP00000247668->ENSP00000359424->ENSP00000384273->ENSP00000263253->ENSP00000367207->ENSP00000281708->ENSP00000262643 6

845.ENSP00000247668->ENSP00000359424->ENSP00000384273->ENSP00000263253->ENSP00000264657->ENSP00000343204->ENSP00000304895->ENSP00000262741 9

846.ENSP00000247668->ENSP00000359424->ENSP00000384273->ENSP00000362649->ENSP00000267163->ENSP00000262904 5

847.ENSP00000247668->ENSP00000359424->ENSP00000384273->ENSP00000263253->ENSP00000262160->ENSP00000341551->ENSP00000262971 9

848.ENSP00000247668->ENSP00000263464 1

849.ENSP00000247668->ENSP00000259808->ENSP00000344818->ENSP00000338018->ENSP00000361125->ENSP00000263826 25

850.ENSP00000247668->ENSP00000259808->ENSP00000344818->ENSP00000275493->ENSP00000263967 7

851.ENSP00000247668->ENSP00000259808->ENSP00000344818->ENSP00000275493->ENSP00000300574->ENSP00000228307->ENSP00000380227->ENSP00000364094->ENSP00000386896->ENSP00000200181->ENSP00000340937->ENSP00000264144 25

852.ENSP00000247668->ENSP00000359424->ENSP00000384273->ENSP00000362649->ENSP00000267163->ENSP00000265734 6

853.ENSP00000247668->ENSP00000359424->ENSP00000384273->ENSP00000362649->ENSP00000267163->ENSP00000266970 5

854.ENSP00000247668->ENSP00000359424->ENSP00000384273->ENSP00000362649->ENSP00000267163 4

855.ENSP00000247668->ENSP00000259808->ENSP00000344818->ENSP00000417281->ENSP00000270202->ENSP00000352121->ENSP00000269300 14

856.ENSP00000247668->ENSP00000259808->ENSP00000344818->ENSP00000269305 4

857.ENSP00000247668->ENSP00000259808->ENSP00000344818->ENSP00000417281->ENSP00000270202 5

858.ENSP00000247668->ENSP00000359424->ENSP00000189444->ENSP00000359206->ENSP00000231487->ENSP00000274255 5

859.ENSP00000247668->ENSP00000259808->ENSP00000344818->ENSP00000275493->ENSP00000264033->ENSP00000274335 6

860.ENSP00000247668->ENSP00000359424->ENSP00000384273->ENSP00000362649->ENSP00000267163->ENSP00000257904->ENSP00000276925 6

861.ENSP00000247668->ENSP00000259808->ENSP00000344818->ENSP00000417281->ENSP00000270202->ENSP00000289153 9

862.ENSP00000247668->ENSP00000259808->ENSP00000344818->ENSP00000275493->ENSP00000300574->ENSP00000228307->ENSP00000380227->ENSP00000364094->ENSP00000296585 10

863.ENSP00000247668->ENSP00000259808->ENSP00000344818->ENSP00000269305->ENSP00000302564 6

864.ENSP00000247668->ENSP00000359424->ENSP00000384273->ENSP00000263253->ENSP00000264657->ENSP00000348461->ENSP00000356505->ENSP00000220764->ENSP00000348380->ENSP00000305638 1023

865.ENSP00000247668->ENSP00000259808->ENSP00000344818->ENSP00000269305->ENSP00000278916->ENSP00000321656->ENSP00000256442->ENSP00000342307->ENSP00000230538->ENSP00000354360->ENSP00000252999->ENSP00000307156 29

866.ENSP00000247668->ENSP00000227758->ENSP00000267169->ENSP00000347858->ENSP00000330237->ENSP00000353059->ENSP00000307786 11

867.ENSP00000247668->ENSP00000359424->ENSP00000384273->ENSP00000362649->ENSP00000267163->ENSP00000266970->ENSP00000309181 6

868.ENSP00000247668->ENSP00000359424->ENSP00000189444->ENSP00000359206->ENSP00000231487->ENSP00000274255->ENSP00000311083 6

869.ENSP00000247668->ENSP00000259808->ENSP00000344818->ENSP00000275493->ENSP00000300574->ENSP00000228307->ENSP00000380227->ENSP00000364094->ENSP00000386896->ENSP00000200181->ENSP00000340937->ENSP00000348384->ENSP00000324532 20

870.ENSP00000247668->ENSP00000359424->ENSP00000226574->ENSP00000327251 21

871.ENSP00000247668->ENSP00000259808->ENSP00000351273->ENSP00000329623 4

872.ENSP00000247668->ENSP00000227758->ENSP00000267169->ENSP00000347858->ENSP00000330237 4

873.ENSP00000247668->ENSP00000359424->ENSP00000384273->ENSP00000263253->ENSP00000325690->ENSP00000399968->ENSP00000419692->ENSP00000332296 22

874.ENSP00000247668->ENSP00000361359->ENSP00000332468 2

875.ENSP00000247668->ENSP00000339151 2

876.ENSP00000247668->ENSP00000359424->ENSP00000384273->ENSP00000263253->ENSP00000264657->ENSP00000350941->ENSP00000341189 6

877.ENSP00000247668->ENSP00000259808->ENSP00000344818->ENSP00000269305->ENSP00000293195->ENSP00000342087 38

878.ENSP00000247668->ENSP00000359424->ENSP00000384273->ENSP00000362649->ENSP00000267163->ENSP00000345571 5

879.ENSP00000247668->ENSP00000359424->ENSP00000384273->ENSP00000263253->ENSP00000262160->ENSP00000221930->ENSP00000346839 8

880.ENSP00000247668->ENSP00000227758->ENSP00000267169->ENSP00000347858 3

881.ENSP00000247668->ENSP00000259808->ENSP00000344818->ENSP00000275493->ENSP00000300574->ENSP00000228307->ENSP00000380227->ENSP00000364094->ENSP00000386896->ENSP00000200181->ENSP00000340937->ENSP00000348384 19

882.ENSP00000247668->ENSP00000359424->ENSP00000384273->ENSP00000263253->ENSP00000367207->ENSP00000351490 5

883.ENSP00000247668->ENSP00000259808->ENSP00000344818->ENSP00000417281->ENSP00000270202->ENSP00000352121 11

884.ENSP00000247668->ENSP00000359424->ENSP00000384273->ENSP00000263253->ENSP00000206249->ENSP00000327213->ENSP00000352900 19

885.ENSP00000247668->ENSP00000227758->ENSP00000267169->ENSP00000347858->ENSP00000330237->ENSP00000353059 5

886.ENSP00000247668->ENSP00000259808->ENSP00000344818->ENSP00000275493->ENSP00000300574->ENSP00000228307->ENSP00000380227->ENSP00000364094->ENSP00000282588->ENSP00000364979->ENSP00000353654 22

887.ENSP00000247668->ENSP00000259808->ENSP00000344818->ENSP00000269305->ENSP00000278916->ENSP00000321656->ENSP00000256442->ENSP00000342307->ENSP00000230538->ENSP00000354360 19

888.ENSP00000247668->ENSP00000359424->ENSP00000384273->ENSP00000362649->ENSP00000267163->ENSP00000355249 5

889.ENSP00000247668->ENSP00000361359->ENSP00000316840->ENSP00000358997->ENSP00000263341->ENSP00000356438 7

890.ENSP00000247668->ENSP00000359424->ENSP00000358622 2

891.ENSP00000247668->ENSP00000359424 1

892.ENSP00000247668->ENSP00000259808->ENSP00000344818->ENSP00000269305->ENSP00000361021 5

893.ENSP00000247668->ENSP00000259808->ENSP00000344818->ENSP00000269305->ENSP00000278916->ENSP00000321656->ENSP00000300093->ENSP00000263753->ENSP00000418447->ENSP00000348784->ENSP00000379866->ENSP00000379823->ENSP00000331902->ENSP00000361290 48

894.ENSP00000247668->ENSP00000361359->ENSP00000362994 2

895.ENSP00000247668->ENSP00000359424->ENSP00000384273->ENSP00000263253->ENSP00000206249->ENSP00000327213->ENSP00000363812 31

896.ENSP00000247668->ENSP00000259808->ENSP00000344818->ENSP00000275493->ENSP00000300574->ENSP00000228307->ENSP00000380227->ENSP00000364094 9

897.ENSP00000247668->ENSP00000259808->ENSP00000344818->ENSP00000275493->ENSP00000300574->ENSP00000228307->ENSP00000380227->ENSP00000364094->ENSP00000282588->ENSP00000364979 21

898.ENSP00000247668->ENSP00000259808->ENSP00000344818->ENSP00000269305->ENSP00000361021->ENSP00000366563 17

899.ENSP00000247668->ENSP00000359424->ENSP00000384273->ENSP00000263253->ENSP00000367207 4

900.ENSP00000247668->ENSP00000359424->ENSP00000384273->ENSP00000263253->ENSP00000206249->ENSP00000254227->ENSP00000342470->ENSP00000363868->ENSP00000372703 124

901.ENSP00000247668->ENSP00000259808->ENSP00000344818->ENSP00000269305->ENSP00000278916->ENSP00000321656->ENSP00000256442->ENSP00000342307->ENSP00000230538->ENSP00000222399->ENSP00000374309 27

902.ENSP00000247668->ENSP00000259808->ENSP00000344818->ENSP00000417281->ENSP00000270202->ENSP00000348986->ENSP00000375892 11

903.ENSP00000247668->ENSP00000359424->ENSP00000384273->ENSP00000263253->ENSP00000264657->ENSP00000376765 7

904.ENSP00000247668->ENSP00000259808->ENSP00000344818->ENSP00000269305->ENSP00000278916->ENSP00000321656->ENSP00000300093->ENSP00000263753->ENSP00000418447->ENSP00000348784->ENSP00000379866 45

905.ENSP00000247668->ENSP00000259808->ENSP00000344818->ENSP00000269305->ENSP00000324897->ENSP00000381648 6

906.ENSP00000247668->ENSP00000359424->ENSP00000384273 2

907.ENSP00000247668->ENSP00000259808->ENSP00000344818->ENSP00000275493->ENSP00000300574->ENSP00000228307->ENSP00000380227->ENSP00000364094->ENSP00000386896 10

908.ENSP00000247668->ENSP00000359424->ENSP00000384273->ENSP00000263253->ENSP00000206249->ENSP00000254227->ENSP00000342470->ENSP00000363868->ENSP00000393286 124

909.ENSP00000247668->ENSP00000359424->ENSP00000384273->ENSP00000263253->ENSP00000206249->ENSP00000254227->ENSP00000342470->ENSP00000363868->ENSP00000393870 124

910.ENSP00000247668->ENSP00000359424->ENSP00000384273->ENSP00000263253->ENSP00000262160->ENSP00000287727->ENSP00000400104 105

911.ENSP00000247668->ENSP00000259808->ENSP00000344818->ENSP00000275493->ENSP00000339007->ENSP00000312435->ENSP00000400365 19

912.ENSP00000247668->ENSP00000359424->ENSP00000384273->ENSP00000263253->ENSP00000206249->ENSP00000254227->ENSP00000342470->ENSP00000363868->ENSP00000402590 124

913.ENSP00000247668->ENSP00000359424->ENSP00000384273->ENSP00000263253->ENSP00000325690->ENSP00000399968->ENSP00000419692 6

914.ENSP00000249636->ENSP00000376076->ENSP00000269305->ENSP00000278916->ENSP00000321656->ENSP00000256442->ENSP00000342307->ENSP00000230538->ENSP00000354360->ENSP00000252999 23

915.ENSP00000249636->ENSP00000354394->ENSP00000263253->ENSP00000367207->ENSP00000257904 5

916.ENSP00000249636->ENSP00000354394->ENSP00000371067->ENSP00000339007->ENSP00000312435->ENSP00000400365->ENSP00000258341 22

917.ENSP00000249636->ENSP00000354394->ENSP00000371067->ENSP00000339007->ENSP00000341189->ENSP00000228307->ENSP00000380227->ENSP00000364094->ENSP00000261023 8

918.ENSP00000249636->ENSP00000354394->ENSP00000263253->ENSP00000384273->ENSP00000359424->ENSP00000247668->ENSP00000263932->ENSP00000261464 8

919.ENSP00000249636->ENSP00000354394->ENSP00000371067->ENSP00000401303->ENSP00000172229->ENSP00000262395 28

920.ENSP00000249636->ENSP00000354394->ENSP00000371067->ENSP00000339007->ENSP00000341189->ENSP00000262017->ENSP00000262407 10

921.ENSP00000249636->ENSP00000354394->ENSP00000263253->ENSP00000367207->ENSP00000281708->ENSP00000262643 5

922.ENSP00000249636->ENSP00000354394->ENSP00000343204->ENSP00000304895->ENSP00000262741 7

923.ENSP00000249636->ENSP00000376076->ENSP00000269305->ENSP00000267163->ENSP00000262904 5

924.ENSP00000249636->ENSP00000354394->ENSP00000263253->ENSP00000262160->ENSP00000341551->ENSP00000262971 8

925.ENSP00000249636->ENSP00000354394->ENSP00000263253->ENSP00000384273->ENSP00000359424->ENSP00000247668->ENSP00000263464 6

926.ENSP00000249636->ENSP00000354394->ENSP00000263253->ENSP00000338018->ENSP00000361125->ENSP00000263826 24

927.ENSP00000249636->ENSP00000354394->ENSP00000371067->ENSP00000339007->ENSP00000274335->ENSP00000263967 5

928.ENSP00000249636->ENSP00000354394->ENSP00000371067->ENSP00000339007->ENSP00000341189->ENSP00000228307->ENSP00000380227->ENSP00000364094->ENSP00000386896->ENSP00000200181->ENSP00000340937->ENSP00000264144 23

929.ENSP00000249636->ENSP00000376076->ENSP00000269305->ENSP00000244741->ENSP00000265734 5

930.ENSP00000249636->ENSP00000376076->ENSP00000269305->ENSP00000266970 4

931.ENSP00000249636->ENSP00000376076->ENSP00000269305->ENSP00000267163 4

932.ENSP00000249636->ENSP00000376076->ENSP00000417281->ENSP00000270202->ENSP00000352121->ENSP00000269300 13

933.ENSP00000249636->ENSP00000376076->ENSP00000269305 3

934.ENSP00000249636->ENSP00000376076->ENSP00000417281->ENSP00000270202 4

935.ENSP00000249636->ENSP00000354394->ENSP00000263253->ENSP00000367207->ENSP00000274255 5

936.ENSP00000249636->ENSP00000354394->ENSP00000371067->ENSP00000339007->ENSP00000274335 4

937.ENSP00000249636->ENSP00000354394->ENSP00000263253->ENSP00000367207->ENSP00000257904->ENSP00000276925 6

938.ENSP00000249636->ENSP00000354394->ENSP00000371067->ENSP00000339007->ENSP00000274335->ENSP00000289153 8

939.ENSP00000249636->ENSP00000354394->ENSP00000371067->ENSP00000339007->ENSP00000341189->ENSP00000228307->ENSP00000380227->ENSP00000364094->ENSP00000296585 8

940.ENSP00000249636->ENSP00000376076->ENSP00000269305->ENSP00000302564 5

941.ENSP00000249636->ENSP00000354394->ENSP00000371067->ENSP00000264657->ENSP00000348461->ENSP00000356505->ENSP00000220764->ENSP00000348380->ENSP00000305638 1022

942.ENSP00000249636->ENSP00000376076->ENSP00000269305->ENSP00000278916->ENSP00000321656->ENSP00000256442->ENSP00000342307->ENSP00000230538->ENSP00000354360->ENSP00000252999->ENSP00000307156 28

943.ENSP00000249636->ENSP00000376076->ENSP00000269305->ENSP00000353059->ENSP00000307786 11

944.ENSP00000249636->ENSP00000376076->ENSP00000269305->ENSP00000266970->ENSP00000309181 5

945.ENSP00000249636->ENSP00000376076->ENSP00000269305->ENSP00000266970->ENSP00000311083 5

946.ENSP00000249636->ENSP00000354394->ENSP00000371067->ENSP00000339007->ENSP00000341189->ENSP00000228307->ENSP00000380227->ENSP00000364094->ENSP00000386896->ENSP00000200181->ENSP00000340937->ENSP00000348384->ENSP00000324532 18

947.ENSP00000249636->ENSP00000354394->ENSP00000263253->ENSP00000384273->ENSP00000226574->ENSP00000327251 23

948.ENSP00000249636->ENSP00000376076->ENSP00000269305->ENSP00000329623 4

949.ENSP00000249636->ENSP00000376076->ENSP00000269305->ENSP00000353059->ENSP00000330237 6

950.ENSP00000249636->ENSP00000354394->ENSP00000263253->ENSP00000206249->ENSP00000399968->ENSP00000419692->ENSP00000332296 21

951.ENSP00000249636->ENSP00000354394->ENSP00000263253->ENSP00000384273->ENSP00000359424->ENSP00000247668->ENSP00000361359->ENSP00000332468 7

952.ENSP00000249636->ENSP00000354394->ENSP00000263253->ENSP00000384273->ENSP00000339151 4

953.ENSP00000249636->ENSP00000354394->ENSP00000371067->ENSP00000339007->ENSP00000341189 4

954.ENSP00000249636->ENSP00000376076->ENSP00000269305->ENSP00000293195->ENSP00000342087 37

955.ENSP00000249636->ENSP00000354394->ENSP00000263253->ENSP00000329357->ENSP00000345571 4

956.ENSP00000249636->ENSP00000354394->ENSP00000263253->ENSP00000262160->ENSP00000221930->ENSP00000346839 7

957.ENSP00000249636->ENSP00000376076->ENSP00000269305->ENSP00000329623->ENSP00000311032->ENSP00000347858 7

958.ENSP00000249636->ENSP00000354394->ENSP00000371067->ENSP00000339007->ENSP00000341189->ENSP00000228307->ENSP00000380227->ENSP00000364094->ENSP00000386896->ENSP00000200181->ENSP00000340937->ENSP00000348384 17

959.ENSP00000249636->ENSP00000354394->ENSP00000263253->ENSP00000367207->ENSP00000351490 4

960.ENSP00000249636->ENSP00000376076->ENSP00000417281->ENSP00000270202->ENSP00000352121 10

961.ENSP00000249636->ENSP00000354394->ENSP00000263253->ENSP00000206249->ENSP00000327213->ENSP00000352900 18

962.ENSP00000249636->ENSP00000376076->ENSP00000269305->ENSP00000353059 5

963.ENSP00000249636->ENSP00000354394->ENSP00000371067->ENSP00000339007->ENSP00000341189->ENSP00000228307->ENSP00000380227->ENSP00000364094->ENSP00000282588->ENSP00000364979->ENSP00000353654 20

964.ENSP00000249636->ENSP00000376076->ENSP00000269305->ENSP00000278916->ENSP00000321656->ENSP00000256442->ENSP00000342307->ENSP00000230538->ENSP00000354360 18

965.ENSP00000249636->ENSP00000376076->ENSP00000269305->ENSP00000267163->ENSP00000355249 5

966.ENSP00000249636->ENSP00000354394->ENSP00000338799->ENSP00000258743->ENSP00000356438 7

967.ENSP00000249636->ENSP00000354394->ENSP00000263253->ENSP00000384273->ENSP00000359424->ENSP00000358622 5

968.ENSP00000249636->ENSP00000354394->ENSP00000263253->ENSP00000384273->ENSP00000359424 4

969.ENSP00000249636->ENSP00000376076->ENSP00000269305->ENSP00000361021 4

970.ENSP00000249636->ENSP00000376076->ENSP00000269305->ENSP00000278916->ENSP00000321656->ENSP00000300093->ENSP00000263753->ENSP00000418447->ENSP00000348784->ENSP00000379866->ENSP00000379823->ENSP00000331902->ENSP00000361290 47

971.ENSP00000249636->ENSP00000354394->ENSP00000263253->ENSP00000384273->ENSP00000226574->ENSP00000362994 7

972.ENSP00000249636->ENSP00000354394->ENSP00000263253->ENSP00000206249->ENSP00000327213->ENSP00000363812 30

973.ENSP00000249636->ENSP00000354394->ENSP00000371067->ENSP00000339007->ENSP00000341189->ENSP00000228307->ENSP00000380227->ENSP00000364094 7

974.ENSP00000249636->ENSP00000354394->ENSP00000371067->ENSP00000339007->ENSP00000341189->ENSP00000228307->ENSP00000380227->ENSP00000364094->ENSP00000282588->ENSP00000364979 19

975.ENSP00000249636->ENSP00000376076->ENSP00000269305->ENSP00000361021->ENSP00000366563 16

976.ENSP00000249636->ENSP00000354394->ENSP00000263253->ENSP00000367207 3

977.ENSP00000249636->ENSP00000354394->ENSP00000263253->ENSP00000206249->ENSP00000254227->ENSP00000342470->ENSP00000363868->ENSP00000372703 123

978.ENSP00000249636->ENSP00000376076->ENSP00000269305->ENSP00000278916->ENSP00000321656->ENSP00000256442->ENSP00000342307->ENSP00000230538->ENSP00000222399->ENSP00000374309 26

979.ENSP00000249636->ENSP00000354394->ENSP00000371067->ENSP00000360683->ENSP00000348986->ENSP00000375892 10

980.ENSP00000249636->ENSP00000354394->ENSP00000371067->ENSP00000264657->ENSP00000376765 6

981.ENSP00000249636->ENSP00000376076->ENSP00000269305->ENSP00000278916->ENSP00000321656->ENSP00000300093->ENSP00000263753->ENSP00000418447->ENSP00000348784->ENSP00000379866 44

982.ENSP00000249636->ENSP00000376076->ENSP00000381648 2

983.ENSP00000249636->ENSP00000354394->ENSP00000263253->ENSP00000384273 3

984.ENSP00000249636->ENSP00000354394->ENSP00000371067->ENSP00000339007->ENSP00000341189->ENSP00000228307->ENSP00000380227->ENSP00000364094->ENSP00000386896 8

985.ENSP00000249636->ENSP00000354394->ENSP00000263253->ENSP00000206249->ENSP00000254227->ENSP00000342470->ENSP00000363868->ENSP00000393286 123

986.ENSP00000249636->ENSP00000354394->ENSP00000263253->ENSP00000206249->ENSP00000254227->ENSP00000342470->ENSP00000363868->ENSP00000393870 123

987.ENSP00000249636->ENSP00000354394->ENSP00000263253->ENSP00000262160->ENSP00000287727->ENSP00000400104 104

988.ENSP00000249636->ENSP00000354394->ENSP00000371067->ENSP00000339007->ENSP00000312435->ENSP00000400365 17

989.ENSP00000249636->ENSP00000354394->ENSP00000263253->ENSP00000206249->ENSP00000254227->ENSP00000342470->ENSP00000363868->ENSP00000402590 123

990.ENSP00000249636->ENSP00000354394->ENSP00000263253->ENSP00000206249->ENSP00000399968->ENSP00000419692 5

991.ENSP00000252999->ENSP00000354360->ENSP00000230538->ENSP00000342307->ENSP00000306043->ENSP00000255465->ENSP00000244741->ENSP00000257904 21

992.ENSP00000252999->ENSP00000258341 2

993.ENSP00000252999->ENSP00000264144->ENSP00000340937->ENSP00000200181->ENSP00000386896->ENSP00000364094->ENSP00000261023 19

994.ENSP00000252999->ENSP00000354360->ENSP00000230538->ENSP00000342307->ENSP00000256442->ENSP00000321656->ENSP00000361275->ENSP00000269305->ENSP00000344818->ENSP00000316840->ENSP00000269485->ENSP00000261464 26

995.ENSP00000252999->ENSP00000354360->ENSP00000400365->ENSP00000312435->ENSP00000339007->ENSP00000401303->ENSP00000172229->ENSP00000262395 46

996.ENSP00000252999->ENSP00000264144->ENSP00000340937->ENSP00000200181->ENSP00000386896->ENSP00000364094->ENSP00000261023->ENSP00000262017->ENSP00000262407 21

997.ENSP00000252999->ENSP00000354360->ENSP00000230538->ENSP00000342307->ENSP00000306043->ENSP00000255465->ENSP00000266970->ENSP00000262643 21

998.ENSP00000252999->ENSP00000354360->ENSP00000400365->ENSP00000312435->ENSP00000339007->ENSP00000304895->ENSP00000262741 24

999.ENSP00000252999->ENSP00000354360->ENSP00000230538->ENSP00000342307->ENSP00000306043->ENSP00000274026->ENSP00000345571->ENSP00000267163->ENSP00000262904 22

1000.ENSP00000252999->ENSP00000354360->ENSP00000230538->ENSP00000342307->ENSP00000306043->ENSP00000274026->ENSP00000345571->ENSP00000329357->ENSP00000332973->ENSP00000341551->ENSP00000262971 27

1001.ENSP00000252999->ENSP00000354360->ENSP00000230538->ENSP00000342307->ENSP00000256442->ENSP00000321656->ENSP00000361275->ENSP00000269305->ENSP00000344818->ENSP00000316840->ENSP00000361359->ENSP00000263464 25

1002.ENSP00000252999->ENSP00000354360->ENSP00000230538->ENSP00000342307->ENSP00000256442->ENSP00000321656->ENSP00000361275->ENSP00000269305->ENSP00000338018->ENSP00000361125->ENSP00000263826 42

1003.ENSP00000252999->ENSP00000354360->ENSP00000400365->ENSP00000312435->ENSP00000339007->ENSP00000274335->ENSP00000263967 22

1004.ENSP00000252999->ENSP00000264144 2

1005.ENSP00000252999->ENSP00000354360->ENSP00000230538->ENSP00000342307->ENSP00000306043->ENSP00000255465->ENSP00000244741->ENSP00000265734 21

1006.ENSP00000252999->ENSP00000354360->ENSP00000230538->ENSP00000342307->ENSP00000306043->ENSP00000255465->ENSP00000266970 20

1007.ENSP00000252999->ENSP00000354360->ENSP00000230538->ENSP00000342307->ENSP00000306043->ENSP00000274026->ENSP00000345571->ENSP00000267163 21

1008.ENSP00000252999->ENSP00000354360->ENSP00000400365->ENSP00000312435->ENSP00000341940->ENSP00000297494->ENSP00000270202->ENSP00000352121->ENSP00000269300 30

1009.ENSP00000252999->ENSP00000354360->ENSP00000230538->ENSP00000342307->ENSP00000256442->ENSP00000321656->ENSP00000361275->ENSP00000269305 20

1010.ENSP00000252999->ENSP00000354360->ENSP00000400365->ENSP00000312435->ENSP00000341940->ENSP00000297494->ENSP00000270202 21

1011.ENSP00000252999->ENSP00000354360->ENSP00000230538->ENSP00000342307->ENSP00000306043->ENSP00000255465->ENSP00000228872->ENSP00000274255 21

1012.ENSP00000252999->ENSP00000354360->ENSP00000400365->ENSP00000312435->ENSP00000339007->ENSP00000274335 21

1013.ENSP00000252999->ENSP00000354360->ENSP00000230538->ENSP00000342307->ENSP00000306043->ENSP00000255465->ENSP00000244741->ENSP00000257904->ENSP00000276925 22

1014.ENSP00000252999->ENSP00000354360->ENSP00000400365->ENSP00000312435->ENSP00000339007->ENSP00000274335->ENSP00000289153 25

1015.ENSP00000252999->ENSP00000264144->ENSP00000340937->ENSP00000200181->ENSP00000386896->ENSP00000364094->ENSP00000296585 19

1016.ENSP00000252999->ENSP00000354360->ENSP00000230538->ENSP00000342307->ENSP00000256442->ENSP00000321656->ENSP00000361275->ENSP00000269305->ENSP00000302564 22

1017.ENSP00000252999->ENSP00000354360->ENSP00000400365->ENSP00000312435->ENSP00000339007->ENSP00000302269->ENSP00000348461->ENSP00000356505->ENSP00000220764->ENSP00000348380->ENSP00000305638 1039

1018.ENSP00000252999->ENSP00000307156 5

1019.ENSP00000252999->ENSP00000354360->ENSP00000230538->ENSP00000342307->ENSP00000256442->ENSP00000321656->ENSP00000361275->ENSP00000269305->ENSP00000353059->ENSP00000307786 28

1020.ENSP00000252999->ENSP00000354360->ENSP00000230538->ENSP00000342307->ENSP00000306043->ENSP00000255465->ENSP00000266970->ENSP00000309181 21

1021.ENSP00000252999->ENSP00000354360->ENSP00000230538->ENSP00000342307->ENSP00000306043->ENSP00000255465->ENSP00000266970->ENSP00000311083 21

1022.ENSP00000252999->ENSP00000264144->ENSP00000324532 17

1023.ENSP00000252999->ENSP00000354360->ENSP00000230538->ENSP00000342307->ENSP00000256442->ENSP00000321656->ENSP00000361275->ENSP00000269305->ENSP00000263253->ENSP00000384273->ENSP00000226574->ENSP00000327251 42

1024.ENSP00000252999->ENSP00000354360->ENSP00000230538->ENSP00000342307->ENSP00000256442->ENSP00000321656->ENSP00000361275->ENSP00000269305->ENSP00000329623 21

1025.ENSP00000252999->ENSP00000354360->ENSP00000230538->ENSP00000342307->ENSP00000256442->ENSP00000321656->ENSP00000361275->ENSP00000269305->ENSP00000353059->ENSP00000330237 23

1026.ENSP00000252999->ENSP00000354360->ENSP00000230538->ENSP00000342307->ENSP00000256442->ENSP00000321656->ENSP00000372023->ENSP00000350283->ENSP00000206249->ENSP00000399968->ENSP00000419692->ENSP00000332296 39

1027.ENSP00000252999->ENSP00000354360->ENSP00000230538->ENSP00000342307->ENSP00000256442->ENSP00000321656->ENSP00000361275->ENSP00000269305->ENSP00000344818->ENSP00000316840->ENSP00000361359->ENSP00000332468 24

1028.ENSP00000252999->ENSP00000354360->ENSP00000230538->ENSP00000342307->ENSP00000256442->ENSP00000321656->ENSP00000361275->ENSP00000269305->ENSP00000263253->ENSP00000384273->ENSP00000339151 23

1029.ENSP00000252999->ENSP00000354360->ENSP00000400365->ENSP00000312435->ENSP00000339007->ENSP00000341189 21

1030.ENSP00000252999->ENSP00000354360->ENSP00000230538->ENSP00000342307->ENSP00000256442->ENSP00000321656->ENSP00000361275->ENSP00000269305->ENSP00000293195->ENSP00000342087 54

1031.ENSP00000252999->ENSP00000354360->ENSP00000230538->ENSP00000342307->ENSP00000306043->ENSP00000274026->ENSP00000345571 20

1032.ENSP00000252999->ENSP00000264144->ENSP00000340937->ENSP00000200181->ENSP00000386896->ENSP00000364094->ENSP00000346839 19

1033.ENSP00000252999->ENSP00000354360->ENSP00000230538->ENSP00000342307->ENSP00000256442->ENSP00000321656->ENSP00000361275->ENSP00000269305->ENSP00000329623->ENSP00000311032->ENSP00000347858 24

1034.ENSP00000252999->ENSP00000264144->ENSP00000324532->ENSP00000348384 18

1035.ENSP00000252999->ENSP00000354360->ENSP00000230538->ENSP00000342307->ENSP00000306043->ENSP00000274026->ENSP00000345571->ENSP00000367207->ENSP00000351490 23

1036.ENSP00000252999->ENSP00000354360->ENSP00000400365->ENSP00000312435->ENSP00000341940->ENSP00000297494->ENSP00000270202->ENSP00000352121 27

1037.ENSP00000252999->ENSP00000354360->ENSP00000230538->ENSP00000342307->ENSP00000256442->ENSP00000321656->ENSP00000372023->ENSP00000350283->ENSP00000206249->ENSP00000327213->ENSP00000352900 36

1038.ENSP00000252999->ENSP00000354360->ENSP00000230538->ENSP00000342307->ENSP00000256442->ENSP00000321656->ENSP00000361275->ENSP00000269305->ENSP00000353059 22

1039.ENSP00000252999->ENSP00000264144->ENSP00000340937->ENSP00000200181->ENSP00000386896->ENSP00000364094->ENSP00000282588->ENSP00000364979->ENSP00000353654 31

1040.ENSP00000252999->ENSP00000354360 5

1041.ENSP00000252999->ENSP00000354360->ENSP00000230538->ENSP00000342307->ENSP00000306043->ENSP00000274026->ENSP00000345571->ENSP00000267163->ENSP00000355249 22

1042.ENSP00000252999->ENSP00000354360->ENSP00000230538->ENSP00000342307->ENSP00000256442->ENSP00000321656->ENSP00000361275->ENSP00000269305->ENSP00000344352->ENSP00000360266->ENSP00000356438 27

1043.ENSP00000252999->ENSP00000354360->ENSP00000230538->ENSP00000342307->ENSP00000256442->ENSP00000321656->ENSP00000361275->ENSP00000269305->ENSP00000344818->ENSP00000316840->ENSP00000358622 23

1044.ENSP00000252999->ENSP00000354360->ENSP00000230538->ENSP00000342307->ENSP00000256442->ENSP00000321656->ENSP00000361275->ENSP00000269305->ENSP00000263253->ENSP00000384273->ENSP00000359424 23

1045.ENSP00000252999->ENSP00000354360->ENSP00000230538->ENSP00000342307->ENSP00000256442->ENSP00000321656->ENSP00000361275->ENSP00000269305->ENSP00000361021 21

1046.ENSP00000252999->ENSP00000354360->ENSP00000230538->ENSP00000342307->ENSP00000256442->ENSP00000300093->ENSP00000263753->ENSP00000418447->ENSP00000348784->ENSP00000379866->ENSP00000379823->ENSP00000331902->ENSP00000361290 59

1047.ENSP00000252999->ENSP00000354360->ENSP00000230538->ENSP00000342307->ENSP00000256442->ENSP00000321656->ENSP00000361275->ENSP00000269305->ENSP00000344818->ENSP00000316840->ENSP00000361359->ENSP00000362994 24

1048.ENSP00000252999->ENSP00000354360->ENSP00000230538->ENSP00000342307->ENSP00000256442->ENSP00000321656->ENSP00000372023->ENSP00000350283->ENSP00000206249->ENSP00000327213->ENSP00000363812 48

1049.ENSP00000252999->ENSP00000264144->ENSP00000340937->ENSP00000200181->ENSP00000386896->ENSP00000364094 18

1050.ENSP00000252999->ENSP00000264144->ENSP00000340937->ENSP00000200181->ENSP00000386896->ENSP00000364094->ENSP00000282588->ENSP00000364979 30

1051.ENSP00000252999->ENSP00000354360->ENSP00000230538->ENSP00000342307->ENSP00000256442->ENSP00000321656->ENSP00000361275->ENSP00000269305->ENSP00000361021->ENSP00000366563 33

1052.ENSP00000252999->ENSP00000354360->ENSP00000230538->ENSP00000342307->ENSP00000306043->ENSP00000274026->ENSP00000345571->ENSP00000367207 22

1053.ENSP00000252999->ENSP00000354360->ENSP00000400365->ENSP00000312435->ENSP00000363827->ENSP00000252486->ENSP00000252444->ENSP00000233242->ENSP00000236850->ENSP00000363868->ENSP00000372703 140

1054.ENSP00000252999->ENSP00000258341->ENSP00000374309 7

1055.ENSP00000252999->ENSP00000354360->ENSP00000400365->ENSP00000312435->ENSP00000339007->ENSP00000304895->ENSP00000348986->ENSP00000375892 27

1056.ENSP00000252999->ENSP00000354360->ENSP00000400365->ENSP00000312435->ENSP00000339007->ENSP00000275493->ENSP00000264657->ENSP00000376765 25

1057.ENSP00000252999->ENSP00000354360->ENSP00000230538->ENSP00000342307->ENSP00000256442->ENSP00000300093->ENSP00000263753->ENSP00000418447->ENSP00000348784->ENSP00000379866 56

1058.ENSP00000252999->ENSP00000354360->ENSP00000230538->ENSP00000342307->ENSP00000256442->ENSP00000321656->ENSP00000361275->ENSP00000269305->ENSP00000324897->ENSP00000381648 22

1059.ENSP00000252999->ENSP00000354360->ENSP00000230538->ENSP00000342307->ENSP00000256442->ENSP00000321656->ENSP00000361275->ENSP00000269305->ENSP00000263253->ENSP00000384273 22

1060.ENSP00000252999->ENSP00000264144->ENSP00000340937->ENSP00000200181->ENSP00000386896 17

1061.ENSP00000252999->ENSP00000354360->ENSP00000400365->ENSP00000312435->ENSP00000363827->ENSP00000252486->ENSP00000252444->ENSP00000233242->ENSP00000236850->ENSP00000363868->ENSP00000393286 140

1062.ENSP00000252999->ENSP00000354360->ENSP00000400365->ENSP00000312435->ENSP00000363827->ENSP00000252486->ENSP00000252444->ENSP00000233242->ENSP00000236850->ENSP00000363868->ENSP00000393870 140

1063.ENSP00000252999->ENSP00000354360->ENSP00000230538->ENSP00000342307->ENSP00000306043->ENSP00000274026->ENSP00000345571->ENSP00000329357->ENSP00000332973->ENSP00000287727->ENSP00000400104 123

1064.ENSP00000252999->ENSP00000354360->ENSP00000400365 6

1065.ENSP00000252999->ENSP00000354360->ENSP00000400365->ENSP00000312435->ENSP00000363827->ENSP00000252486->ENSP00000252444->ENSP00000233242->ENSP00000236850->ENSP00000363868->ENSP00000402590 140

1066.ENSP00000252999->ENSP00000354360->ENSP00000230538->ENSP00000342307->ENSP00000256442->ENSP00000321656->ENSP00000372023->ENSP00000350283->ENSP00000206249->ENSP00000399968->ENSP00000419692 23

1067.ENSP00000257904->ENSP00000244741->ENSP00000274026->ENSP00000306043->ENSP00000342307->ENSP00000230538->ENSP00000354360->ENSP00000400365->ENSP00000258341 22

1068.ENSP00000257904->ENSP00000228872->ENSP00000270202->ENSP00000299421->ENSP00000228307->ENSP00000380227->ENSP00000346839->ENSP00000261023 8

1069.ENSP00000257904->ENSP00000244741->ENSP00000344818->ENSP00000316840->ENSP00000269485->ENSP00000261464 8

1070.ENSP00000257904->ENSP00000227507->ENSP00000345571->ENSP00000332643->ENSP00000172229->ENSP00000262395 29

1071.ENSP00000257904->ENSP00000228872->ENSP00000270202->ENSP00000299421->ENSP00000228307->ENSP00000380227->ENSP00000346839->ENSP00000261023->ENSP00000262017->ENSP00000262407 10

1072.ENSP00000257904->ENSP00000244741->ENSP00000262643 2

1073.ENSP00000257904->ENSP00000355153->ENSP00000269305->ENSP00000353483->ENSP00000304895->ENSP00000262741 7

1074.ENSP00000257904->ENSP00000267163->ENSP00000262904 2

1075.ENSP00000257904->ENSP00000367207->ENSP00000263253->ENSP00000262160->ENSP00000341551->ENSP00000262971 9

1076.ENSP00000257904->ENSP00000267163->ENSP00000362649->ENSP00000384273->ENSP00000359424->ENSP00000247668->ENSP00000263464 6

1077.ENSP00000257904->ENSP00000355153->ENSP00000269305->ENSP00000338018->ENSP00000361125->ENSP00000263826 24

1078.ENSP00000257904->ENSP00000228872->ENSP00000270202->ENSP00000263967 4

1079.ENSP00000257904->ENSP00000228872->ENSP00000270202->ENSP00000299421->ENSP00000228307->ENSP00000380227->ENSP00000364094->ENSP00000386896->ENSP00000200181->ENSP00000340937->ENSP00000264144 23

1080.ENSP00000257904->ENSP00000362082->ENSP00000265734 2

1081.ENSP00000257904->ENSP00000227507->ENSP00000266970 2

1082.ENSP00000257904->ENSP00000267163 1

1083.ENSP00000257904->ENSP00000228872->ENSP00000270202->ENSP00000352121->ENSP00000269300 11

1084.ENSP00000257904->ENSP00000355153->ENSP00000269305 2

1085.ENSP00000257904->ENSP00000228872->ENSP00000270202 2

1086.ENSP00000257904->ENSP00000228872->ENSP00000274255 2

1087.ENSP00000257904->ENSP00000227507->ENSP00000344456->ENSP00000274335 5

1088.ENSP00000257904->ENSP00000276925 1

1089.ENSP00000257904->ENSP00000228872->ENSP00000270202->ENSP00000289153 6

1090.ENSP00000257904->ENSP00000228872->ENSP00000270202->ENSP00000299421->ENSP00000228307->ENSP00000380227->ENSP00000364094->ENSP00000296585 8

1091.ENSP00000257904->ENSP00000355153->ENSP00000269305->ENSP00000302564 4

1092.ENSP00000257904->ENSP00000228872->ENSP00000270202->ENSP00000348461->ENSP00000356505->ENSP00000220764->ENSP00000348380->ENSP00000305638 1020

1093.ENSP00000257904->ENSP00000244741->ENSP00000274026->ENSP00000306043->ENSP00000342307->ENSP00000230538->ENSP00000354360->ENSP00000252999->ENSP00000307156 26

1094.ENSP00000257904->ENSP00000355153->ENSP00000269305->ENSP00000353059->ENSP00000307786 10

1095.ENSP00000257904->ENSP00000227507->ENSP00000266970->ENSP00000309181 3

1096.ENSP00000257904->ENSP00000227507->ENSP00000266970->ENSP00000311083 3

1097.ENSP00000257904->ENSP00000228872->ENSP00000270202->ENSP00000299421->ENSP00000228307->ENSP00000380227->ENSP00000364094->ENSP00000386896->ENSP00000200181->ENSP00000340937->ENSP00000348384->ENSP00000324532 18

1098.ENSP00000257904->ENSP00000267163->ENSP00000362649->ENSP00000384273->ENSP00000226574->ENSP00000327251 23

1099.ENSP00000257904->ENSP00000355153->ENSP00000269305->ENSP00000329623 3

1100.ENSP00000257904->ENSP00000355153->ENSP00000269305->ENSP00000353059->ENSP00000330237 5

1101.ENSP00000257904->ENSP00000227507->ENSP00000206249->ENSP00000399968->ENSP00000419692->ENSP00000332296 21

1102.ENSP00000257904->ENSP00000244741->ENSP00000344818->ENSP00000316840->ENSP00000361359->ENSP00000332468 6

1103.ENSP00000257904->ENSP00000267163->ENSP00000362649->ENSP00000384273->ENSP00000339151 4

1104.ENSP00000257904->ENSP00000355153->ENSP00000269305->ENSP00000361021->ENSP00000341189 5

1105.ENSP00000257904->ENSP00000355153->ENSP00000269305->ENSP00000293195->ENSP00000342087 36

1106.ENSP00000257904->ENSP00000227507->ENSP00000345571 2

1107.ENSP00000257904->ENSP00000228872->ENSP00000270202->ENSP00000299421->ENSP00000228307->ENSP00000380227->ENSP00000346839 7

1108.ENSP00000257904->ENSP00000355153->ENSP00000269305->ENSP00000355759->ENSP00000311032->ENSP00000347858 6

1109.ENSP00000257904->ENSP00000228872->ENSP00000270202->ENSP00000299421->ENSP00000228307->ENSP00000380227->ENSP00000364094->ENSP00000386896->ENSP00000200181->ENSP00000340937->ENSP00000348384 17

1110.ENSP00000257904->ENSP00000367207->ENSP00000351490 3

1111.ENSP00000257904->ENSP00000228872->ENSP00000270202->ENSP00000352121 8

1112.ENSP00000257904->ENSP00000227507->ENSP00000206249->ENSP00000327213->ENSP00000352900 18

1113.ENSP00000257904->ENSP00000355153->ENSP00000269305->ENSP00000353059 4

1114.ENSP00000257904->ENSP00000228872->ENSP00000270202->ENSP00000299421->ENSP00000228307->ENSP00000380227->ENSP00000364094->ENSP00000282588->ENSP00000364979->ENSP00000353654 20

1115.ENSP00000257904->ENSP00000244741->ENSP00000274026->ENSP00000306043->ENSP00000342307->ENSP00000230538->ENSP00000354360 16

1116.ENSP00000257904->ENSP00000267163->ENSP00000355249 2

1117.ENSP00000257904->ENSP00000227507->ENSP00000264657->ENSP00000258743->ENSP00000356438 8

1118.ENSP00000257904->ENSP00000267163->ENSP00000362649->ENSP00000384273->ENSP00000339151->ENSP00000358622 5

1119.ENSP00000257904->ENSP00000267163->ENSP00000362649->ENSP00000384273->ENSP00000359424 4

1120.ENSP00000257904->ENSP00000355153->ENSP00000269305->ENSP00000361021 3

1121.ENSP00000257904->ENSP00000355153->ENSP00000269305->ENSP00000278916->ENSP00000321656->ENSP00000300093->ENSP00000263753->ENSP00000418447->ENSP00000348784->ENSP00000379866->ENSP00000379823->ENSP00000331902->ENSP00000361290 46

1122.ENSP00000257904->ENSP00000244741->ENSP00000344818->ENSP00000316840->ENSP00000361359->ENSP00000362994 6

1123.ENSP00000257904->ENSP00000227507->ENSP00000206249->ENSP00000327213->ENSP00000363812 30

1124.ENSP00000257904->ENSP00000228872->ENSP00000270202->ENSP00000299421->ENSP00000228307->ENSP00000380227->ENSP00000364094 7

1125.ENSP00000257904->ENSP00000228872->ENSP00000270202->ENSP00000299421->ENSP00000228307->ENSP00000380227->ENSP00000364094->ENSP00000282588->ENSP00000364979 19

1126.ENSP00000257904->ENSP00000355153->ENSP00000269305->ENSP00000361021->ENSP00000366563 15

1127.ENSP00000257904->ENSP00000367207 2

1128.ENSP00000257904->ENSP00000227507->ENSP00000344456->ENSP00000326366->ENSP00000284981->ENSP00000252486->ENSP00000252444->ENSP00000233242->ENSP00000236850->ENSP00000363868->ENSP00000372703 123

1129.ENSP00000257904->ENSP00000244741->ENSP00000274026->ENSP00000306043->ENSP00000342307->ENSP00000230538->ENSP00000222399->ENSP00000374309 24

1130.ENSP00000257904->ENSP00000228872->ENSP00000270202->ENSP00000348986->ENSP00000375892 8

1131.ENSP00000257904->ENSP00000227507->ENSP00000264657->ENSP00000376765 6

1132.ENSP00000257904->ENSP00000355153->ENSP00000269305->ENSP00000278916->ENSP00000321656->ENSP00000300093->ENSP00000263753->ENSP00000418447->ENSP00000348784->ENSP00000379866 43

1133.ENSP00000257904->ENSP00000355153->ENSP00000269305->ENSP00000324897->ENSP00000381648 4

1134.ENSP00000257904->ENSP00000267163->ENSP00000362649->ENSP00000384273 3

1135.ENSP00000257904->ENSP00000228872->ENSP00000270202->ENSP00000299421->ENSP00000228307->ENSP00000380227->ENSP00000364094->ENSP00000386896 8

1136.ENSP00000257904->ENSP00000227507->ENSP00000344456->ENSP00000326366->ENSP00000284981->ENSP00000252486->ENSP00000252444->ENSP00000233242->ENSP00000236850->ENSP00000363868->ENSP00000393286 123

1137.ENSP00000257904->ENSP00000227507->ENSP00000344456->ENSP00000326366->ENSP00000284981->ENSP00000252486->ENSP00000252444->ENSP00000233242->ENSP00000236850->ENSP00000363868->ENSP00000393870 123

1138.ENSP00000257904->ENSP00000367207->ENSP00000263253->ENSP00000262160->ENSP00000287727->ENSP00000400104 105

1139.ENSP00000257904->ENSP00000244741->ENSP00000274026->ENSP00000306043->ENSP00000342307->ENSP00000230538->ENSP00000354360->ENSP00000400365 17

1140.ENSP00000257904->ENSP00000227507->ENSP00000344456->ENSP00000326366->ENSP00000284981->ENSP00000252486->ENSP00000252444->ENSP00000233242->ENSP00000236850->ENSP00000363868->ENSP00000402590 123

1141.ENSP00000257904->ENSP00000227507->ENSP00000206249->ENSP00000399968->ENSP00000419692 5

1142.ENSP00000258341->ENSP00000252999->ENSP00000264144->ENSP00000340937->ENSP00000200181->ENSP00000386896->ENSP00000364094->ENSP00000261023 21

1143.ENSP00000258341->ENSP00000400365->ENSP00000312435->ENSP00000339007->ENSP00000275493->ENSP00000344818->ENSP00000316840->ENSP00000269485->ENSP00000261464 26

1144.ENSP00000258341->ENSP00000400365->ENSP00000312435->ENSP00000339007->ENSP00000401303->ENSP00000172229->ENSP00000262395 45

1145.ENSP00000258341->ENSP00000252999->ENSP00000264144->ENSP00000340937->ENSP00000200181->ENSP00000386896->ENSP00000364094->ENSP00000261023->ENSP00000262017->ENSP00000262407 23

1146.ENSP00000258341->ENSP00000400365->ENSP00000312435->ENSP00000341940->ENSP00000297494->ENSP00000270202->ENSP00000228872->ENSP00000262643 22

1147.ENSP00000258341->ENSP00000400365->ENSP00000312435->ENSP00000339007->ENSP00000304895->ENSP00000262741 23

1148.ENSP00000258341->ENSP00000400365->ENSP00000312435->ENSP00000341940->ENSP00000297494->ENSP00000270202->ENSP00000417281->ENSP00000267163->ENSP00000262904 23

1149.ENSP00000258341->ENSP00000400365->ENSP00000312435->ENSP00000339007->ENSP00000275493->ENSP00000264657->ENSP00000263253->ENSP00000262160->ENSP00000341551->ENSP00000262971 28

1150.ENSP00000258341->ENSP00000400365->ENSP00000312435->ENSP00000339007->ENSP00000275493->ENSP00000344818->ENSP00000316840->ENSP00000361359->ENSP00000263464 25

1151.ENSP00000258341->ENSP00000400365->ENSP00000312435->ENSP00000339007->ENSP00000261799->ENSP00000244007->ENSP00000263923->ENSP00000361125->ENSP00000263826 43

1152.ENSP00000258341->ENSP00000400365->ENSP00000312435->ENSP00000339007->ENSP00000274335->ENSP00000263967 21

1153.ENSP00000258341->ENSP00000252999->ENSP00000264144 4

1154.ENSP00000258341->ENSP00000400365->ENSP00000312435->ENSP00000341940->ENSP00000297494->ENSP00000270202->ENSP00000228872->ENSP00000265734 22

1155.ENSP00000258341->ENSP00000400365->ENSP00000354360->ENSP00000230538->ENSP00000342307->ENSP00000306043->ENSP00000255465->ENSP00000266970 21

1156.ENSP00000258341->ENSP00000400365->ENSP00000312435->ENSP00000341940->ENSP00000297494->ENSP00000270202->ENSP00000417281->ENSP00000267163 22

1157.ENSP00000258341->ENSP00000400365->ENSP00000312435->ENSP00000341940->ENSP00000297494->ENSP00000270202->ENSP00000352121->ENSP00000269300 29

1158.ENSP00000258341->ENSP00000400365->ENSP00000354360->ENSP00000230538->ENSP00000342307->ENSP00000256442->ENSP00000321656->ENSP00000278916->ENSP00000269305 21

1159.ENSP00000258341->ENSP00000400365->ENSP00000312435->ENSP00000341940->ENSP00000297494->ENSP00000270202 20

1160.ENSP00000258341->ENSP00000400365->ENSP00000312435->ENSP00000341940->ENSP00000297494->ENSP00000270202->ENSP00000228872->ENSP00000274255 22

1161.ENSP00000258341->ENSP00000400365->ENSP00000312435->ENSP00000339007->ENSP00000274335 20

1162.ENSP00000258341->ENSP00000400365->ENSP00000312435->ENSP00000341940->ENSP00000297494->ENSP00000270202->ENSP00000228872->ENSP00000257904->ENSP00000276925 23

1163.ENSP00000258341->ENSP00000400365->ENSP00000312435->ENSP00000341940->ENSP00000297494->ENSP00000270202->ENSP00000289153 24

1164.ENSP00000258341->ENSP00000252999->ENSP00000264144->ENSP00000340937->ENSP00000200181->ENSP00000386896->ENSP00000364094->ENSP00000296585 21

1165.ENSP00000258341->ENSP00000400365->ENSP00000312435->ENSP00000341940->ENSP00000297494->ENSP00000270202->ENSP00000309103->ENSP00000302564 22

1166.ENSP00000258341->ENSP00000400365->ENSP00000312435->ENSP00000341940->ENSP00000297494->ENSP00000270202->ENSP00000348461->ENSP00000356505->ENSP00000220764->ENSP00000348380->ENSP00000305638 1038

1167.ENSP00000258341->ENSP00000252999->ENSP00000307156 7

1168.ENSP00000258341->ENSP00000400365->ENSP00000354360->ENSP00000230538->ENSP00000342307->ENSP00000256442->ENSP00000321656->ENSP00000278916->ENSP00000269305->ENSP00000353059->ENSP00000307786 29

1169.ENSP00000258341->ENSP00000400365->ENSP00000354360->ENSP00000230538->ENSP00000342307->ENSP00000306043->ENSP00000255465->ENSP00000266970->ENSP00000309181 22

1170.ENSP00000258341->ENSP00000400365->ENSP00000354360->ENSP00000230538->ENSP00000342307->ENSP00000306043->ENSP00000255465->ENSP00000266970->ENSP00000311083 22

1171.ENSP00000258341->ENSP00000252999->ENSP00000264144->ENSP00000324532 19

1172.ENSP00000258341->ENSP00000400365->ENSP00000312435->ENSP00000341940->ENSP00000297494->ENSP00000335153->ENSP00000231509->ENSP00000226574->ENSP00000327251 43

1173.ENSP00000258341->ENSP00000400365->ENSP00000312435->ENSP00000341940->ENSP00000297494->ENSP00000270202->ENSP00000309103->ENSP00000329623 22

1174.ENSP00000258341->ENSP00000400365->ENSP00000354360->ENSP00000230538->ENSP00000342307->ENSP00000256442->ENSP00000321656->ENSP00000278916->ENSP00000269305->ENSP00000353059->ENSP00000330237 24

1175.ENSP00000258341->ENSP00000400365->ENSP00000312435->ENSP00000341940->ENSP00000297494->ENSP00000270202->ENSP00000206249->ENSP00000399968->ENSP00000419692->ENSP00000332296 40

1176.ENSP00000258341->ENSP00000400365->ENSP00000312435->ENSP00000339007->ENSP00000275493->ENSP00000344818->ENSP00000316840->ENSP00000361359->ENSP00000332468 24

1177.ENSP00000258341->ENSP00000400365->ENSP00000312435->ENSP00000341940->ENSP00000297494->ENSP00000270202->ENSP00000298552->ENSP00000339151 24

1178.ENSP00000258341->ENSP00000400365->ENSP00000312435->ENSP00000339007->ENSP00000341189 20

1179.ENSP00000258341->ENSP00000400365->ENSP00000354360->ENSP00000230538->ENSP00000342307->ENSP00000256442->ENSP00000321656->ENSP00000278916->ENSP00000269305->ENSP00000293195->ENSP00000342087 55

1180.ENSP00000258341->ENSP00000400365->ENSP00000354360->ENSP00000230538->ENSP00000342307->ENSP00000306043->ENSP00000274026->ENSP00000345571 21

1181.ENSP00000258341->ENSP00000252999->ENSP00000264144->ENSP00000340937->ENSP00000200181->ENSP00000386896->ENSP00000364094->ENSP00000346839 21

1182.ENSP00000258341->ENSP00000400365->ENSP00000312435->ENSP00000339007->ENSP00000275493->ENSP00000344818->ENSP00000316840->ENSP00000216160->ENSP00000347858 24

1183.ENSP00000258341->ENSP00000252999->ENSP00000264144->ENSP00000324532->ENSP00000348384 20

1184.ENSP00000258341->ENSP00000400365->ENSP00000312435->ENSP00000339007->ENSP00000215832->ENSP00000367207->ENSP00000351490 23

1185.ENSP00000258341->ENSP00000400365->ENSP00000312435->ENSP00000341940->ENSP00000297494->ENSP00000270202->ENSP00000352121 26

1186.ENSP00000258341->ENSP00000400365->ENSP00000312435->ENSP00000341940->ENSP00000297494->ENSP00000270202->ENSP00000206249->ENSP00000327213->ENSP00000352900 37

1187.ENSP00000258341->ENSP00000400365->ENSP00000354360->ENSP00000230538->ENSP00000342307->ENSP00000256442->ENSP00000321656->ENSP00000278916->ENSP00000269305->ENSP00000353059 23

1188.ENSP00000258341->ENSP00000252999->ENSP00000264144->ENSP00000340937->ENSP00000200181->ENSP00000386896->ENSP00000364094->ENSP00000282588->ENSP00000364979->ENSP00000353654 33

1189.ENSP00000258341->ENSP00000400365->ENSP00000354360 6

1190.ENSP00000258341->ENSP00000400365->ENSP00000312435->ENSP00000341940->ENSP00000297494->ENSP00000270202->ENSP00000417281->ENSP00000267163->ENSP00000355249 23

1191.ENSP00000258341->ENSP00000400365->ENSP00000312435->ENSP00000339007->ENSP00000275493->ENSP00000264657->ENSP00000258743->ENSP00000356438 26

1192.ENSP00000258341->ENSP00000400365->ENSP00000312435->ENSP00000339007->ENSP00000275493->ENSP00000344818->ENSP00000316840->ENSP00000358622 23

1193.ENSP00000258341->ENSP00000400365->ENSP00000312435->ENSP00000339007->ENSP00000275493->ENSP00000264657->ENSP00000263253->ENSP00000384273->ENSP00000359424 24

1194.ENSP00000258341->ENSP00000400365->ENSP00000312435->ENSP00000339007->ENSP00000341189->ENSP00000361021 22

1195.ENSP00000258341->ENSP00000400365->ENSP00000354360->ENSP00000230538->ENSP00000342307->ENSP00000256442->ENSP00000300093->ENSP00000263753->ENSP00000418447->ENSP00000348784->ENSP00000379866->ENSP00000379823->ENSP00000331902->ENSP00000361290 60

1196.ENSP00000258341->ENSP00000400365->ENSP00000312435->ENSP00000339007->ENSP00000275493->ENSP00000344818->ENSP00000316840->ENSP00000361359->ENSP00000362994 24

1197.ENSP00000258341->ENSP00000400365->ENSP00000312435->ENSP00000341940->ENSP00000297494->ENSP00000270202->ENSP00000206249->ENSP00000327213->ENSP00000363812 49

1198.ENSP00000258341->ENSP00000252999->ENSP00000264144->ENSP00000340937->ENSP00000200181->ENSP00000386896->ENSP00000364094 20

1199.ENSP00000258341->ENSP00000252999->ENSP00000264144->ENSP00000340937->ENSP00000200181->ENSP00000386896->ENSP00000364094->ENSP00000282588->ENSP00000364979 32

1200.ENSP00000258341->ENSP00000400365->ENSP00000312435->ENSP00000339007->ENSP00000341189->ENSP00000361021->ENSP00000366563 34

1201.ENSP00000258341->ENSP00000400365->ENSP00000312435->ENSP00000339007->ENSP00000215832->ENSP00000367207 22

1202.ENSP00000258341->ENSP00000400365->ENSP00000312435->ENSP00000363827->ENSP00000252486->ENSP00000252444->ENSP00000233242->ENSP00000236850->ENSP00000363868->ENSP00000372703 139

1203.ENSP00000258341->ENSP00000374309 5

1204.ENSP00000258341->ENSP00000400365->ENSP00000312435->ENSP00000341940->ENSP00000297494->ENSP00000270202->ENSP00000348986->ENSP00000375892 26

1205.ENSP00000258341->ENSP00000400365->ENSP00000312435->ENSP00000339007->ENSP00000275493->ENSP00000264657->ENSP00000376765 24

1206.ENSP00000258341->ENSP00000400365->ENSP00000354360->ENSP00000230538->ENSP00000342307->ENSP00000256442->ENSP00000300093->ENSP00000263753->ENSP00000418447->ENSP00000348784->ENSP00000379866 57

1207.ENSP00000258341->ENSP00000400365->ENSP00000354360->ENSP00000230538->ENSP00000342307->ENSP00000256442->ENSP00000321656->ENSP00000278916->ENSP00000269305->ENSP00000324897->ENSP00000381648 23

1208.ENSP00000258341->ENSP00000400365->ENSP00000312435->ENSP00000339007->ENSP00000275493->ENSP00000264657->ENSP00000263253->ENSP00000384273 23

1209.ENSP00000258341->ENSP00000252999->ENSP00000264144->ENSP00000340937->ENSP00000200181->ENSP00000386896 19

1210.ENSP00000258341->ENSP00000400365->ENSP00000312435->ENSP00000363827->ENSP00000252486->ENSP00000252444->ENSP00000233242->ENSP00000236850->ENSP00000363868->ENSP00000393286 139

1211.ENSP00000258341->ENSP00000400365->ENSP00000312435->ENSP00000363827->ENSP00000252486->ENSP00000252444->ENSP00000233242->ENSP00000236850->ENSP00000363868->ENSP00000393870 139

1212.ENSP00000258341->ENSP00000400365->ENSP00000312435->ENSP00000339007->ENSP00000275493->ENSP00000264657->ENSP00000263253->ENSP00000262160->ENSP00000287727->ENSP00000400104 124

1213.ENSP00000258341->ENSP00000400365 5

1214.ENSP00000258341->ENSP00000400365->ENSP00000312435->ENSP00000363827->ENSP00000252486->ENSP00000252444->ENSP00000233242->ENSP00000236850->ENSP00000363868->ENSP00000402590 139

1215.ENSP00000258341->ENSP00000400365->ENSP00000312435->ENSP00000341940->ENSP00000297494->ENSP00000270202->ENSP00000206249->ENSP00000399968->ENSP00000419692 24

1216.ENSP00000261023->ENSP00000361125->ENSP00000338018->ENSP00000344818->ENSP00000316840->ENSP00000269485->ENSP00000261464 12

1217.ENSP00000261023->ENSP00000364094->ENSP00000380227->ENSP00000228307->ENSP00000341189->ENSP00000339007->ENSP00000401303->ENSP00000172229->ENSP00000262395 31

1218.ENSP00000261023->ENSP00000262017->ENSP00000262407 2

1219.ENSP00000261023->ENSP00000364094->ENSP00000380227->ENSP00000228307->ENSP00000299421->ENSP00000270202->ENSP00000228872->ENSP00000262643 8

1220.ENSP00000261023->ENSP00000364094->ENSP00000380227->ENSP00000228307->ENSP00000341189->ENSP00000339007->ENSP00000304895->ENSP00000262741 9

1221.ENSP00000261023->ENSP00000361125->ENSP00000338018->ENSP00000269305->ENSP00000267163->ENSP00000262904 9

1222.ENSP00000261023->ENSP00000346839->ENSP00000221930->ENSP00000364133->ENSP00000341551->ENSP00000262971 10

1223.ENSP00000261023->ENSP00000346839->ENSP00000221930->ENSP00000262160->ENSP00000263253->ENSP00000384273->ENSP00000359424->ENSP00000247668->ENSP00000263464 10

1224.ENSP00000261023->ENSP00000361125->ENSP00000263826 25

1225.ENSP00000261023->ENSP00000364094->ENSP00000380227->ENSP00000228307->ENSP00000341189->ENSP00000339007->ENSP00000274335->ENSP00000263967 7

1226.ENSP00000261023->ENSP00000364094->ENSP00000386896->ENSP00000200181->ENSP00000340937->ENSP00000264144 17

1227.ENSP00000261023->ENSP00000364094->ENSP00000380227->ENSP00000228307->ENSP00000299421->ENSP00000270202->ENSP00000228872->ENSP00000265734 8

1228.ENSP00000261023->ENSP00000361125->ENSP00000338018->ENSP00000269305->ENSP00000266970 8

1229.ENSP00000261023->ENSP00000361125->ENSP00000338018->ENSP00000269305->ENSP00000267163 8

1230.ENSP00000261023->ENSP00000364094->ENSP00000380227->ENSP00000228307->ENSP00000299421->ENSP00000270202->ENSP00000352121->ENSP00000269300 15

1231.ENSP00000261023->ENSP00000361125->ENSP00000338018->ENSP00000269305 7

1232.ENSP00000261023->ENSP00000364094->ENSP00000380227->ENSP00000228307->ENSP00000299421->ENSP00000270202 6

1233.ENSP00000261023->ENSP00000364094->ENSP00000380227->ENSP00000228307->ENSP00000299421->ENSP00000270202->ENSP00000228872->ENSP00000274255 8

1234.ENSP00000261023->ENSP00000364094->ENSP00000380227->ENSP00000228307->ENSP00000341189->ENSP00000339007->ENSP00000274335 6

1235.ENSP00000261023->ENSP00000364094->ENSP00000380227->ENSP00000228307->ENSP00000299421->ENSP00000270202->ENSP00000228872->ENSP00000257904->ENSP00000276925 9

1236.ENSP00000261023->ENSP00000364094->ENSP00000380227->ENSP00000228307->ENSP00000341189->ENSP00000339007->ENSP00000274335->ENSP00000289153 10

1237.ENSP00000261023->ENSP00000364094->ENSP00000296585 2

1238.ENSP00000261023->ENSP00000364094->ENSP00000380227->ENSP00000228307->ENSP00000299421->ENSP00000270202->ENSP00000309103->ENSP00000302564 8

1239.ENSP00000261023->ENSP00000364094->ENSP00000380227->ENSP00000228307->ENSP00000300574->ENSP00000381107->ENSP00000348461->ENSP00000356505->ENSP00000220764->ENSP00000348380->ENSP00000305638 1023

1240.ENSP00000261023->ENSP00000364094->ENSP00000386896->ENSP00000200181->ENSP00000340937->ENSP00000264144->ENSP00000252999->ENSP00000307156 24

1241.ENSP00000261023->ENSP00000361125->ENSP00000338018->ENSP00000269305->ENSP00000353059->ENSP00000307786 15

1242.ENSP00000261023->ENSP00000361125->ENSP00000338018->ENSP00000269305->ENSP00000266970->ENSP00000309181 9

1243.ENSP00000261023->ENSP00000361125->ENSP00000338018->ENSP00000269305->ENSP00000266970->ENSP00000311083 9

1244.ENSP00000261023->ENSP00000364094->ENSP00000386896->ENSP00000200181->ENSP00000340937->ENSP00000348384->ENSP00000324532 12

1245.ENSP00000261023->ENSP00000226218->ENSP00000223095->ENSP00000361850->ENSP00000226574->ENSP00000327251 27

1246.ENSP00000261023->ENSP00000361125->ENSP00000338018->ENSP00000269305->ENSP00000329623 8

1247.ENSP00000261023->ENSP00000361125->ENSP00000338018->ENSP00000269305->ENSP00000353059->ENSP00000330237 10

1248.ENSP00000261023->ENSP00000364094->ENSP00000380227->ENSP00000228307->ENSP00000341189->ENSP00000350941->ENSP00000206249->ENSP00000399968->ENSP00000419692->ENSP00000332296 24

1249.ENSP00000261023->ENSP00000361125->ENSP00000338018->ENSP00000344818->ENSP00000316840->ENSP00000361359->ENSP00000332468 10

1250.ENSP00000261023->ENSP00000346839->ENSP00000221930->ENSP00000262160->ENSP00000263253->ENSP00000384273->ENSP00000339151 8

1251.ENSP00000261023->ENSP00000364094->ENSP00000380227->ENSP00000228307->ENSP00000341189 4

1252.ENSP00000261023->ENSP00000361125->ENSP00000338018->ENSP00000269305->ENSP00000293195->ENSP00000342087 41

1253.ENSP00000261023->ENSP00000346839->ENSP00000221930->ENSP00000262160->ENSP00000263253->ENSP00000329357->ENSP00000345571 8

1254.ENSP00000261023->ENSP00000346839 1

1255.ENSP00000261023->ENSP00000361125->ENSP00000338018->ENSP00000344818->ENSP00000316840->ENSP00000216160->ENSP00000347858 10

1256.ENSP00000261023->ENSP00000364094->ENSP00000386896->ENSP00000200181->ENSP00000340937->ENSP00000348384 11

1257.ENSP00000261023->ENSP00000346839->ENSP00000221930->ENSP00000262160->ENSP00000263253->ENSP00000367207->ENSP00000351490 8

1258.ENSP00000261023->ENSP00000364094->ENSP00000380227->ENSP00000228307->ENSP00000299421->ENSP00000270202->ENSP00000352121 12

1259.ENSP00000261023->ENSP00000364094->ENSP00000380227->ENSP00000228307->ENSP00000341189->ENSP00000350941->ENSP00000206249->ENSP00000327213->ENSP00000352900 21

1260.ENSP00000261023->ENSP00000361125->ENSP00000338018->ENSP00000269305->ENSP00000353059 9

1261.ENSP00000261023->ENSP00000364094->ENSP00000282588->ENSP00000364979->ENSP00000353654 14

1262.ENSP00000261023->ENSP00000364094->ENSP00000380227->ENSP00000228307->ENSP00000341189->ENSP00000339007->ENSP00000312435->ENSP00000400365->ENSP00000354360 20

1263.ENSP00000261023->ENSP00000361125->ENSP00000338018->ENSP00000269305->ENSP00000267163->ENSP00000355249 9

1264.ENSP00000261023->ENSP00000364094->ENSP00000380227->ENSP00000228307->ENSP00000341189->ENSP00000350941->ENSP00000264657->ENSP00000258743->ENSP00000356438 11

1265.ENSP00000261023->ENSP00000361125->ENSP00000338018->ENSP00000344818->ENSP00000316840->ENSP00000358622 9

1266.ENSP00000261023->ENSP00000346839->ENSP00000221930->ENSP00000262160->ENSP00000263253->ENSP00000384273->ENSP00000359424 8

1267.ENSP00000261023->ENSP00000364094->ENSP00000380227->ENSP00000228307->ENSP00000341189->ENSP00000361021 6

1268.ENSP00000261023->ENSP00000361125->ENSP00000338018->ENSP00000269305->ENSP00000372023->ENSP00000321656->ENSP00000300093->ENSP00000263753->ENSP00000418447->ENSP00000348784->ENSP00000379866->ENSP00000379823->ENSP00000331902->ENSP00000361290 51

1269.ENSP00000261023->ENSP00000361125->ENSP00000338018->ENSP00000344818->ENSP00000316840->ENSP00000361359->ENSP00000362994 10

1270.ENSP00000261023->ENSP00000364094->ENSP00000380227->ENSP00000228307->ENSP00000341189->ENSP00000350941->ENSP00000206249->ENSP00000327213->ENSP00000363812 33

1271.ENSP00000261023->ENSP00000364094 1

1272.ENSP00000261023->ENSP00000364094->ENSP00000282588->ENSP00000364979 13

1273.ENSP00000261023->ENSP00000364094->ENSP00000380227->ENSP00000228307->ENSP00000341189->ENSP00000361021->ENSP00000366563 18

1274.ENSP00000261023->ENSP00000346839->ENSP00000221930->ENSP00000262160->ENSP00000263253->ENSP00000367207 7

1275.ENSP00000261023->ENSP00000364094->ENSP00000380227->ENSP00000228307->ENSP00000341189->ENSP00000350941->ENSP00000206249->ENSP00000254227->ENSP00000342470->ENSP00000363868->ENSP00000372703 126

1276.ENSP00000261023->ENSP00000364094->ENSP00000386896->ENSP00000200181->ENSP00000340937->ENSP00000264144->ENSP00000252999->ENSP00000258341->ENSP00000374309 26

1277.ENSP00000261023->ENSP00000364094->ENSP00000380227->ENSP00000228307->ENSP00000341189->ENSP00000339007->ENSP00000304895->ENSP00000348986->ENSP00000375892 12

1278.ENSP00000261023->ENSP00000364094->ENSP00000380227->ENSP00000228307->ENSP00000341189->ENSP00000350941->ENSP00000264657->ENSP00000376765 9

1279.ENSP00000261023->ENSP00000361125->ENSP00000338018->ENSP00000269305->ENSP00000372023->ENSP00000321656->ENSP00000300093->ENSP00000263753->ENSP00000418447->ENSP00000348784->ENSP00000379866 48

1280.ENSP00000261023->ENSP00000361125->ENSP00000338018->ENSP00000269305->ENSP00000324897->ENSP00000381648 9

1281.ENSP00000261023->ENSP00000346839->ENSP00000221930->ENSP00000262160->ENSP00000263253->ENSP00000384273 7

1282.ENSP00000261023->ENSP00000364094->ENSP00000386896 2

1283.ENSP00000261023->ENSP00000364094->ENSP00000380227->ENSP00000228307->ENSP00000341189->ENSP00000350941->ENSP00000206249->ENSP00000254227->ENSP00000342470->ENSP00000363868->ENSP00000393286 126

1284.ENSP00000261023->ENSP00000364094->ENSP00000380227->ENSP00000228307->ENSP00000341189->ENSP00000350941->ENSP00000206249->ENSP00000254227->ENSP00000342470->ENSP00000363868->ENSP00000393870 126

1285.ENSP00000261023->ENSP00000346839->ENSP00000221930->ENSP00000262160->ENSP00000287727->ENSP00000400104 106

1286.ENSP00000261023->ENSP00000364094->ENSP00000380227->ENSP00000228307->ENSP00000341189->ENSP00000339007->ENSP00000312435->ENSP00000400365 19

1287.ENSP00000261023->ENSP00000364094->ENSP00000380227->ENSP00000228307->ENSP00000341189->ENSP00000350941->ENSP00000206249->ENSP00000254227->ENSP00000342470->ENSP00000363868->ENSP00000402590 126

1288.ENSP00000261023->ENSP00000364094->ENSP00000380227->ENSP00000228307->ENSP00000341189->ENSP00000350941->ENSP00000206249->ENSP00000399968->ENSP00000419692 8

1289.ENSP00000261464->ENSP00000269485->ENSP00000316840->ENSP00000344818->ENSP00000275493->ENSP00000401303->ENSP00000172229->ENSP00000262395 32

1290.ENSP00000261464->ENSP00000269485->ENSP00000316840->ENSP00000344818->ENSP00000275493->ENSP00000339007->ENSP00000341189->ENSP00000262017->ENSP00000262407 14

1291.ENSP00000261464->ENSP00000269485->ENSP00000316840->ENSP00000344818->ENSP00000269305->ENSP00000266970->ENSP00000262643 8

1292.ENSP00000261464->ENSP00000269485->ENSP00000316840->ENSP00000344818->ENSP00000275493->ENSP00000340944->ENSP00000304895->ENSP00000262741 11

1293.ENSP00000261464->ENSP00000263932->ENSP00000247668->ENSP00000359424->ENSP00000384273->ENSP00000362649->ENSP00000267163->ENSP00000262904 8

1294.ENSP00000261464->ENSP00000263932->ENSP00000247668->ENSP00000359424->ENSP00000384273->ENSP00000263253->ENSP00000262160->ENSP00000341551->ENSP00000262971 12

1295.ENSP00000261464->ENSP00000263932->ENSP00000247668->ENSP00000263464 4

1296.ENSP00000261464->ENSP00000269485->ENSP00000316840->ENSP00000344818->ENSP00000338018->ENSP00000361125->ENSP00000263826 27

1297.ENSP00000261464->ENSP00000269485->ENSP00000316840->ENSP00000344818->ENSP00000275493->ENSP00000263967 9

1298.ENSP00000261464->ENSP00000269485->ENSP00000316840->ENSP00000344818->ENSP00000275493->ENSP00000300574->ENSP00000228307->ENSP00000380227->ENSP00000364094->ENSP00000386896->ENSP00000200181->ENSP00000340937->ENSP00000264144 27

1299.ENSP00000261464->ENSP00000269485->ENSP00000316840->ENSP00000344818->ENSP00000269305->ENSP00000355153->ENSP00000265734 8

1300.ENSP00000261464->ENSP00000269485->ENSP00000316840->ENSP00000344818->ENSP00000269305->ENSP00000266970 7

1301.ENSP00000261464->ENSP00000263932->ENSP00000247668->ENSP00000359424->ENSP00000384273->ENSP00000362649->ENSP00000267163 7

1302.ENSP00000261464->ENSP00000269485->ENSP00000316840->ENSP00000344818->ENSP00000417281->ENSP00000270202->ENSP00000352121->ENSP00000269300 16

1303.ENSP00000261464->ENSP00000269485->ENSP00000316840->ENSP00000344818->ENSP00000269305 6

1304.ENSP00000261464->ENSP00000269485->ENSP00000316840->ENSP00000344818->ENSP00000417281->ENSP00000270202 7

1305.ENSP00000261464->ENSP00000263932->ENSP00000247668->ENSP00000359424->ENSP00000216797->ENSP00000359206->ENSP00000326804->ENSP00000274255 8

1306.ENSP00000261464->ENSP00000269485->ENSP00000316840->ENSP00000344818->ENSP00000275493->ENSP00000264033->ENSP00000274335 8

1307.ENSP00000261464->ENSP00000263932->ENSP00000247668->ENSP00000359424->ENSP00000384273->ENSP00000362649->ENSP00000267163->ENSP00000257904->ENSP00000276925 9

1308.ENSP00000261464->ENSP00000269485->ENSP00000316840->ENSP00000344818->ENSP00000417281->ENSP00000270202->ENSP00000289153 11

1309.ENSP00000261464->ENSP00000269485->ENSP00000316840->ENSP00000344818->ENSP00000275493->ENSP00000300574->ENSP00000228307->ENSP00000380227->ENSP00000364094->ENSP00000296585 12

1310.ENSP00000261464->ENSP00000269485->ENSP00000316840->ENSP00000344818->ENSP00000269305->ENSP00000302564 8

1311.ENSP00000261464->ENSP00000269485->ENSP00000316840->ENSP00000344818->ENSP00000417281->ENSP00000270202->ENSP00000348461->ENSP00000356505->ENSP00000220764->ENSP00000348380->ENSP00000305638 1025

1312.ENSP00000261464->ENSP00000269485->ENSP00000316840->ENSP00000344818->ENSP00000269305->ENSP00000278916->ENSP00000321656->ENSP00000256442->ENSP00000342307->ENSP00000230538->ENSP00000354360->ENSP00000252999->ENSP00000307156 31

1313.ENSP00000261464->ENSP00000269485->ENSP00000316840->ENSP00000344818->ENSP00000269305->ENSP00000353059->ENSP00000307786 14

1314.ENSP00000261464->ENSP00000269485->ENSP00000316840->ENSP00000344818->ENSP00000269305->ENSP00000266970->ENSP00000309181 8

1315.ENSP00000261464->ENSP00000269485->ENSP00000316840->ENSP00000344818->ENSP00000269305->ENSP00000266970->ENSP00000311083 8

1316.ENSP00000261464->ENSP00000269485->ENSP00000316840->ENSP00000344818->ENSP00000275493->ENSP00000300574->ENSP00000228307->ENSP00000380227->ENSP00000364094->ENSP00000386896->ENSP00000200181->ENSP00000340937->ENSP00000348384->ENSP00000324532 22

1317.ENSP00000261464->ENSP00000263932->ENSP00000247668->ENSP00000359424->ENSP00000226574->ENSP00000327251 24

1318.ENSP00000261464->ENSP00000263932->ENSP00000247668->ENSP00000259808->ENSP00000351273->ENSP00000329623 7

1319.ENSP00000261464->ENSP00000269485->ENSP00000316840->ENSP00000216160->ENSP00000347858->ENSP00000330237 7

1320.ENSP00000261464->ENSP00000263932->ENSP00000247668->ENSP00000359424->ENSP00000384273->ENSP00000263253->ENSP00000206249->ENSP00000399968->ENSP00000419692->ENSP00000332296 25

1321.ENSP00000261464->ENSP00000361359->ENSP00000332468 5

1322.ENSP00000261464->ENSP00000263932->ENSP00000247668->ENSP00000339151 5

1323.ENSP00000261464->ENSP00000269485->ENSP00000316840->ENSP00000344818->ENSP00000275493->ENSP00000339007->ENSP00000341189 8

1324.ENSP00000261464->ENSP00000269485->ENSP00000316840->ENSP00000344818->ENSP00000269305->ENSP00000293195->ENSP00000342087 40

1325.ENSP00000261464->ENSP00000263932->ENSP00000247668->ENSP00000359424->ENSP00000384273->ENSP00000263253->ENSP00000329357->ENSP00000345571 8

1326.ENSP00000261464->ENSP00000269485->ENSP00000316840->ENSP00000344818->ENSP00000275493->ENSP00000265171->ENSP00000346839 11

1327.ENSP00000261464->ENSP00000269485->ENSP00000316840->ENSP00000216160->ENSP00000347858 6

1328.ENSP00000261464->ENSP00000269485->ENSP00000316840->ENSP00000344818->ENSP00000275493->ENSP00000300574->ENSP00000228307->ENSP00000380227->ENSP00000364094->ENSP00000386896->ENSP00000200181->ENSP00000340937->ENSP00000348384 21

1329.ENSP00000261464->ENSP00000263932->ENSP00000247668->ENSP00000359424->ENSP00000384273->ENSP00000263253->ENSP00000367207->ENSP00000351490 8

1330.ENSP00000261464->ENSP00000269485->ENSP00000316840->ENSP00000344818->ENSP00000417281->ENSP00000270202->ENSP00000352121 13

1331.ENSP00000261464->ENSP00000263932->ENSP00000247668->ENSP00000359424->ENSP00000384273->ENSP00000263253->ENSP00000206249->ENSP00000327213->ENSP00000352900 22

1332.ENSP00000261464->ENSP00000269485->ENSP00000316840->ENSP00000344818->ENSP00000269305->ENSP00000353059 8

1333.ENSP00000261464->ENSP00000269485->ENSP00000316840->ENSP00000344818->ENSP00000275493->ENSP00000300574->ENSP00000228307->ENSP00000380227->ENSP00000364094->ENSP00000282588->ENSP00000364979->ENSP00000353654 24

1334.ENSP00000261464->ENSP00000269485->ENSP00000316840->ENSP00000344818->ENSP00000269305->ENSP00000278916->ENSP00000321656->ENSP00000256442->ENSP00000342307->ENSP00000230538->ENSP00000354360 21

1335.ENSP00000261464->ENSP00000263932->ENSP00000247668->ENSP00000359424->ENSP00000384273->ENSP00000362649->ENSP00000267163->ENSP00000355249 8

1336.ENSP00000261464->ENSP00000269485->ENSP00000316840->ENSP00000358997->ENSP00000263341->ENSP00000356438 9

1337.ENSP00000261464->ENSP00000269485->ENSP00000316840->ENSP00000358622 5

1338.ENSP00000261464->ENSP00000263932->ENSP00000247668->ENSP00000359424 4

1339.ENSP00000261464->ENSP00000269485->ENSP00000316840->ENSP00000344818->ENSP00000269305->ENSP00000361021 7

1340.ENSP00000261464->ENSP00000269485->ENSP00000316840->ENSP00000344818->ENSP00000269305->ENSP00000278916->ENSP00000312995->ENSP00000300093->ENSP00000263753->ENSP00000418447->ENSP00000348784->ENSP00000379866->ENSP00000379823->ENSP00000331902->ENSP00000361290 50

1341.ENSP00000261464->ENSP00000361359->ENSP00000362994 5

1342.ENSP00000261464->ENSP00000263932->ENSP00000247668->ENSP00000359424->ENSP00000384273->ENSP00000263253->ENSP00000206249->ENSP00000327213->ENSP00000363812 34

1343.ENSP00000261464->ENSP00000269485->ENSP00000316840->ENSP00000344818->ENSP00000275493->ENSP00000300574->ENSP00000228307->ENSP00000380227->ENSP00000364094 11

1344.ENSP00000261464->ENSP00000269485->ENSP00000316840->ENSP00000344818->ENSP00000275493->ENSP00000300574->ENSP00000228307->ENSP00000380227->ENSP00000364094->ENSP00000282588->ENSP00000364979 23

1345.ENSP00000261464->ENSP00000269485->ENSP00000316840->ENSP00000344818->ENSP00000269305->ENSP00000361021->ENSP00000366563 19

1346.ENSP00000261464->ENSP00000263932->ENSP00000247668->ENSP00000359424->ENSP00000384273->ENSP00000263253->ENSP00000367207 7

1347.ENSP00000261464->ENSP00000263932->ENSP00000247668->ENSP00000359424->ENSP00000384273->ENSP00000263253->ENSP00000206249->ENSP00000254227->ENSP00000342470->ENSP00000363868->ENSP00000372703 127

1348.ENSP00000261464->ENSP00000269485->ENSP00000316840->ENSP00000344818->ENSP00000269305->ENSP00000278916->ENSP00000321656->ENSP00000256442->ENSP00000342307->ENSP00000230538->ENSP00000222399->ENSP00000374309 29

1349.ENSP00000261464->ENSP00000269485->ENSP00000316840->ENSP00000344818->ENSP00000417281->ENSP00000270202->ENSP00000348986->ENSP00000375892 13

1350.ENSP00000261464->ENSP00000263932->ENSP00000247668->ENSP00000359424->ENSP00000384273->ENSP00000263253->ENSP00000264657->ENSP00000376765 10

1351.ENSP00000261464->ENSP00000269485->ENSP00000316840->ENSP00000344818->ENSP00000269305->ENSP00000278916->ENSP00000312995->ENSP00000300093->ENSP00000263753->ENSP00000418447->ENSP00000348784->ENSP00000379866 47

1352.ENSP00000261464->ENSP00000269485->ENSP00000316840->ENSP00000344818->ENSP00000269305->ENSP00000324897->ENSP00000381648 8

1353.ENSP00000261464->ENSP00000263932->ENSP00000247668->ENSP00000359424->ENSP00000384273 5

1354.ENSP00000261464->ENSP00000269485->ENSP00000316840->ENSP00000344818->ENSP00000275493->ENSP00000300574->ENSP00000228307->ENSP00000380227->ENSP00000364094->ENSP00000386896 12

1355.ENSP00000261464->ENSP00000263932->ENSP00000247668->ENSP00000359424->ENSP00000384273->ENSP00000263253->ENSP00000206249->ENSP00000254227->ENSP00000342470->ENSP00000363868->ENSP00000393286 127

1356.ENSP00000261464->ENSP00000263932->ENSP00000247668->ENSP00000359424->ENSP00000384273->ENSP00000263253->ENSP00000206249->ENSP00000254227->ENSP00000342470->ENSP00000363868->ENSP00000393870 127

1357.ENSP00000261464->ENSP00000263932->ENSP00000247668->ENSP00000359424->ENSP00000384273->ENSP00000263253->ENSP00000262160->ENSP00000287727->ENSP00000400104 108

1358.ENSP00000261464->ENSP00000269485->ENSP00000316840->ENSP00000344818->ENSP00000275493->ENSP00000339007->ENSP00000312435->ENSP00000400365 21

1359.ENSP00000261464->ENSP00000263932->ENSP00000247668->ENSP00000359424->ENSP00000384273->ENSP00000263253->ENSP00000206249->ENSP00000254227->ENSP00000342470->ENSP00000363868->ENSP00000402590 127

1360.ENSP00000261464->ENSP00000263932->ENSP00000247668->ENSP00000359424->ENSP00000384273->ENSP00000263253->ENSP00000206249->ENSP00000399968->ENSP00000419692 9

1361.ENSP00000262395->ENSP00000172229->ENSP00000351486->ENSP00000339007->ENSP00000341189->ENSP00000262017->ENSP00000262407 33

1362.ENSP00000262395->ENSP00000172229->ENSP00000332643->ENSP00000345571->ENSP00000266970->ENSP00000262643 30

1363.ENSP00000262395->ENSP00000172229->ENSP00000401303->ENSP00000304895->ENSP00000262741 30

1364.ENSP00000262395->ENSP00000172229->ENSP00000332643->ENSP00000345571->ENSP00000267163->ENSP00000262904 29

1365.ENSP00000262395->ENSP00000172229->ENSP00000332643->ENSP00000345571->ENSP00000329357->ENSP00000332973->ENSP00000341551->ENSP00000262971 34

1366.ENSP00000262395->ENSP00000172229->ENSP00000401303->ENSP00000275493->ENSP00000344818->ENSP00000316840->ENSP00000361359->ENSP00000263464 31

1367.ENSP00000262395->ENSP00000172229->ENSP00000351486->ENSP00000244007->ENSP00000263923->ENSP00000361125->ENSP00000263826 48

1368.ENSP00000262395->ENSP00000172229->ENSP00000401303->ENSP00000274335->ENSP00000263967 27

1369.ENSP00000262395->ENSP00000172229->ENSP00000351486->ENSP00000339007->ENSP00000341189->ENSP00000228307->ENSP00000380227->ENSP00000364094->ENSP00000386896->ENSP00000200181->ENSP00000340937->ENSP00000264144 46

1370.ENSP00000262395->ENSP00000172229->ENSP00000332643->ENSP00000345571->ENSP00000227507->ENSP00000265734 29

1371.ENSP00000262395->ENSP00000172229->ENSP00000332643->ENSP00000345571->ENSP00000266970 29

1372.ENSP00000262395->ENSP00000172229->ENSP00000332643->ENSP00000345571->ENSP00000267163 28

1373.ENSP00000262395->ENSP00000172229->ENSP00000401303->ENSP00000384675->ENSP00000309845->ENSP00000352121->ENSP00000269300 37

1374.ENSP00000262395->ENSP00000172229->ENSP00000401303->ENSP00000275493->ENSP00000344818->ENSP00000269305 28

1375.ENSP00000262395->ENSP00000172229->ENSP00000401303->ENSP00000303830->ENSP00000348986->ENSP00000270202 28

1376.ENSP00000262395->ENSP00000172229->ENSP00000332643->ENSP00000345571->ENSP00000227507->ENSP00000228872->ENSP00000274255 30

1377.ENSP00000262395->ENSP00000172229->ENSP00000401303->ENSP00000274335 26

1378.ENSP00000262395->ENSP00000172229->ENSP00000332643->ENSP00000345571->ENSP00000227507->ENSP00000257904->ENSP00000276925 30

1379.ENSP00000262395->ENSP00000172229->ENSP00000401303->ENSP00000274335->ENSP00000289153 30

1380.ENSP00000262395->ENSP00000172229->ENSP00000351486->ENSP00000339007->ENSP00000341189->ENSP00000228307->ENSP00000380227->ENSP00000364094->ENSP00000296585 31

1381.ENSP00000262395->ENSP00000172229->ENSP00000401303->ENSP00000275493->ENSP00000344818->ENSP00000269305->ENSP00000302564 30

1382.ENSP00000262395->ENSP00000172229->ENSP00000351486->ENSP00000339007->ENSP00000302269->ENSP00000348461->ENSP00000356505->ENSP00000220764->ENSP00000348380->ENSP00000305638 1045

1383.ENSP00000262395->ENSP00000172229->ENSP00000351486->ENSP00000339007->ENSP00000312435->ENSP00000400365->ENSP00000354360->ENSP00000252999->ENSP00000307156 51

1384.ENSP00000262395->ENSP00000172229->ENSP00000401303->ENSP00000275493->ENSP00000344818->ENSP00000269305->ENSP00000353059->ENSP00000307786 36

1385.ENSP00000262395->ENSP00000172229->ENSP00000332643->ENSP00000345571->ENSP00000266970->ENSP00000309181 30

1386.ENSP00000262395->ENSP00000172229->ENSP00000332643->ENSP00000345571->ENSP00000266970->ENSP00000311083 30

1387.ENSP00000262395->ENSP00000172229->ENSP00000351486->ENSP00000339007->ENSP00000341189->ENSP00000228307->ENSP00000380227->ENSP00000364094->ENSP00000386896->ENSP00000200181->ENSP00000340937->ENSP00000348384->ENSP00000324532 41

1388.ENSP00000262395->ENSP00000172229->ENSP00000401303->ENSP00000371067->ENSP00000264657->ENSP00000263253->ENSP00000384273->ENSP00000226574->ENSP00000327251 49

1389.ENSP00000262395->ENSP00000172229->ENSP00000401303->ENSP00000275493->ENSP00000344818->ENSP00000269305->ENSP00000329623 29

1390.ENSP00000262395->ENSP00000172229->ENSP00000401303->ENSP00000275493->ENSP00000344818->ENSP00000269305->ENSP00000353059->ENSP00000330237 31

1391.ENSP00000262395->ENSP00000172229->ENSP00000401303->ENSP00000268035->ENSP00000206249->ENSP00000399968->ENSP00000419692->ENSP00000332296 45

1392.ENSP00000262395->ENSP00000172229->ENSP00000401303->ENSP00000275493->ENSP00000344818->ENSP00000316840->ENSP00000361359->ENSP00000332468 30

1393.ENSP00000262395->ENSP00000172229->ENSP00000401303->ENSP00000371067->ENSP00000264657->ENSP00000263253->ENSP00000384273->ENSP00000339151 30

1394.ENSP00000262395->ENSP00000172229->ENSP00000351486->ENSP00000339007->ENSP00000341189 27

1395.ENSP00000262395->ENSP00000172229->ENSP00000401303->ENSP00000275493->ENSP00000344818->ENSP00000269305->ENSP00000293195->ENSP00000342087 62

1396.ENSP00000262395->ENSP00000172229->ENSP00000332643->ENSP00000345571 27

1397.ENSP00000262395->ENSP00000172229->ENSP00000351486->ENSP00000339007->ENSP00000341189->ENSP00000228307->ENSP00000380227->ENSP00000346839 30

1398.ENSP00000262395->ENSP00000172229->ENSP00000401303->ENSP00000275493->ENSP00000344818->ENSP00000316840->ENSP00000216160->ENSP00000347858 30

1399.ENSP00000262395->ENSP00000172229->ENSP00000351486->ENSP00000339007->ENSP00000341189->ENSP00000228307->ENSP00000380227->ENSP00000364094->ENSP00000386896->ENSP00000200181->ENSP00000340937->ENSP00000348384 40

1400.ENSP00000262395->ENSP00000172229->ENSP00000332643->ENSP00000345571->ENSP00000367207->ENSP00000351490 30

1401.ENSP00000262395->ENSP00000172229->ENSP00000401303->ENSP00000384675->ENSP00000309845->ENSP00000352121 34

1402.ENSP00000262395->ENSP00000172229->ENSP00000401303->ENSP00000268035->ENSP00000206249->ENSP00000327213->ENSP00000352900 42

1403.ENSP00000262395->ENSP00000172229->ENSP00000401303->ENSP00000275493->ENSP00000344818->ENSP00000269305->ENSP00000353059 30

1404.ENSP00000262395->ENSP00000172229->ENSP00000351486->ENSP00000339007->ENSP00000341189->ENSP00000228307->ENSP00000380227->ENSP00000364094->ENSP00000282588->ENSP00000364979->ENSP00000353654 43

1405.ENSP00000262395->ENSP00000172229->ENSP00000351486->ENSP00000339007->ENSP00000312435->ENSP00000400365->ENSP00000354360 41

1406.ENSP00000262395->ENSP00000172229->ENSP00000332643->ENSP00000345571->ENSP00000267163->ENSP00000355249 29

1407.ENSP00000262395->ENSP00000172229->ENSP00000401303->ENSP00000371067->ENSP00000264657->ENSP00000258743->ENSP00000356438 32

1408.ENSP00000262395->ENSP00000172229->ENSP00000401303->ENSP00000275493->ENSP00000344818->ENSP00000316840->ENSP00000358622 29

1409.ENSP00000262395->ENSP00000172229->ENSP00000401303->ENSP00000371067->ENSP00000264657->ENSP00000263253->ENSP00000384273->ENSP00000359424 30

1410.ENSP00000262395->ENSP00000172229->ENSP00000351486->ENSP00000339007->ENSP00000341189->ENSP00000361021 29

1411.ENSP00000262395->ENSP00000172229->ENSP00000332643->ENSP00000345571->ENSP00000274026->ENSP00000306043->ENSP00000256442->ENSP00000300093->ENSP00000263753->ENSP00000418447->ENSP00000348784->ENSP00000379866->ENSP00000379823->ENSP00000331902->ENSP00000361290 72

1412.ENSP00000262395->ENSP00000172229->ENSP00000401303->ENSP00000275493->ENSP00000344818->ENSP00000316840->ENSP00000361359->ENSP00000362994 30

1413.ENSP00000262395->ENSP00000172229->ENSP00000401303->ENSP00000268035->ENSP00000206249->ENSP00000327213->ENSP00000363812 54

1414.ENSP00000262395->ENSP00000172229->ENSP00000351486->ENSP00000339007->ENSP00000341189->ENSP00000228307->ENSP00000380227->ENSP00000364094 30

1415.ENSP00000262395->ENSP00000172229->ENSP00000351486->ENSP00000339007->ENSP00000341189->ENSP00000228307->ENSP00000380227->ENSP00000364094->ENSP00000282588->ENSP00000364979 42

1416.ENSP00000262395->ENSP00000172229->ENSP00000351486->ENSP00000339007->ENSP00000341189->ENSP00000361021->ENSP00000366563 41

1417.ENSP00000262395->ENSP00000172229->ENSP00000332643->ENSP00000345571->ENSP00000367207 29

1418.ENSP00000262395->ENSP00000172229->ENSP00000401303->ENSP00000268035->ENSP00000206249->ENSP00000254227->ENSP00000342470->ENSP00000363868->ENSP00000372703 147

1419.ENSP00000262395->ENSP00000172229->ENSP00000351486->ENSP00000339007->ENSP00000312435->ENSP00000400365->ENSP00000222399->ENSP00000374309 50

1420.ENSP00000262395->ENSP00000172229->ENSP00000401303->ENSP00000303830->ENSP00000348986->ENSP00000375892 32

1421.ENSP00000262395->ENSP00000172229->ENSP00000401303->ENSP00000371067->ENSP00000264657->ENSP00000376765 30

1422.ENSP00000262395->ENSP00000172229->ENSP00000332643->ENSP00000345571->ENSP00000274026->ENSP00000306043->ENSP00000256442->ENSP00000300093->ENSP00000263753->ENSP00000418447->ENSP00000348784->ENSP00000379866 69

1423.ENSP00000262395->ENSP00000172229->ENSP00000401303->ENSP00000371067->ENSP00000354394->ENSP00000249636->ENSP00000376076->ENSP00000381648 30

1424.ENSP00000262395->ENSP00000172229->ENSP00000401303->ENSP00000371067->ENSP00000264657->ENSP00000263253->ENSP00000384273 29

1425.ENSP00000262395->ENSP00000172229->ENSP00000351486->ENSP00000339007->ENSP00000341189->ENSP00000228307->ENSP00000380227->ENSP00000364094->ENSP00000386896 31

1426.ENSP00000262395->ENSP00000172229->ENSP00000401303->ENSP00000268035->ENSP00000206249->ENSP00000254227->ENSP00000342470->ENSP00000363868->ENSP00000393286 147

1427.ENSP00000262395->ENSP00000172229->ENSP00000401303->ENSP00000268035->ENSP00000206249->ENSP00000254227->ENSP00000342470->ENSP00000363868->ENSP00000393870 147

1428.ENSP00000262395->ENSP00000172229->ENSP00000332643->ENSP00000345571->ENSP00000329357->ENSP00000332973->ENSP00000287727->ENSP00000400104 130

1429.ENSP00000262395->ENSP00000172229->ENSP00000351486->ENSP00000339007->ENSP00000312435->ENSP00000400365 40

1430.ENSP00000262395->ENSP00000172229->ENSP00000401303->ENSP00000268035->ENSP00000206249->ENSP00000254227->ENSP00000342470->ENSP00000363868->ENSP00000402590 147

1431.ENSP00000262395->ENSP00000172229->ENSP00000401303->ENSP00000268035->ENSP00000206249->ENSP00000399968->ENSP00000419692 29

1432.ENSP00000262407->ENSP00000262017->ENSP00000261023->ENSP00000364094->ENSP00000380227->ENSP00000228307->ENSP00000299421->ENSP00000270202->ENSP00000228872->ENSP00000262643 10

1433.ENSP00000262407->ENSP00000262017->ENSP00000341189->ENSP00000339007->ENSP00000304895->ENSP00000262741 11

1434.ENSP00000262407->ENSP00000262017->ENSP00000261023->ENSP00000346839->ENSP00000221930->ENSP00000262160->ENSP00000263253->ENSP00000269305->ENSP00000267163->ENSP00000262904 11

1435.ENSP00000262407->ENSP00000262017->ENSP00000261023->ENSP00000346839->ENSP00000221930->ENSP00000364133->ENSP00000341551->ENSP00000262971 12

1436.ENSP00000262407->ENSP00000262017->ENSP00000261023->ENSP00000346839->ENSP00000221930->ENSP00000262160->ENSP00000263253->ENSP00000384273->ENSP00000359424->ENSP00000247668->ENSP00000263464 12

1437.ENSP00000262407->ENSP00000262017->ENSP00000261023->ENSP00000361125->ENSP00000263826 27

1438.ENSP00000262407->ENSP00000262017->ENSP00000341189->ENSP00000339007->ENSP00000274335->ENSP00000263967 9

1439.ENSP00000262407->ENSP00000262017->ENSP00000261023->ENSP00000364094->ENSP00000386896->ENSP00000200181->ENSP00000340937->ENSP00000264144 19

1440.ENSP00000262407->ENSP00000262017->ENSP00000261023->ENSP00000364094->ENSP00000380227->ENSP00000228307->ENSP00000299421->ENSP00000270202->ENSP00000228872->ENSP00000265734 10

1441.ENSP00000262407->ENSP00000262017->ENSP00000261023->ENSP00000346839->ENSP00000221930->ENSP00000262160->ENSP00000263253->ENSP00000269305->ENSP00000266970 10

1442.ENSP00000262407->ENSP00000262017->ENSP00000261023->ENSP00000346839->ENSP00000221930->ENSP00000262160->ENSP00000263253->ENSP00000269305->ENSP00000267163 10

1443.ENSP00000262407->ENSP00000262017->ENSP00000261023->ENSP00000364094->ENSP00000380227->ENSP00000228307->ENSP00000299421->ENSP00000270202->ENSP00000352121->ENSP00000269300 17

1444.ENSP00000262407->ENSP00000262017->ENSP00000261023->ENSP00000346839->ENSP00000221930->ENSP00000262160->ENSP00000263253->ENSP00000269305 9

1445.ENSP00000262407->ENSP00000262017->ENSP00000261023->ENSP00000364094->ENSP00000380227->ENSP00000228307->ENSP00000299421->ENSP00000270202 8

1446.ENSP00000262407->ENSP00000262017->ENSP00000261023->ENSP00000364094->ENSP00000380227->ENSP00000228307->ENSP00000299421->ENSP00000270202->ENSP00000228872->ENSP00000274255 10

1447.ENSP00000262407->ENSP00000262017->ENSP00000341189->ENSP00000339007->ENSP00000274335 8

1448.ENSP00000262407->ENSP00000262017->ENSP00000261023->ENSP00000364094->ENSP00000380227->ENSP00000228307->ENSP00000299421->ENSP00000270202->ENSP00000228872->ENSP00000257904->ENSP00000276925 11

1449.ENSP00000262407->ENSP00000262017->ENSP00000341189->ENSP00000339007->ENSP00000274335->ENSP00000289153 12

1450.ENSP00000262407->ENSP00000262017->ENSP00000261023->ENSP00000364094->ENSP00000296585 4

1451.ENSP00000262407->ENSP00000262017->ENSP00000261023->ENSP00000364094->ENSP00000380227->ENSP00000228307->ENSP00000299421->ENSP00000270202->ENSP00000309103->ENSP00000302564 10

1452.ENSP00000262407->ENSP00000262017->ENSP00000261023->ENSP00000364094->ENSP00000380227->ENSP00000228307->ENSP00000300574->ENSP00000381107->ENSP00000348461->ENSP00000356505->ENSP00000220764->ENSP00000348380->ENSP00000305638 1025

1453.ENSP00000262407->ENSP00000262017->ENSP00000261023->ENSP00000364094->ENSP00000386896->ENSP00000200181->ENSP00000340937->ENSP00000264144->ENSP00000252999->ENSP00000307156 26

1454.ENSP00000262407->ENSP00000262017->ENSP00000261023->ENSP00000346839->ENSP00000221930->ENSP00000262160->ENSP00000263253->ENSP00000269305->ENSP00000353059->ENSP00000307786 17

1455.ENSP00000262407->ENSP00000262017->ENSP00000261023->ENSP00000346839->ENSP00000221930->ENSP00000262160->ENSP00000263253->ENSP00000269305->ENSP00000266970->ENSP00000309181 11

1456.ENSP00000262407->ENSP00000262017->ENSP00000261023->ENSP00000346839->ENSP00000221930->ENSP00000262160->ENSP00000263253->ENSP00000269305->ENSP00000266970->ENSP00000311083 11

1457.ENSP00000262407->ENSP00000262017->ENSP00000261023->ENSP00000364094->ENSP00000386896->ENSP00000200181->ENSP00000340937->ENSP00000348384->ENSP00000324532 14

1458.ENSP00000262407->ENSP00000262017->ENSP00000226218->ENSP00000223095->ENSP00000361850->ENSP00000226574->ENSP00000327251 29

1459.ENSP00000262407->ENSP00000262017->ENSP00000261023->ENSP00000346839->ENSP00000221930->ENSP00000262160->ENSP00000263253->ENSP00000269305->ENSP00000329623 10

1460.ENSP00000262407->ENSP00000262017->ENSP00000261023->ENSP00000346839->ENSP00000221930->ENSP00000262160->ENSP00000263253->ENSP00000269305->ENSP00000353059->ENSP00000330237 12

1461.ENSP00000262407->ENSP00000262017->ENSP00000341189->ENSP00000350941->ENSP00000206249->ENSP00000399968->ENSP00000419692->ENSP00000332296 26

1462.ENSP00000262407->ENSP00000262017->ENSP00000261023->ENSP00000364094->ENSP00000380227->ENSP00000228307->ENSP00000300574->ENSP00000275493->ENSP00000344818->ENSP00000316840->ENSP00000361359->ENSP00000332468 12

1463.ENSP00000262407->ENSP00000262017->ENSP00000261023->ENSP00000346839->ENSP00000221930->ENSP00000262160->ENSP00000263253->ENSP00000384273->ENSP00000339151 10

1464.ENSP00000262407->ENSP00000262017->ENSP00000341189 6

1465.ENSP00000262407->ENSP00000262017->ENSP00000261023->ENSP00000346839->ENSP00000221930->ENSP00000262160->ENSP00000263253->ENSP00000269305->ENSP00000293195->ENSP00000342087 43

1466.ENSP00000262407->ENSP00000262017->ENSP00000261023->ENSP00000346839->ENSP00000221930->ENSP00000262160->ENSP00000263253->ENSP00000329357->ENSP00000345571 10

1467.ENSP00000262407->ENSP00000262017->ENSP00000261023->ENSP00000346839 3

1468.ENSP00000262407->ENSP00000262017->ENSP00000261023->ENSP00000364094->ENSP00000380227->ENSP00000228307->ENSP00000300574->ENSP00000275493->ENSP00000344818->ENSP00000316840->ENSP00000216160->ENSP00000347858 12

1469.ENSP00000262407->ENSP00000262017->ENSP00000261023->ENSP00000364094->ENSP00000386896->ENSP00000200181->ENSP00000340937->ENSP00000348384 13

1470.ENSP00000262407->ENSP00000262017->ENSP00000261023->ENSP00000346839->ENSP00000221930->ENSP00000262160->ENSP00000263253->ENSP00000367207->ENSP00000351490 10

1471.ENSP00000262407->ENSP00000262017->ENSP00000261023->ENSP00000364094->ENSP00000380227->ENSP00000228307->ENSP00000299421->ENSP00000270202->ENSP00000352121 14

1472.ENSP00000262407->ENSP00000262017->ENSP00000341189->ENSP00000350941->ENSP00000206249->ENSP00000327213->ENSP00000352900 23

1473.ENSP00000262407->ENSP00000262017->ENSP00000261023->ENSP00000346839->ENSP00000221930->ENSP00000262160->ENSP00000263253->ENSP00000269305->ENSP00000353059 11

1474.ENSP00000262407->ENSP00000262017->ENSP00000261023->ENSP00000364094->ENSP00000282588->ENSP00000364979->ENSP00000353654 16

1475.ENSP00000262407->ENSP00000262017->ENSP00000341189->ENSP00000339007->ENSP00000312435->ENSP00000400365->ENSP00000354360 22

1476.ENSP00000262407->ENSP00000262017->ENSP00000261023->ENSP00000346839->ENSP00000221930->ENSP00000262160->ENSP00000263253->ENSP00000269305->ENSP00000267163->ENSP00000355249 11

1477.ENSP00000262407->ENSP00000262017->ENSP00000341189->ENSP00000350941->ENSP00000264657->ENSP00000258743->ENSP00000356438 13

1478.ENSP00000262407->ENSP00000262017->ENSP00000261023->ENSP00000346839->ENSP00000221930->ENSP00000262160->ENSP00000263253->ENSP00000384273->ENSP00000339151->ENSP00000358622 11

1479.ENSP00000262407->ENSP00000262017->ENSP00000261023->ENSP00000346839->ENSP00000221930->ENSP00000262160->ENSP00000263253->ENSP00000384273->ENSP00000359424 10

1480.ENSP00000262407->ENSP00000262017->ENSP00000341189->ENSP00000361021 8

1481.ENSP00000262407->ENSP00000262017->ENSP00000261023->ENSP00000346839->ENSP00000221930->ENSP00000262160->ENSP00000263253->ENSP00000269305->ENSP00000278916->ENSP00000312995->ENSP00000300093->ENSP00000263753->ENSP00000418447->ENSP00000348784->ENSP00000379866->ENSP00000379823->ENSP00000331902->ENSP00000361290 53

1482.ENSP00000262407->ENSP00000262017->ENSP00000261023->ENSP00000364094->ENSP00000380227->ENSP00000228307->ENSP00000300574->ENSP00000275493->ENSP00000344818->ENSP00000316840->ENSP00000361359->ENSP00000362994 12

1483.ENSP00000262407->ENSP00000262017->ENSP00000341189->ENSP00000350941->ENSP00000206249->ENSP00000327213->ENSP00000363812 35

1484.ENSP00000262407->ENSP00000262017->ENSP00000261023->ENSP00000364094 3

1485.ENSP00000262407->ENSP00000262017->ENSP00000261023->ENSP00000364094->ENSP00000282588->ENSP00000364979 15

1486.ENSP00000262407->ENSP00000262017->ENSP00000341189->ENSP00000361021->ENSP00000366563 20

1487.ENSP00000262407->ENSP00000262017->ENSP00000261023->ENSP00000346839->ENSP00000221930->ENSP00000262160->ENSP00000263253->ENSP00000367207 9

1488.ENSP00000262407->ENSP00000262017->ENSP00000341189->ENSP00000350941->ENSP00000206249->ENSP00000254227->ENSP00000342470->ENSP00000363868->ENSP00000372703 128

1489.ENSP00000262407->ENSP00000262017->ENSP00000261023->ENSP00000364094->ENSP00000386896->ENSP00000200181->ENSP00000340937->ENSP00000264144->ENSP00000252999->ENSP00000258341->ENSP00000374309 28

1490.ENSP00000262407->ENSP00000262017->ENSP00000341189->ENSP00000339007->ENSP00000304895->ENSP00000348986->ENSP00000375892 14

1491.ENSP00000262407->ENSP00000262017->ENSP00000341189->ENSP00000350941->ENSP00000264657->ENSP00000376765 11

1492.ENSP00000262407->ENSP00000262017->ENSP00000261023->ENSP00000346839->ENSP00000221930->ENSP00000262160->ENSP00000263253->ENSP00000269305->ENSP00000278916->ENSP00000312995->ENSP00000300093->ENSP00000263753->ENSP00000418447->ENSP00000348784->ENSP00000379866 50

1493.ENSP00000262407->ENSP00000262017->ENSP00000261023->ENSP00000346839->ENSP00000221930->ENSP00000262160->ENSP00000263253->ENSP00000269305->ENSP00000324897->ENSP00000381648 11

1494.ENSP00000262407->ENSP00000262017->ENSP00000261023->ENSP00000346839->ENSP00000221930->ENSP00000262160->ENSP00000263253->ENSP00000384273 9

1495.ENSP00000262407->ENSP00000262017->ENSP00000261023->ENSP00000364094->ENSP00000386896 4

1496.ENSP00000262407->ENSP00000262017->ENSP00000341189->ENSP00000350941->ENSP00000206249->ENSP00000254227->ENSP00000342470->ENSP00000363868->ENSP00000393286 128

1497.ENSP00000262407->ENSP00000262017->ENSP00000341189->ENSP00000350941->ENSP00000206249->ENSP00000254227->ENSP00000342470->ENSP00000363868->ENSP00000393870 128

1498.ENSP00000262407->ENSP00000262017->ENSP00000261023->ENSP00000346839->ENSP00000221930->ENSP00000262160->ENSP00000287727->ENSP00000400104 108

1499.ENSP00000262407->ENSP00000262017->ENSP00000341189->ENSP00000339007->ENSP00000312435->ENSP00000400365 21

1500.ENSP00000262407->ENSP00000262017->ENSP00000341189->ENSP00000350941->ENSP00000206249->ENSP00000254227->ENSP00000342470->ENSP00000363868->ENSP00000402590 128

1501.ENSP00000262407->ENSP00000262017->ENSP00000341189->ENSP00000350941->ENSP00000206249->ENSP00000399968->ENSP00000419692 10

1502.ENSP00000262643->ENSP00000228872->ENSP00000270202->ENSP00000348986->ENSP00000304895->ENSP00000262741 7

1503.ENSP00000262643->ENSP00000266970->ENSP00000267163->ENSP00000262904 3

1504.ENSP00000262643->ENSP00000281708->ENSP00000367207->ENSP00000263253->ENSP00000262160->ENSP00000341551->ENSP00000262971 9

1505.ENSP00000262643->ENSP00000244741->ENSP00000344818->ENSP00000316840->ENSP00000361359->ENSP00000263464 7

1506.ENSP00000262643->ENSP00000266970->ENSP00000269305->ENSP00000338018->ENSP00000361125->ENSP00000263826 24

1507.ENSP00000262643->ENSP00000228872->ENSP00000270202->ENSP00000263967 4

1508.ENSP00000262643->ENSP00000228872->ENSP00000270202->ENSP00000299421->ENSP00000228307->ENSP00000380227->ENSP00000364094->ENSP00000386896->ENSP00000200181->ENSP00000340937->ENSP00000264144 23

1509.ENSP00000262643->ENSP00000244741->ENSP00000265734 2

1510.ENSP00000262643->ENSP00000266970 1

1511.ENSP00000262643->ENSP00000266970->ENSP00000267163 2

1512.ENSP00000262643->ENSP00000228872->ENSP00000270202->ENSP00000352121->ENSP00000269300 11

1513.ENSP00000262643->ENSP00000266970->ENSP00000269305 2

1514.ENSP00000262643->ENSP00000228872->ENSP00000270202 2

1515.ENSP00000262643->ENSP00000228872->ENSP00000274255 2

1516.ENSP00000262643->ENSP00000228872->ENSP00000270202->ENSP00000274335 5

1517.ENSP00000262643->ENSP00000244741->ENSP00000257904->ENSP00000276925 3

1518.ENSP00000262643->ENSP00000228872->ENSP00000270202->ENSP00000289153 6

1519.ENSP00000262643->ENSP00000228872->ENSP00000270202->ENSP00000299421->ENSP00000228307->ENSP00000380227->ENSP00000364094->ENSP00000296585 8

1520.ENSP00000262643->ENSP00000266970->ENSP00000269305->ENSP00000302564 4

1521.ENSP00000262643->ENSP00000228872->ENSP00000270202->ENSP00000348461->ENSP00000356505->ENSP00000220764->ENSP00000348380->ENSP00000305638 1020

1522.ENSP00000262643->ENSP00000266970->ENSP00000255465->ENSP00000306043->ENSP00000342307->ENSP00000230538->ENSP00000354360->ENSP00000252999->ENSP00000307156 26

1523.ENSP00000262643->ENSP00000266970->ENSP00000269305->ENSP00000353059->ENSP00000307786 10

1524.ENSP00000262643->ENSP00000266970->ENSP00000309181 2

1525.ENSP00000262643->ENSP00000266970->ENSP00000311083 2

1526.ENSP00000262643->ENSP00000228872->ENSP00000270202->ENSP00000299421->ENSP00000228307->ENSP00000380227->ENSP00000364094->ENSP00000386896->ENSP00000200181->ENSP00000340937->ENSP00000348384->ENSP00000324532 18

1527.ENSP00000262643->ENSP00000281708->ENSP00000231487->ENSP00000359206->ENSP00000216797->ENSP00000226574->ENSP00000327251 24

1528.ENSP00000262643->ENSP00000266970->ENSP00000269305->ENSP00000329623 3

1529.ENSP00000262643->ENSP00000266970->ENSP00000269305->ENSP00000353059->ENSP00000330237 5

1530.ENSP00000262643->ENSP00000281708->ENSP00000367207->ENSP00000263253->ENSP00000325690->ENSP00000399968->ENSP00000419692->ENSP00000332296 22

1531.ENSP00000262643->ENSP00000244741->ENSP00000344818->ENSP00000316840->ENSP00000361359->ENSP00000332468 6

1532.ENSP00000262643->ENSP00000281708->ENSP00000231487->ENSP00000359206->ENSP00000216797->ENSP00000339151 5

1533.ENSP00000262643->ENSP00000266970->ENSP00000269305->ENSP00000361021->ENSP00000341189 5

1534.ENSP00000262643->ENSP00000266970->ENSP00000269305->ENSP00000293195->ENSP00000342087 36

1535.ENSP00000262643->ENSP00000266970->ENSP00000345571 3

1536.ENSP00000262643->ENSP00000228872->ENSP00000270202->ENSP00000299421->ENSP00000228307->ENSP00000380227->ENSP00000346839 7

1537.ENSP00000262643->ENSP00000266970->ENSP00000269305->ENSP00000329623->ENSP00000311032->ENSP00000347858 6

1538.ENSP00000262643->ENSP00000228872->ENSP00000270202->ENSP00000299421->ENSP00000228307->ENSP00000380227->ENSP00000364094->ENSP00000386896->ENSP00000200181->ENSP00000340937->ENSP00000348384 17

1539.ENSP00000262643->ENSP00000281708->ENSP00000367207->ENSP00000351490 3

1540.ENSP00000262643->ENSP00000228872->ENSP00000270202->ENSP00000352121 8

1541.ENSP00000262643->ENSP00000228872->ENSP00000270202->ENSP00000206249->ENSP00000327213->ENSP00000352900 19

1542.ENSP00000262643->ENSP00000266970->ENSP00000269305->ENSP00000353059 4

1543.ENSP00000262643->ENSP00000228872->ENSP00000270202->ENSP00000299421->ENSP00000228307->ENSP00000380227->ENSP00000364094->ENSP00000282588->ENSP00000364979->ENSP00000353654 20

1544.ENSP00000262643->ENSP00000266970->ENSP00000255465->ENSP00000306043->ENSP00000342307->ENSP00000230538->ENSP00000354360 16

1545.ENSP00000262643->ENSP00000266970->ENSP00000267163->ENSP00000355249 3

1546.ENSP00000262643->ENSP00000266970->ENSP00000269305->ENSP00000344352->ENSP00000360266->ENSP00000356438 9

1547.ENSP00000262643->ENSP00000244741->ENSP00000344818->ENSP00000316840->ENSP00000358622 5

1548.ENSP00000262643->ENSP00000281708->ENSP00000231487->ENSP00000359206->ENSP00000216797->ENSP00000359424 5

1549.ENSP00000262643->ENSP00000266970->ENSP00000269305->ENSP00000361021 3

1550.ENSP00000262643->ENSP00000266970->ENSP00000255465->ENSP00000306043->ENSP00000256442->ENSP00000300093->ENSP00000263753->ENSP00000418447->ENSP00000348784->ENSP00000379866->ENSP00000379823->ENSP00000331902->ENSP00000361290 46

1551.ENSP00000262643->ENSP00000244741->ENSP00000344818->ENSP00000316840->ENSP00000361359->ENSP00000362994 6

1552.ENSP00000262643->ENSP00000228872->ENSP00000270202->ENSP00000206249->ENSP00000327213->ENSP00000363812 31

1553.ENSP00000262643->ENSP00000228872->ENSP00000270202->ENSP00000299421->ENSP00000228307->ENSP00000380227->ENSP00000364094 7

1554.ENSP00000262643->ENSP00000228872->ENSP00000270202->ENSP00000299421->ENSP00000228307->ENSP00000380227->ENSP00000364094->ENSP00000282588->ENSP00000364979 19

1555.ENSP00000262643->ENSP00000266970->ENSP00000269305->ENSP00000361021->ENSP00000366563 15

1556.ENSP00000262643->ENSP00000281708->ENSP00000367207 2

1557.ENSP00000262643->ENSP00000228872->ENSP00000270202->ENSP00000206249->ENSP00000254227->ENSP00000342470->ENSP00000363868->ENSP00000372703 124

1558.ENSP00000262643->ENSP00000266970->ENSP00000255465->ENSP00000306043->ENSP00000342307->ENSP00000230538->ENSP00000222399->ENSP00000374309 24

1559.ENSP00000262643->ENSP00000228872->ENSP00000270202->ENSP00000348986->ENSP00000375892 8

1560.ENSP00000262643->ENSP00000266970->ENSP00000227507->ENSP00000264657->ENSP00000376765 7

1561.ENSP00000262643->ENSP00000266970->ENSP00000255465->ENSP00000306043->ENSP00000256442->ENSP00000300093->ENSP00000263753->ENSP00000418447->ENSP00000348784->ENSP00000379866 43

1562.ENSP00000262643->ENSP00000266970->ENSP00000269305->ENSP00000324897->ENSP00000381648 4

1563.ENSP00000262643->ENSP00000281708->ENSP00000367207->ENSP00000263253->ENSP00000384273 4

1564.ENSP00000262643->ENSP00000228872->ENSP00000270202->ENSP00000299421->ENSP00000228307->ENSP00000380227->ENSP00000364094->ENSP00000386896 8

1565.ENSP00000262643->ENSP00000228872->ENSP00000270202->ENSP00000206249->ENSP00000254227->ENSP00000342470->ENSP00000363868->ENSP00000393286 124

1566.ENSP00000262643->ENSP00000228872->ENSP00000270202->ENSP00000206249->ENSP00000254227->ENSP00000342470->ENSP00000363868->ENSP00000393870 124

1567.ENSP00000262643->ENSP00000281708->ENSP00000367207->ENSP00000263253->ENSP00000262160->ENSP00000287727->ENSP00000400104 105

1568.ENSP00000262643->ENSP00000266970->ENSP00000255465->ENSP00000306043->ENSP00000342307->ENSP00000230538->ENSP00000354360->ENSP00000400365 17

1569.ENSP00000262643->ENSP00000228872->ENSP00000270202->ENSP00000206249->ENSP00000254227->ENSP00000342470->ENSP00000363868->ENSP00000402590 124

1570.ENSP00000262643->ENSP00000281708->ENSP00000367207->ENSP00000263253->ENSP00000325690->ENSP00000399968->ENSP00000419692 6

1571.ENSP00000262741->ENSP00000304895->ENSP00000353483->ENSP00000269305->ENSP00000267163->ENSP00000262904 7

1572.ENSP00000262741->ENSP00000304895->ENSP00000353483->ENSP00000360266->ENSP00000341551->ENSP00000262971 12

1573.ENSP00000262741->ENSP00000304895->ENSP00000353483->ENSP00000316840->ENSP00000361359->ENSP00000263464 10

1574.ENSP00000262741->ENSP00000304895->ENSP00000353483->ENSP00000269305->ENSP00000338018->ENSP00000361125->ENSP00000263826 27

1575.ENSP00000262741->ENSP00000304895->ENSP00000263967 4

1576.ENSP00000262741->ENSP00000304895->ENSP00000339007->ENSP00000341189->ENSP00000228307->ENSP00000380227->ENSP00000364094->ENSP00000386896->ENSP00000200181->ENSP00000340937->ENSP00000264144 24

1577.ENSP00000262741->ENSP00000304895->ENSP00000348986->ENSP00000270202->ENSP00000228872->ENSP00000265734 7

1578.ENSP00000262741->ENSP00000304895->ENSP00000353483->ENSP00000269305->ENSP00000266970 6

1579.ENSP00000262741->ENSP00000304895->ENSP00000353483->ENSP00000269305->ENSP00000267163 6

1580.ENSP00000262741->ENSP00000304895->ENSP00000348986->ENSP00000270202->ENSP00000352121->ENSP00000269300 14

1581.ENSP00000262741->ENSP00000304895->ENSP00000353483->ENSP00000269305 5

1582.ENSP00000262741->ENSP00000304895->ENSP00000348986->ENSP00000270202 5

1583.ENSP00000262741->ENSP00000304895->ENSP00000348986->ENSP00000270202->ENSP00000228872->ENSP00000274255 7

1584.ENSP00000262741->ENSP00000304895->ENSP00000274335 4

1585.ENSP00000262741->ENSP00000304895->ENSP00000348986->ENSP00000270202->ENSP00000228872->ENSP00000257904->ENSP00000276925 8

1586.ENSP00000262741->ENSP00000304895->ENSP00000274335->ENSP00000289153 8

1587.ENSP00000262741->ENSP00000304895->ENSP00000339007->ENSP00000341189->ENSP00000228307->ENSP00000380227->ENSP00000364094->ENSP00000296585 9

1588.ENSP00000262741->ENSP00000304895->ENSP00000353483->ENSP00000269305->ENSP00000302564 7

1589.ENSP00000262741->ENSP00000304895->ENSP00000348986->ENSP00000270202->ENSP00000348461->ENSP00000356505->ENSP00000220764->ENSP00000348380->ENSP00000305638 1023

1590.ENSP00000262741->ENSP00000304895->ENSP00000339007->ENSP00000312435->ENSP00000400365->ENSP00000354360->ENSP00000252999->ENSP00000307156 29

1591.ENSP00000262741->ENSP00000304895->ENSP00000353483->ENSP00000269305->ENSP00000353059->ENSP00000307786 13

1592.ENSP00000262741->ENSP00000304895->ENSP00000353483->ENSP00000269305->ENSP00000266970->ENSP00000309181 7

1593.ENSP00000262741->ENSP00000304895->ENSP00000353483->ENSP00000269305->ENSP00000266970->ENSP00000311083 7

1594.ENSP00000262741->ENSP00000304895->ENSP00000339007->ENSP00000341189->ENSP00000228307->ENSP00000380227->ENSP00000364094->ENSP00000386896->ENSP00000200181->ENSP00000340937->ENSP00000348384->ENSP00000324532 19

1595.ENSP00000262741->ENSP00000304895->ENSP00000353483->ENSP00000269305->ENSP00000262367->ENSP00000384273->ENSP00000226574->ENSP00000327251 27

1596.ENSP00000262741->ENSP00000304895->ENSP00000353483->ENSP00000269305->ENSP00000329623 6

1597.ENSP00000262741->ENSP00000304895->ENSP00000353483->ENSP00000269305->ENSP00000353059->ENSP00000330237 8

1598.ENSP00000262741->ENSP00000304895->ENSP00000268035->ENSP00000206249->ENSP00000399968->ENSP00000419692->ENSP00000332296 23

1599.ENSP00000262741->ENSP00000304895->ENSP00000353483->ENSP00000316840->ENSP00000361359->ENSP00000332468 9

1600.ENSP00000262741->ENSP00000304895->ENSP00000353483->ENSP00000269305->ENSP00000262367->ENSP00000384273->ENSP00000339151 8

1601.ENSP00000262741->ENSP00000304895->ENSP00000339007->ENSP00000341189 5

1602.ENSP00000262741->ENSP00000304895->ENSP00000353483->ENSP00000269305->ENSP00000293195->ENSP00000342087 39

1603.ENSP00000262741->ENSP00000304895->ENSP00000353483->ENSP00000269305->ENSP00000267163->ENSP00000345571 7

1604.ENSP00000262741->ENSP00000304895->ENSP00000339007->ENSP00000341189->ENSP00000228307->ENSP00000380227->ENSP00000346839 8

1605.ENSP00000262741->ENSP00000304895->ENSP00000353483->ENSP00000269305->ENSP00000329623->ENSP00000311032->ENSP00000347858 9

1606.ENSP00000262741->ENSP00000304895->ENSP00000339007->ENSP00000341189->ENSP00000228307->ENSP00000380227->ENSP00000364094->ENSP00000386896->ENSP00000200181->ENSP00000340937->ENSP00000348384 18

1607.ENSP00000262741->ENSP00000304895->ENSP00000268035->ENSP00000206249->ENSP00000367207->ENSP00000351490 8

1608.ENSP00000262741->ENSP00000304895->ENSP00000348986->ENSP00000270202->ENSP00000352121 11

1609.ENSP00000262741->ENSP00000304895->ENSP00000268035->ENSP00000206249->ENSP00000327213->ENSP00000352900 20

1610.ENSP00000262741->ENSP00000304895->ENSP00000353483->ENSP00000269305->ENSP00000353059 7

1611.ENSP00000262741->ENSP00000304895->ENSP00000339007->ENSP00000341189->ENSP00000228307->ENSP00000380227->ENSP00000364094->ENSP00000282588->ENSP00000364979->ENSP00000353654 21

1612.ENSP00000262741->ENSP00000304895->ENSP00000339007->ENSP00000312435->ENSP00000400365->ENSP00000354360 19

1613.ENSP00000262741->ENSP00000304895->ENSP00000353483->ENSP00000269305->ENSP00000267163->ENSP00000355249 7

1614.ENSP00000262741->ENSP00000304895->ENSP00000353483->ENSP00000360266->ENSP00000356438 10

1615.ENSP00000262741->ENSP00000304895->ENSP00000353483->ENSP00000316840->ENSP00000358622 8

1616.ENSP00000262741->ENSP00000304895->ENSP00000353483->ENSP00000269305->ENSP00000262367->ENSP00000384273->ENSP00000359424 8

1617.ENSP00000262741->ENSP00000304895->ENSP00000263967->ENSP00000361021 6

1618.ENSP00000262741->ENSP00000304895->ENSP00000353483->ENSP00000269305->ENSP00000278916->ENSP00000312995->ENSP00000300093->ENSP00000263753->ENSP00000418447->ENSP00000348784->ENSP00000379866->ENSP00000379823->ENSP00000331902->ENSP00000361290 49

1619.ENSP00000262741->ENSP00000304895->ENSP00000353483->ENSP00000316840->ENSP00000361359->ENSP00000362994 9

1620.ENSP00000262741->ENSP00000304895->ENSP00000268035->ENSP00000206249->ENSP00000327213->ENSP00000363812 32

1621.ENSP00000262741->ENSP00000304895->ENSP00000339007->ENSP00000341189->ENSP00000228307->ENSP00000380227->ENSP00000364094 8

1622.ENSP00000262741->ENSP00000304895->ENSP00000339007->ENSP00000341189->ENSP00000228307->ENSP00000380227->ENSP00000364094->ENSP00000282588->ENSP00000364979 20

1623.ENSP00000262741->ENSP00000304895->ENSP00000263967->ENSP00000361021->ENSP00000366563 18

1624.ENSP00000262741->ENSP00000304895->ENSP00000268035->ENSP00000206249->ENSP00000367207 7

1625.ENSP00000262741->ENSP00000304895->ENSP00000268035->ENSP00000206249->ENSP00000254227->ENSP00000342470->ENSP00000363868->ENSP00000372703 125

1626.ENSP00000262741->ENSP00000304895->ENSP00000339007->ENSP00000312435->ENSP00000400365->ENSP00000258341->ENSP00000374309 28

1627.ENSP00000262741->ENSP00000304895->ENSP00000348986->ENSP00000375892 9

1628.ENSP00000262741->ENSP00000304895->ENSP00000343204->ENSP00000264657->ENSP00000376765 8

1629.ENSP00000262741->ENSP00000304895->ENSP00000353483->ENSP00000269305->ENSP00000278916->ENSP00000312995->ENSP00000300093->ENSP00000263753->ENSP00000418447->ENSP00000348784->ENSP00000379866 46

1630.ENSP00000262741->ENSP00000304895->ENSP00000353483->ENSP00000269305->ENSP00000324897->ENSP00000381648 7

1631.ENSP00000262741->ENSP00000304895->ENSP00000353483->ENSP00000269305->ENSP00000262367->ENSP00000384273 7

1632.ENSP00000262741->ENSP00000304895->ENSP00000339007->ENSP00000341189->ENSP00000228307->ENSP00000380227->ENSP00000364094->ENSP00000386896 9

1633.ENSP00000262741->ENSP00000304895->ENSP00000268035->ENSP00000206249->ENSP00000254227->ENSP00000342470->ENSP00000363868->ENSP00000393286 125

1634.ENSP00000262741->ENSP00000304895->ENSP00000268035->ENSP00000206249->ENSP00000254227->ENSP00000342470->ENSP00000363868->ENSP00000393870 125

1635.ENSP00000262741->ENSP00000304895->ENSP00000353483->ENSP00000269305->ENSP00000263253->ENSP00000262160->ENSP00000287727->ENSP00000400104 108

1636.ENSP00000262741->ENSP00000304895->ENSP00000339007->ENSP00000312435->ENSP00000400365 18

1637.ENSP00000262741->ENSP00000304895->ENSP00000268035->ENSP00000206249->ENSP00000254227->ENSP00000342470->ENSP00000363868->ENSP00000402590 125

1638.ENSP00000262741->ENSP00000304895->ENSP00000268035->ENSP00000206249->ENSP00000399968->ENSP00000419692 7

1639.ENSP00000262904->ENSP00000267163->ENSP00000362649->ENSP00000329357->ENSP00000332973->ENSP00000341551->ENSP00000262971 9

1640.ENSP00000262904->ENSP00000267163->ENSP00000362649->ENSP00000384273->ENSP00000359424->ENSP00000247668->ENSP00000263464 6

1641.ENSP00000262904->ENSP00000267163->ENSP00000269305->ENSP00000338018->ENSP00000361125->ENSP00000263826 24

1642.ENSP00000262904->ENSP00000267163->ENSP00000417281->ENSP00000270202->ENSP00000263967 5

1643.ENSP00000262904->ENSP00000267163->ENSP00000269305->ENSP00000361021->ENSP00000341189->ENSP00000228307->ENSP00000380227->ENSP00000364094->ENSP00000386896->ENSP00000200181->ENSP00000340937->ENSP00000264144 24

1644.ENSP00000262904->ENSP00000267163->ENSP00000265734 3

1645.ENSP00000262904->ENSP00000267163->ENSP00000266970 2

1646.ENSP00000262904->ENSP00000267163 1

1647.ENSP00000262904->ENSP00000267163->ENSP00000417281->ENSP00000270202->ENSP00000352121->ENSP00000269300 12

1648.ENSP00000262904->ENSP00000267163->ENSP00000269305 2

1649.ENSP00000262904->ENSP00000267163->ENSP00000417281->ENSP00000270202 3

1650.ENSP00000262904->ENSP00000267163->ENSP00000266970->ENSP00000274255 4

1651.ENSP00000262904->ENSP00000267163->ENSP00000269305->ENSP00000353483->ENSP00000304895->ENSP00000274335 5

1652.ENSP00000262904->ENSP00000267163->ENSP00000257904->ENSP00000276925 3

1653.ENSP00000262904->ENSP00000267163->ENSP00000417281->ENSP00000270202->ENSP00000289153 7

1654.ENSP00000262904->ENSP00000267163->ENSP00000269305->ENSP00000361021->ENSP00000341189->ENSP00000228307->ENSP00000380227->ENSP00000364094->ENSP00000296585 9

1655.ENSP00000262904->ENSP00000267163->ENSP00000269305->ENSP00000302564 4

1656.ENSP00000262904->ENSP00000267163->ENSP00000417281->ENSP00000270202->ENSP00000348461->ENSP00000356505->ENSP00000220764->ENSP00000348380->ENSP00000305638 1021

1657.ENSP00000262904->ENSP00000267163->ENSP00000266970->ENSP00000255465->ENSP00000306043->ENSP00000342307->ENSP00000230538->ENSP00000354360->ENSP00000252999->ENSP00000307156 27

1658.ENSP00000262904->ENSP00000267163->ENSP00000269305->ENSP00000353059->ENSP00000307786 10

1659.ENSP00000262904->ENSP00000267163->ENSP00000266970->ENSP00000309181 3

1660.ENSP00000262904->ENSP00000267163->ENSP00000266970->ENSP00000311083 3

1661.ENSP00000262904->ENSP00000267163->ENSP00000269305->ENSP00000361021->ENSP00000341189->ENSP00000228307->ENSP00000380227->ENSP00000364094->ENSP00000386896->ENSP00000200181->ENSP00000340937->ENSP00000348384->ENSP00000324532 19

1662.ENSP00000262904->ENSP00000267163->ENSP00000362649->ENSP00000384273->ENSP00000226574->ENSP00000327251 23

1663.ENSP00000262904->ENSP00000267163->ENSP00000269305->ENSP00000329623 3

1664.ENSP00000262904->ENSP00000267163->ENSP00000269305->ENSP00000353059->ENSP00000330237 5

1665.ENSP00000262904->ENSP00000267163->ENSP00000350720->ENSP00000231509->ENSP00000320940->ENSP00000348827->ENSP00000419692->ENSP00000332296 22

1666.ENSP00000262904->ENSP00000267163->ENSP00000417281->ENSP00000344818->ENSP00000316840->ENSP00000361359->ENSP00000332468 6

1667.ENSP00000262904->ENSP00000267163->ENSP00000362649->ENSP00000384273->ENSP00000339151 4

1668.ENSP00000262904->ENSP00000267163->ENSP00000269305->ENSP00000361021->ENSP00000341189 5

1669.ENSP00000262904->ENSP00000267163->ENSP00000269305->ENSP00000293195->ENSP00000342087 36

1670.ENSP00000262904->ENSP00000267163->ENSP00000345571 2

1671.ENSP00000262904->ENSP00000267163->ENSP00000269305->ENSP00000263253->ENSP00000262160->ENSP00000221930->ENSP00000346839 8

1672.ENSP00000262904->ENSP00000267163->ENSP00000269305->ENSP00000329623->ENSP00000311032->ENSP00000347858 6

1673.ENSP00000262904->ENSP00000267163->ENSP00000269305->ENSP00000361021->ENSP00000341189->ENSP00000228307->ENSP00000380227->ENSP00000364094->ENSP00000386896->ENSP00000200181->ENSP00000340937->ENSP00000348384 18

1674.ENSP00000262904->ENSP00000267163->ENSP00000367207->ENSP00000351490 4

1675.ENSP00000262904->ENSP00000267163->ENSP00000417281->ENSP00000270202->ENSP00000352121 9

1676.ENSP00000262904->ENSP00000267163->ENSP00000227507->ENSP00000206249->ENSP00000327213->ENSP00000352900 19

1677.ENSP00000262904->ENSP00000267163->ENSP00000269305->ENSP00000353059 4

1678.ENSP00000262904->ENSP00000267163->ENSP00000269305->ENSP00000361021->ENSP00000341189->ENSP00000228307->ENSP00000380227->ENSP00000364094->ENSP00000282588->ENSP00000364979->ENSP00000353654 21

1679.ENSP00000262904->ENSP00000267163->ENSP00000266970->ENSP00000255465->ENSP00000306043->ENSP00000342307->ENSP00000230538->ENSP00000354360 17

1680.ENSP00000262904->ENSP00000267163->ENSP00000355249 2

1681.ENSP00000262904->ENSP00000267163->ENSP00000269305->ENSP00000344352->ENSP00000360266->ENSP00000356438 9

1682.ENSP00000262904->ENSP00000267163->ENSP00000362649->ENSP00000384273->ENSP00000359424->ENSP00000358622 5

1683.ENSP00000262904->ENSP00000267163->ENSP00000362649->ENSP00000384273->ENSP00000359424 4

1684.ENSP00000262904->ENSP00000267163->ENSP00000269305->ENSP00000361021 3

1685.ENSP00000262904->ENSP00000267163->ENSP00000269305->ENSP00000278916->ENSP00000312995->ENSP00000300093->ENSP00000263753->ENSP00000418447->ENSP00000348784->ENSP00000379866->ENSP00000379823->ENSP00000331902->ENSP00000361290 46

1686.ENSP00000262904->ENSP00000267163->ENSP00000417281->ENSP00000344818->ENSP00000316840->ENSP00000361359->ENSP00000362994 6

1687.ENSP00000262904->ENSP00000267163->ENSP00000227507->ENSP00000206249->ENSP00000327213->ENSP00000363812 31

1688.ENSP00000262904->ENSP00000267163->ENSP00000269305->ENSP00000361021->ENSP00000341189->ENSP00000228307->ENSP00000380227->ENSP00000364094 8

1689.ENSP00000262904->ENSP00000267163->ENSP00000269305->ENSP00000361021->ENSP00000341189->ENSP00000228307->ENSP00000380227->ENSP00000364094->ENSP00000282588->ENSP00000364979 20

1690.ENSP00000262904->ENSP00000267163->ENSP00000269305->ENSP00000361021->ENSP00000366563 15

1691.ENSP00000262904->ENSP00000267163->ENSP00000367207 3

1692.ENSP00000262904->ENSP00000267163->ENSP00000227507->ENSP00000206249->ENSP00000254227->ENSP00000342470->ENSP00000363868->ENSP00000372703 124

1693.ENSP00000262904->ENSP00000267163->ENSP00000266970->ENSP00000255465->ENSP00000306043->ENSP00000342307->ENSP00000230538->ENSP00000222399->ENSP00000374309 25

1694.ENSP00000262904->ENSP00000267163->ENSP00000417281->ENSP00000270202->ENSP00000348986->ENSP00000375892 9

1695.ENSP00000262904->ENSP00000267163->ENSP00000227507->ENSP00000264657->ENSP00000376765 7

1696.ENSP00000262904->ENSP00000267163->ENSP00000269305->ENSP00000278916->ENSP00000312995->ENSP00000300093->ENSP00000263753->ENSP00000418447->ENSP00000348784->ENSP00000379866 43

1697.ENSP00000262904->ENSP00000267163->ENSP00000269305->ENSP00000324897->ENSP00000381648 4

1698.ENSP00000262904->ENSP00000267163->ENSP00000362649->ENSP00000384273 3

1699.ENSP00000262904->ENSP00000267163->ENSP00000269305->ENSP00000361021->ENSP00000341189->ENSP00000228307->ENSP00000380227->ENSP00000364094->ENSP00000386896 9

1700.ENSP00000262904->ENSP00000267163->ENSP00000227507->ENSP00000206249->ENSP00000254227->ENSP00000342470->ENSP00000363868->ENSP00000393286 124

1701.ENSP00000262904->ENSP00000267163->ENSP00000227507->ENSP00000206249->ENSP00000254227->ENSP00000342470->ENSP00000363868->ENSP00000393870 124

1702.ENSP00000262904->ENSP00000267163->ENSP00000362649->ENSP00000329357->ENSP00000332973->ENSP00000287727->ENSP00000400104 105

1703.ENSP00000262904->ENSP00000267163->ENSP00000417281->ENSP00000270202->ENSP00000297494->ENSP00000341940->ENSP00000312435->ENSP00000400365 18

1704.ENSP00000262904->ENSP00000267163->ENSP00000227507->ENSP00000206249->ENSP00000254227->ENSP00000342470->ENSP00000363868->ENSP00000402590 124

1705.ENSP00000262904->ENSP00000267163->ENSP00000350720->ENSP00000231509->ENSP00000320940->ENSP00000348827->ENSP00000419692 6

1706.ENSP00000262971->ENSP00000341551->ENSP00000262160->ENSP00000263253->ENSP00000384273->ENSP00000359424->ENSP00000247668->ENSP00000263464 10

1707.ENSP00000262971->ENSP00000341551->ENSP00000262160->ENSP00000263253->ENSP00000338018->ENSP00000361125->ENSP00000263826 28

1708.ENSP00000262971->ENSP00000341551->ENSP00000262160->ENSP00000263253->ENSP00000269305->ENSP00000361021->ENSP00000263967 10

1709.ENSP00000262971->ENSP00000341551->ENSP00000262160->ENSP00000221930->ENSP00000346839->ENSP00000364094->ENSP00000386896->ENSP00000200181->ENSP00000340937->ENSP00000264144 26

1710.ENSP00000262971->ENSP00000341551->ENSP00000262160->ENSP00000263253->ENSP00000269305->ENSP00000355153->ENSP00000265734 9

1711.ENSP00000262971->ENSP00000341551->ENSP00000262160->ENSP00000263253->ENSP00000269305->ENSP00000266970 8

1712.ENSP00000262971->ENSP00000341551->ENSP00000262160->ENSP00000263253->ENSP00000269305->ENSP00000267163 8

1713.ENSP00000262971->ENSP00000341551->ENSP00000262160->ENSP00000263253->ENSP00000206249->ENSP00000270202->ENSP00000352121->ENSP00000269300 18

1714.ENSP00000262971->ENSP00000341551->ENSP00000262160->ENSP00000263253->ENSP00000269305 7

1715.ENSP00000262971->ENSP00000341551->ENSP00000262160->ENSP00000263253->ENSP00000206249->ENSP00000270202 9

1716.ENSP00000262971->ENSP00000341551->ENSP00000262160->ENSP00000263253->ENSP00000367207->ENSP00000274255 9

1717.ENSP00000262971->ENSP00000341551->ENSP00000262160->ENSP00000263253->ENSP00000206249->ENSP00000268035->ENSP00000274335 10

1718.ENSP00000262971->ENSP00000341551->ENSP00000262160->ENSP00000263253->ENSP00000367207->ENSP00000257904->ENSP00000276925 10

1719.ENSP00000262971->ENSP00000341551->ENSP00000262160->ENSP00000263253->ENSP00000206249->ENSP00000270202->ENSP00000289153 13

1720.ENSP00000262971->ENSP00000341551->ENSP00000262160->ENSP00000221930->ENSP00000346839->ENSP00000364094->ENSP00000296585 11

1721.ENSP00000262971->ENSP00000341551->ENSP00000262160->ENSP00000263253->ENSP00000269305->ENSP00000302564 9

1722.ENSP00000262971->ENSP00000341551->ENSP00000262160->ENSP00000263253->ENSP00000264657->ENSP00000348461->ENSP00000356505->ENSP00000220764->ENSP00000348380->ENSP00000305638 1026

1723.ENSP00000262971->ENSP00000341551->ENSP00000332973->ENSP00000329357->ENSP00000345571->ENSP00000274026->ENSP00000306043->ENSP00000342307->ENSP00000230538->ENSP00000354360->ENSP00000252999->ENSP00000307156 32

1724.ENSP00000262971->ENSP00000341551->ENSP00000262160->ENSP00000263253->ENSP00000269305->ENSP00000353059->ENSP00000307786 15

1725.ENSP00000262971->ENSP00000341551->ENSP00000262160->ENSP00000263253->ENSP00000269305->ENSP00000266970->ENSP00000309181 9

1726.ENSP00000262971->ENSP00000341551->ENSP00000262160->ENSP00000263253->ENSP00000269305->ENSP00000266970->ENSP00000311083 9

1727.ENSP00000262971->ENSP00000341551->ENSP00000262160->ENSP00000221930->ENSP00000346839->ENSP00000364094->ENSP00000386896->ENSP00000200181->ENSP00000340937->ENSP00000348384->ENSP00000324532 21

1728.ENSP00000262971->ENSP00000341551->ENSP00000262160->ENSP00000263253->ENSP00000384273->ENSP00000226574->ENSP00000327251 27

1729.ENSP00000262971->ENSP00000341551->ENSP00000262160->ENSP00000263253->ENSP00000269305->ENSP00000329623 8

1730.ENSP00000262971->ENSP00000341551->ENSP00000262160->ENSP00000263253->ENSP00000269305->ENSP00000353059->ENSP00000330237 10

1731.ENSP00000262971->ENSP00000341551->ENSP00000262160->ENSP00000263253->ENSP00000206249->ENSP00000399968->ENSP00000419692->ENSP00000332296 25

1732.ENSP00000262971->ENSP00000341551->ENSP00000262160->ENSP00000263253->ENSP00000269305->ENSP00000344818->ENSP00000316840->ENSP00000361359->ENSP00000332468 11

1733.ENSP00000262971->ENSP00000341551->ENSP00000262160->ENSP00000263253->ENSP00000384273->ENSP00000339151 8

1734.ENSP00000262971->ENSP00000341551->ENSP00000262160->ENSP00000263253->ENSP00000206249->ENSP00000350941->ENSP00000341189 9

1735.ENSP00000262971->ENSP00000341551->ENSP00000262160->ENSP00000263253->ENSP00000269305->ENSP00000293195->ENSP00000342087 41

1736.ENSP00000262971->ENSP00000341551->ENSP00000332973->ENSP00000329357->ENSP00000345571 7

1737.ENSP00000262971->ENSP00000341551->ENSP00000262160->ENSP00000221930->ENSP00000346839 9

1738.ENSP00000262971->ENSP00000341551->ENSP00000262160->ENSP00000263253->ENSP00000269305->ENSP00000329623->ENSP00000311032->ENSP00000347858 11

1739.ENSP00000262971->ENSP00000341551->ENSP00000262160->ENSP00000221930->ENSP00000346839->ENSP00000364094->ENSP00000386896->ENSP00000200181->ENSP00000340937->ENSP00000348384 20

1740.ENSP00000262971->ENSP00000341551->ENSP00000262160->ENSP00000263253->ENSP00000367207->ENSP00000351490 8

1741.ENSP00000262971->ENSP00000341551->ENSP00000262160->ENSP00000263253->ENSP00000206249->ENSP00000270202->ENSP00000352121 15

1742.ENSP00000262971->ENSP00000341551->ENSP00000262160->ENSP00000263253->ENSP00000206249->ENSP00000327213->ENSP00000352900 22

1743.ENSP00000262971->ENSP00000341551->ENSP00000262160->ENSP00000263253->ENSP00000269305->ENSP00000353059 9

1744.ENSP00000262971->ENSP00000341551->ENSP00000262160->ENSP00000221930->ENSP00000346839->ENSP00000364094->ENSP00000282588->ENSP00000364979->ENSP00000353654 23

1745.ENSP00000262971->ENSP00000341551->ENSP00000332973->ENSP00000329357->ENSP00000345571->ENSP00000274026->ENSP00000306043->ENSP00000342307->ENSP00000230538->ENSP00000354360 22

1746.ENSP00000262971->ENSP00000341551->ENSP00000262160->ENSP00000263253->ENSP00000269305->ENSP00000267163->ENSP00000355249 9

1747.ENSP00000262971->ENSP00000341551->ENSP00000360266->ENSP00000356438 12

1748.ENSP00000262971->ENSP00000341551->ENSP00000262160->ENSP00000263253->ENSP00000384273->ENSP00000216797->ENSP00000358622 9

1749.ENSP00000262971->ENSP00000341551->ENSP00000262160->ENSP00000263253->ENSP00000384273->ENSP00000359424 8

1750.ENSP00000262971->ENSP00000341551->ENSP00000262160->ENSP00000263253->ENSP00000269305->ENSP00000361021 8

1751.ENSP00000262971->ENSP00000341551->ENSP00000262160->ENSP00000263253->ENSP00000269305->ENSP00000278916->ENSP00000321656->ENSP00000300093->ENSP00000263753->ENSP00000418447->ENSP00000348784->ENSP00000379866->ENSP00000379823->ENSP00000331902->ENSP00000361290 51

1752.ENSP00000262971->ENSP00000341551->ENSP00000262160->ENSP00000263253->ENSP00000384273->ENSP00000226574->ENSP00000362994 11

1753.ENSP00000262971->ENSP00000341551->ENSP00000262160->ENSP00000263253->ENSP00000206249->ENSP00000327213->ENSP00000363812 34

1754.ENSP00000262971->ENSP00000341551->ENSP00000262160->ENSP00000221930->ENSP00000346839->ENSP00000364094 10

1755.ENSP00000262971->ENSP00000341551->ENSP00000262160->ENSP00000221930->ENSP00000346839->ENSP00000364094->ENSP00000282588->ENSP00000364979 22

1756.ENSP00000262971->ENSP00000341551->ENSP00000262160->ENSP00000263253->ENSP00000269305->ENSP00000361021->ENSP00000366563 20

1757.ENSP00000262971->ENSP00000341551->ENSP00000262160->ENSP00000263253->ENSP00000367207 7

1758.ENSP00000262971->ENSP00000341551->ENSP00000262160->ENSP00000263253->ENSP00000206249->ENSP00000399968->ENSP00000419692->ENSP00000342470->ENSP00000363868->ENSP00000372703 127

1759.ENSP00000262971->ENSP00000341551->ENSP00000332973->ENSP00000329357->ENSP00000345571->ENSP00000274026->ENSP00000306043->ENSP00000342307->ENSP00000230538->ENSP00000222399->ENSP00000374309 30

1760.ENSP00000262971->ENSP00000341551->ENSP00000360266->ENSP00000353483->ENSP00000304895->ENSP00000348986->ENSP00000375892 15

1761.ENSP00000262971->ENSP00000341551->ENSP00000262160->ENSP00000263253->ENSP00000264657->ENSP00000376765 10

1762.ENSP00000262971->ENSP00000341551->ENSP00000262160->ENSP00000263253->ENSP00000269305->ENSP00000278916->ENSP00000321656->ENSP00000300093->ENSP00000263753->ENSP00000418447->ENSP00000348784->ENSP00000379866 48

1763.ENSP00000262971->ENSP00000341551->ENSP00000262160->ENSP00000263253->ENSP00000269305->ENSP00000324897->ENSP00000381648 9

1764.ENSP00000262971->ENSP00000341551->ENSP00000262160->ENSP00000263253->ENSP00000384273 7

1765.ENSP00000262971->ENSP00000341551->ENSP00000262160->ENSP00000221930->ENSP00000346839->ENSP00000364094->ENSP00000386896 11

1766.ENSP00000262971->ENSP00000341551->ENSP00000262160->ENSP00000263253->ENSP00000206249->ENSP00000399968->ENSP00000419692->ENSP00000342470->ENSP00000363868->ENSP00000393286 127

1767.ENSP00000262971->ENSP00000341551->ENSP00000262160->ENSP00000263253->ENSP00000206249->ENSP00000399968->ENSP00000419692->ENSP00000342470->ENSP00000363868->ENSP00000393870 127

1768.ENSP00000262971->ENSP00000341551->ENSP00000332973->ENSP00000287727->ENSP00000400104 106

1769.ENSP00000262971->ENSP00000341551->ENSP00000332973->ENSP00000329357->ENSP00000345571->ENSP00000274026->ENSP00000306043->ENSP00000342307->ENSP00000230538->ENSP00000354360->ENSP00000400365 23

1770.ENSP00000262971->ENSP00000341551->ENSP00000262160->ENSP00000263253->ENSP00000206249->ENSP00000399968->ENSP00000419692->ENSP00000342470->ENSP00000363868->ENSP00000402590 127

1771.ENSP00000262971->ENSP00000341551->ENSP00000262160->ENSP00000263253->ENSP00000206249->ENSP00000399968->ENSP00000419692 9

1772.ENSP00000263464->ENSP00000247668->ENSP00000259808->ENSP00000344818->ENSP00000338018->ENSP00000361125->ENSP00000263826 26

1773.ENSP00000263464->ENSP00000247668->ENSP00000259808->ENSP00000344818->ENSP00000275493->ENSP00000263967 8

1774.ENSP00000263464->ENSP00000247668->ENSP00000259808->ENSP00000344818->ENSP00000275493->ENSP00000300574->ENSP00000228307->ENSP00000380227->ENSP00000364094->ENSP00000386896->ENSP00000200181->ENSP00000340937->ENSP00000264144 26

1775.ENSP00000263464->ENSP00000247668->ENSP00000359424->ENSP00000384273->ENSP00000362649->ENSP00000267163->ENSP00000265734 7

1776.ENSP00000263464->ENSP00000247668->ENSP00000359424->ENSP00000384273->ENSP00000362649->ENSP00000269305->ENSP00000266970 6

1777.ENSP00000263464->ENSP00000247668->ENSP00000359424->ENSP00000384273->ENSP00000362649->ENSP00000267163 5

1778.ENSP00000263464->ENSP00000247668->ENSP00000259808->ENSP00000344818->ENSP00000417281->ENSP00000270202->ENSP00000352121->ENSP00000269300 15

1779.ENSP00000263464->ENSP00000247668->ENSP00000359424->ENSP00000384273->ENSP00000362649->ENSP00000269305 5

1780.ENSP00000263464->ENSP00000247668->ENSP00000259808->ENSP00000344818->ENSP00000417281->ENSP00000270202 6

1781.ENSP00000263464->ENSP00000247668->ENSP00000359424->ENSP00000216797->ENSP00000359206->ENSP00000326804->ENSP00000274255 6

1782.ENSP00000263464->ENSP00000247668->ENSP00000259808->ENSP00000344818->ENSP00000275493->ENSP00000264033->ENSP00000274335 7

1783.ENSP00000263464->ENSP00000247668->ENSP00000359424->ENSP00000384273->ENSP00000362649->ENSP00000267163->ENSP00000257904->ENSP00000276925 7

1784.ENSP00000263464->ENSP00000247668->ENSP00000259808->ENSP00000344818->ENSP00000417281->ENSP00000270202->ENSP00000289153 10

1785.ENSP00000263464->ENSP00000247668->ENSP00000259808->ENSP00000344818->ENSP00000275493->ENSP00000300574->ENSP00000228307->ENSP00000380227->ENSP00000364094->ENSP00000296585 11

1786.ENSP00000263464->ENSP00000267169->ENSP00000347858->ENSP00000311032->ENSP00000350311->ENSP00000302564 6

1787.ENSP00000263464->ENSP00000247668->ENSP00000359424->ENSP00000384273->ENSP00000263253->ENSP00000264657->ENSP00000348461->ENSP00000356505->ENSP00000220764->ENSP00000348380->ENSP00000305638 1024

1788.ENSP00000263464->ENSP00000267169->ENSP00000301633->ENSP00000342307->ENSP00000230538->ENSP00000354360->ENSP00000252999->ENSP00000307156 30

1789.ENSP00000263464->ENSP00000267169->ENSP00000347858->ENSP00000330237->ENSP00000353059->ENSP00000307786 10

1790.ENSP00000263464->ENSP00000247668->ENSP00000359424->ENSP00000384273->ENSP00000362649->ENSP00000269305->ENSP00000266970->ENSP00000309181 7

1791.ENSP00000263464->ENSP00000247668->ENSP00000359424->ENSP00000216797->ENSP00000359206->ENSP00000326804->ENSP00000274255->ENSP00000311083 7

1792.ENSP00000263464->ENSP00000247668->ENSP00000259808->ENSP00000344818->ENSP00000275493->ENSP00000300574->ENSP00000228307->ENSP00000380227->ENSP00000364094->ENSP00000386896->ENSP00000200181->ENSP00000340937->ENSP00000348384->ENSP00000324532 21

1793.ENSP00000263464->ENSP00000247668->ENSP00000359424->ENSP00000226574->ENSP00000327251 22

1794.ENSP00000263464->ENSP00000247668->ENSP00000259808->ENSP00000351273->ENSP00000329623 5

1795.ENSP00000263464->ENSP00000267169->ENSP00000347858->ENSP00000330237 3

1796.ENSP00000263464->ENSP00000247668->ENSP00000359424->ENSP00000384273->ENSP00000263253->ENSP00000206249->ENSP00000399968->ENSP00000419692->ENSP00000332296 23

1797.ENSP00000263464->ENSP00000332468 2

1798.ENSP00000263464->ENSP00000247668->ENSP00000339151 3

1799.ENSP00000263464->ENSP00000247668->ENSP00000259808->ENSP00000344818->ENSP00000275493->ENSP00000339007->ENSP00000341189 7

1800.ENSP00000263464->ENSP00000247668->ENSP00000359424->ENSP00000384273->ENSP00000362649->ENSP00000269305->ENSP00000293195->ENSP00000342087 39

1801.ENSP00000263464->ENSP00000247668->ENSP00000359424->ENSP00000384273->ENSP00000362649->ENSP00000267163->ENSP00000345571 6

1802.ENSP00000263464->ENSP00000247668->ENSP00000359424->ENSP00000384273->ENSP00000263253->ENSP00000262160->ENSP00000221930->ENSP00000346839 9

1803.ENSP00000263464->ENSP00000267169->ENSP00000347858 2

1804.ENSP00000263464->ENSP00000247668->ENSP00000259808->ENSP00000344818->ENSP00000275493->ENSP00000300574->ENSP00000228307->ENSP00000380227->ENSP00000364094->ENSP00000386896->ENSP00000200181->ENSP00000340937->ENSP00000348384 20

1805.ENSP00000263464->ENSP00000247668->ENSP00000359424->ENSP00000384273->ENSP00000263253->ENSP00000367207->ENSP00000351490 6

1806.ENSP00000263464->ENSP00000247668->ENSP00000259808->ENSP00000344818->ENSP00000417281->ENSP00000270202->ENSP00000352121 12

1807.ENSP00000263464->ENSP00000247668->ENSP00000359424->ENSP00000384273->ENSP00000263253->ENSP00000206249->ENSP00000327213->ENSP00000352900 20

1808.ENSP00000263464->ENSP00000267169->ENSP00000347858->ENSP00000330237->ENSP00000353059 4

1809.ENSP00000263464->ENSP00000247668->ENSP00000259808->ENSP00000344818->ENSP00000275493->ENSP00000300574->ENSP00000228307->ENSP00000380227->ENSP00000364094->ENSP00000282588->ENSP00000364979->ENSP00000353654 23

1810.ENSP00000263464->ENSP00000267169->ENSP00000301633->ENSP00000342307->ENSP00000230538->ENSP00000354360 20

1811.ENSP00000263464->ENSP00000247668->ENSP00000359424->ENSP00000384273->ENSP00000362649->ENSP00000267163->ENSP00000355249 6

1812.ENSP00000263464->ENSP00000247668->ENSP00000269485->ENSP00000316840->ENSP00000358997->ENSP00000263341->ENSP00000356438 8

1813.ENSP00000263464->ENSP00000247668->ENSP00000259808->ENSP00000358622 3

1814.ENSP00000263464->ENSP00000247668->ENSP00000359424 2

1815.ENSP00000263464->ENSP00000247668->ENSP00000359424->ENSP00000384273->ENSP00000362649->ENSP00000269305->ENSP00000361021 6

1816.ENSP00000263464->ENSP00000247668->ENSP00000359424->ENSP00000384273->ENSP00000362649->ENSP00000269305->ENSP00000372023->ENSP00000321656->ENSP00000300093->ENSP00000263753->ENSP00000418447->ENSP00000348784->ENSP00000379866->ENSP00000379823->ENSP00000331902->ENSP00000361290 49

1817.ENSP00000263464->ENSP00000362994 1

1818.ENSP00000263464->ENSP00000247668->ENSP00000359424->ENSP00000384273->ENSP00000263253->ENSP00000206249->ENSP00000327213->ENSP00000363812 32

1819.ENSP00000263464->ENSP00000247668->ENSP00000259808->ENSP00000344818->ENSP00000275493->ENSP00000300574->ENSP00000228307->ENSP00000380227->ENSP00000364094 10

1820.ENSP00000263464->ENSP00000247668->ENSP00000259808->ENSP00000344818->ENSP00000275493->ENSP00000300574->ENSP00000228307->ENSP00000380227->ENSP00000364094->ENSP00000282588->ENSP00000364979 22

1821.ENSP00000263464->ENSP00000247668->ENSP00000359424->ENSP00000384273->ENSP00000362649->ENSP00000269305->ENSP00000361021->ENSP00000366563 18

1822.ENSP00000263464->ENSP00000247668->ENSP00000359424->ENSP00000384273->ENSP00000263253->ENSP00000367207 5

1823.ENSP00000263464->ENSP00000247668->ENSP00000359424->ENSP00000384273->ENSP00000263253->ENSP00000206249->ENSP00000254227->ENSP00000342470->ENSP00000363868->ENSP00000372703 125

1824.ENSP00000263464->ENSP00000267169->ENSP00000301633->ENSP00000342307->ENSP00000230538->ENSP00000222399->ENSP00000374309 28

1825.ENSP00000263464->ENSP00000247668->ENSP00000259808->ENSP00000344818->ENSP00000417281->ENSP00000270202->ENSP00000348986->ENSP00000375892 12

1826.ENSP00000263464->ENSP00000247668->ENSP00000359424->ENSP00000384273->ENSP00000263253->ENSP00000264657->ENSP00000376765 8

1827.ENSP00000263464->ENSP00000247668->ENSP00000359424->ENSP00000384273->ENSP00000362649->ENSP00000269305->ENSP00000372023->ENSP00000321656->ENSP00000300093->ENSP00000263753->ENSP00000418447->ENSP00000348784->ENSP00000379866 46

1828.ENSP00000263464->ENSP00000247668->ENSP00000359424->ENSP00000384273->ENSP00000362649->ENSP00000269305->ENSP00000324897->ENSP00000381648 7

1829.ENSP00000263464->ENSP00000247668->ENSP00000359424->ENSP00000384273 3

1830.ENSP00000263464->ENSP00000247668->ENSP00000259808->ENSP00000344818->ENSP00000275493->ENSP00000300574->ENSP00000228307->ENSP00000380227->ENSP00000364094->ENSP00000386896 11

1831.ENSP00000263464->ENSP00000247668->ENSP00000359424->ENSP00000384273->ENSP00000263253->ENSP00000206249->ENSP00000254227->ENSP00000342470->ENSP00000363868->ENSP00000393286 125

1832.ENSP00000263464->ENSP00000247668->ENSP00000359424->ENSP00000384273->ENSP00000263253->ENSP00000206249->ENSP00000254227->ENSP00000342470->ENSP00000363868->ENSP00000393870 125

1833.ENSP00000263464->ENSP00000247668->ENSP00000359424->ENSP00000384273->ENSP00000263253->ENSP00000262160->ENSP00000287727->ENSP00000400104 106

1834.ENSP00000263464->ENSP00000247668->ENSP00000259808->ENSP00000344818->ENSP00000275493->ENSP00000339007->ENSP00000312435->ENSP00000400365 20

1835.ENSP00000263464->ENSP00000247668->ENSP00000359424->ENSP00000384273->ENSP00000263253->ENSP00000206249->ENSP00000254227->ENSP00000342470->ENSP00000363868->ENSP00000402590 125

1836.ENSP00000263464->ENSP00000247668->ENSP00000359424->ENSP00000384273->ENSP00000263253->ENSP00000206249->ENSP00000399968->ENSP00000419692 7

1837.ENSP00000263826->ENSP00000361125->ENSP00000338018->ENSP00000269305->ENSP00000361021->ENSP00000263967 25

1838.ENSP00000263826->ENSP00000361125->ENSP00000261023->ENSP00000364094->ENSP00000386896->ENSP00000200181->ENSP00000340937->ENSP00000264144 42

1839.ENSP00000263826->ENSP00000361125->ENSP00000338018->ENSP00000269305->ENSP00000244741->ENSP00000265734 24

1840.ENSP00000263826->ENSP00000361125->ENSP00000338018->ENSP00000269305->ENSP00000266970 23

1841.ENSP00000263826->ENSP00000361125->ENSP00000338018->ENSP00000269305->ENSP00000267163 23

1842.ENSP00000263826->ENSP00000361125->ENSP00000270202->ENSP00000352121->ENSP00000269300 33

1843.ENSP00000263826->ENSP00000361125->ENSP00000338018->ENSP00000269305 22

1844.ENSP00000263826->ENSP00000361125->ENSP00000270202 24

1845.ENSP00000263826->ENSP00000361125->ENSP00000338018->ENSP00000269305->ENSP00000266970->ENSP00000274255 25

1846.ENSP00000263826->ENSP00000361125->ENSP00000263923->ENSP00000244007->ENSP00000261799->ENSP00000274335 24

1847.ENSP00000263826->ENSP00000361125->ENSP00000338018->ENSP00000269305->ENSP00000267163->ENSP00000257904->ENSP00000276925 25

1848.ENSP00000263826->ENSP00000361125->ENSP00000270202->ENSP00000289153 28

1849.ENSP00000263826->ENSP00000361125->ENSP00000261023->ENSP00000364094->ENSP00000296585 27

1850.ENSP00000263826->ENSP00000361125->ENSP00000338018->ENSP00000269305->ENSP00000302564 24

1851.ENSP00000263826->ENSP00000361125->ENSP00000338018->ENSP00000263253->ENSP00000264657->ENSP00000348461->ENSP00000356505->ENSP00000220764->ENSP00000348380->ENSP00000305638 1042

1852.ENSP00000263826->ENSP00000361125->ENSP00000338018->ENSP00000269305->ENSP00000278916->ENSP00000321656->ENSP00000256442->ENSP00000342307->ENSP00000230538->ENSP00000354360->ENSP00000252999->ENSP00000307156 47

1853.ENSP00000263826->ENSP00000361125->ENSP00000338018->ENSP00000269305->ENSP00000353059->ENSP00000307786 30

1854.ENSP00000263826->ENSP00000361125->ENSP00000338018->ENSP00000269305->ENSP00000266970->ENSP00000309181 24

1855.ENSP00000263826->ENSP00000361125->ENSP00000338018->ENSP00000269305->ENSP00000266970->ENSP00000311083 24

1856.ENSP00000263826->ENSP00000361125->ENSP00000261023->ENSP00000364094->ENSP00000386896->ENSP00000200181->ENSP00000340937->ENSP00000348384->ENSP00000324532 37

1857.ENSP00000263826->ENSP00000361125->ENSP00000338018->ENSP00000262367->ENSP00000384273->ENSP00000226574->ENSP00000327251 43

1858.ENSP00000263826->ENSP00000361125->ENSP00000338018->ENSP00000269305->ENSP00000329623 23

1859.ENSP00000263826->ENSP00000361125->ENSP00000338018->ENSP00000269305->ENSP00000353059->ENSP00000330237 25

1860.ENSP00000263826->ENSP00000361125->ENSP00000338018->ENSP00000263253->ENSP00000206249->ENSP00000399968->ENSP00000419692->ENSP00000332296 41

1861.ENSP00000263826->ENSP00000361125->ENSP00000338018->ENSP00000344818->ENSP00000316840->ENSP00000361359->ENSP00000332468 25

1862.ENSP00000263826->ENSP00000361125->ENSP00000338018->ENSP00000262367->ENSP00000384273->ENSP00000339151 24

1863.ENSP00000263826->ENSP00000361125->ENSP00000338018->ENSP00000269305->ENSP00000361021->ENSP00000341189 25

1864.ENSP00000263826->ENSP00000361125->ENSP00000338018->ENSP00000269305->ENSP00000293195->ENSP00000342087 56

1865.ENSP00000263826->ENSP00000361125->ENSP00000338018->ENSP00000329357->ENSP00000345571 24

1866.ENSP00000263826->ENSP00000361125->ENSP00000261023->ENSP00000346839 26

1867.ENSP00000263826->ENSP00000361125->ENSP00000338018->ENSP00000344818->ENSP00000316840->ENSP00000216160->ENSP00000347858 25

1868.ENSP00000263826->ENSP00000361125->ENSP00000261023->ENSP00000364094->ENSP00000386896->ENSP00000200181->ENSP00000340937->ENSP00000348384 36

1869.ENSP00000263826->ENSP00000361125->ENSP00000338018->ENSP00000263253->ENSP00000367207->ENSP00000351490 24

1870.ENSP00000263826->ENSP00000361125->ENSP00000270202->ENSP00000352121 30

1871.ENSP00000263826->ENSP00000361125->ENSP00000338018->ENSP00000263253->ENSP00000206249->ENSP00000327213->ENSP00000352900 38

1872.ENSP00000263826->ENSP00000361125->ENSP00000338018->ENSP00000269305->ENSP00000353059 24

1873.ENSP00000263826->ENSP00000361125->ENSP00000261023->ENSP00000364094->ENSP00000282588->ENSP00000364979->ENSP00000353654 39

1874.ENSP00000263826->ENSP00000361125->ENSP00000338018->ENSP00000269305->ENSP00000278916->ENSP00000321656->ENSP00000256442->ENSP00000342307->ENSP00000230538->ENSP00000354360 37

1875.ENSP00000263826->ENSP00000361125->ENSP00000338018->ENSP00000269305->ENSP00000267163->ENSP00000355249 24

1876.ENSP00000263826->ENSP00000361125->ENSP00000338018->ENSP00000263253->ENSP00000264657->ENSP00000258743->ENSP00000356438 28

1877.ENSP00000263826->ENSP00000361125->ENSP00000338018->ENSP00000344818->ENSP00000316840->ENSP00000358622 24

1878.ENSP00000263826->ENSP00000361125->ENSP00000338018->ENSP00000262367->ENSP00000384273->ENSP00000359424 24

1879.ENSP00000263826->ENSP00000361125->ENSP00000338018->ENSP00000269305->ENSP00000361021 23

1880.ENSP00000263826->ENSP00000361125->ENSP00000338018->ENSP00000269305->ENSP00000278916->ENSP00000321656->ENSP00000300093->ENSP00000263753->ENSP00000418447->ENSP00000348784->ENSP00000379866->ENSP00000379823->ENSP00000331902->ENSP00000361290 66

1881.ENSP00000263826->ENSP00000361125->ENSP00000338018->ENSP00000344818->ENSP00000316840->ENSP00000361359->ENSP00000362994 25

1882.ENSP00000263826->ENSP00000361125->ENSP00000338018->ENSP00000263253->ENSP00000206249->ENSP00000327213->ENSP00000363812 50

1883.ENSP00000263826->ENSP00000361125->ENSP00000261023->ENSP00000364094 26

1884.ENSP00000263826->ENSP00000361125->ENSP00000261023->ENSP00000364094->ENSP00000282588->ENSP00000364979 38

1885.ENSP00000263826->ENSP00000361125->ENSP00000338018->ENSP00000269305->ENSP00000361021->ENSP00000366563 35

1886.ENSP00000263826->ENSP00000361125->ENSP00000338018->ENSP00000263253->ENSP00000367207 23

1887.ENSP00000263826->ENSP00000361125->ENSP00000338018->ENSP00000263253->ENSP00000206249->ENSP00000254227->ENSP00000342470->ENSP00000363868->ENSP00000372703 143

1888.ENSP00000263826->ENSP00000361125->ENSP00000338018->ENSP00000269305->ENSP00000278916->ENSP00000321656->ENSP00000256442->ENSP00000342307->ENSP00000230538->ENSP00000222399->ENSP00000374309 45

1889.ENSP00000263826->ENSP00000361125->ENSP00000270202->ENSP00000348986->ENSP00000375892 30

1890.ENSP00000263826->ENSP00000361125->ENSP00000338018->ENSP00000263253->ENSP00000264657->ENSP00000376765 26

1891.ENSP00000263826->ENSP00000361125->ENSP00000338018->ENSP00000269305->ENSP00000278916->ENSP00000321656->ENSP00000300093->ENSP00000263753->ENSP00000418447->ENSP00000348784->ENSP00000379866 63

1892.ENSP00000263826->ENSP00000361125->ENSP00000338018->ENSP00000269305->ENSP00000324897->ENSP00000381648 24

1893.ENSP00000263826->ENSP00000361125->ENSP00000338018->ENSP00000262367->ENSP00000384273 23

1894.ENSP00000263826->ENSP00000361125->ENSP00000261023->ENSP00000364094->ENSP00000386896 27

1895.ENSP00000263826->ENSP00000361125->ENSP00000338018->ENSP00000263253->ENSP00000206249->ENSP00000254227->ENSP00000342470->ENSP00000363868->ENSP00000393286 143

1896.ENSP00000263826->ENSP00000361125->ENSP00000338018->ENSP00000263253->ENSP00000206249->ENSP00000254227->ENSP00000342470->ENSP00000363868->ENSP00000393870 143

1897.ENSP00000263826->ENSP00000361125->ENSP00000338018->ENSP00000263253->ENSP00000262160->ENSP00000287727->ENSP00000400104 124

1898.ENSP00000263826->ENSP00000361125->ENSP00000338018->ENSP00000269305->ENSP00000278916->ENSP00000321656->ENSP00000256442->ENSP00000342307->ENSP00000230538->ENSP00000354360->ENSP00000400365 38

1899.ENSP00000263826->ENSP00000361125->ENSP00000338018->ENSP00000263253->ENSP00000206249->ENSP00000254227->ENSP00000342470->ENSP00000363868->ENSP00000402590 143

1900.ENSP00000263826->ENSP00000361125->ENSP00000338018->ENSP00000263253->ENSP00000206249->ENSP00000399968->ENSP00000419692 25

1901.ENSP00000263967->ENSP00000304895->ENSP00000339007->ENSP00000341189->ENSP00000228307->ENSP00000380227->ENSP00000364094->ENSP00000386896->ENSP00000200181->ENSP00000340937->ENSP00000264144 22

1902.ENSP00000263967->ENSP00000270202->ENSP00000228872->ENSP00000265734 4

1903.ENSP00000263967->ENSP00000270202->ENSP00000228872->ENSP00000266970 4

1904.ENSP00000263967->ENSP00000270202->ENSP00000417281->ENSP00000267163 4

1905.ENSP00000263967->ENSP00000309845->ENSP00000352121->ENSP00000269300 11

1906.ENSP00000263967->ENSP00000304895->ENSP00000353483->ENSP00000269305 3

1907.ENSP00000263967->ENSP00000270202 2

1908.ENSP00000263967->ENSP00000270202->ENSP00000228872->ENSP00000274255 4

1909.ENSP00000263967->ENSP00000274335 1

1910.ENSP00000263967->ENSP00000270202->ENSP00000228872->ENSP00000257904->ENSP00000276925 5

1911.ENSP00000263967->ENSP00000274335->ENSP00000289153 5

1912.ENSP00000263967->ENSP00000304895->ENSP00000339007->ENSP00000341189->ENSP00000228307->ENSP00000380227->ENSP00000364094->ENSP00000296585 7

1913.ENSP00000263967->ENSP00000270202->ENSP00000309103->ENSP00000302564 4

1914.ENSP00000263967->ENSP00000270202->ENSP00000348461->ENSP00000356505->ENSP00000220764->ENSP00000348380->ENSP00000305638 1020

1915.ENSP00000263967->ENSP00000304895->ENSP00000339007->ENSP00000312435->ENSP00000400365->ENSP00000354360->ENSP00000252999->ENSP00000307156 27

1916.ENSP00000263967->ENSP00000304895->ENSP00000353483->ENSP00000269305->ENSP00000353059->ENSP00000307786 11

1917.ENSP00000263967->ENSP00000270202->ENSP00000228872->ENSP00000266970->ENSP00000309181 5

1918.ENSP00000263967->ENSP00000270202->ENSP00000228872->ENSP00000274255->ENSP00000311083 5

1919.ENSP00000263967->ENSP00000304895->ENSP00000339007->ENSP00000341189->ENSP00000228307->ENSP00000380227->ENSP00000364094->ENSP00000386896->ENSP00000200181->ENSP00000340937->ENSP00000348384->ENSP00000324532 17

1920.ENSP00000263967->ENSP00000309845->ENSP00000251849->ENSP00000335153->ENSP00000231509->ENSP00000226574->ENSP00000327251 25

1921.ENSP00000263967->ENSP00000270202->ENSP00000309103->ENSP00000329623 4

1922.ENSP00000263967->ENSP00000304895->ENSP00000353483->ENSP00000269305->ENSP00000353059->ENSP00000330237 6

1923.ENSP00000263967->ENSP00000304895->ENSP00000268035->ENSP00000206249->ENSP00000399968->ENSP00000419692->ENSP00000332296 21

1924.ENSP00000263967->ENSP00000304895->ENSP00000353483->ENSP00000316840->ENSP00000361359->ENSP00000332468 7

1925.ENSP00000263967->ENSP00000270202->ENSP00000298552->ENSP00000339151 6

1926.ENSP00000263967->ENSP00000304895->ENSP00000339007->ENSP00000341189 3

1927.ENSP00000263967->ENSP00000304895->ENSP00000353483->ENSP00000269305->ENSP00000293195->ENSP00000342087 37

1928.ENSP00000263967->ENSP00000270202->ENSP00000228872->ENSP00000274026->ENSP00000345571 5

1929.ENSP00000263967->ENSP00000304895->ENSP00000339007->ENSP00000341189->ENSP00000228307->ENSP00000380227->ENSP00000346839 6

1930.ENSP00000263967->ENSP00000270202->ENSP00000309103->ENSP00000329623->ENSP00000311032->ENSP00000347858 7

1931.ENSP00000263967->ENSP00000304895->ENSP00000339007->ENSP00000341189->ENSP00000228307->ENSP00000380227->ENSP00000364094->ENSP00000386896->ENSP00000200181->ENSP00000340937->ENSP00000348384 16

1932.ENSP00000263967->ENSP00000304895->ENSP00000339007->ENSP00000215832->ENSP00000367207->ENSP00000351490 6

1933.ENSP00000263967->ENSP00000309845->ENSP00000352121 8

1934.ENSP00000263967->ENSP00000304895->ENSP00000268035->ENSP00000206249->ENSP00000327213->ENSP00000352900 18

1935.ENSP00000263967->ENSP00000304895->ENSP00000353483->ENSP00000269305->ENSP00000353059 5

1936.ENSP00000263967->ENSP00000304895->ENSP00000339007->ENSP00000341189->ENSP00000228307->ENSP00000380227->ENSP00000364094->ENSP00000282588->ENSP00000364979->ENSP00000353654 19

1937.ENSP00000263967->ENSP00000304895->ENSP00000339007->ENSP00000312435->ENSP00000400365->ENSP00000354360 17

1938.ENSP00000263967->ENSP00000270202->ENSP00000417281->ENSP00000267163->ENSP00000355249 5

1939.ENSP00000263967->ENSP00000304895->ENSP00000353483->ENSP00000360266->ENSP00000356438 8

1940.ENSP00000263967->ENSP00000304895->ENSP00000353483->ENSP00000316840->ENSP00000358622 6

1941.ENSP00000263967->ENSP00000304895->ENSP00000343204->ENSP00000264657->ENSP00000263253->ENSP00000384273->ENSP00000359424 6

1942.ENSP00000263967->ENSP00000361021 2

1943.ENSP00000263967->ENSP00000304895->ENSP00000353483->ENSP00000269305->ENSP00000278916->ENSP00000321656->ENSP00000300093->ENSP00000263753->ENSP00000418447->ENSP00000348784->ENSP00000379866->ENSP00000379823->ENSP00000331902->ENSP00000361290 47

1944.ENSP00000263967->ENSP00000304895->ENSP00000353483->ENSP00000316840->ENSP00000361359->ENSP00000362994 7

1945.ENSP00000263967->ENSP00000304895->ENSP00000268035->ENSP00000206249->ENSP00000327213->ENSP00000363812 30

1946.ENSP00000263967->ENSP00000304895->ENSP00000339007->ENSP00000341189->ENSP00000228307->ENSP00000380227->ENSP00000364094 6

1947.ENSP00000263967->ENSP00000304895->ENSP00000339007->ENSP00000341189->ENSP00000228307->ENSP00000380227->ENSP00000364094->ENSP00000282588->ENSP00000364979 18

1948.ENSP00000263967->ENSP00000361021->ENSP00000366563 14

1949.ENSP00000263967->ENSP00000304895->ENSP00000339007->ENSP00000215832->ENSP00000367207 5

1950.ENSP00000263967->ENSP00000304895->ENSP00000268035->ENSP00000206249->ENSP00000254227->ENSP00000342470->ENSP00000363868->ENSP00000372703 123

1951.ENSP00000263967->ENSP00000304895->ENSP00000339007->ENSP00000312435->ENSP00000400365->ENSP00000258341->ENSP00000374309 26

1952.ENSP00000263967->ENSP00000304895->ENSP00000348986->ENSP00000375892 7

1953.ENSP00000263967->ENSP00000304895->ENSP00000343204->ENSP00000264657->ENSP00000376765 6

1954.ENSP00000263967->ENSP00000304895->ENSP00000353483->ENSP00000269305->ENSP00000278916->ENSP00000321656->ENSP00000300093->ENSP00000263753->ENSP00000418447->ENSP00000348784->ENSP00000379866 44

1955.ENSP00000263967->ENSP00000304895->ENSP00000353483->ENSP00000269305->ENSP00000324897->ENSP00000381648 5

1956.ENSP00000263967->ENSP00000304895->ENSP00000343204->ENSP00000264657->ENSP00000263253->ENSP00000384273 5

1957.ENSP00000263967->ENSP00000304895->ENSP00000339007->ENSP00000341189->ENSP00000228307->ENSP00000380227->ENSP00000364094->ENSP00000386896 7

1958.ENSP00000263967->ENSP00000304895->ENSP00000268035->ENSP00000206249->ENSP00000254227->ENSP00000342470->ENSP00000363868->ENSP00000393286 123

1959.ENSP00000263967->ENSP00000304895->ENSP00000268035->ENSP00000206249->ENSP00000254227->ENSP00000342470->ENSP00000363868->ENSP00000393870 123

1960.ENSP00000263967->ENSP00000304895->ENSP00000343204->ENSP00000264657->ENSP00000263253->ENSP00000262160->ENSP00000287727->ENSP00000400104 106

1961.ENSP00000263967->ENSP00000304895->ENSP00000339007->ENSP00000312435->ENSP00000400365 16

1962.ENSP00000263967->ENSP00000304895->ENSP00000268035->ENSP00000206249->ENSP00000254227->ENSP00000342470->ENSP00000363868->ENSP00000402590 123

1963.ENSP00000263967->ENSP00000304895->ENSP00000268035->ENSP00000206249->ENSP00000399968->ENSP00000419692 5

1964.ENSP00000264144->ENSP00000340937->ENSP00000200181->ENSP00000386896->ENSP00000364094->ENSP00000380227->ENSP00000228307->ENSP00000299421->ENSP00000270202->ENSP00000228872->ENSP00000265734 23

1965.ENSP00000264144->ENSP00000252999->ENSP00000354360->ENSP00000230538->ENSP00000342307->ENSP00000306043->ENSP00000255465->ENSP00000266970 22

1966.ENSP00000264144->ENSP00000340937->ENSP00000200181->ENSP00000386896->ENSP00000364094->ENSP00000380227->ENSP00000228307->ENSP00000299421->ENSP00000270202->ENSP00000417281->ENSP00000267163 23

1967.ENSP00000264144->ENSP00000340937->ENSP00000200181->ENSP00000386896->ENSP00000364094->ENSP00000380227->ENSP00000228307->ENSP00000299421->ENSP00000270202->ENSP00000352121->ENSP00000269300 30

1968.ENSP00000264144->ENSP00000340937->ENSP00000200181->ENSP00000386896->ENSP00000364094->ENSP00000380227->ENSP00000228307->ENSP00000341189->ENSP00000361021->ENSP00000269305 22

1969.ENSP00000264144->ENSP00000340937->ENSP00000200181->ENSP00000386896->ENSP00000364094->ENSP00000380227->ENSP00000228307->ENSP00000299421->ENSP00000270202 21

1970.ENSP00000264144->ENSP00000340937->ENSP00000200181->ENSP00000386896->ENSP00000364094->ENSP00000380227->ENSP00000228307->ENSP00000299421->ENSP00000270202->ENSP00000228872->ENSP00000274255 23

1971.ENSP00000264144->ENSP00000340937->ENSP00000200181->ENSP00000386896->ENSP00000364094->ENSP00000380227->ENSP00000228307->ENSP00000346300->ENSP00000264033->ENSP00000274335 21

1972.ENSP00000264144->ENSP00000340937->ENSP00000200181->ENSP00000386896->ENSP00000364094->ENSP00000380227->ENSP00000228307->ENSP00000299421->ENSP00000270202->ENSP00000228872->ENSP00000257904->ENSP00000276925 24

1973.ENSP00000264144->ENSP00000340937->ENSP00000200181->ENSP00000386896->ENSP00000364094->ENSP00000380227->ENSP00000228307->ENSP00000299421->ENSP00000270202->ENSP00000289153 25

1974.ENSP00000264144->ENSP00000340937->ENSP00000200181->ENSP00000386896->ENSP00000364094->ENSP00000296585 17

1975.ENSP00000264144->ENSP00000340937->ENSP00000200181->ENSP00000386896->ENSP00000364094->ENSP00000380227->ENSP00000228307->ENSP00000299421->ENSP00000270202->ENSP00000309103->ENSP00000302564 23

1976.ENSP00000264144->ENSP00000340937->ENSP00000200181->ENSP00000386896->ENSP00000364094->ENSP00000380227->ENSP00000228307->ENSP00000300574->ENSP00000381107->ENSP00000348461->ENSP00000356505->ENSP00000220764->ENSP00000348380->ENSP00000305638 1038

1977.ENSP00000264144->ENSP00000252999->ENSP00000307156 7

1978.ENSP00000264144->ENSP00000340937->ENSP00000200181->ENSP00000386896->ENSP00000364094->ENSP00000380227->ENSP00000228307->ENSP00000341189->ENSP00000361021->ENSP00000269305->ENSP00000353059->ENSP00000307786 30

1979.ENSP00000264144->ENSP00000252999->ENSP00000354360->ENSP00000230538->ENSP00000342307->ENSP00000306043->ENSP00000255465->ENSP00000266970->ENSP00000309181 23

1980.ENSP00000264144->ENSP00000252999->ENSP00000354360->ENSP00000230538->ENSP00000342307->ENSP00000306043->ENSP00000255465->ENSP00000266970->ENSP00000311083 23

1981.ENSP00000264144->ENSP00000324532 15

1982.ENSP00000264144->ENSP00000340937->ENSP00000200181->ENSP00000386896->ENSP00000364094->ENSP00000367316->ENSP00000303242->ENSP00000264832->ENSP00000226574->ENSP00000327251 43

1983.ENSP00000264144->ENSP00000340937->ENSP00000200181->ENSP00000386896->ENSP00000364094->ENSP00000380227->ENSP00000228307->ENSP00000299421->ENSP00000270202->ENSP00000309103->ENSP00000329623 23

1984.ENSP00000264144->ENSP00000340937->ENSP00000200181->ENSP00000386896->ENSP00000364094->ENSP00000380227->ENSP00000228307->ENSP00000341189->ENSP00000361021->ENSP00000269305->ENSP00000353059->ENSP00000330237 25

1985.ENSP00000264144->ENSP00000340937->ENSP00000200181->ENSP00000386896->ENSP00000364094->ENSP00000380227->ENSP00000228307->ENSP00000341189->ENSP00000350941->ENSP00000206249->ENSP00000399968->ENSP00000419692->ENSP00000332296 39

1986.ENSP00000264144->ENSP00000340937->ENSP00000200181->ENSP00000386896->ENSP00000364094->ENSP00000380227->ENSP00000228307->ENSP00000300574->ENSP00000275493->ENSP00000344818->ENSP00000316840->ENSP00000361359->ENSP00000332468 25

1987.ENSP00000264144->ENSP00000340937->ENSP00000200181->ENSP00000386896->ENSP00000364094->ENSP00000380227->ENSP00000228307->ENSP00000341189->ENSP00000350941->ENSP00000206249->ENSP00000263253->ENSP00000384273->ENSP00000339151 24

1988.ENSP00000264144->ENSP00000340937->ENSP00000200181->ENSP00000386896->ENSP00000364094->ENSP00000380227->ENSP00000228307->ENSP00000341189 19

1989.ENSP00000264144->ENSP00000340937->ENSP00000200181->ENSP00000386896->ENSP00000364094->ENSP00000380227->ENSP00000228307->ENSP00000341189->ENSP00000361021->ENSP00000269305->ENSP00000293195->ENSP00000342087 56

1990.ENSP00000264144->ENSP00000252999->ENSP00000354360->ENSP00000230538->ENSP00000342307->ENSP00000306043->ENSP00000274026->ENSP00000345571 22

1991.ENSP00000264144->ENSP00000340937->ENSP00000200181->ENSP00000386896->ENSP00000364094->ENSP00000346839 17

1992.ENSP00000264144->ENSP00000340937->ENSP00000200181->ENSP00000386896->ENSP00000364094->ENSP00000380227->ENSP00000228307->ENSP00000300574->ENSP00000275493->ENSP00000344818->ENSP00000316840->ENSP00000216160->ENSP00000347858 25

1993.ENSP00000264144->ENSP00000324532->ENSP00000348384 16

1994.ENSP00000264144->ENSP00000340937->ENSP00000200181->ENSP00000386896->ENSP00000364094->ENSP00000380227->ENSP00000228307->ENSP00000341189->ENSP00000350941->ENSP00000206249->ENSP00000367207->ENSP00000351490 24

1995.ENSP00000264144->ENSP00000340937->ENSP00000200181->ENSP00000386896->ENSP00000364094->ENSP00000380227->ENSP00000228307->ENSP00000299421->ENSP00000270202->ENSP00000352121 27

1996.ENSP00000264144->ENSP00000340937->ENSP00000200181->ENSP00000386896->ENSP00000364094->ENSP00000380227->ENSP00000228307->ENSP00000341189->ENSP00000350941->ENSP00000206249->ENSP00000327213->ENSP00000352900 36

1997.ENSP00000264144->ENSP00000340937->ENSP00000200181->ENSP00000386896->ENSP00000364094->ENSP00000380227->ENSP00000228307->ENSP00000341189->ENSP00000361021->ENSP00000269305->ENSP00000353059 24

1998.ENSP00000264144->ENSP00000340937->ENSP00000200181->ENSP00000386896->ENSP00000364094->ENSP00000282588->ENSP00000364979->ENSP00000353654 29

1999.ENSP00000264144->ENSP00000252999->ENSP00000354360 7

2000.ENSP00000264144->ENSP00000340937->ENSP00000200181->ENSP00000386896->ENSP00000364094->ENSP00000380227->ENSP00000228307->ENSP00000299421->ENSP00000270202->ENSP00000417281->ENSP00000267163->ENSP00000355249 24

2001.ENSP00000264144->ENSP00000340937->ENSP00000200181->ENSP00000386896->ENSP00000364094->ENSP00000380227->ENSP00000228307->ENSP00000341189->ENSP00000350941->ENSP00000264657->ENSP00000258743->ENSP00000356438 26

2002.ENSP00000264144->ENSP00000340937->ENSP00000200181->ENSP00000386896->ENSP00000364094->ENSP00000380227->ENSP00000228307->ENSP00000300574->ENSP00000275493->ENSP00000344818->ENSP00000316840->ENSP00000358622 24

2003.ENSP00000264144->ENSP00000340937->ENSP00000200181->ENSP00000386896->ENSP00000364094->ENSP00000380227->ENSP00000228307->ENSP00000341189->ENSP00000350941->ENSP00000206249->ENSP00000263253->ENSP00000384273->ENSP00000359424 24

2004.ENSP00000264144->ENSP00000340937->ENSP00000200181->ENSP00000386896->ENSP00000364094->ENSP00000380227->ENSP00000228307->ENSP00000341189->ENSP00000361021 21

2005.ENSP00000264144->ENSP00000252999->ENSP00000354360->ENSP00000230538->ENSP00000342307->ENSP00000256442->ENSP00000300093->ENSP00000263753->ENSP00000418447->ENSP00000348784->ENSP00000379866->ENSP00000379823->ENSP00000331902->ENSP00000361290 61

2006.ENSP00000264144->ENSP00000340937->ENSP00000200181->ENSP00000386896->ENSP00000364094->ENSP00000380227->ENSP00000228307->ENSP00000300574->ENSP00000275493->ENSP00000344818->ENSP00000316840->ENSP00000361359->ENSP00000362994 25

2007.ENSP00000264144->ENSP00000340937->ENSP00000200181->ENSP00000386896->ENSP00000364094->ENSP00000380227->ENSP00000228307->ENSP00000341189->ENSP00000350941->ENSP00000206249->ENSP00000327213->ENSP00000363812 48

2008.ENSP00000264144->ENSP00000340937->ENSP00000200181->ENSP00000386896->ENSP00000364094 16

2009.ENSP00000264144->ENSP00000340937->ENSP00000200181->ENSP00000386896->ENSP00000364094->ENSP00000282588->ENSP00000364979 28

2010.ENSP00000264144->ENSP00000340937->ENSP00000200181->ENSP00000386896->ENSP00000364094->ENSP00000380227->ENSP00000228307->ENSP00000341189->ENSP00000361021->ENSP00000366563 33

2011.ENSP00000264144->ENSP00000340937->ENSP00000200181->ENSP00000386896->ENSP00000364094->ENSP00000380227->ENSP00000228307->ENSP00000341189->ENSP00000350941->ENSP00000206249->ENSP00000367207 23

2012.ENSP00000264144->ENSP00000340937->ENSP00000200181->ENSP00000386896->ENSP00000364094->ENSP00000380227->ENSP00000228307->ENSP00000341189->ENSP00000350941->ENSP00000206249->ENSP00000254227->ENSP00000342470->ENSP00000363868->ENSP00000372703 141

2013.ENSP00000264144->ENSP00000252999->ENSP00000258341->ENSP00000374309 9

2014.ENSP00000264144->ENSP00000340937->ENSP00000200181->ENSP00000386896->ENSP00000364094->ENSP00000380227->ENSP00000228307->ENSP00000299421->ENSP00000270202->ENSP00000348986->ENSP00000375892 27

2015.ENSP00000264144->ENSP00000340937->ENSP00000200181->ENSP00000386896->ENSP00000364094->ENSP00000380227->ENSP00000228307->ENSP00000341189->ENSP00000350941->ENSP00000264657->ENSP00000376765 24

2016.ENSP00000264144->ENSP00000252999->ENSP00000354360->ENSP00000230538->ENSP00000342307->ENSP00000256442->ENSP00000300093->ENSP00000263753->ENSP00000418447->ENSP00000348784->ENSP00000379866 58

2017.ENSP00000264144->ENSP00000340937->ENSP00000200181->ENSP00000386896->ENSP00000364094->ENSP00000380227->ENSP00000228307->ENSP00000341189->ENSP00000361021->ENSP00000269305->ENSP00000324897->ENSP00000381648 24

2018.ENSP00000264144->ENSP00000340937->ENSP00000200181->ENSP00000386896->ENSP00000364094->ENSP00000380227->ENSP00000228307->ENSP00000341189->ENSP00000350941->ENSP00000206249->ENSP00000263253->ENSP00000384273 23

2019.ENSP00000264144->ENSP00000340937->ENSP00000200181->ENSP00000386896 15

2020.ENSP00000264144->ENSP00000340937->ENSP00000200181->ENSP00000386896->ENSP00000364094->ENSP00000380227->ENSP00000228307->ENSP00000341189->ENSP00000350941->ENSP00000206249->ENSP00000254227->ENSP00000342470->ENSP00000363868->ENSP00000393286 141

2021.ENSP00000264144->ENSP00000340937->ENSP00000200181->ENSP00000386896->ENSP00000364094->ENSP00000380227->ENSP00000228307->ENSP00000341189->ENSP00000350941->ENSP00000206249->ENSP00000254227->ENSP00000342470->ENSP00000363868->ENSP00000393870 141

2022.ENSP00000264144->ENSP00000340937->ENSP00000200181->ENSP00000386896->ENSP00000364094->ENSP00000346839->ENSP00000221930->ENSP00000262160->ENSP00000287727->ENSP00000400104 122

2023.ENSP00000264144->ENSP00000252999->ENSP00000354360->ENSP00000400365 8

2024.ENSP00000264144->ENSP00000340937->ENSP00000200181->ENSP00000386896->ENSP00000364094->ENSP00000380227->ENSP00000228307->ENSP00000341189->ENSP00000350941->ENSP00000206249->ENSP00000254227->ENSP00000342470->ENSP00000363868->ENSP00000402590 141

2025.ENSP00000264144->ENSP00000340937->ENSP00000200181->ENSP00000386896->ENSP00000364094->ENSP00000380227->ENSP00000228307->ENSP00000341189->ENSP00000350941->ENSP00000206249->ENSP00000399968->ENSP00000419692 23

2026.ENSP00000265734->ENSP00000244741->ENSP00000266970 2

2027.ENSP00000265734->ENSP00000267163 2

2028.ENSP00000265734->ENSP00000228872->ENSP00000270202->ENSP00000352121->ENSP00000269300 11

2029.ENSP00000265734->ENSP00000355153->ENSP00000269305 2

2030.ENSP00000265734->ENSP00000228872->ENSP00000270202 2

2031.ENSP00000265734->ENSP00000228872->ENSP00000274255 2

2032.ENSP00000265734->ENSP00000228872->ENSP00000270202->ENSP00000274335 5

2033.ENSP00000265734->ENSP00000362082->ENSP00000257904->ENSP00000276925 3

2034.ENSP00000265734->ENSP00000228872->ENSP00000270202->ENSP00000289153 6

2035.ENSP00000265734->ENSP00000228872->ENSP00000270202->ENSP00000299421->ENSP00000228307->ENSP00000380227->ENSP00000364094->ENSP00000296585 8

2036.ENSP00000265734->ENSP00000355153->ENSP00000269305->ENSP00000302564 4

2037.ENSP00000265734->ENSP00000228872->ENSP00000270202->ENSP00000348461->ENSP00000356505->ENSP00000220764->ENSP00000348380->ENSP00000305638 1020

2038.ENSP00000265734->ENSP00000244741->ENSP00000255465->ENSP00000306043->ENSP00000342307->ENSP00000230538->ENSP00000354360->ENSP00000252999->ENSP00000307156 26

2039.ENSP00000265734->ENSP00000355153->ENSP00000269305->ENSP00000353059->ENSP00000307786 10

2040.ENSP00000265734->ENSP00000244741->ENSP00000266970->ENSP00000309181 3

2041.ENSP00000265734->ENSP00000244741->ENSP00000266970->ENSP00000311083 3

2042.ENSP00000265734->ENSP00000228872->ENSP00000270202->ENSP00000299421->ENSP00000228307->ENSP00000380227->ENSP00000364094->ENSP00000386896->ENSP00000200181->ENSP00000340937->ENSP00000348384->ENSP00000324532 18

2043.ENSP00000265734->ENSP00000227507->ENSP00000344456->ENSP00000359206->ENSP00000216797->ENSP00000226574->ENSP00000327251 24

2044.ENSP00000265734->ENSP00000355153->ENSP00000269305->ENSP00000329623 3

2045.ENSP00000265734->ENSP00000355153->ENSP00000269305->ENSP00000353059->ENSP00000330237 5

2046.ENSP00000265734->ENSP00000227507->ENSP00000344456->ENSP00000363822->ENSP00000399968->ENSP00000419692->ENSP00000332296 21

2047.ENSP00000265734->ENSP00000244741->ENSP00000344818->ENSP00000316840->ENSP00000361359->ENSP00000332468 6

2048.ENSP00000265734->ENSP00000227507->ENSP00000344456->ENSP00000359206->ENSP00000216797->ENSP00000339151 5

2049.ENSP00000265734->ENSP00000355153->ENSP00000269305->ENSP00000361021->ENSP00000341189 5

2050.ENSP00000265734->ENSP00000355153->ENSP00000269305->ENSP00000293195->ENSP00000342087 36

2051.ENSP00000265734->ENSP00000227507->ENSP00000345571 2

2052.ENSP00000265734->ENSP00000228872->ENSP00000270202->ENSP00000299421->ENSP00000228307->ENSP00000380227->ENSP00000346839 7

2053.ENSP00000265734->ENSP00000355153->ENSP00000269305->ENSP00000329623->ENSP00000311032->ENSP00000347858 6

2054.ENSP00000265734->ENSP00000228872->ENSP00000270202->ENSP00000299421->ENSP00000228307->ENSP00000380227->ENSP00000364094->ENSP00000386896->ENSP00000200181->ENSP00000340937->ENSP00000348384 17

2055.ENSP00000265734->ENSP00000244741->ENSP00000367207->ENSP00000351490 4

2056.ENSP00000265734->ENSP00000228872->ENSP00000270202->ENSP00000352121 8

2057.ENSP00000265734->ENSP00000227507->ENSP00000206249->ENSP00000327213->ENSP00000352900 18

2058.ENSP00000265734->ENSP00000355153->ENSP00000269305->ENSP00000353059 4

2059.ENSP00000265734->ENSP00000228872->ENSP00000270202->ENSP00000299421->ENSP00000228307->ENSP00000380227->ENSP00000364094->ENSP00000282588->ENSP00000364979->ENSP00000353654 20

2060.ENSP00000265734->ENSP00000244741->ENSP00000255465->ENSP00000306043->ENSP00000342307->ENSP00000230538->ENSP00000354360 16

2061.ENSP00000265734->ENSP00000267163->ENSP00000355249 3

2062.ENSP00000265734->ENSP00000227507->ENSP00000264657->ENSP00000258743->ENSP00000356438 8

2063.ENSP00000265734->ENSP00000227507->ENSP00000344456->ENSP00000359206->ENSP00000216797->ENSP00000358622 5

2064.ENSP00000265734->ENSP00000227507->ENSP00000344456->ENSP00000359206->ENSP00000216797->ENSP00000359424 5

2065.ENSP00000265734->ENSP00000355153->ENSP00000269305->ENSP00000361021 3

2066.ENSP00000265734->ENSP00000355153->ENSP00000269305->ENSP00000278916->ENSP00000312995->ENSP00000300093->ENSP00000263753->ENSP00000418447->ENSP00000348784->ENSP00000379866->ENSP00000379823->ENSP00000331902->ENSP00000361290 46

2067.ENSP00000265734->ENSP00000244741->ENSP00000344818->ENSP00000316840->ENSP00000361359->ENSP00000362994 6

2068.ENSP00000265734->ENSP00000227507->ENSP00000206249->ENSP00000327213->ENSP00000363812 30

2069.ENSP00000265734->ENSP00000228872->ENSP00000270202->ENSP00000299421->ENSP00000228307->ENSP00000380227->ENSP00000364094 7

2070.ENSP00000265734->ENSP00000228872->ENSP00000270202->ENSP00000299421->ENSP00000228307->ENSP00000380227->ENSP00000364094->ENSP00000282588->ENSP00000364979 19

2071.ENSP00000265734->ENSP00000355153->ENSP00000269305->ENSP00000361021->ENSP00000366563 15

2072.ENSP00000265734->ENSP00000244741->ENSP00000367207 3

2073.ENSP00000265734->ENSP00000227507->ENSP00000344456->ENSP00000326366->ENSP00000284981->ENSP00000252486->ENSP00000252444->ENSP00000233242->ENSP00000236850->ENSP00000363868->ENSP00000372703 123

2074.ENSP00000265734->ENSP00000244741->ENSP00000255465->ENSP00000306043->ENSP00000342307->ENSP00000230538->ENSP00000222399->ENSP00000374309 24

2075.ENSP00000265734->ENSP00000228872->ENSP00000270202->ENSP00000348986->ENSP00000375892 8

2076.ENSP00000265734->ENSP00000227507->ENSP00000264657->ENSP00000376765 6

2077.ENSP00000265734->ENSP00000355153->ENSP00000269305->ENSP00000278916->ENSP00000312995->ENSP00000300093->ENSP00000263753->ENSP00000418447->ENSP00000348784->ENSP00000379866 43

2078.ENSP00000265734->ENSP00000355153->ENSP00000269305->ENSP00000324897->ENSP00000381648 4

2079.ENSP00000265734->ENSP00000355153->ENSP00000269305->ENSP00000262367->ENSP00000384273 4

2080.ENSP00000265734->ENSP00000228872->ENSP00000270202->ENSP00000299421->ENSP00000228307->ENSP00000380227->ENSP00000364094->ENSP00000386896 8

2081.ENSP00000265734->ENSP00000227507->ENSP00000344456->ENSP00000326366->ENSP00000284981->ENSP00000252486->ENSP00000252444->ENSP00000233242->ENSP00000236850->ENSP00000363868->ENSP00000393286 123

2082.ENSP00000265734->ENSP00000227507->ENSP00000344456->ENSP00000326366->ENSP00000284981->ENSP00000252486->ENSP00000252444->ENSP00000233242->ENSP00000236850->ENSP00000363868->ENSP00000393870 123

2083.ENSP00000265734->ENSP00000227507->ENSP00000345571->ENSP00000329357->ENSP00000332973->ENSP00000287727->ENSP00000400104 105

2084.ENSP00000265734->ENSP00000244741->ENSP00000255465->ENSP00000306043->ENSP00000342307->ENSP00000230538->ENSP00000354360->ENSP00000400365 17

2085.ENSP00000265734->ENSP00000227507->ENSP00000344456->ENSP00000326366->ENSP00000284981->ENSP00000252486->ENSP00000252444->ENSP00000233242->ENSP00000236850->ENSP00000363868->ENSP00000402590 123

2086.ENSP00000265734->ENSP00000227507->ENSP00000344456->ENSP00000363822->ENSP00000399968->ENSP00000419692 5

2087.ENSP00000266970->ENSP00000267163 1

2088.ENSP00000266970->ENSP00000228872->ENSP00000270202->ENSP00000352121->ENSP00000269300 11

2089.ENSP00000266970->ENSP00000269305 1

2090.ENSP00000266970->ENSP00000228872->ENSP00000270202 2

2091.ENSP00000266970->ENSP00000274255 2

2092.ENSP00000266970->ENSP00000269305->ENSP00000353483->ENSP00000304895->ENSP00000274335 4

2093.ENSP00000266970->ENSP00000228872->ENSP00000257904->ENSP00000276925 3

2094.ENSP00000266970->ENSP00000228872->ENSP00000270202->ENSP00000289153 6

2095.ENSP00000266970->ENSP00000228872->ENSP00000270202->ENSP00000299421->ENSP00000228307->ENSP00000380227->ENSP00000364094->ENSP00000296585 8

2096.ENSP00000266970->ENSP00000269305->ENSP00000302564 3

2097.ENSP00000266970->ENSP00000228872->ENSP00000270202->ENSP00000348461->ENSP00000356505->ENSP00000220764->ENSP00000348380->ENSP00000305638 1020

2098.ENSP00000266970->ENSP00000274026->ENSP00000306043->ENSP00000342307->ENSP00000230538->ENSP00000354360->ENSP00000252999->ENSP00000307156 25

2099.ENSP00000266970->ENSP00000269305->ENSP00000353059->ENSP00000307786 9

2100.ENSP00000266970->ENSP00000309181 1

2101.ENSP00000266970->ENSP00000311083 1

2102.ENSP00000266970->ENSP00000228872->ENSP00000270202->ENSP00000299421->ENSP00000228307->ENSP00000380227->ENSP00000364094->ENSP00000386896->ENSP00000200181->ENSP00000340937->ENSP00000348384->ENSP00000324532 18

2103.ENSP00000266970->ENSP00000269305->ENSP00000263253->ENSP00000384273->ENSP00000226574->ENSP00000327251 23

2104.ENSP00000266970->ENSP00000269305->ENSP00000329623 2

2105.ENSP00000266970->ENSP00000269305->ENSP00000353059->ENSP00000330237 4

2106.ENSP00000266970->ENSP00000269305->ENSP00000212015->ENSP00000264867->ENSP00000287820->ENSP00000419692->ENSP00000332296 21

2107.ENSP00000266970->ENSP00000269305->ENSP00000344818->ENSP00000316840->ENSP00000361359->ENSP00000332468 5

2108.ENSP00000266970->ENSP00000269305->ENSP00000263253->ENSP00000384273->ENSP00000339151 4

2109.ENSP00000266970->ENSP00000269305->ENSP00000361021->ENSP00000341189 4

2110.ENSP00000266970->ENSP00000269305->ENSP00000293195->ENSP00000342087 35

2111.ENSP00000266970->ENSP00000345571 2

2112.ENSP00000266970->ENSP00000269305->ENSP00000263253->ENSP00000262160->ENSP00000221930->ENSP00000346839 7

2113.ENSP00000266970->ENSP00000269305->ENSP00000329623->ENSP00000311032->ENSP00000347858 5

2114.ENSP00000266970->ENSP00000228872->ENSP00000270202->ENSP00000299421->ENSP00000228307->ENSP00000380227->ENSP00000364094->ENSP00000386896->ENSP00000200181->ENSP00000340937->ENSP00000348384 17

2115.ENSP00000266970->ENSP00000244741->ENSP00000367207->ENSP00000351490 4

2116.ENSP00000266970->ENSP00000228872->ENSP00000270202->ENSP00000352121 8

2117.ENSP00000266970->ENSP00000227507->ENSP00000206249->ENSP00000327213->ENSP00000352900 18

2118.ENSP00000266970->ENSP00000269305->ENSP00000353059 3

2119.ENSP00000266970->ENSP00000228872->ENSP00000270202->ENSP00000299421->ENSP00000228307->ENSP00000380227->ENSP00000364094->ENSP00000282588->ENSP00000364979->ENSP00000353654 20

2120.ENSP00000266970->ENSP00000274026->ENSP00000306043->ENSP00000342307->ENSP00000230538->ENSP00000354360 15

2121.ENSP00000266970->ENSP00000267163->ENSP00000355249 2

2122.ENSP00000266970->ENSP00000269305->ENSP00000344352->ENSP00000360266->ENSP00000356438 8

2123.ENSP00000266970->ENSP00000269305->ENSP00000344818->ENSP00000316840->ENSP00000358622 4

2124.ENSP00000266970->ENSP00000269305->ENSP00000263253->ENSP00000384273->ENSP00000359424 4

2125.ENSP00000266970->ENSP00000269305->ENSP00000361021 2

2126.ENSP00000266970->ENSP00000303706->ENSP00000278916->ENSP00000312995->ENSP00000300093->ENSP00000263753->ENSP00000418447->ENSP00000348784->ENSP00000379866->ENSP00000379823->ENSP00000331902->ENSP00000361290 45

2127.ENSP00000266970->ENSP00000269305->ENSP00000344818->ENSP00000316840->ENSP00000361359->ENSP00000362994 5

2128.ENSP00000266970->ENSP00000227507->ENSP00000206249->ENSP00000327213->ENSP00000363812 30

2129.ENSP00000266970->ENSP00000228872->ENSP00000270202->ENSP00000299421->ENSP00000228307->ENSP00000380227->ENSP00000364094 7

2130.ENSP00000266970->ENSP00000228872->ENSP00000270202->ENSP00000299421->ENSP00000228307->ENSP00000380227->ENSP00000364094->ENSP00000282588->ENSP00000364979 19

2131.ENSP00000266970->ENSP00000269305->ENSP00000361021->ENSP00000366563 14

2132.ENSP00000266970->ENSP00000244741->ENSP00000367207 3

2133.ENSP00000266970->ENSP00000227507->ENSP00000344456->ENSP00000326366->ENSP00000284981->ENSP00000252486->ENSP00000252444->ENSP00000233242->ENSP00000236850->ENSP00000363868->ENSP00000372703 123

2134.ENSP00000266970->ENSP00000274026->ENSP00000306043->ENSP00000342307->ENSP00000230538->ENSP00000222399->ENSP00000374309 23

2135.ENSP00000266970->ENSP00000228872->ENSP00000270202->ENSP00000348986->ENSP00000375892 8

2136.ENSP00000266970->ENSP00000227507->ENSP00000264657->ENSP00000376765 6

2137.ENSP00000266970->ENSP00000303706->ENSP00000278916->ENSP00000312995->ENSP00000300093->ENSP00000263753->ENSP00000418447->ENSP00000348784->ENSP00000379866 42

2138.ENSP00000266970->ENSP00000269305->ENSP00000324897->ENSP00000381648 3

2139.ENSP00000266970->ENSP00000269305->ENSP00000263253->ENSP00000384273 3

2140.ENSP00000266970->ENSP00000228872->ENSP00000270202->ENSP00000299421->ENSP00000228307->ENSP00000380227->ENSP00000364094->ENSP00000386896 8

2141.ENSP00000266970->ENSP00000227507->ENSP00000344456->ENSP00000326366->ENSP00000284981->ENSP00000252486->ENSP00000252444->ENSP00000233242->ENSP00000236850->ENSP00000363868->ENSP00000393286 123

2142.ENSP00000266970->ENSP00000227507->ENSP00000344456->ENSP00000326366->ENSP00000284981->ENSP00000252486->ENSP00000252444->ENSP00000233242->ENSP00000236850->ENSP00000363868->ENSP00000393870 123

2143.ENSP00000266970->ENSP00000269305->ENSP00000329357->ENSP00000332973->ENSP00000287727->ENSP00000400104 104

2144.ENSP00000266970->ENSP00000274026->ENSP00000306043->ENSP00000342307->ENSP00000230538->ENSP00000354360->ENSP00000400365 16

2145.ENSP00000266970->ENSP00000227507->ENSP00000344456->ENSP00000326366->ENSP00000284981->ENSP00000252486->ENSP00000252444->ENSP00000233242->ENSP00000236850->ENSP00000363868->ENSP00000402590 123

2146.ENSP00000266970->ENSP00000269305->ENSP00000212015->ENSP00000264867->ENSP00000287820->ENSP00000419692 5

2147.ENSP00000267163->ENSP00000417281->ENSP00000270202->ENSP00000352121->ENSP00000269300 11

2148.ENSP00000267163->ENSP00000269305 1

2149.ENSP00000267163->ENSP00000417281->ENSP00000270202 2

2150.ENSP00000267163->ENSP00000266970->ENSP00000274255 3

2151.ENSP00000267163->ENSP00000269305->ENSP00000353483->ENSP00000304895->ENSP00000274335 4

2152.ENSP00000267163->ENSP00000257904->ENSP00000276925 2

2153.ENSP00000267163->ENSP00000417281->ENSP00000270202->ENSP00000289153 6

2154.ENSP00000267163->ENSP00000269305->ENSP00000361021->ENSP00000341189->ENSP00000228307->ENSP00000380227->ENSP00000364094->ENSP00000296585 8

2155.ENSP00000267163->ENSP00000269305->ENSP00000302564 3

2156.ENSP00000267163->ENSP00000417281->ENSP00000270202->ENSP00000348461->ENSP00000356505->ENSP00000220764->ENSP00000348380->ENSP00000305638 1020

2157.ENSP00000267163->ENSP00000266970->ENSP00000255465->ENSP00000306043->ENSP00000342307->ENSP00000230538->ENSP00000354360->ENSP00000252999->ENSP00000307156 26

2158.ENSP00000267163->ENSP00000269305->ENSP00000353059->ENSP00000307786 9

2159.ENSP00000267163->ENSP00000266970->ENSP00000309181 2

2160.ENSP00000267163->ENSP00000266970->ENSP00000311083 2

2161.ENSP00000267163->ENSP00000269305->ENSP00000361021->ENSP00000341189->ENSP00000228307->ENSP00000380227->ENSP00000364094->ENSP00000386896->ENSP00000200181->ENSP00000340937->ENSP00000348384->ENSP00000324532 18

2162.ENSP00000267163->ENSP00000362649->ENSP00000384273->ENSP00000226574->ENSP00000327251 22

2163.ENSP00000267163->ENSP00000269305->ENSP00000329623 2

2164.ENSP00000267163->ENSP00000269305->ENSP00000353059->ENSP00000330237 4

2165.ENSP00000267163->ENSP00000350720->ENSP00000231509->ENSP00000320940->ENSP00000348827->ENSP00000419692->ENSP00000332296 21

2166.ENSP00000267163->ENSP00000417281->ENSP00000344818->ENSP00000316840->ENSP00000361359->ENSP00000332468 5

2167.ENSP00000267163->ENSP00000362649->ENSP00000384273->ENSP00000339151 3

2168.ENSP00000267163->ENSP00000269305->ENSP00000361021->ENSP00000341189 4

2169.ENSP00000267163->ENSP00000269305->ENSP00000293195->ENSP00000342087 35

2170.ENSP00000267163->ENSP00000345571 1

2171.ENSP00000267163->ENSP00000269305->ENSP00000263253->ENSP00000262160->ENSP00000221930->ENSP00000346839 7

2172.ENSP00000267163->ENSP00000269305->ENSP00000355759->ENSP00000311032->ENSP00000347858 5

2173.ENSP00000267163->ENSP00000269305->ENSP00000361021->ENSP00000341189->ENSP00000228307->ENSP00000380227->ENSP00000364094->ENSP00000386896->ENSP00000200181->ENSP00000340937->ENSP00000348384 17

2174.ENSP00000267163->ENSP00000367207->ENSP00000351490 3

2175.ENSP00000267163->ENSP00000417281->ENSP00000270202->ENSP00000352121 8

2176.ENSP00000267163->ENSP00000227507->ENSP00000206249->ENSP00000327213->ENSP00000352900 18

2177.ENSP00000267163->ENSP00000269305->ENSP00000353059 3

2178.ENSP00000267163->ENSP00000269305->ENSP00000361021->ENSP00000341189->ENSP00000228307->ENSP00000380227->ENSP00000364094->ENSP00000282588->ENSP00000364979->ENSP00000353654 20

2179.ENSP00000267163->ENSP00000266970->ENSP00000255465->ENSP00000306043->ENSP00000342307->ENSP00000230538->ENSP00000354360 16

2180.ENSP00000267163->ENSP00000355249 1

2181.ENSP00000267163->ENSP00000269305->ENSP00000344352->ENSP00000360266->ENSP00000356438 8

2182.ENSP00000267163->ENSP00000417281->ENSP00000344818->ENSP00000316840->ENSP00000358622 4

2183.ENSP00000267163->ENSP00000362649->ENSP00000384273->ENSP00000359424 3

2184.ENSP00000267163->ENSP00000269305->ENSP00000361021 2

2185.ENSP00000267163->ENSP00000269305->ENSP00000372023->ENSP00000321656->ENSP00000300093->ENSP00000263753->ENSP00000418447->ENSP00000348784->ENSP00000379866->ENSP00000379823->ENSP00000331902->ENSP00000361290 45

2186.ENSP00000267163->ENSP00000417281->ENSP00000344818->ENSP00000316840->ENSP00000361359->ENSP00000362994 5

2187.ENSP00000267163->ENSP00000227507->ENSP00000206249->ENSP00000327213->ENSP00000363812 30

2188.ENSP00000267163->ENSP00000269305->ENSP00000361021->ENSP00000341189->ENSP00000228307->ENSP00000380227->ENSP00000364094 7

2189.ENSP00000267163->ENSP00000269305->ENSP00000361021->ENSP00000341189->ENSP00000228307->ENSP00000380227->ENSP00000364094->ENSP00000282588->ENSP00000364979 19

2190.ENSP00000267163->ENSP00000269305->ENSP00000361021->ENSP00000366563 14

2191.ENSP00000267163->ENSP00000367207 2

2192.ENSP00000267163->ENSP00000227507->ENSP00000344456->ENSP00000326366->ENSP00000284981->ENSP00000252486->ENSP00000252444->ENSP00000233242->ENSP00000236850->ENSP00000363868->ENSP00000372703 123

2193.ENSP00000267163->ENSP00000266970->ENSP00000255465->ENSP00000306043->ENSP00000342307->ENSP00000230538->ENSP00000222399->ENSP00000374309 24

2194.ENSP00000267163->ENSP00000417281->ENSP00000270202->ENSP00000348986->ENSP00000375892 8

2195.ENSP00000267163->ENSP00000227507->ENSP00000264657->ENSP00000376765 6

2196.ENSP00000267163->ENSP00000269305->ENSP00000372023->ENSP00000321656->ENSP00000300093->ENSP00000263753->ENSP00000418447->ENSP00000348784->ENSP00000379866 42

2197.ENSP00000267163->ENSP00000269305->ENSP00000324897->ENSP00000381648 3

2198.ENSP00000267163->ENSP00000362649->ENSP00000384273 2

2199.ENSP00000267163->ENSP00000269305->ENSP00000361021->ENSP00000341189->ENSP00000228307->ENSP00000380227->ENSP00000364094->ENSP00000386896 8

2200.ENSP00000267163->ENSP00000227507->ENSP00000344456->ENSP00000326366->ENSP00000284981->ENSP00000252486->ENSP00000252444->ENSP00000233242->ENSP00000236850->ENSP00000363868->ENSP00000393286 123

2201.ENSP00000267163->ENSP00000227507->ENSP00000344456->ENSP00000326366->ENSP00000284981->ENSP00000252486->ENSP00000252444->ENSP00000233242->ENSP00000236850->ENSP00000363868->ENSP00000393870 123

2202.ENSP00000267163->ENSP00000362649->ENSP00000329357->ENSP00000332973->ENSP00000287727->ENSP00000400104 104

2203.ENSP00000267163->ENSP00000417281->ENSP00000270202->ENSP00000297494->ENSP00000341940->ENSP00000312435->ENSP00000400365 17

2204.ENSP00000267163->ENSP00000227507->ENSP00000344456->ENSP00000326366->ENSP00000284981->ENSP00000252486->ENSP00000252444->ENSP00000233242->ENSP00000236850->ENSP00000363868->ENSP00000402590 123

2205.ENSP00000267163->ENSP00000350720->ENSP00000231509->ENSP00000320940->ENSP00000348827->ENSP00000419692 5

2206.ENSP00000269300->ENSP00000352121->ENSP00000270202->ENSP00000417281->ENSP00000269305 11

2207.ENSP00000269300->ENSP00000352121->ENSP00000270202 9

2208.ENSP00000269300->ENSP00000352121->ENSP00000270202->ENSP00000228872->ENSP00000274255 11

2209.ENSP00000269300->ENSP00000352121->ENSP00000270202->ENSP00000274335 12

2210.ENSP00000269300->ENSP00000352121->ENSP00000270202->ENSP00000228872->ENSP00000257904->ENSP00000276925 12

2211.ENSP00000269300->ENSP00000352121->ENSP00000270202->ENSP00000289153 13

2212.ENSP00000269300->ENSP00000352121->ENSP00000270202->ENSP00000299421->ENSP00000228307->ENSP00000380227->ENSP00000364094->ENSP00000296585 15

2213.ENSP00000269300->ENSP00000352121->ENSP00000270202->ENSP00000309103->ENSP00000302564 11

2214.ENSP00000269300->ENSP00000352121->ENSP00000270202->ENSP00000348461->ENSP00000356505->ENSP00000220764->ENSP00000348380->ENSP00000305638 1027

2215.ENSP00000269300->ENSP00000352121->ENSP00000270202->ENSP00000228872->ENSP00000274026->ENSP00000306043->ENSP00000342307->ENSP00000230538->ENSP00000354360->ENSP00000252999->ENSP00000307156 35

2216.ENSP00000269300->ENSP00000352121->ENSP00000270202->ENSP00000417281->ENSP00000269305->ENSP00000353059->ENSP00000307786 19

2217.ENSP00000269300->ENSP00000352121->ENSP00000270202->ENSP00000228872->ENSP00000266970->ENSP00000309181 12

2218.ENSP00000269300->ENSP00000352121->ENSP00000270202->ENSP00000228872->ENSP00000274255->ENSP00000311083 12

2219.ENSP00000269300->ENSP00000352121->ENSP00000270202->ENSP00000299421->ENSP00000228307->ENSP00000380227->ENSP00000364094->ENSP00000386896->ENSP00000200181->ENSP00000340937->ENSP00000348384->ENSP00000324532 25

2220.ENSP00000269300->ENSP00000352121->ENSP00000270202->ENSP00000335153->ENSP00000231509->ENSP00000226574->ENSP00000327251 33

2221.ENSP00000269300->ENSP00000352121->ENSP00000270202->ENSP00000309103->ENSP00000329623 11

2222.ENSP00000269300->ENSP00000352121->ENSP00000270202->ENSP00000417281->ENSP00000269305->ENSP00000353059->ENSP00000330237 14

2223.ENSP00000269300->ENSP00000352121->ENSP00000270202->ENSP00000206249->ENSP00000399968->ENSP00000419692->ENSP00000332296 29

2224.ENSP00000269300->ENSP00000352121->ENSP00000270202->ENSP00000417281->ENSP00000344818->ENSP00000316840->ENSP00000361359->ENSP00000332468 14

2225.ENSP00000269300->ENSP00000352121->ENSP00000270202->ENSP00000298552->ENSP00000339151 13

2226.ENSP00000269300->ENSP00000352121->ENSP00000361021->ENSP00000341189 13

2227.ENSP00000269300->ENSP00000352121->ENSP00000270202->ENSP00000417281->ENSP00000269305->ENSP00000293195->ENSP00000342087 45

2228.ENSP00000269300->ENSP00000352121->ENSP00000270202->ENSP00000417281->ENSP00000267163->ENSP00000345571 12

2229.ENSP00000269300->ENSP00000352121->ENSP00000270202->ENSP00000299421->ENSP00000228307->ENSP00000380227->ENSP00000346839 14

2230.ENSP00000269300->ENSP00000352121->ENSP00000270202->ENSP00000309103->ENSP00000329623->ENSP00000311032->ENSP00000347858 14

2231.ENSP00000269300->ENSP00000352121->ENSP00000270202->ENSP00000299421->ENSP00000228307->ENSP00000380227->ENSP00000364094->ENSP00000386896->ENSP00000200181->ENSP00000340937->ENSP00000348384 24

2232.ENSP00000269300->ENSP00000352121->ENSP00000270202->ENSP00000244741->ENSP00000367207->ENSP00000351490 14

2233.ENSP00000269300->ENSP00000352121 3

2234.ENSP00000269300->ENSP00000352121->ENSP00000270202->ENSP00000206249->ENSP00000327213->ENSP00000352900 26

2235.ENSP00000269300->ENSP00000352121->ENSP00000270202->ENSP00000417281->ENSP00000269305->ENSP00000353059 13

2236.ENSP00000269300->ENSP00000352121->ENSP00000270202->ENSP00000299421->ENSP00000228307->ENSP00000380227->ENSP00000364094->ENSP00000282588->ENSP00000364979->ENSP00000353654 27

2237.ENSP00000269300->ENSP00000352121->ENSP00000270202->ENSP00000228872->ENSP00000274026->ENSP00000306043->ENSP00000342307->ENSP00000230538->ENSP00000354360 25

2238.ENSP00000269300->ENSP00000352121->ENSP00000270202->ENSP00000417281->ENSP00000267163->ENSP00000355249 12

2239.ENSP00000269300->ENSP00000352121->ENSP00000270202->ENSP00000387699->ENSP00000306245->ENSP00000356438 17

2240.ENSP00000269300->ENSP00000352121->ENSP00000270202->ENSP00000417281->ENSP00000344818->ENSP00000316840->ENSP00000358622 13

2241.ENSP00000269300->ENSP00000352121->ENSP00000270202->ENSP00000359424 14

2242.ENSP00000269300->ENSP00000352121->ENSP00000361021 11

2243.ENSP00000269300->ENSP00000352121->ENSP00000270202->ENSP00000417281->ENSP00000269305->ENSP00000361275->ENSP00000321656->ENSP00000300093->ENSP00000263753->ENSP00000418447->ENSP00000348784->ENSP00000379866->ENSP00000379823->ENSP00000331902->ENSP00000361290 55

2244.ENSP00000269300->ENSP00000352121->ENSP00000270202->ENSP00000417281->ENSP00000344818->ENSP00000316840->ENSP00000361359->ENSP00000362994 14

2245.ENSP00000269300->ENSP00000352121->ENSP00000270202->ENSP00000206249->ENSP00000327213->ENSP00000363812 38

2246.ENSP00000269300->ENSP00000352121->ENSP00000270202->ENSP00000299421->ENSP00000228307->ENSP00000380227->ENSP00000364094 14

2247.ENSP00000269300->ENSP00000352121->ENSP00000270202->ENSP00000299421->ENSP00000228307->ENSP00000380227->ENSP00000364094->ENSP00000282588->ENSP00000364979 26

2248.ENSP00000269300->ENSP00000352121->ENSP00000361021->ENSP00000366563 23

2249.ENSP00000269300->ENSP00000352121->ENSP00000270202->ENSP00000244741->ENSP00000367207 13

2250.ENSP00000269300->ENSP00000352121->ENSP00000270202->ENSP00000206249->ENSP00000399968->ENSP00000419692->ENSP00000342470->ENSP00000363868->ENSP00000372703 131

2251.ENSP00000269300->ENSP00000352121->ENSP00000270202->ENSP00000228872->ENSP00000274026->ENSP00000306043->ENSP00000342307->ENSP00000230538->ENSP00000222399->ENSP00000374309 33

2252.ENSP00000269300->ENSP00000352121->ENSP00000270202->ENSP00000348986->ENSP00000375892 15

2253.ENSP00000269300->ENSP00000352121->ENSP00000270202->ENSP00000348461->ENSP00000264657->ENSP00000376765 15

2254.ENSP00000269300->ENSP00000352121->ENSP00000270202->ENSP00000417281->ENSP00000269305->ENSP00000361275->ENSP00000321656->ENSP00000300093->ENSP00000263753->ENSP00000418447->ENSP00000348784->ENSP00000379866 52

2255.ENSP00000269300->ENSP00000352121->ENSP00000270202->ENSP00000417281->ENSP00000269305->ENSP00000324897->ENSP00000381648 13

2256.ENSP00000269300->ENSP00000352121->ENSP00000270202->ENSP00000387699->ENSP00000262367->ENSP00000384273 13

2257.ENSP00000269300->ENSP00000352121->ENSP00000270202->ENSP00000299421->ENSP00000228307->ENSP00000380227->ENSP00000364094->ENSP00000386896 15

2258.ENSP00000269300->ENSP00000352121->ENSP00000270202->ENSP00000206249->ENSP00000399968->ENSP00000419692->ENSP00000342470->ENSP00000363868->ENSP00000393286 131

2259.ENSP00000269300->ENSP00000352121->ENSP00000270202->ENSP00000206249->ENSP00000399968->ENSP00000419692->ENSP00000342470->ENSP00000363868->ENSP00000393870 131

2260.ENSP00000269300->ENSP00000352121->ENSP00000270202->ENSP00000354558->ENSP00000371138->ENSP00000364133->ENSP00000262160->ENSP00000287727->ENSP00000400104 114

2261.ENSP00000269300->ENSP00000352121->ENSP00000270202->ENSP00000297494->ENSP00000341940->ENSP00000312435->ENSP00000400365 24

2262.ENSP00000269300->ENSP00000352121->ENSP00000270202->ENSP00000206249->ENSP00000399968->ENSP00000419692->ENSP00000342470->ENSP00000363868->ENSP00000402590 131

2263.ENSP00000269300->ENSP00000352121->ENSP00000270202->ENSP00000206249->ENSP00000399968->ENSP00000419692 13

2264.ENSP00000269305->ENSP00000417281->ENSP00000270202 2

2265.ENSP00000269305->ENSP00000266970->ENSP00000274255 3

2266.ENSP00000269305->ENSP00000353483->ENSP00000304895->ENSP00000274335 3

2267.ENSP00000269305->ENSP00000244741->ENSP00000257904->ENSP00000276925 3

2268.ENSP00000269305->ENSP00000417281->ENSP00000270202->ENSP00000289153 6

2269.ENSP00000269305->ENSP00000361021->ENSP00000341189->ENSP00000228307->ENSP00000380227->ENSP00000364094->ENSP00000296585 7

2270.ENSP00000269305->ENSP00000302564 2

2271.ENSP00000269305->ENSP00000417281->ENSP00000270202->ENSP00000348461->ENSP00000356505->ENSP00000220764->ENSP00000348380->ENSP00000305638 1020

2272.ENSP00000269305->ENSP00000278916->ENSP00000321656->ENSP00000256442->ENSP00000342307->ENSP00000230538->ENSP00000354360->ENSP00000252999->ENSP00000307156 25

2273.ENSP00000269305->ENSP00000353059->ENSP00000307786 8

2274.ENSP00000269305->ENSP00000266970->ENSP00000309181 2

2275.ENSP00000269305->ENSP00000266970->ENSP00000311083 2

2276.ENSP00000269305->ENSP00000361021->ENSP00000341189->ENSP00000228307->ENSP00000380227->ENSP00000364094->ENSP00000386896->ENSP00000200181->ENSP00000340937->ENSP00000348384->ENSP00000324532 17

2277.ENSP00000269305->ENSP00000262367->ENSP00000384273->ENSP00000226574->ENSP00000327251 22

2278.ENSP00000269305->ENSP00000329623 1

2279.ENSP00000269305->ENSP00000353059->ENSP00000330237 3

2280.ENSP00000269305->ENSP00000212015->ENSP00000264867->ENSP00000287820->ENSP00000419692->ENSP00000332296 20

2281.ENSP00000269305->ENSP00000344818->ENSP00000316840->ENSP00000361359->ENSP00000332468 4

2282.ENSP00000269305->ENSP00000262367->ENSP00000384273->ENSP00000339151 3

2283.ENSP00000269305->ENSP00000361021->ENSP00000341189 3

2284.ENSP00000269305->ENSP00000293195->ENSP00000342087 34

2285.ENSP00000269305->ENSP00000267163->ENSP00000345571 2

2286.ENSP00000269305->ENSP00000263253->ENSP00000262160->ENSP00000221930->ENSP00000346839 6

2287.ENSP00000269305->ENSP00000344818->ENSP00000316840->ENSP00000216160->ENSP00000347858 4

2288.ENSP00000269305->ENSP00000361021->ENSP00000341189->ENSP00000228307->ENSP00000380227->ENSP00000364094->ENSP00000386896->ENSP00000200181->ENSP00000340937->ENSP00000348384 16

2289.ENSP00000269305->ENSP00000263253->ENSP00000367207->ENSP00000351490 3

2290.ENSP00000269305->ENSP00000417281->ENSP00000270202->ENSP00000352121 8

2291.ENSP00000269305->ENSP00000263253->ENSP00000206249->ENSP00000327213->ENSP00000352900 17

2292.ENSP00000269305->ENSP00000353059 2

2293.ENSP00000269305->ENSP00000361021->ENSP00000341189->ENSP00000228307->ENSP00000380227->ENSP00000364094->ENSP00000282588->ENSP00000364979->ENSP00000353654 19

2294.ENSP00000269305->ENSP00000278916->ENSP00000321656->ENSP00000256442->ENSP00000342307->ENSP00000230538->ENSP00000354360 15

2295.ENSP00000269305->ENSP00000267163->ENSP00000355249 2

2296.ENSP00000269305->ENSP00000344352->ENSP00000360266->ENSP00000356438 7

2297.ENSP00000269305->ENSP00000344818->ENSP00000316840->ENSP00000358622 3

2298.ENSP00000269305->ENSP00000262367->ENSP00000384273->ENSP00000359424 3

2299.ENSP00000269305->ENSP00000361021 1

2300.ENSP00000269305->ENSP00000278916->ENSP00000321656->ENSP00000300093->ENSP00000263753->ENSP00000418447->ENSP00000348784->ENSP00000379866->ENSP00000379823->ENSP00000331902->ENSP00000361290 44

2301.ENSP00000269305->ENSP00000344818->ENSP00000316840->ENSP00000361359->ENSP00000362994 4

2302.ENSP00000269305->ENSP00000263253->ENSP00000206249->ENSP00000327213->ENSP00000363812 29

2303.ENSP00000269305->ENSP00000361021->ENSP00000341189->ENSP00000228307->ENSP00000380227->ENSP00000364094 6

2304.ENSP00000269305->ENSP00000361021->ENSP00000341189->ENSP00000228307->ENSP00000380227->ENSP00000364094->ENSP00000282588->ENSP00000364979 18

2305.ENSP00000269305->ENSP00000361021->ENSP00000366563 13

2306.ENSP00000269305->ENSP00000263253->ENSP00000367207 2

2307.ENSP00000269305->ENSP00000263253->ENSP00000206249->ENSP00000254227->ENSP00000342470->ENSP00000363868->ENSP00000372703 122

2308.ENSP00000269305->ENSP00000278916->ENSP00000321656->ENSP00000256442->ENSP00000342307->ENSP00000230538->ENSP00000222399->ENSP00000374309 23

2309.ENSP00000269305->ENSP00000353483->ENSP00000304895->ENSP00000348986->ENSP00000375892 8

2310.ENSP00000269305->ENSP00000263253->ENSP00000264657->ENSP00000376765 5

2311.ENSP00000269305->ENSP00000278916->ENSP00000321656->ENSP00000300093->ENSP00000263753->ENSP00000418447->ENSP00000348784->ENSP00000379866 41

2312.ENSP00000269305->ENSP00000324897->ENSP00000381648 2

2313.ENSP00000269305->ENSP00000262367->ENSP00000384273 2

2314.ENSP00000269305->ENSP00000361021->ENSP00000341189->ENSP00000228307->ENSP00000380227->ENSP00000364094->ENSP00000386896 7

2315.ENSP00000269305->ENSP00000263253->ENSP00000206249->ENSP00000254227->ENSP00000342470->ENSP00000363868->ENSP00000393286 122

2316.ENSP00000269305->ENSP00000263253->ENSP00000206249->ENSP00000254227->ENSP00000342470->ENSP00000363868->ENSP00000393870 122

2317.ENSP00000269305->ENSP00000329357->ENSP00000332973->ENSP00000287727->ENSP00000400104 103

2318.ENSP00000269305->ENSP00000278916->ENSP00000321656->ENSP00000256442->ENSP00000342307->ENSP00000230538->ENSP00000354360->ENSP00000400365 16

2319.ENSP00000269305->ENSP00000263253->ENSP00000206249->ENSP00000254227->ENSP00000342470->ENSP00000363868->ENSP00000402590 122

2320.ENSP00000269305->ENSP00000212015->ENSP00000264867->ENSP00000287820->ENSP00000419692 4

2321.ENSP00000270202->ENSP00000228872->ENSP00000274255 2

2322.ENSP00000270202->ENSP00000274335 3

2323.ENSP00000270202->ENSP00000228872->ENSP00000257904->ENSP00000276925 3

2324.ENSP00000270202->ENSP00000289153 4

2325.ENSP00000270202->ENSP00000299421->ENSP00000228307->ENSP00000380227->ENSP00000364094->ENSP00000296585 6

2326.ENSP00000270202->ENSP00000309103->ENSP00000302564 2

2327.ENSP00000270202->ENSP00000348461->ENSP00000356505->ENSP00000220764->ENSP00000348380->ENSP00000305638 1018

2328.ENSP00000270202->ENSP00000228872->ENSP00000274026->ENSP00000306043->ENSP00000342307->ENSP00000230538->ENSP00000354360->ENSP00000252999->ENSP00000307156 26

2329.ENSP00000270202->ENSP00000417281->ENSP00000269305->ENSP00000353059->ENSP00000307786 10

2330.ENSP00000270202->ENSP00000228872->ENSP00000266970->ENSP00000309181 3

2331.ENSP00000270202->ENSP00000228872->ENSP00000274255->ENSP00000311083 3

2332.ENSP00000270202->ENSP00000299421->ENSP00000228307->ENSP00000380227->ENSP00000364094->ENSP00000386896->ENSP00000200181->ENSP00000340937->ENSP00000348384->ENSP00000324532 16

2333.ENSP00000270202->ENSP00000335153->ENSP00000231509->ENSP00000226574->ENSP00000327251 24

2334.ENSP00000270202->ENSP00000309103->ENSP00000329623 2

2335.ENSP00000270202->ENSP00000417281->ENSP00000269305->ENSP00000353059->ENSP00000330237 5

2336.ENSP00000270202->ENSP00000206249->ENSP00000399968->ENSP00000419692->ENSP00000332296 20

2337.ENSP00000270202->ENSP00000417281->ENSP00000344818->ENSP00000316840->ENSP00000361359->ENSP00000332468 5

2338.ENSP00000270202->ENSP00000298552->ENSP00000339151 4

2339.ENSP00000270202->ENSP00000348986->ENSP00000304895->ENSP00000339007->ENSP00000341189 4

2340.ENSP00000270202->ENSP00000417281->ENSP00000269305->ENSP00000293195->ENSP00000342087 36

2341.ENSP00000270202->ENSP00000417281->ENSP00000267163->ENSP00000345571 3

2342.ENSP00000270202->ENSP00000299421->ENSP00000228307->ENSP00000380227->ENSP00000346839 5

2343.ENSP00000270202->ENSP00000309103->ENSP00000329623->ENSP00000311032->ENSP00000347858 5

2344.ENSP00000270202->ENSP00000299421->ENSP00000228307->ENSP00000380227->ENSP00000364094->ENSP00000386896->ENSP00000200181->ENSP00000340937->ENSP00000348384 15

2345.ENSP00000270202->ENSP00000244741->ENSP00000367207->ENSP00000351490 5

2346.ENSP00000270202->ENSP00000352121 6

2347.ENSP00000270202->ENSP00000206249->ENSP00000327213->ENSP00000352900 17

2348.ENSP00000270202->ENSP00000417281->ENSP00000269305->ENSP00000353059 4

2349.ENSP00000270202->ENSP00000299421->ENSP00000228307->ENSP00000380227->ENSP00000364094->ENSP00000282588->ENSP00000364979->ENSP00000353654 18

2350.ENSP00000270202->ENSP00000228872->ENSP00000274026->ENSP00000306043->ENSP00000342307->ENSP00000230538->ENSP00000354360 16

2351.ENSP00000270202->ENSP00000417281->ENSP00000267163->ENSP00000355249 3

2352.ENSP00000270202->ENSP00000348461->ENSP00000264657->ENSP00000258743->ENSP00000356438 8

2353.ENSP00000270202->ENSP00000417281->ENSP00000344818->ENSP00000316840->ENSP00000358622 4

2354.ENSP00000270202->ENSP00000359424 5

2355.ENSP00000270202->ENSP00000361021 3

2356.ENSP00000270202->ENSP00000417281->ENSP00000269305->ENSP00000278916->ENSP00000321656->ENSP00000300093->ENSP00000263753->ENSP00000418447->ENSP00000348784->ENSP00000379866->ENSP00000379823->ENSP00000331902->ENSP00000361290 46

2357.ENSP00000270202->ENSP00000417281->ENSP00000344818->ENSP00000316840->ENSP00000361359->ENSP00000362994 5

2358.ENSP00000270202->ENSP00000206249->ENSP00000327213->ENSP00000363812 29

2359.ENSP00000270202->ENSP00000299421->ENSP00000228307->ENSP00000380227->ENSP00000364094 5

2360.ENSP00000270202->ENSP00000299421->ENSP00000228307->ENSP00000380227->ENSP00000364094->ENSP00000282588->ENSP00000364979 17

2361.ENSP00000270202->ENSP00000361021->ENSP00000366563 15

2362.ENSP00000270202->ENSP00000244741->ENSP00000367207 4

2363.ENSP00000270202->ENSP00000206249->ENSP00000254227->ENSP00000342470->ENSP00000363868->ENSP00000372703 122

2364.ENSP00000270202->ENSP00000228872->ENSP00000274026->ENSP00000306043->ENSP00000342307->ENSP00000230538->ENSP00000222399->ENSP00000374309 24

2365.ENSP00000270202->ENSP00000348986->ENSP00000375892 6

2366.ENSP00000270202->ENSP00000348461->ENSP00000264657->ENSP00000376765 6

2367.ENSP00000270202->ENSP00000417281->ENSP00000269305->ENSP00000278916->ENSP00000321656->ENSP00000300093->ENSP00000263753->ENSP00000418447->ENSP00000348784->ENSP00000379866 43

2368.ENSP00000270202->ENSP00000417281->ENSP00000269305->ENSP00000324897->ENSP00000381648 4

2369.ENSP00000270202->ENSP00000417281->ENSP00000269305->ENSP00000362649->ENSP00000384273 4

2370.ENSP00000270202->ENSP00000299421->ENSP00000228307->ENSP00000380227->ENSP00000364094->ENSP00000386896 6

2371.ENSP00000270202->ENSP00000206249->ENSP00000254227->ENSP00000342470->ENSP00000363868->ENSP00000393286 122

2372.ENSP00000270202->ENSP00000206249->ENSP00000254227->ENSP00000342470->ENSP00000363868->ENSP00000393870 122

2373.ENSP00000270202->ENSP00000417281->ENSP00000269305->ENSP00000329357->ENSP00000332973->ENSP00000287727->ENSP00000400104 105

2374.ENSP00000270202->ENSP00000297494->ENSP00000341940->ENSP00000312435->ENSP00000400365 15

2375.ENSP00000270202->ENSP00000206249->ENSP00000254227->ENSP00000342470->ENSP00000363868->ENSP00000402590 122

2376.ENSP00000270202->ENSP00000206249->ENSP00000399968->ENSP00000419692 4

2377.ENSP00000274255->ENSP00000228872->ENSP00000270202->ENSP00000274335 5

2378.ENSP00000274255->ENSP00000228872->ENSP00000257904->ENSP00000276925 3

2379.ENSP00000274255->ENSP00000228872->ENSP00000270202->ENSP00000289153 6

2380.ENSP00000274255->ENSP00000228872->ENSP00000270202->ENSP00000299421->ENSP00000228307->ENSP00000380227->ENSP00000364094->ENSP00000296585 8

2381.ENSP00000274255->ENSP00000228872->ENSP00000270202->ENSP00000309103->ENSP00000302564 4

2382.ENSP00000274255->ENSP00000228872->ENSP00000270202->ENSP00000348461->ENSP00000356505->ENSP00000220764->ENSP00000348380->ENSP00000305638 1020

2383.ENSP00000274255->ENSP00000228872->ENSP00000255465->ENSP00000306043->ENSP00000342307->ENSP00000230538->ENSP00000354360->ENSP00000252999->ENSP00000307156 26

2384.ENSP00000274255->ENSP00000266970->ENSP00000269305->ENSP00000353059->ENSP00000307786 11

2385.ENSP00000274255->ENSP00000266970->ENSP00000309181 3

2386.ENSP00000274255->ENSP00000311083 1

2387.ENSP00000274255->ENSP00000228872->ENSP00000270202->ENSP00000299421->ENSP00000228307->ENSP00000380227->ENSP00000364094->ENSP00000386896->ENSP00000200181->ENSP00000340937->ENSP00000348384->ENSP00000324532 18

2388.ENSP00000274255->ENSP00000326804->ENSP00000359206->ENSP00000216797->ENSP00000226574->ENSP00000327251 23

2389.ENSP00000274255->ENSP00000367207->ENSP00000329623 4

2390.ENSP00000274255->ENSP00000266970->ENSP00000269305->ENSP00000353059->ENSP00000330237 6

2391.ENSP00000274255->ENSP00000367207->ENSP00000263253->ENSP00000325690->ENSP00000399968->ENSP00000419692->ENSP00000332296 22

2392.ENSP00000274255->ENSP00000266970->ENSP00000269305->ENSP00000344818->ENSP00000316840->ENSP00000361359->ENSP00000332468 7

2393.ENSP00000274255->ENSP00000326804->ENSP00000359206->ENSP00000216797->ENSP00000339151 4

2394.ENSP00000274255->ENSP00000266970->ENSP00000269305->ENSP00000361021->ENSP00000341189 6

2395.ENSP00000274255->ENSP00000266970->ENSP00000269305->ENSP00000293195->ENSP00000342087 37

2396.ENSP00000274255->ENSP00000228872->ENSP00000227507->ENSP00000345571 3

2397.ENSP00000274255->ENSP00000228872->ENSP00000270202->ENSP00000299421->ENSP00000228307->ENSP00000380227->ENSP00000346839 7

2398.ENSP00000274255->ENSP00000266970->ENSP00000269305->ENSP00000355759->ENSP00000311032->ENSP00000347858 7

2399.ENSP00000274255->ENSP00000228872->ENSP00000270202->ENSP00000299421->ENSP00000228307->ENSP00000380227->ENSP00000364094->ENSP00000386896->ENSP00000200181->ENSP00000340937->ENSP00000348384 17

2400.ENSP00000274255->ENSP00000367207->ENSP00000351490 3

2401.ENSP00000274255->ENSP00000228872->ENSP00000270202->ENSP00000352121 8

2402.ENSP00000274255->ENSP00000367207->ENSP00000206249->ENSP00000327213->ENSP00000352900 19

2403.ENSP00000274255->ENSP00000266970->ENSP00000269305->ENSP00000353059 5

2404.ENSP00000274255->ENSP00000228872->ENSP00000270202->ENSP00000299421->ENSP00000228307->ENSP00000380227->ENSP00000364094->ENSP00000282588->ENSP00000364979->ENSP00000353654 20

2405.ENSP00000274255->ENSP00000228872->ENSP00000255465->ENSP00000306043->ENSP00000342307->ENSP00000230538->ENSP00000354360 16

2406.ENSP00000274255->ENSP00000266970->ENSP00000267163->ENSP00000355249 4

2407.ENSP00000274255->ENSP00000228872->ENSP00000227507->ENSP00000264657->ENSP00000258743->ENSP00000356438 9

2408.ENSP00000274255->ENSP00000326804->ENSP00000359206->ENSP00000216797->ENSP00000358622 4

2409.ENSP00000274255->ENSP00000326804->ENSP00000359206->ENSP00000189444->ENSP00000359424 4

2410.ENSP00000274255->ENSP00000266970->ENSP00000269305->ENSP00000361021 4

2411.ENSP00000274255->ENSP00000228872->ENSP00000255465->ENSP00000306043->ENSP00000256442->ENSP00000300093->ENSP00000263753->ENSP00000418447->ENSP00000348784->ENSP00000379866->ENSP00000379823->ENSP00000331902->ENSP00000361290 46

2412.ENSP00000274255->ENSP00000326804->ENSP00000359206->ENSP00000216797->ENSP00000226574->ENSP00000362994 7

2413.ENSP00000274255->ENSP00000367207->ENSP00000206249->ENSP00000327213->ENSP00000363812 31

2414.ENSP00000274255->ENSP00000228872->ENSP00000270202->ENSP00000299421->ENSP00000228307->ENSP00000380227->ENSP00000364094 7

2415.ENSP00000274255->ENSP00000228872->ENSP00000270202->ENSP00000299421->ENSP00000228307->ENSP00000380227->ENSP00000364094->ENSP00000282588->ENSP00000364979 19

2416.ENSP00000274255->ENSP00000266970->ENSP00000269305->ENSP00000361021->ENSP00000366563 16

2417.ENSP00000274255->ENSP00000367207 2

2418.ENSP00000274255->ENSP00000367207->ENSP00000206249->ENSP00000254227->ENSP00000342470->ENSP00000363868->ENSP00000372703 124

2419.ENSP00000274255->ENSP00000228872->ENSP00000255465->ENSP00000306043->ENSP00000342307->ENSP00000230538->ENSP00000222399->ENSP00000374309 24

2420.ENSP00000274255->ENSP00000228872->ENSP00000270202->ENSP00000348986->ENSP00000375892 8

2421.ENSP00000274255->ENSP00000228872->ENSP00000227507->ENSP00000264657->ENSP00000376765 7

2422.ENSP00000274255->ENSP00000228872->ENSP00000255465->ENSP00000306043->ENSP00000256442->ENSP00000300093->ENSP00000263753->ENSP00000418447->ENSP00000348784->ENSP00000379866 43

2423.ENSP00000274255->ENSP00000266970->ENSP00000269305->ENSP00000324897->ENSP00000381648 5

2424.ENSP00000274255->ENSP00000367207->ENSP00000263253->ENSP00000384273 4

2425.ENSP00000274255->ENSP00000228872->ENSP00000270202->ENSP00000299421->ENSP00000228307->ENSP00000380227->ENSP00000364094->ENSP00000386896 8

2426.ENSP00000274255->ENSP00000367207->ENSP00000206249->ENSP00000254227->ENSP00000342470->ENSP00000363868->ENSP00000393286 124

2427.ENSP00000274255->ENSP00000367207->ENSP00000206249->ENSP00000254227->ENSP00000342470->ENSP00000363868->ENSP00000393870 124

2428.ENSP00000274255->ENSP00000367207->ENSP00000329357->ENSP00000332973->ENSP00000287727->ENSP00000400104 105

2429.ENSP00000274255->ENSP00000228872->ENSP00000255465->ENSP00000306043->ENSP00000342307->ENSP00000230538->ENSP00000354360->ENSP00000400365 17

2430.ENSP00000274255->ENSP00000367207->ENSP00000206249->ENSP00000254227->ENSP00000342470->ENSP00000363868->ENSP00000402590 124

2431.ENSP00000274255->ENSP00000367207->ENSP00000263253->ENSP00000325690->ENSP00000399968->ENSP00000419692 6

2432.ENSP00000274335->ENSP00000270202->ENSP00000228872->ENSP00000257904->ENSP00000276925 6

2433.ENSP00000274335->ENSP00000289153 4

2434.ENSP00000274335->ENSP00000339007->ENSP00000341189->ENSP00000228307->ENSP00000380227->ENSP00000364094->ENSP00000296585 6

2435.ENSP00000274335->ENSP00000304895->ENSP00000353483->ENSP00000269305->ENSP00000302564 5

2436.ENSP00000274335->ENSP00000339007->ENSP00000302269->ENSP00000348461->ENSP00000356505->ENSP00000220764->ENSP00000348380->ENSP00000305638 1020

2437.ENSP00000274335->ENSP00000339007->ENSP00000312435->ENSP00000400365->ENSP00000354360->ENSP00000252999->ENSP00000307156 26

2438.ENSP00000274335->ENSP00000304895->ENSP00000353483->ENSP00000269305->ENSP00000353059->ENSP00000307786 11

2439.ENSP00000274335->ENSP00000304895->ENSP00000353483->ENSP00000269305->ENSP00000266970->ENSP00000309181 5

2440.ENSP00000274335->ENSP00000304895->ENSP00000353483->ENSP00000269305->ENSP00000266970->ENSP00000311083 5

2441.ENSP00000274335->ENSP00000339007->ENSP00000341189->ENSP00000228307->ENSP00000380227->ENSP00000364094->ENSP00000386896->ENSP00000200181->ENSP00000340937->ENSP00000348384->ENSP00000324532 16

2442.ENSP00000274335->ENSP00000344456->ENSP00000359206->ENSP00000216797->ENSP00000226574->ENSP00000327251 25

2443.ENSP00000274335->ENSP00000304895->ENSP00000353483->ENSP00000269305->ENSP00000329623 4

2444.ENSP00000274335->ENSP00000304895->ENSP00000353483->ENSP00000269305->ENSP00000353059->ENSP00000330237 6

2445.ENSP00000274335->ENSP00000268035->ENSP00000206249->ENSP00000399968->ENSP00000419692->ENSP00000332296 21

2446.ENSP00000274335->ENSP00000401303->ENSP00000275493->ENSP00000344818->ENSP00000316840->ENSP00000361359->ENSP00000332468 6

2447.ENSP00000274335->ENSP00000344456->ENSP00000359206->ENSP00000216797->ENSP00000339151 6

2448.ENSP00000274335->ENSP00000339007->ENSP00000341189 2

2449.ENSP00000274335->ENSP00000304895->ENSP00000353483->ENSP00000269305->ENSP00000293195->ENSP00000342087 37

2450.ENSP00000274335->ENSP00000344456->ENSP00000227507->ENSP00000345571 5

2451.ENSP00000274335->ENSP00000339007->ENSP00000341189->ENSP00000228307->ENSP00000380227->ENSP00000346839 5

2452.ENSP00000274335->ENSP00000401303->ENSP00000275493->ENSP00000344818->ENSP00000316840->ENSP00000216160->ENSP00000347858 6

2453.ENSP00000274335->ENSP00000339007->ENSP00000341189->ENSP00000228307->ENSP00000380227->ENSP00000364094->ENSP00000386896->ENSP00000200181->ENSP00000340937->ENSP00000348384 15

2454.ENSP00000274335->ENSP00000339007->ENSP00000215832->ENSP00000367207->ENSP00000351490 5

2455.ENSP00000274335->ENSP00000263967->ENSP00000309845->ENSP00000352121 9

2456.ENSP00000274335->ENSP00000268035->ENSP00000206249->ENSP00000327213->ENSP00000352900 18

2457.ENSP00000274335->ENSP00000304895->ENSP00000353483->ENSP00000269305->ENSP00000353059 5

2458.ENSP00000274335->ENSP00000339007->ENSP00000341189->ENSP00000228307->ENSP00000380227->ENSP00000364094->ENSP00000282588->ENSP00000364979->ENSP00000353654 18

2459.ENSP00000274335->ENSP00000339007->ENSP00000312435->ENSP00000400365->ENSP00000354360 16

2460.ENSP00000274335->ENSP00000304895->ENSP00000353483->ENSP00000269305->ENSP00000267163->ENSP00000355249 5

2461.ENSP00000274335->ENSP00000304895->ENSP00000353483->ENSP00000360266->ENSP00000356438 8

2462.ENSP00000274335->ENSP00000401303->ENSP00000275493->ENSP00000344818->ENSP00000316840->ENSP00000358622 5

2463.ENSP00000274335->ENSP00000344456->ENSP00000359206->ENSP00000216797->ENSP00000359424 6

2464.ENSP00000274335->ENSP00000263967->ENSP00000361021 3

2465.ENSP00000274335->ENSP00000304895->ENSP00000353483->ENSP00000269305->ENSP00000278916->ENSP00000312995->ENSP00000300093->ENSP00000263753->ENSP00000418447->ENSP00000348784->ENSP00000379866->ENSP00000379823->ENSP00000331902->ENSP00000361290 47

2466.ENSP00000274335->ENSP00000401303->ENSP00000275493->ENSP00000344818->ENSP00000316840->ENSP00000361359->ENSP00000362994 6

2467.ENSP00000274335->ENSP00000268035->ENSP00000206249->ENSP00000327213->ENSP00000363812 30

2468.ENSP00000274335->ENSP00000339007->ENSP00000341189->ENSP00000228307->ENSP00000380227->ENSP00000364094 5

2469.ENSP00000274335->ENSP00000339007->ENSP00000341189->ENSP00000228307->ENSP00000380227->ENSP00000364094->ENSP00000282588->ENSP00000364979 17

2470.ENSP00000274335->ENSP00000263967->ENSP00000361021->ENSP00000366563 15

2471.ENSP00000274335->ENSP00000339007->ENSP00000215832->ENSP00000367207 4

2472.ENSP00000274335->ENSP00000268035->ENSP00000206249->ENSP00000254227->ENSP00000342470->ENSP00000363868->ENSP00000372703 123

2473.ENSP00000274335->ENSP00000339007->ENSP00000312435->ENSP00000400365->ENSP00000222399->ENSP00000374309 25

2474.ENSP00000274335->ENSP00000365016->ENSP00000348986->ENSP00000375892 7

2475.ENSP00000274335->ENSP00000401303->ENSP00000275493->ENSP00000264657->ENSP00000376765 6

2476.ENSP00000274335->ENSP00000304895->ENSP00000353483->ENSP00000269305->ENSP00000278916->ENSP00000312995->ENSP00000300093->ENSP00000263753->ENSP00000418447->ENSP00000348784->ENSP00000379866 44

2477.ENSP00000274335->ENSP00000304895->ENSP00000353483->ENSP00000269305->ENSP00000324897->ENSP00000381648 5

2478.ENSP00000274335->ENSP00000304895->ENSP00000353483->ENSP00000269305->ENSP00000262367->ENSP00000384273 5

2479.ENSP00000274335->ENSP00000339007->ENSP00000341189->ENSP00000228307->ENSP00000380227->ENSP00000364094->ENSP00000386896 6

2480.ENSP00000274335->ENSP00000268035->ENSP00000206249->ENSP00000254227->ENSP00000342470->ENSP00000363868->ENSP00000393286 123

2481.ENSP00000274335->ENSP00000268035->ENSP00000206249->ENSP00000254227->ENSP00000342470->ENSP00000363868->ENSP00000393870 123

2482.ENSP00000274335->ENSP00000304895->ENSP00000353483->ENSP00000269305->ENSP00000263253->ENSP00000262160->ENSP00000287727->ENSP00000400104 106

2483.ENSP00000274335->ENSP00000339007->ENSP00000312435->ENSP00000400365 15

2484.ENSP00000274335->ENSP00000268035->ENSP00000206249->ENSP00000254227->ENSP00000342470->ENSP00000363868->ENSP00000402590 123

2485.ENSP00000274335->ENSP00000268035->ENSP00000206249->ENSP00000399968->ENSP00000419692 5

2486.ENSP00000276925->ENSP00000257904->ENSP00000228872->ENSP00000270202->ENSP00000289153 7

2487.ENSP00000276925->ENSP00000257904->ENSP00000228872->ENSP00000270202->ENSP00000299421->ENSP00000228307->ENSP00000380227->ENSP00000364094->ENSP00000296585 9

2488.ENSP00000276925->ENSP00000257904->ENSP00000355153->ENSP00000269305->ENSP00000302564 5

2489.ENSP00000276925->ENSP00000257904->ENSP00000228872->ENSP00000270202->ENSP00000348461->ENSP00000356505->ENSP00000220764->ENSP00000348380->ENSP00000305638 1021

2490.ENSP00000276925->ENSP00000257904->ENSP00000244741->ENSP00000274026->ENSP00000306043->ENSP00000342307->ENSP00000230538->ENSP00000354360->ENSP00000252999->ENSP00000307156 27

2491.ENSP00000276925->ENSP00000257904->ENSP00000355153->ENSP00000269305->ENSP00000353059->ENSP00000307786 11

2492.ENSP00000276925->ENSP00000257904->ENSP00000267163->ENSP00000266970->ENSP00000309181 4

2493.ENSP00000276925->ENSP00000257904->ENSP00000267163->ENSP00000266970->ENSP00000311083 4

2494.ENSP00000276925->ENSP00000257904->ENSP00000228872->ENSP00000270202->ENSP00000299421->ENSP00000228307->ENSP00000380227->ENSP00000364094->ENSP00000386896->ENSP00000200181->ENSP00000340937->ENSP00000348384->ENSP00000324532 19

2495.ENSP00000276925->ENSP00000257904->ENSP00000267163->ENSP00000362649->ENSP00000384273->ENSP00000226574->ENSP00000327251 24

2496.ENSP00000276925->ENSP00000257904->ENSP00000355153->ENSP00000269305->ENSP00000329623 4

2497.ENSP00000276925->ENSP00000257904->ENSP00000355153->ENSP00000269305->ENSP00000353059->ENSP00000330237 6

2498.ENSP00000276925->ENSP00000257904->ENSP00000227507->ENSP00000344456->ENSP00000363822->ENSP00000399968->ENSP00000419692->ENSP00000332296 22

2499.ENSP00000276925->ENSP00000257904->ENSP00000244741->ENSP00000344818->ENSP00000316840->ENSP00000361359->ENSP00000332468 7

2500.ENSP00000276925->ENSP00000257904->ENSP00000267163->ENSP00000362649->ENSP00000384273->ENSP00000339151 5

2501.ENSP00000276925->ENSP00000257904->ENSP00000355153->ENSP00000269305->ENSP00000361021->ENSP00000341189 6

2502.ENSP00000276925->ENSP00000257904->ENSP00000355153->ENSP00000269305->ENSP00000293195->ENSP00000342087 37

2503.ENSP00000276925->ENSP00000257904->ENSP00000267163->ENSP00000345571 3

2504.ENSP00000276925->ENSP00000257904->ENSP00000228872->ENSP00000270202->ENSP00000299421->ENSP00000228307->ENSP00000380227->ENSP00000346839 8

2505.ENSP00000276925->ENSP00000257904->ENSP00000355153->ENSP00000269305->ENSP00000329623->ENSP00000311032->ENSP00000347858 7

2506.ENSP00000276925->ENSP00000257904->ENSP00000228872->ENSP00000270202->ENSP00000299421->ENSP00000228307->ENSP00000380227->ENSP00000364094->ENSP00000386896->ENSP00000200181->ENSP00000340937->ENSP00000348384 18

2507.ENSP00000276925->ENSP00000257904->ENSP00000367207->ENSP00000351490 4

2508.ENSP00000276925->ENSP00000257904->ENSP00000228872->ENSP00000270202->ENSP00000352121 9

2509.ENSP00000276925->ENSP00000257904->ENSP00000227507->ENSP00000206249->ENSP00000327213->ENSP00000352900 19

2510.ENSP00000276925->ENSP00000257904->ENSP00000355153->ENSP00000269305->ENSP00000353059 5

2511.ENSP00000276925->ENSP00000257904->ENSP00000228872->ENSP00000270202->ENSP00000299421->ENSP00000228307->ENSP00000380227->ENSP00000364094->ENSP00000282588->ENSP00000364979->ENSP00000353654 21

2512.ENSP00000276925->ENSP00000257904->ENSP00000244741->ENSP00000274026->ENSP00000306043->ENSP00000342307->ENSP00000230538->ENSP00000354360 17

2513.ENSP00000276925->ENSP00000257904->ENSP00000267163->ENSP00000355249 3

2514.ENSP00000276925->ENSP00000257904->ENSP00000227507->ENSP00000264657->ENSP00000258743->ENSP00000356438 9

2515.ENSP00000276925->ENSP00000257904->ENSP00000227507->ENSP00000344456->ENSP00000359206->ENSP00000216797->ENSP00000358622 6

2516.ENSP00000276925->ENSP00000257904->ENSP00000267163->ENSP00000362649->ENSP00000384273->ENSP00000359424 5

2517.ENSP00000276925->ENSP00000257904->ENSP00000355153->ENSP00000269305->ENSP00000361021 4

2518.ENSP00000276925->ENSP00000257904->ENSP00000355153->ENSP00000269305->ENSP00000361275->ENSP00000321656->ENSP00000300093->ENSP00000263753->ENSP00000418447->ENSP00000348784->ENSP00000379866->ENSP00000379823->ENSP00000331902->ENSP00000361290 47

2519.ENSP00000276925->ENSP00000257904->ENSP00000244741->ENSP00000344818->ENSP00000316840->ENSP00000361359->ENSP00000362994 7

2520.ENSP00000276925->ENSP00000257904->ENSP00000227507->ENSP00000206249->ENSP00000327213->ENSP00000363812 31

2521.ENSP00000276925->ENSP00000257904->ENSP00000228872->ENSP00000270202->ENSP00000299421->ENSP00000228307->ENSP00000380227->ENSP00000364094 8

2522.ENSP00000276925->ENSP00000257904->ENSP00000228872->ENSP00000270202->ENSP00000299421->ENSP00000228307->ENSP00000380227->ENSP00000364094->ENSP00000282588->ENSP00000364979 20

2523.ENSP00000276925->ENSP00000257904->ENSP00000355153->ENSP00000269305->ENSP00000361021->ENSP00000366563 16

2524.ENSP00000276925->ENSP00000257904->ENSP00000367207 3

2525.ENSP00000276925->ENSP00000257904->ENSP00000227507->ENSP00000344456->ENSP00000326366->ENSP00000284981->ENSP00000252486->ENSP00000252444->ENSP00000233242->ENSP00000236850->ENSP00000363868->ENSP00000372703 124

2526.ENSP00000276925->ENSP00000257904->ENSP00000244741->ENSP00000274026->ENSP00000306043->ENSP00000342307->ENSP00000230538->ENSP00000222399->ENSP00000374309 25

2527.ENSP00000276925->ENSP00000257904->ENSP00000228872->ENSP00000270202->ENSP00000348986->ENSP00000375892 9

2528.ENSP00000276925->ENSP00000257904->ENSP00000227507->ENSP00000264657->ENSP00000376765 7

2529.ENSP00000276925->ENSP00000257904->ENSP00000355153->ENSP00000269305->ENSP00000361275->ENSP00000321656->ENSP00000300093->ENSP00000263753->ENSP00000418447->ENSP00000348784->ENSP00000379866 44

2530.ENSP00000276925->ENSP00000257904->ENSP00000355153->ENSP00000269305->ENSP00000324897->ENSP00000381648 5

2531.ENSP00000276925->ENSP00000257904->ENSP00000267163->ENSP00000362649->ENSP00000384273 4

2532.ENSP00000276925->ENSP00000257904->ENSP00000228872->ENSP00000270202->ENSP00000299421->ENSP00000228307->ENSP00000380227->ENSP00000364094->ENSP00000386896 9

2533.ENSP00000276925->ENSP00000257904->ENSP00000227507->ENSP00000344456->ENSP00000326366->ENSP00000284981->ENSP00000252486->ENSP00000252444->ENSP00000233242->ENSP00000236850->ENSP00000363868->ENSP00000393286 124

2534.ENSP00000276925->ENSP00000257904->ENSP00000227507->ENSP00000344456->ENSP00000326366->ENSP00000284981->ENSP00000252486->ENSP00000252444->ENSP00000233242->ENSP00000236850->ENSP00000363868->ENSP00000393870 124

2535.ENSP00000276925->ENSP00000257904->ENSP00000367207->ENSP00000329357->ENSP00000332973->ENSP00000287727->ENSP00000400104 106

2536.ENSP00000276925->ENSP00000257904->ENSP00000244741->ENSP00000274026->ENSP00000306043->ENSP00000342307->ENSP00000230538->ENSP00000354360->ENSP00000400365 18

2537.ENSP00000276925->ENSP00000257904->ENSP00000227507->ENSP00000344456->ENSP00000326366->ENSP00000284981->ENSP00000252486->ENSP00000252444->ENSP00000233242->ENSP00000236850->ENSP00000363868->ENSP00000402590 124

2538.ENSP00000276925->ENSP00000257904->ENSP00000227507->ENSP00000344456->ENSP00000363822->ENSP00000399968->ENSP00000419692 6

2539.ENSP00000289153->ENSP00000270202->ENSP00000299421->ENSP00000228307->ENSP00000380227->ENSP00000364094->ENSP00000296585 10

2540.ENSP00000289153->ENSP00000270202->ENSP00000309103->ENSP00000302564 6

2541.ENSP00000289153->ENSP00000270202->ENSP00000348461->ENSP00000356505->ENSP00000220764->ENSP00000348380->ENSP00000305638 1022

2542.ENSP00000289153->ENSP00000270202->ENSP00000228872->ENSP00000274026->ENSP00000306043->ENSP00000342307->ENSP00000230538->ENSP00000354360->ENSP00000252999->ENSP00000307156 30

2543.ENSP00000289153->ENSP00000270202->ENSP00000417281->ENSP00000269305->ENSP00000353059->ENSP00000307786 14

2544.ENSP00000289153->ENSP00000270202->ENSP00000228872->ENSP00000266970->ENSP00000309181 7

2545.ENSP00000289153->ENSP00000270202->ENSP00000228872->ENSP00000266970->ENSP00000311083 7

2546.ENSP00000289153->ENSP00000270202->ENSP00000299421->ENSP00000228307->ENSP00000380227->ENSP00000364094->ENSP00000386896->ENSP00000200181->ENSP00000340937->ENSP00000348384->ENSP00000324532 20

2547.ENSP00000289153->ENSP00000270202->ENSP00000335153->ENSP00000231509->ENSP00000226574->ENSP00000327251 28

2548.ENSP00000289153->ENSP00000270202->ENSP00000309103->ENSP00000329623 6

2549.ENSP00000289153->ENSP00000270202->ENSP00000417281->ENSP00000269305->ENSP00000353059->ENSP00000330237 9

2550.ENSP00000289153->ENSP00000270202->ENSP00000206249->ENSP00000399968->ENSP00000419692->ENSP00000332296 24

2551.ENSP00000289153->ENSP00000270202->ENSP00000417281->ENSP00000344818->ENSP00000316840->ENSP00000361359->ENSP00000332468 9

2552.ENSP00000289153->ENSP00000270202->ENSP00000298552->ENSP00000339151 8

2553.ENSP00000289153->ENSP00000274335->ENSP00000339007->ENSP00000341189 6

2554.ENSP00000289153->ENSP00000270202->ENSP00000417281->ENSP00000269305->ENSP00000293195->ENSP00000342087 40

2555.ENSP00000289153->ENSP00000270202->ENSP00000417281->ENSP00000267163->ENSP00000345571 7

2556.ENSP00000289153->ENSP00000270202->ENSP00000299421->ENSP00000228307->ENSP00000380227->ENSP00000346839 9

2557.ENSP00000289153->ENSP00000270202->ENSP00000309103->ENSP00000329623->ENSP00000311032->ENSP00000347858 9

2558.ENSP00000289153->ENSP00000270202->ENSP00000299421->ENSP00000228307->ENSP00000380227->ENSP00000364094->ENSP00000386896->ENSP00000200181->ENSP00000340937->ENSP00000348384 19

2559.ENSP00000289153->ENSP00000270202->ENSP00000206249->ENSP00000367207->ENSP00000351490 9

2560.ENSP00000289153->ENSP00000270202->ENSP00000352121 10

2561.ENSP00000289153->ENSP00000270202->ENSP00000206249->ENSP00000327213->ENSP00000352900 21

2562.ENSP00000289153->ENSP00000270202->ENSP00000417281->ENSP00000269305->ENSP00000353059 8

2563.ENSP00000289153->ENSP00000270202->ENSP00000299421->ENSP00000228307->ENSP00000380227->ENSP00000364094->ENSP00000282588->ENSP00000364979->ENSP00000353654 22

2564.ENSP00000289153->ENSP00000270202->ENSP00000228872->ENSP00000274026->ENSP00000306043->ENSP00000342307->ENSP00000230538->ENSP00000354360 20

2565.ENSP00000289153->ENSP00000270202->ENSP00000417281->ENSP00000267163->ENSP00000355249 7

2566.ENSP00000289153->ENSP00000274335->ENSP00000304895->ENSP00000353483->ENSP00000360266->ENSP00000356438 12

2567.ENSP00000289153->ENSP00000270202->ENSP00000417281->ENSP00000344818->ENSP00000316840->ENSP00000358622 8

2568.ENSP00000289153->ENSP00000270202->ENSP00000359424 9

2569.ENSP00000289153->ENSP00000270202->ENSP00000361021 7

2570.ENSP00000289153->ENSP00000270202->ENSP00000417281->ENSP00000269305->ENSP00000278916->ENSP00000312995->ENSP00000300093->ENSP00000263753->ENSP00000418447->ENSP00000348784->ENSP00000379866->ENSP00000379823->ENSP00000331902->ENSP00000361290 50

2571.ENSP00000289153->ENSP00000270202->ENSP00000417281->ENSP00000344818->ENSP00000316840->ENSP00000361359->ENSP00000362994 9

2572.ENSP00000289153->ENSP00000270202->ENSP00000206249->ENSP00000327213->ENSP00000363812 33

2573.ENSP00000289153->ENSP00000270202->ENSP00000299421->ENSP00000228307->ENSP00000380227->ENSP00000364094 9

2574.ENSP00000289153->ENSP00000270202->ENSP00000299421->ENSP00000228307->ENSP00000380227->ENSP00000364094->ENSP00000282588->ENSP00000364979 21

2575.ENSP00000289153->ENSP00000270202->ENSP00000361021->ENSP00000366563 19

2576.ENSP00000289153->ENSP00000270202->ENSP00000206249->ENSP00000367207 8

2577.ENSP00000289153->ENSP00000270202->ENSP00000206249->ENSP00000254227->ENSP00000342470->ENSP00000363868->ENSP00000372703 126

2578.ENSP00000289153->ENSP00000270202->ENSP00000228872->ENSP00000274026->ENSP00000306043->ENSP00000342307->ENSP00000230538->ENSP00000222399->ENSP00000374309 28

2579.ENSP00000289153->ENSP00000270202->ENSP00000348986->ENSP00000375892 10

2580.ENSP00000289153->ENSP00000270202->ENSP00000348461->ENSP00000264657->ENSP00000376765 10

2581.ENSP00000289153->ENSP00000270202->ENSP00000417281->ENSP00000269305->ENSP00000278916->ENSP00000312995->ENSP00000300093->ENSP00000263753->ENSP00000418447->ENSP00000348784->ENSP00000379866 47

2582.ENSP00000289153->ENSP00000270202->ENSP00000417281->ENSP00000269305->ENSP00000324897->ENSP00000381648 8

2583.ENSP00000289153->ENSP00000270202->ENSP00000206249->ENSP00000263253->ENSP00000384273 8

2584.ENSP00000289153->ENSP00000270202->ENSP00000299421->ENSP00000228307->ENSP00000380227->ENSP00000364094->ENSP00000386896 10

2585.ENSP00000289153->ENSP00000270202->ENSP00000206249->ENSP00000254227->ENSP00000342470->ENSP00000363868->ENSP00000393286 126

2586.ENSP00000289153->ENSP00000270202->ENSP00000206249->ENSP00000254227->ENSP00000342470->ENSP00000363868->ENSP00000393870 126

2587.ENSP00000289153->ENSP00000270202->ENSP00000354558->ENSP00000371138->ENSP00000364133->ENSP00000262160->ENSP00000287727->ENSP00000400104 109

2588.ENSP00000289153->ENSP00000274335->ENSP00000339007->ENSP00000312435->ENSP00000400365 19

2589.ENSP00000289153->ENSP00000270202->ENSP00000206249->ENSP00000254227->ENSP00000342470->ENSP00000363868->ENSP00000402590 126

2590.ENSP00000289153->ENSP00000270202->ENSP00000206249->ENSP00000399968->ENSP00000419692 8

2591.ENSP00000296585->ENSP00000364094->ENSP00000380227->ENSP00000228307->ENSP00000299421->ENSP00000270202->ENSP00000309103->ENSP00000302564 8

2592.ENSP00000296585->ENSP00000364094->ENSP00000380227->ENSP00000228307->ENSP00000300574->ENSP00000381107->ENSP00000348461->ENSP00000356505->ENSP00000220764->ENSP00000348380->ENSP00000305638 1023

2593.ENSP00000296585->ENSP00000364094->ENSP00000386896->ENSP00000200181->ENSP00000340937->ENSP00000264144->ENSP00000252999->ENSP00000307156 24

2594.ENSP00000296585->ENSP00000364094->ENSP00000380227->ENSP00000228307->ENSP00000341189->ENSP00000361021->ENSP00000269305->ENSP00000353059->ENSP00000307786 15

2595.ENSP00000296585->ENSP00000364094->ENSP00000380227->ENSP00000228307->ENSP00000299421->ENSP00000270202->ENSP00000228872->ENSP00000266970->ENSP00000309181 9

2596.ENSP00000296585->ENSP00000364094->ENSP00000380227->ENSP00000228307->ENSP00000299421->ENSP00000270202->ENSP00000228872->ENSP00000274255->ENSP00000311083 9

2597.ENSP00000296585->ENSP00000364094->ENSP00000386896->ENSP00000200181->ENSP00000340937->ENSP00000348384->ENSP00000324532 12

2598.ENSP00000296585->ENSP00000364094->ENSP00000367316->ENSP00000303242->ENSP00000264832->ENSP00000226574->ENSP00000327251 28

2599.ENSP00000296585->ENSP00000364094->ENSP00000380227->ENSP00000228307->ENSP00000299421->ENSP00000270202->ENSP00000309103->ENSP00000329623 8

2600.ENSP00000296585->ENSP00000364094->ENSP00000380227->ENSP00000228307->ENSP00000341189->ENSP00000361021->ENSP00000269305->ENSP00000353059->ENSP00000330237 10

2601.ENSP00000296585->ENSP00000364094->ENSP00000380227->ENSP00000228307->ENSP00000341189->ENSP00000350941->ENSP00000206249->ENSP00000399968->ENSP00000419692->ENSP00000332296 24

2602.ENSP00000296585->ENSP00000364094->ENSP00000380227->ENSP00000228307->ENSP00000300574->ENSP00000275493->ENSP00000344818->ENSP00000316840->ENSP00000361359->ENSP00000332468 10

2603.ENSP00000296585->ENSP00000364094->ENSP00000380227->ENSP00000228307->ENSP00000341189->ENSP00000350941->ENSP00000206249->ENSP00000263253->ENSP00000384273->ENSP00000339151 9

2604.ENSP00000296585->ENSP00000364094->ENSP00000380227->ENSP00000228307->ENSP00000341189 4

2605.ENSP00000296585->ENSP00000364094->ENSP00000380227->ENSP00000228307->ENSP00000341189->ENSP00000361021->ENSP00000269305->ENSP00000293195->ENSP00000342087 41

2606.ENSP00000296585->ENSP00000364094->ENSP00000380227->ENSP00000228307->ENSP00000341189->ENSP00000350941->ENSP00000206249->ENSP00000329357->ENSP00000345571 8

2607.ENSP00000296585->ENSP00000364094->ENSP00000346839 2

2608.ENSP00000296585->ENSP00000364094->ENSP00000380227->ENSP00000228307->ENSP00000300574->ENSP00000275493->ENSP00000344818->ENSP00000316840->ENSP00000216160->ENSP00000347858 10

2609.ENSP00000296585->ENSP00000364094->ENSP00000386896->ENSP00000200181->ENSP00000340937->ENSP00000348384 11

2610.ENSP00000296585->ENSP00000364094->ENSP00000380227->ENSP00000228307->ENSP00000341189->ENSP00000350941->ENSP00000206249->ENSP00000367207->ENSP00000351490 9

2611.ENSP00000296585->ENSP00000364094->ENSP00000380227->ENSP00000228307->ENSP00000299421->ENSP00000270202->ENSP00000352121 12

2612.ENSP00000296585->ENSP00000364094->ENSP00000380227->ENSP00000228307->ENSP00000341189->ENSP00000350941->ENSP00000206249->ENSP00000327213->ENSP00000352900 21

2613.ENSP00000296585->ENSP00000364094->ENSP00000380227->ENSP00000228307->ENSP00000341189->ENSP00000361021->ENSP00000269305->ENSP00000353059 9

2614.ENSP00000296585->ENSP00000364094->ENSP00000282588->ENSP00000364979->ENSP00000353654 14

2615.ENSP00000296585->ENSP00000364094->ENSP00000380227->ENSP00000228307->ENSP00000341189->ENSP00000339007->ENSP00000312435->ENSP00000400365->ENSP00000354360 20

2616.ENSP00000296585->ENSP00000364094->ENSP00000380227->ENSP00000228307->ENSP00000299421->ENSP00000270202->ENSP00000417281->ENSP00000267163->ENSP00000355249 9

2617.ENSP00000296585->ENSP00000364094->ENSP00000380227->ENSP00000228307->ENSP00000341189->ENSP00000350941->ENSP00000264657->ENSP00000258743->ENSP00000356438 11

2618.ENSP00000296585->ENSP00000364094->ENSP00000380227->ENSP00000228307->ENSP00000300574->ENSP00000275493->ENSP00000344818->ENSP00000316840->ENSP00000358622 9

2619.ENSP00000296585->ENSP00000364094->ENSP00000380227->ENSP00000228307->ENSP00000341189->ENSP00000350941->ENSP00000206249->ENSP00000263253->ENSP00000384273->ENSP00000359424 9

2620.ENSP00000296585->ENSP00000364094->ENSP00000380227->ENSP00000228307->ENSP00000341189->ENSP00000361021 6

2621.ENSP00000296585->ENSP00000364094->ENSP00000380227->ENSP00000228307->ENSP00000341189->ENSP00000350941->ENSP00000206249->ENSP00000350283->ENSP00000372023->ENSP00000321656->ENSP00000300093->ENSP00000263753->ENSP00000418447->ENSP00000348784->ENSP00000379866->ENSP00000379823->ENSP00000331902->ENSP00000361290 51

2622.ENSP00000296585->ENSP00000364094->ENSP00000380227->ENSP00000228307->ENSP00000300574->ENSP00000275493->ENSP00000344818->ENSP00000316840->ENSP00000361359->ENSP00000362994 10

2623.ENSP00000296585->ENSP00000364094->ENSP00000380227->ENSP00000228307->ENSP00000341189->ENSP00000350941->ENSP00000206249->ENSP00000327213->ENSP00000363812 33

2624.ENSP00000296585->ENSP00000364094 1

2625.ENSP00000296585->ENSP00000364094->ENSP00000282588->ENSP00000364979 13

2626.ENSP00000296585->ENSP00000364094->ENSP00000380227->ENSP00000228307->ENSP00000341189->ENSP00000361021->ENSP00000366563 18

2627.ENSP00000296585->ENSP00000364094->ENSP00000380227->ENSP00000228307->ENSP00000341189->ENSP00000350941->ENSP00000206249->ENSP00000367207 8

2628.ENSP00000296585->ENSP00000364094->ENSP00000380227->ENSP00000228307->ENSP00000341189->ENSP00000350941->ENSP00000206249->ENSP00000254227->ENSP00000342470->ENSP00000363868->ENSP00000372703 126

2629.ENSP00000296585->ENSP00000364094->ENSP00000386896->ENSP00000200181->ENSP00000340937->ENSP00000264144->ENSP00000252999->ENSP00000258341->ENSP00000374309 26

2630.ENSP00000296585->ENSP00000364094->ENSP00000380227->ENSP00000228307->ENSP00000341189->ENSP00000339007->ENSP00000304895->ENSP00000348986->ENSP00000375892 12

2631.ENSP00000296585->ENSP00000364094->ENSP00000380227->ENSP00000228307->ENSP00000341189->ENSP00000350941->ENSP00000264657->ENSP00000376765 9

2632.ENSP00000296585->ENSP00000364094->ENSP00000380227->ENSP00000228307->ENSP00000341189->ENSP00000350941->ENSP00000206249->ENSP00000350283->ENSP00000372023->ENSP00000321656->ENSP00000300093->ENSP00000263753->ENSP00000418447->ENSP00000348784->ENSP00000379866 48

2633.ENSP00000296585->ENSP00000364094->ENSP00000380227->ENSP00000228307->ENSP00000341189->ENSP00000361021->ENSP00000269305->ENSP00000324897->ENSP00000381648 9

2634.ENSP00000296585->ENSP00000364094->ENSP00000380227->ENSP00000228307->ENSP00000341189->ENSP00000350941->ENSP00000206249->ENSP00000263253->ENSP00000384273 8

2635.ENSP00000296585->ENSP00000364094->ENSP00000386896 2

2636.ENSP00000296585->ENSP00000364094->ENSP00000380227->ENSP00000228307->ENSP00000341189->ENSP00000350941->ENSP00000206249->ENSP00000254227->ENSP00000342470->ENSP00000363868->ENSP00000393286 126

2637.ENSP00000296585->ENSP00000364094->ENSP00000380227->ENSP00000228307->ENSP00000341189->ENSP00000350941->ENSP00000206249->ENSP00000254227->ENSP00000342470->ENSP00000363868->ENSP00000393870 126

2638.ENSP00000296585->ENSP00000364094->ENSP00000346839->ENSP00000221930->ENSP00000262160->ENSP00000287727->ENSP00000400104 107

2639.ENSP00000296585->ENSP00000364094->ENSP00000380227->ENSP00000228307->ENSP00000341189->ENSP00000339007->ENSP00000312435->ENSP00000400365 19

2640.ENSP00000296585->ENSP00000364094->ENSP00000380227->ENSP00000228307->ENSP00000341189->ENSP00000350941->ENSP00000206249->ENSP00000254227->ENSP00000342470->ENSP00000363868->ENSP00000402590 126

2641.ENSP00000296585->ENSP00000364094->ENSP00000380227->ENSP00000228307->ENSP00000341189->ENSP00000350941->ENSP00000206249->ENSP00000399968->ENSP00000419692 8

2642.ENSP00000302564->ENSP00000309103->ENSP00000270202->ENSP00000348461->ENSP00000356505->ENSP00000220764->ENSP00000348380->ENSP00000305638 1020

2643.ENSP00000302564->ENSP00000269305->ENSP00000372023->ENSP00000321656->ENSP00000256442->ENSP00000342307->ENSP00000230538->ENSP00000354360->ENSP00000252999->ENSP00000307156 27

2644.ENSP00000302564->ENSP00000353059->ENSP00000307786 9

2645.ENSP00000302564->ENSP00000269305->ENSP00000266970->ENSP00000309181 4

2646.ENSP00000302564->ENSP00000269305->ENSP00000266970->ENSP00000311083 4

2647.ENSP00000302564->ENSP00000309103->ENSP00000270202->ENSP00000299421->ENSP00000228307->ENSP00000380227->ENSP00000364094->ENSP00000386896->ENSP00000200181->ENSP00000340937->ENSP00000348384->ENSP00000324532 18

2648.ENSP00000302564->ENSP00000269305->ENSP00000263253->ENSP00000384273->ENSP00000226574->ENSP00000327251 24

2649.ENSP00000302564->ENSP00000404503->ENSP00000329623 2

2650.ENSP00000302564->ENSP00000353059->ENSP00000330237 4

2651.ENSP00000302564->ENSP00000269305->ENSP00000212015->ENSP00000264867->ENSP00000287820->ENSP00000419692->ENSP00000332296 22

2652.ENSP00000302564->ENSP00000269305->ENSP00000344818->ENSP00000316840->ENSP00000361359->ENSP00000332468 6

2653.ENSP00000302564->ENSP00000269305->ENSP00000263253->ENSP00000384273->ENSP00000339151 5

2654.ENSP00000302564->ENSP00000269305->ENSP00000361021->ENSP00000341189 5

2655.ENSP00000302564->ENSP00000269305->ENSP00000293195->ENSP00000342087 36

2656.ENSP00000302564->ENSP00000269305->ENSP00000267163->ENSP00000345571 4

2657.ENSP00000302564->ENSP00000309103->ENSP00000270202->ENSP00000299421->ENSP00000228307->ENSP00000380227->ENSP00000346839 7

2658.ENSP00000302564->ENSP00000350311->ENSP00000311032->ENSP00000347858 4

2659.ENSP00000302564->ENSP00000309103->ENSP00000270202->ENSP00000299421->ENSP00000228307->ENSP00000380227->ENSP00000364094->ENSP00000386896->ENSP00000200181->ENSP00000340937->ENSP00000348384 17

2660.ENSP00000302564->ENSP00000404503->ENSP00000329623->ENSP00000367207->ENSP00000351490 5

2661.ENSP00000302564->ENSP00000309103->ENSP00000270202->ENSP00000352121 8

2662.ENSP00000302564->ENSP00000309103->ENSP00000270202->ENSP00000206249->ENSP00000327213->ENSP00000352900 19

2663.ENSP00000302564->ENSP00000353059 3

2664.ENSP00000302564->ENSP00000309103->ENSP00000270202->ENSP00000299421->ENSP00000228307->ENSP00000380227->ENSP00000364094->ENSP00000282588->ENSP00000364979->ENSP00000353654 20

2665.ENSP00000302564->ENSP00000269305->ENSP00000372023->ENSP00000321656->ENSP00000256442->ENSP00000342307->ENSP00000230538->ENSP00000354360 17

2666.ENSP00000302564->ENSP00000269305->ENSP00000267163->ENSP00000355249 4

2667.ENSP00000302564->ENSP00000269305->ENSP00000353483->ENSP00000360266->ENSP00000356438 9

2668.ENSP00000302564->ENSP00000269305->ENSP00000344818->ENSP00000316840->ENSP00000358622 5

2669.ENSP00000302564->ENSP00000269305->ENSP00000263253->ENSP00000384273->ENSP00000359424 5

2670.ENSP00000302564->ENSP00000269305->ENSP00000361021 3

2671.ENSP00000302564->ENSP00000269305->ENSP00000278916->ENSP00000312995->ENSP00000300093->ENSP00000263753->ENSP00000418447->ENSP00000348784->ENSP00000379866->ENSP00000379823->ENSP00000331902->ENSP00000361290 46

2672.ENSP00000302564->ENSP00000269305->ENSP00000344818->ENSP00000316840->ENSP00000361359->ENSP00000362994 6

2673.ENSP00000302564->ENSP00000309103->ENSP00000270202->ENSP00000206249->ENSP00000327213->ENSP00000363812 31

2674.ENSP00000302564->ENSP00000309103->ENSP00000270202->ENSP00000299421->ENSP00000228307->ENSP00000380227->ENSP00000364094 7

2675.ENSP00000302564->ENSP00000309103->ENSP00000270202->ENSP00000299421->ENSP00000228307->ENSP00000380227->ENSP00000364094->ENSP00000282588->ENSP00000364979 19

2676.ENSP00000302564->ENSP00000269305->ENSP00000361021->ENSP00000366563 15

2677.ENSP00000302564->ENSP00000404503->ENSP00000329623->ENSP00000367207 4

2678.ENSP00000302564->ENSP00000309103->ENSP00000270202->ENSP00000206249->ENSP00000254227->ENSP00000342470->ENSP00000363868->ENSP00000372703 124

2679.ENSP00000302564->ENSP00000269305->ENSP00000372023->ENSP00000321656->ENSP00000256442->ENSP00000342307->ENSP00000230538->ENSP00000222399->ENSP00000374309 25

2680.ENSP00000302564->ENSP00000309103->ENSP00000270202->ENSP00000348986->ENSP00000375892 8

2681.ENSP00000302564->ENSP00000269305->ENSP00000263253->ENSP00000264657->ENSP00000376765 7

2682.ENSP00000302564->ENSP00000269305->ENSP00000278916->ENSP00000312995->ENSP00000300093->ENSP00000263753->ENSP00000418447->ENSP00000348784->ENSP00000379866 43

2683.ENSP00000302564->ENSP00000269305->ENSP00000324897->ENSP00000381648 4

2684.ENSP00000302564->ENSP00000269305->ENSP00000263253->ENSP00000384273 4

2685.ENSP00000302564->ENSP00000309103->ENSP00000270202->ENSP00000299421->ENSP00000228307->ENSP00000380227->ENSP00000364094->ENSP00000386896 8

2686.ENSP00000302564->ENSP00000309103->ENSP00000270202->ENSP00000206249->ENSP00000254227->ENSP00000342470->ENSP00000363868->ENSP00000393286 124

2687.ENSP00000302564->ENSP00000309103->ENSP00000270202->ENSP00000206249->ENSP00000254227->ENSP00000342470->ENSP00000363868->ENSP00000393870 124

2688.ENSP00000302564->ENSP00000269305->ENSP00000263253->ENSP00000262160->ENSP00000287727->ENSP00000400104 105

2689.ENSP00000302564->ENSP00000309103->ENSP00000270202->ENSP00000297494->ENSP00000341940->ENSP00000312435->ENSP00000400365 17

2690.ENSP00000302564->ENSP00000309103->ENSP00000270202->ENSP00000206249->ENSP00000254227->ENSP00000342470->ENSP00000363868->ENSP00000402590 124

2691.ENSP00000302564->ENSP00000269305->ENSP00000212015->ENSP00000264867->ENSP00000287820->ENSP00000419692 6

2692.ENSP00000305638->ENSP00000348380->ENSP00000220764->ENSP00000356505->ENSP00000348461->ENSP00000270202->ENSP00000228872->ENSP00000274026->ENSP00000306043->ENSP00000342307->ENSP00000230538->ENSP00000354360->ENSP00000252999->ENSP00000307156 1044

2693.ENSP00000305638->ENSP00000348380->ENSP00000220764->ENSP00000356505->ENSP00000348461->ENSP00000270202->ENSP00000417281->ENSP00000269305->ENSP00000353059->ENSP00000307786 1028

2694.ENSP00000305638->ENSP00000348380->ENSP00000220764->ENSP00000356505->ENSP00000348461->ENSP00000270202->ENSP00000228872->ENSP00000266970->ENSP00000309181 1021

2695.ENSP00000305638->ENSP00000348380->ENSP00000220764->ENSP00000356505->ENSP00000348461->ENSP00000270202->ENSP00000228872->ENSP00000274255->ENSP00000311083 1021

2696.ENSP00000305638->ENSP00000348380->ENSP00000220764->ENSP00000356505->ENSP00000348461->ENSP00000381107->ENSP00000300574->ENSP00000228307->ENSP00000380227->ENSP00000364094->ENSP00000386896->ENSP00000200181->ENSP00000340937->ENSP00000348384->ENSP00000324532 1033

2697.ENSP00000305638->ENSP00000348380->ENSP00000220764->ENSP00000356505->ENSP00000348461->ENSP00000264657->ENSP00000263253->ENSP00000384273->ENSP00000226574->ENSP00000327251 1041

2698.ENSP00000305638->ENSP00000348380->ENSP00000220764->ENSP00000356505->ENSP00000348461->ENSP00000270202->ENSP00000309103->ENSP00000329623 1020

2699.ENSP00000305638->ENSP00000348380->ENSP00000220764->ENSP00000356505->ENSP00000348461->ENSP00000270202->ENSP00000417281->ENSP00000269305->ENSP00000353059->ENSP00000330237 1023

2700.ENSP00000305638->ENSP00000348380->ENSP00000220764->ENSP00000356505->ENSP00000348461->ENSP00000270202->ENSP00000206249->ENSP00000399968->ENSP00000419692->ENSP00000332296 1038

2701.ENSP00000305638->ENSP00000348380->ENSP00000220764->ENSP00000356505->ENSP00000348461->ENSP00000270202->ENSP00000417281->ENSP00000344818->ENSP00000316840->ENSP00000361359->ENSP00000332468 1023

2702.ENSP00000305638->ENSP00000348380->ENSP00000220764->ENSP00000356505->ENSP00000348461->ENSP00000270202->ENSP00000298552->ENSP00000339151 1022

2703.ENSP00000305638->ENSP00000348380->ENSP00000220764->ENSP00000356505->ENSP00000348461->ENSP00000302269->ENSP00000339007->ENSP00000341189 1020

2704.ENSP00000305638->ENSP00000348380->ENSP00000220764->ENSP00000356505->ENSP00000348461->ENSP00000270202->ENSP00000417281->ENSP00000269305->ENSP00000293195->ENSP00000342087 1054

2705.ENSP00000305638->ENSP00000348380->ENSP00000220764->ENSP00000356505->ENSP00000348461->ENSP00000270202->ENSP00000417281->ENSP00000267163->ENSP00000345571 1021

2706.ENSP00000305638->ENSP00000348380->ENSP00000220764->ENSP00000356505->ENSP00000348461->ENSP00000381107->ENSP00000300574->ENSP00000228307->ENSP00000380227->ENSP00000346839 1022

2707.ENSP00000305638->ENSP00000348380->ENSP00000220764->ENSP00000356505->ENSP00000348461->ENSP00000270202->ENSP00000309103->ENSP00000329623->ENSP00000311032->ENSP00000347858 1023

2708.ENSP00000305638->ENSP00000348380->ENSP00000220764->ENSP00000356505->ENSP00000348461->ENSP00000381107->ENSP00000300574->ENSP00000228307->ENSP00000380227->ENSP00000364094->ENSP00000386896->ENSP00000200181->ENSP00000340937->ENSP00000348384 1032

2709.ENSP00000305638->ENSP00000348380->ENSP00000220764->ENSP00000356505->ENSP00000348461->ENSP00000264657->ENSP00000263253->ENSP00000367207->ENSP00000351490 1022

2710.ENSP00000305638->ENSP00000348380->ENSP00000220764->ENSP00000356505->ENSP00000348461->ENSP00000270202->ENSP00000352121 1024

2711.ENSP00000305638->ENSP00000348380->ENSP00000220764->ENSP00000356505->ENSP00000348461->ENSP00000270202->ENSP00000206249->ENSP00000327213->ENSP00000352900 1035

2712.ENSP00000305638->ENSP00000348380->ENSP00000220764->ENSP00000356505->ENSP00000348461->ENSP00000270202->ENSP00000417281->ENSP00000269305->ENSP00000353059 1022

2713.ENSP00000305638->ENSP00000348380->ENSP00000220764->ENSP00000356505->ENSP00000348461->ENSP00000381107->ENSP00000300574->ENSP00000228307->ENSP00000380227->ENSP00000364094->ENSP00000282588->ENSP00000364979->ENSP00000353654 1035

2714.ENSP00000305638->ENSP00000348380->ENSP00000220764->ENSP00000356505->ENSP00000348461->ENSP00000270202->ENSP00000228872->ENSP00000274026->ENSP00000306043->ENSP00000342307->ENSP00000230538->ENSP00000354360 1034

2715.ENSP00000305638->ENSP00000348380->ENSP00000220764->ENSP00000356505->ENSP00000348461->ENSP00000270202->ENSP00000417281->ENSP00000267163->ENSP00000355249 1021

2716.ENSP00000305638->ENSP00000348380->ENSP00000220764->ENSP00000356505->ENSP00000348461->ENSP00000264657->ENSP00000258743->ENSP00000356438 1024

2717.ENSP00000305638->ENSP00000348380->ENSP00000220764->ENSP00000356505->ENSP00000348461->ENSP00000270202->ENSP00000417281->ENSP00000344818->ENSP00000316840->ENSP00000358622 1022

2718.ENSP00000305638->ENSP00000348380->ENSP00000220764->ENSP00000356505->ENSP00000348461->ENSP00000264657->ENSP00000263253->ENSP00000384273->ENSP00000359424 1022

2719.ENSP00000305638->ENSP00000348380->ENSP00000220764->ENSP00000356505->ENSP00000348461->ENSP00000270202->ENSP00000361021 1021

2720.ENSP00000305638->ENSP00000348380->ENSP00000220764->ENSP00000356505->ENSP00000348461->ENSP00000270202->ENSP00000417281->ENSP00000269305->ENSP00000278916->ENSP00000312995->ENSP00000300093->ENSP00000263753->ENSP00000418447->ENSP00000348784->ENSP00000379866->ENSP00000379823->ENSP00000331902->ENSP00000361290 1064

2721.ENSP00000305638->ENSP00000348380->ENSP00000220764->ENSP00000356505->ENSP00000348461->ENSP00000270202->ENSP00000417281->ENSP00000344818->ENSP00000316840->ENSP00000361359->ENSP00000362994 1023

2722.ENSP00000305638->ENSP00000348380->ENSP00000220764->ENSP00000356505->ENSP00000348461->ENSP00000270202->ENSP00000206249->ENSP00000327213->ENSP00000363812 1047

2723.ENSP00000305638->ENSP00000348380->ENSP00000220764->ENSP00000356505->ENSP00000348461->ENSP00000381107->ENSP00000300574->ENSP00000228307->ENSP00000380227->ENSP00000364094 1022

2724.ENSP00000305638->ENSP00000348380->ENSP00000220764->ENSP00000356505->ENSP00000348461->ENSP00000381107->ENSP00000300574->ENSP00000228307->ENSP00000380227->ENSP00000364094->ENSP00000282588->ENSP00000364979 1034

2725.ENSP00000305638->ENSP00000348380->ENSP00000220764->ENSP00000356505->ENSP00000348461->ENSP00000270202->ENSP00000361021->ENSP00000366563 1033

2726.ENSP00000305638->ENSP00000348380->ENSP00000220764->ENSP00000356505->ENSP00000348461->ENSP00000264657->ENSP00000263253->ENSP00000367207 1021

2727.ENSP00000305638->ENSP00000348380->ENSP00000220764->ENSP00000356505->ENSP00000348461->ENSP00000270202->ENSP00000206249->ENSP00000254227->ENSP00000342470->ENSP00000363868->ENSP00000372703 1140

2728.ENSP00000305638->ENSP00000348380->ENSP00000220764->ENSP00000356505->ENSP00000348461->ENSP00000270202->ENSP00000228872->ENSP00000274026->ENSP00000306043->ENSP00000342307->ENSP00000230538->ENSP00000222399->ENSP00000374309 1042

2729.ENSP00000305638->ENSP00000348380->ENSP00000220764->ENSP00000356505->ENSP00000348461->ENSP00000270202->ENSP00000348986->ENSP00000375892 1024

2730.ENSP00000305638->ENSP00000348380->ENSP00000220764->ENSP00000356505->ENSP00000348461->ENSP00000264657->ENSP00000376765 1022

2731.ENSP00000305638->ENSP00000348380->ENSP00000220764->ENSP00000356505->ENSP00000348461->ENSP00000270202->ENSP00000417281->ENSP00000269305->ENSP00000278916->ENSP00000312995->ENSP00000300093->ENSP00000263753->ENSP00000418447->ENSP00000348784->ENSP00000379866 1061

2732.ENSP00000305638->ENSP00000348380->ENSP00000220764->ENSP00000356505->ENSP00000348461->ENSP00000270202->ENSP00000417281->ENSP00000376076->ENSP00000381648 1022

2733.ENSP00000305638->ENSP00000348380->ENSP00000220764->ENSP00000356505->ENSP00000348461->ENSP00000264657->ENSP00000263253->ENSP00000384273 1021

2734.ENSP00000305638->ENSP00000348380->ENSP00000220764->ENSP00000356505->ENSP00000348461->ENSP00000381107->ENSP00000300574->ENSP00000228307->ENSP00000380227->ENSP00000364094->ENSP00000386896 1023

2735.ENSP00000305638->ENSP00000348380->ENSP00000220764->ENSP00000356505->ENSP00000348461->ENSP00000270202->ENSP00000206249->ENSP00000254227->ENSP00000342470->ENSP00000363868->ENSP00000393286 1140

2736.ENSP00000305638->ENSP00000348380->ENSP00000220764->ENSP00000356505->ENSP00000348461->ENSP00000270202->ENSP00000206249->ENSP00000254227->ENSP00000342470->ENSP00000363868->ENSP00000393870 1140

2737.ENSP00000305638->ENSP00000348380->ENSP00000220764->ENSP00000356505->ENSP00000348461->ENSP00000264657->ENSP00000263253->ENSP00000262160->ENSP00000287727->ENSP00000400104 1122

2738.ENSP00000305638->ENSP00000348380->ENSP00000220764->ENSP00000356505->ENSP00000348461->ENSP00000302269->ENSP00000339007->ENSP00000312435->ENSP00000400365 1033

2739.ENSP00000305638->ENSP00000348380->ENSP00000220764->ENSP00000356505->ENSP00000348461->ENSP00000270202->ENSP00000206249->ENSP00000254227->ENSP00000342470->ENSP00000363868->ENSP00000402590 1140

2740.ENSP00000305638->ENSP00000348380->ENSP00000220764->ENSP00000356505->ENSP00000348461->ENSP00000270202->ENSP00000206249->ENSP00000399968->ENSP00000419692 1022

2741.ENSP00000307156->ENSP00000252999->ENSP00000354360->ENSP00000230538->ENSP00000342307->ENSP00000256442->ENSP00000321656->ENSP00000372023->ENSP00000269305->ENSP00000353059->ENSP00000307786 33

2742.ENSP00000307156->ENSP00000252999->ENSP00000354360->ENSP00000230538->ENSP00000342307->ENSP00000306043->ENSP00000274026->ENSP00000266970->ENSP00000309181 26

2743.ENSP00000307156->ENSP00000252999->ENSP00000354360->ENSP00000230538->ENSP00000342307->ENSP00000306043->ENSP00000274026->ENSP00000266970->ENSP00000311083 26

2744.ENSP00000307156->ENSP00000252999->ENSP00000264144->ENSP00000324532 22

2745.ENSP00000307156->ENSP00000252999->ENSP00000354360->ENSP00000230538->ENSP00000342307->ENSP00000256442->ENSP00000321656->ENSP00000372023->ENSP00000269305->ENSP00000263253->ENSP00000384273->ENSP00000226574->ENSP00000327251 47

2746.ENSP00000307156->ENSP00000252999->ENSP00000354360->ENSP00000230538->ENSP00000342307->ENSP00000256442->ENSP00000321656->ENSP00000372023->ENSP00000269305->ENSP00000329623 26

2747.ENSP00000307156->ENSP00000252999->ENSP00000354360->ENSP00000230538->ENSP00000342307->ENSP00000256442->ENSP00000321656->ENSP00000372023->ENSP00000269305->ENSP00000353059->ENSP00000330237 28

2748.ENSP00000307156->ENSP00000252999->ENSP00000354360->ENSP00000230538->ENSP00000342307->ENSP00000256442->ENSP00000321656->ENSP00000372023->ENSP00000350283->ENSP00000206249->ENSP00000399968->ENSP00000419692->ENSP00000332296 44

2749.ENSP00000307156->ENSP00000252999->ENSP00000354360->ENSP00000230538->ENSP00000342307->ENSP00000256442->ENSP00000321656->ENSP00000372023->ENSP00000269305->ENSP00000344818->ENSP00000316840->ENSP00000361359->ENSP00000332468 29

2750.ENSP00000307156->ENSP00000252999->ENSP00000354360->ENSP00000230538->ENSP00000342307->ENSP00000256442->ENSP00000321656->ENSP00000372023->ENSP00000269305->ENSP00000263253->ENSP00000384273->ENSP00000339151 28

2751.ENSP00000307156->ENSP00000252999->ENSP00000354360->ENSP00000400365->ENSP00000312435->ENSP00000339007->ENSP00000341189 26

2752.ENSP00000307156->ENSP00000252999->ENSP00000354360->ENSP00000230538->ENSP00000342307->ENSP00000256442->ENSP00000321656->ENSP00000372023->ENSP00000269305->ENSP00000293195->ENSP00000342087 59

2753.ENSP00000307156->ENSP00000252999->ENSP00000354360->ENSP00000230538->ENSP00000342307->ENSP00000306043->ENSP00000274026->ENSP00000345571 25

2754.ENSP00000307156->ENSP00000252999->ENSP00000264144->ENSP00000340937->ENSP00000200181->ENSP00000386896->ENSP00000364094->ENSP00000346839 24

2755.ENSP00000307156->ENSP00000252999->ENSP00000354360->ENSP00000230538->ENSP00000342307->ENSP00000256442->ENSP00000321656->ENSP00000372023->ENSP00000269305->ENSP00000329623->ENSP00000311032->ENSP00000347858 29

2756.ENSP00000307156->ENSP00000252999->ENSP00000264144->ENSP00000324532->ENSP00000348384 23

2757.ENSP00000307156->ENSP00000252999->ENSP00000354360->ENSP00000230538->ENSP00000342307->ENSP00000306043->ENSP00000274026->ENSP00000244741->ENSP00000367207->ENSP00000351490 28

2758.ENSP00000307156->ENSP00000252999->ENSP00000354360->ENSP00000400365->ENSP00000312435->ENSP00000341940->ENSP00000297494->ENSP00000270202->ENSP00000352121 32

2759.ENSP00000307156->ENSP00000252999->ENSP00000354360->ENSP00000230538->ENSP00000342307->ENSP00000256442->ENSP00000321656->ENSP00000372023->ENSP00000350283->ENSP00000206249->ENSP00000327213->ENSP00000352900 41

2760.ENSP00000307156->ENSP00000252999->ENSP00000354360->ENSP00000230538->ENSP00000342307->ENSP00000256442->ENSP00000321656->ENSP00000372023->ENSP00000269305->ENSP00000353059 27

2761.ENSP00000307156->ENSP00000252999->ENSP00000264144->ENSP00000340937->ENSP00000200181->ENSP00000386896->ENSP00000364094->ENSP00000282588->ENSP00000364979->ENSP00000353654 36

2762.ENSP00000307156->ENSP00000252999->ENSP00000354360 10

2763.ENSP00000307156->ENSP00000252999->ENSP00000354360->ENSP00000230538->ENSP00000342307->ENSP00000256442->ENSP00000321656->ENSP00000372023->ENSP00000269305->ENSP00000267163->ENSP00000355249 27

2764.ENSP00000307156->ENSP00000252999->ENSP00000354360->ENSP00000230538->ENSP00000342307->ENSP00000256442->ENSP00000321656->ENSP00000372023->ENSP00000269305->ENSP00000353483->ENSP00000360266->ENSP00000356438 32

2765.ENSP00000307156->ENSP00000252999->ENSP00000354360->ENSP00000230538->ENSP00000342307->ENSP00000256442->ENSP00000321656->ENSP00000372023->ENSP00000269305->ENSP00000344818->ENSP00000316840->ENSP00000358622 28

2766.ENSP00000307156->ENSP00000252999->ENSP00000354360->ENSP00000230538->ENSP00000342307->ENSP00000256442->ENSP00000321656->ENSP00000372023->ENSP00000269305->ENSP00000263253->ENSP00000384273->ENSP00000359424 28

2767.ENSP00000307156->ENSP00000252999->ENSP00000354360->ENSP00000230538->ENSP00000342307->ENSP00000256442->ENSP00000321656->ENSP00000372023->ENSP00000269305->ENSP00000361021 26

2768.ENSP00000307156->ENSP00000252999->ENSP00000354360->ENSP00000230538->ENSP00000342307->ENSP00000256442->ENSP00000300093->ENSP00000263753->ENSP00000418447->ENSP00000348784->ENSP00000379866->ENSP00000379823->ENSP00000331902->ENSP00000361290 64

2769.ENSP00000307156->ENSP00000252999->ENSP00000354360->ENSP00000230538->ENSP00000342307->ENSP00000256442->ENSP00000321656->ENSP00000372023->ENSP00000269305->ENSP00000344818->ENSP00000316840->ENSP00000361359->ENSP00000362994 29

2770.ENSP00000307156->ENSP00000252999->ENSP00000354360->ENSP00000230538->ENSP00000342307->ENSP00000256442->ENSP00000321656->ENSP00000372023->ENSP00000350283->ENSP00000206249->ENSP00000327213->ENSP00000363812 53

2771.ENSP00000307156->ENSP00000252999->ENSP00000264144->ENSP00000340937->ENSP00000200181->ENSP00000386896->ENSP00000364094 23

2772.ENSP00000307156->ENSP00000252999->ENSP00000264144->ENSP00000340937->ENSP00000200181->ENSP00000386896->ENSP00000364094->ENSP00000282588->ENSP00000364979 35

2773.ENSP00000307156->ENSP00000252999->ENSP00000354360->ENSP00000230538->ENSP00000342307->ENSP00000256442->ENSP00000321656->ENSP00000372023->ENSP00000269305->ENSP00000361021->ENSP00000366563 38

2774.ENSP00000307156->ENSP00000252999->ENSP00000354360->ENSP00000230538->ENSP00000342307->ENSP00000306043->ENSP00000274026->ENSP00000244741->ENSP00000367207 27

2775.ENSP00000307156->ENSP00000252999->ENSP00000354360->ENSP00000400365->ENSP00000312435->ENSP00000363827->ENSP00000252486->ENSP00000252444->ENSP00000233242->ENSP00000236850->ENSP00000363868->ENSP00000372703 145

2776.ENSP00000307156->ENSP00000252999->ENSP00000258341->ENSP00000374309 12

2777.ENSP00000307156->ENSP00000252999->ENSP00000354360->ENSP00000400365->ENSP00000312435->ENSP00000339007->ENSP00000303830->ENSP00000348986->ENSP00000375892 32

2778.ENSP00000307156->ENSP00000252999->ENSP00000354360->ENSP00000400365->ENSP00000312435->ENSP00000339007->ENSP00000371067->ENSP00000264657->ENSP00000376765 30

2779.ENSP00000307156->ENSP00000252999->ENSP00000354360->ENSP00000230538->ENSP00000342307->ENSP00000256442->ENSP00000300093->ENSP00000263753->ENSP00000418447->ENSP00000348784->ENSP00000379866 61

2780.ENSP00000307156->ENSP00000252999->ENSP00000354360->ENSP00000230538->ENSP00000342307->ENSP00000256442->ENSP00000321656->ENSP00000372023->ENSP00000269305->ENSP00000324897->ENSP00000381648 27

2781.ENSP00000307156->ENSP00000252999->ENSP00000354360->ENSP00000230538->ENSP00000342307->ENSP00000256442->ENSP00000321656->ENSP00000372023->ENSP00000269305->ENSP00000263253->ENSP00000384273 27

2782.ENSP00000307156->ENSP00000252999->ENSP00000264144->ENSP00000340937->ENSP00000200181->ENSP00000386896 22

2783.ENSP00000307156->ENSP00000252999->ENSP00000354360->ENSP00000400365->ENSP00000312435->ENSP00000363827->ENSP00000252486->ENSP00000252444->ENSP00000233242->ENSP00000236850->ENSP00000363868->ENSP00000393286 145

2784.ENSP00000307156->ENSP00000252999->ENSP00000354360->ENSP00000400365->ENSP00000312435->ENSP00000363827->ENSP00000252486->ENSP00000252444->ENSP00000233242->ENSP00000236850->ENSP00000363868->ENSP00000393870 145

2785.ENSP00000307156->ENSP00000252999->ENSP00000354360->ENSP00000230538->ENSP00000342307->ENSP00000256442->ENSP00000321656->ENSP00000372023->ENSP00000269305->ENSP00000263253->ENSP00000262160->ENSP00000287727->ENSP00000400104 128

2786.ENSP00000307156->ENSP00000252999->ENSP00000354360->ENSP00000400365 11

2787.ENSP00000307156->ENSP00000252999->ENSP00000354360->ENSP00000400365->ENSP00000312435->ENSP00000363827->ENSP00000252486->ENSP00000252444->ENSP00000233242->ENSP00000236850->ENSP00000363868->ENSP00000402590 145

2788.ENSP00000307156->ENSP00000252999->ENSP00000354360->ENSP00000230538->ENSP00000342307->ENSP00000256442->ENSP00000321656->ENSP00000372023->ENSP00000350283->ENSP00000206249->ENSP00000399968->ENSP00000419692 28

2789.ENSP00000307786->ENSP00000353059->ENSP00000269305->ENSP00000266970->ENSP00000309181 10

2790.ENSP00000307786->ENSP00000353059->ENSP00000269305->ENSP00000266970->ENSP00000311083 10

2791.ENSP00000307786->ENSP00000353059->ENSP00000269305->ENSP00000361021->ENSP00000341189->ENSP00000228307->ENSP00000380227->ENSP00000364094->ENSP00000386896->ENSP00000200181->ENSP00000340937->ENSP00000348384->ENSP00000324532 25

2792.ENSP00000307786->ENSP00000353059->ENSP00000269305->ENSP00000262367->ENSP00000384273->ENSP00000226574->ENSP00000327251 30

2793.ENSP00000307786->ENSP00000353059->ENSP00000269305->ENSP00000329623 9

2794.ENSP00000307786->ENSP00000353059->ENSP00000330237 7

2795.ENSP00000307786->ENSP00000353059->ENSP00000269305->ENSP00000212015->ENSP00000264867->ENSP00000287820->ENSP00000419692->ENSP00000332296 28

2796.ENSP00000307786->ENSP00000353059->ENSP00000330237->ENSP00000347858->ENSP00000267169->ENSP00000227758->ENSP00000332468 11

2797.ENSP00000307786->ENSP00000353059->ENSP00000269305->ENSP00000262367->ENSP00000384273->ENSP00000339151 11

2798.ENSP00000307786->ENSP00000353059->ENSP00000269305->ENSP00000361021->ENSP00000341189 11

2799.ENSP00000307786->ENSP00000353059->ENSP00000269305->ENSP00000293195->ENSP00000342087 42

2800.ENSP00000307786->ENSP00000353059->ENSP00000345571 10

2801.ENSP00000307786->ENSP00000353059->ENSP00000269305->ENSP00000263253->ENSP00000262160->ENSP00000221930->ENSP00000346839 14

2802.ENSP00000307786->ENSP00000353059->ENSP00000330237->ENSP00000347858 8

2803.ENSP00000307786->ENSP00000353059->ENSP00000269305->ENSP00000361021->ENSP00000341189->ENSP00000228307->ENSP00000380227->ENSP00000364094->ENSP00000386896->ENSP00000200181->ENSP00000340937->ENSP00000348384 24

2804.ENSP00000307786->ENSP00000353059->ENSP00000269305->ENSP00000263253->ENSP00000367207->ENSP00000351490 11

2805.ENSP00000307786->ENSP00000353059->ENSP00000269305->ENSP00000417281->ENSP00000270202->ENSP00000352121 16

2806.ENSP00000307786->ENSP00000353059->ENSP00000269305->ENSP00000263253->ENSP00000206249->ENSP00000327213->ENSP00000352900 25

2807.ENSP00000307786->ENSP00000353059 6

2808.ENSP00000307786->ENSP00000353059->ENSP00000269305->ENSP00000361021->ENSP00000341189->ENSP00000228307->ENSP00000380227->ENSP00000364094->ENSP00000282588->ENSP00000364979->ENSP00000353654 27

2809.ENSP00000307786->ENSP00000353059->ENSP00000269305->ENSP00000278916->ENSP00000321656->ENSP00000256442->ENSP00000342307->ENSP00000230538->ENSP00000354360 23

2810.ENSP00000307786->ENSP00000353059->ENSP00000269305->ENSP00000267163->ENSP00000355249 10

2811.ENSP00000307786->ENSP00000353059->ENSP00000269305->ENSP00000353483->ENSP00000360266->ENSP00000356438 15

2812.ENSP00000307786->ENSP00000353059->ENSP00000330237->ENSP00000347858->ENSP00000216160->ENSP00000316840->ENSP00000358622 11

2813.ENSP00000307786->ENSP00000353059->ENSP00000269305->ENSP00000262367->ENSP00000384273->ENSP00000359424 11

2814.ENSP00000307786->ENSP00000353059->ENSP00000269305->ENSP00000361021 9

2815.ENSP00000307786->ENSP00000353059->ENSP00000269305->ENSP00000278916->ENSP00000312995->ENSP00000300093->ENSP00000263753->ENSP00000418447->ENSP00000348784->ENSP00000379866->ENSP00000379823->ENSP00000331902->ENSP00000361290 52

2816.ENSP00000307786->ENSP00000353059->ENSP00000330237->ENSP00000347858->ENSP00000267169->ENSP00000263464->ENSP00000362994 11

2817.ENSP00000307786->ENSP00000353059->ENSP00000269305->ENSP00000263253->ENSP00000206249->ENSP00000327213->ENSP00000363812 37

2818.ENSP00000307786->ENSP00000353059->ENSP00000269305->ENSP00000361021->ENSP00000341189->ENSP00000228307->ENSP00000380227->ENSP00000364094 14

2819.ENSP00000307786->ENSP00000353059->ENSP00000269305->ENSP00000361021->ENSP00000341189->ENSP00000228307->ENSP00000380227->ENSP00000364094->ENSP00000282588->ENSP00000364979 26

2820.ENSP00000307786->ENSP00000353059->ENSP00000269305->ENSP00000361021->ENSP00000366563 21

2821.ENSP00000307786->ENSP00000353059->ENSP00000269305->ENSP00000263253->ENSP00000367207 10

2822.ENSP00000307786->ENSP00000353059->ENSP00000269305->ENSP00000263253->ENSP00000206249->ENSP00000254227->ENSP00000342470->ENSP00000363868->ENSP00000372703 130

2823.ENSP00000307786->ENSP00000353059->ENSP00000269305->ENSP00000278916->ENSP00000321656->ENSP00000256442->ENSP00000342307->ENSP00000230538->ENSP00000222399->ENSP00000374309 31

2824.ENSP00000307786->ENSP00000353059->ENSP00000269305->ENSP00000353483->ENSP00000304895->ENSP00000348986->ENSP00000375892 16

2825.ENSP00000307786->ENSP00000353059->ENSP00000269305->ENSP00000263253->ENSP00000264657->ENSP00000376765 13

2826.ENSP00000307786->ENSP00000353059->ENSP00000269305->ENSP00000278916->ENSP00000312995->ENSP00000300093->ENSP00000263753->ENSP00000418447->ENSP00000348784->ENSP00000379866 49

2827.ENSP00000307786->ENSP00000353059->ENSP00000269305->ENSP00000324897->ENSP00000381648 10

2828.ENSP00000307786->ENSP00000353059->ENSP00000269305->ENSP00000262367->ENSP00000384273 10

2829.ENSP00000307786->ENSP00000353059->ENSP00000269305->ENSP00000361021->ENSP00000341189->ENSP00000228307->ENSP00000380227->ENSP00000364094->ENSP00000386896 15

2830.ENSP00000307786->ENSP00000353059->ENSP00000269305->ENSP00000263253->ENSP00000206249->ENSP00000254227->ENSP00000342470->ENSP00000363868->ENSP00000393286 130

2831.ENSP00000307786->ENSP00000353059->ENSP00000269305->ENSP00000263253->ENSP00000206249->ENSP00000254227->ENSP00000342470->ENSP00000363868->ENSP00000393870 130

2832.ENSP00000307786->ENSP00000353059->ENSP00000269305->ENSP00000329357->ENSP00000332973->ENSP00000287727->ENSP00000400104 111

2833.ENSP00000307786->ENSP00000353059->ENSP00000269305->ENSP00000278916->ENSP00000321656->ENSP00000256442->ENSP00000342307->ENSP00000230538->ENSP00000354360->ENSP00000400365 24

2834.ENSP00000307786->ENSP00000353059->ENSP00000269305->ENSP00000263253->ENSP00000206249->ENSP00000254227->ENSP00000342470->ENSP00000363868->ENSP00000402590 130

2835.ENSP00000307786->ENSP00000353059->ENSP00000269305->ENSP00000212015->ENSP00000264867->ENSP00000287820->ENSP00000419692 12

2836.ENSP00000309181->ENSP00000266970->ENSP00000311083 2

2837.ENSP00000309181->ENSP00000266970->ENSP00000269305->ENSP00000361021->ENSP00000341189->ENSP00000228307->ENSP00000380227->ENSP00000364094->ENSP00000386896->ENSP00000200181->ENSP00000340937->ENSP00000348384->ENSP00000324532 19

2838.ENSP00000309181->ENSP00000266970->ENSP00000269305->ENSP00000263253->ENSP00000384273->ENSP00000226574->ENSP00000327251 24

2839.ENSP00000309181->ENSP00000266970->ENSP00000269305->ENSP00000329623 3

2840.ENSP00000309181->ENSP00000266970->ENSP00000269305->ENSP00000353059->ENSP00000330237 5

2841.ENSP00000309181->ENSP00000266970->ENSP00000227507->ENSP00000344456->ENSP00000363822->ENSP00000399968->ENSP00000419692->ENSP00000332296 22

2842.ENSP00000309181->ENSP00000266970->ENSP00000269305->ENSP00000344818->ENSP00000316840->ENSP00000361359->ENSP00000332468 6

2843.ENSP00000309181->ENSP00000266970->ENSP00000269305->ENSP00000263253->ENSP00000384273->ENSP00000339151 5

2844.ENSP00000309181->ENSP00000266970->ENSP00000269305->ENSP00000361021->ENSP00000341189 5

2845.ENSP00000309181->ENSP00000266970->ENSP00000269305->ENSP00000293195->ENSP00000342087 36

2846.ENSP00000309181->ENSP00000266970->ENSP00000345571 3

2847.ENSP00000309181->ENSP00000266970->ENSP00000269305->ENSP00000263253->ENSP00000262160->ENSP00000221930->ENSP00000346839 8

2848.ENSP00000309181->ENSP00000266970->ENSP00000269305->ENSP00000329623->ENSP00000311032->ENSP00000347858 6

2849.ENSP00000309181->ENSP00000266970->ENSP00000269305->ENSP00000361021->ENSP00000341189->ENSP00000228307->ENSP00000380227->ENSP00000364094->ENSP00000386896->ENSP00000200181->ENSP00000340937->ENSP00000348384 18

2850.ENSP00000309181->ENSP00000266970->ENSP00000244741->ENSP00000367207->ENSP00000351490 5

2851.ENSP00000309181->ENSP00000266970->ENSP00000228872->ENSP00000270202->ENSP00000352121 9

2852.ENSP00000309181->ENSP00000266970->ENSP00000227507->ENSP00000206249->ENSP00000327213->ENSP00000352900 19

2853.ENSP00000309181->ENSP00000266970->ENSP00000269305->ENSP00000353059 4

2854.ENSP00000309181->ENSP00000266970->ENSP00000269305->ENSP00000361021->ENSP00000341189->ENSP00000228307->ENSP00000380227->ENSP00000364094->ENSP00000282588->ENSP00000364979->ENSP00000353654 21

2855.ENSP00000309181->ENSP00000266970->ENSP00000274026->ENSP00000306043->ENSP00000342307->ENSP00000230538->ENSP00000354360 16

2856.ENSP00000309181->ENSP00000266970->ENSP00000267163->ENSP00000355249 3

2857.ENSP00000309181->ENSP00000266970->ENSP00000269305->ENSP00000353483->ENSP00000360266->ENSP00000356438 9

2858.ENSP00000309181->ENSP00000266970->ENSP00000269305->ENSP00000344818->ENSP00000316840->ENSP00000358622 5

2859.ENSP00000309181->ENSP00000266970->ENSP00000269305->ENSP00000263253->ENSP00000384273->ENSP00000359424 5

2860.ENSP00000309181->ENSP00000266970->ENSP00000269305->ENSP00000361021 3

2861.ENSP00000309181->ENSP00000266970->ENSP00000303706->ENSP00000278916->ENSP00000312995->ENSP00000300093->ENSP00000263753->ENSP00000418447->ENSP00000348784->ENSP00000379866->ENSP00000379823->ENSP00000331902->ENSP00000361290 46

2862.ENSP00000309181->ENSP00000266970->ENSP00000269305->ENSP00000344818->ENSP00000316840->ENSP00000361359->ENSP00000362994 6

2863.ENSP00000309181->ENSP00000266970->ENSP00000227507->ENSP00000206249->ENSP00000327213->ENSP00000363812 31

2864.ENSP00000309181->ENSP00000266970->ENSP00000269305->ENSP00000361021->ENSP00000341189->ENSP00000228307->ENSP00000380227->ENSP00000364094 8

2865.ENSP00000309181->ENSP00000266970->ENSP00000269305->ENSP00000361021->ENSP00000341189->ENSP00000228307->ENSP00000380227->ENSP00000364094->ENSP00000282588->ENSP00000364979 20

2866.ENSP00000309181->ENSP00000266970->ENSP00000269305->ENSP00000361021->ENSP00000366563 15

2867.ENSP00000309181->ENSP00000266970->ENSP00000244741->ENSP00000367207 4

2868.ENSP00000309181->ENSP00000266970->ENSP00000227507->ENSP00000206249->ENSP00000254227->ENSP00000342470->ENSP00000363868->ENSP00000372703 124

2869.ENSP00000309181->ENSP00000266970->ENSP00000274026->ENSP00000306043->ENSP00000342307->ENSP00000230538->ENSP00000222399->ENSP00000374309 24

2870.ENSP00000309181->ENSP00000266970->ENSP00000228872->ENSP00000270202->ENSP00000348986->ENSP00000375892 9

2871.ENSP00000309181->ENSP00000266970->ENSP00000227507->ENSP00000264657->ENSP00000376765 7

2872.ENSP00000309181->ENSP00000266970->ENSP00000303706->ENSP00000278916->ENSP00000312995->ENSP00000300093->ENSP00000263753->ENSP00000418447->ENSP00000348784->ENSP00000379866 43

2873.ENSP00000309181->ENSP00000266970->ENSP00000269305->ENSP00000324897->ENSP00000381648 4

2874.ENSP00000309181->ENSP00000266970->ENSP00000269305->ENSP00000263253->ENSP00000384273 4

2875.ENSP00000309181->ENSP00000266970->ENSP00000269305->ENSP00000361021->ENSP00000341189->ENSP00000228307->ENSP00000380227->ENSP00000364094->ENSP00000386896 9

2876.ENSP00000309181->ENSP00000266970->ENSP00000227507->ENSP00000206249->ENSP00000254227->ENSP00000342470->ENSP00000363868->ENSP00000393286 124

2877.ENSP00000309181->ENSP00000266970->ENSP00000227507->ENSP00000206249->ENSP00000254227->ENSP00000342470->ENSP00000363868->ENSP00000393870 124

2878.ENSP00000309181->ENSP00000266970->ENSP00000269305->ENSP00000263253->ENSP00000262160->ENSP00000287727->ENSP00000400104 105

2879.ENSP00000309181->ENSP00000266970->ENSP00000274026->ENSP00000306043->ENSP00000342307->ENSP00000230538->ENSP00000354360->ENSP00000400365 17

2880.ENSP00000309181->ENSP00000266970->ENSP00000227507->ENSP00000206249->ENSP00000254227->ENSP00000342470->ENSP00000363868->ENSP00000402590 124

2881.ENSP00000309181->ENSP00000266970->ENSP00000227507->ENSP00000344456->ENSP00000363822->ENSP00000399968->ENSP00000419692 6

2882.ENSP00000311083->ENSP00000266970->ENSP00000269305->ENSP00000361021->ENSP00000341189->ENSP00000228307->ENSP00000380227->ENSP00000364094->ENSP00000386896->ENSP00000200181->ENSP00000340937->ENSP00000348384->ENSP00000324532 19

2883.ENSP00000311083->ENSP00000274255->ENSP00000231487->ENSP00000359206->ENSP00000216797->ENSP00000226574->ENSP00000327251 24

2884.ENSP00000311083->ENSP00000266970->ENSP00000269305->ENSP00000329623 3

2885.ENSP00000311083->ENSP00000266970->ENSP00000269305->ENSP00000353059->ENSP00000330237 5

2886.ENSP00000311083->ENSP00000266970->ENSP00000269305->ENSP00000212015->ENSP00000264867->ENSP00000287820->ENSP00000419692->ENSP00000332296 22

2887.ENSP00000311083->ENSP00000266970->ENSP00000269305->ENSP00000344818->ENSP00000316840->ENSP00000361359->ENSP00000332468 6

2888.ENSP00000311083->ENSP00000274255->ENSP00000231487->ENSP00000359206->ENSP00000216797->ENSP00000339151 5

2889.ENSP00000311083->ENSP00000266970->ENSP00000269305->ENSP00000361021->ENSP00000341189 5

2890.ENSP00000311083->ENSP00000266970->ENSP00000269305->ENSP00000293195->ENSP00000342087 36

2891.ENSP00000311083->ENSP00000266970->ENSP00000345571 3

2892.ENSP00000311083->ENSP00000266970->ENSP00000269305->ENSP00000263253->ENSP00000262160->ENSP00000221930->ENSP00000346839 8

2893.ENSP00000311083->ENSP00000266970->ENSP00000269305->ENSP00000329623->ENSP00000311032->ENSP00000347858 6

2894.ENSP00000311083->ENSP00000266970->ENSP00000269305->ENSP00000361021->ENSP00000341189->ENSP00000228307->ENSP00000380227->ENSP00000364094->ENSP00000386896->ENSP00000200181->ENSP00000340937->ENSP00000348384 18

2895.ENSP00000311083->ENSP00000274255->ENSP00000367207->ENSP00000351490 4

2896.ENSP00000311083->ENSP00000274255->ENSP00000228872->ENSP00000270202->ENSP00000352121 9

2897.ENSP00000311083->ENSP00000266970->ENSP00000227507->ENSP00000206249->ENSP00000327213->ENSP00000352900 19

2898.ENSP00000311083->ENSP00000266970->ENSP00000269305->ENSP00000353059 4

2899.ENSP00000311083->ENSP00000266970->ENSP00000269305->ENSP00000361021->ENSP00000341189->ENSP00000228307->ENSP00000380227->ENSP00000364094->ENSP00000282588->ENSP00000364979->ENSP00000353654 21

2900.ENSP00000311083->ENSP00000266970->ENSP00000274026->ENSP00000306043->ENSP00000342307->ENSP00000230538->ENSP00000354360 16

2901.ENSP00000311083->ENSP00000266970->ENSP00000267163->ENSP00000355249 3

2902.ENSP00000311083->ENSP00000266970->ENSP00000269305->ENSP00000344352->ENSP00000360266->ENSP00000356438 9

2903.ENSP00000311083->ENSP00000266970->ENSP00000269305->ENSP00000344818->ENSP00000316840->ENSP00000358622 5

2904.ENSP00000311083->ENSP00000274255->ENSP00000231487->ENSP00000359206->ENSP00000216797->ENSP00000359424 5

2905.ENSP00000311083->ENSP00000266970->ENSP00000269305->ENSP00000361021 3

2906.ENSP00000311083->ENSP00000266970->ENSP00000303706->ENSP00000278916->ENSP00000321656->ENSP00000300093->ENSP00000263753->ENSP00000418447->ENSP00000348784->ENSP00000379866->ENSP00000379823->ENSP00000331902->ENSP00000361290 46

2907.ENSP00000311083->ENSP00000266970->ENSP00000269305->ENSP00000344818->ENSP00000316840->ENSP00000361359->ENSP00000362994 6

2908.ENSP00000311083->ENSP00000266970->ENSP00000227507->ENSP00000206249->ENSP00000327213->ENSP00000363812 31

2909.ENSP00000311083->ENSP00000266970->ENSP00000269305->ENSP00000361021->ENSP00000341189->ENSP00000228307->ENSP00000380227->ENSP00000364094 8

2910.ENSP00000311083->ENSP00000266970->ENSP00000269305->ENSP00000361021->ENSP00000341189->ENSP00000228307->ENSP00000380227->ENSP00000364094->ENSP00000282588->ENSP00000364979 20

2911.ENSP00000311083->ENSP00000266970->ENSP00000269305->ENSP00000361021->ENSP00000366563 15

2912.ENSP00000311083->ENSP00000274255->ENSP00000367207 3

2913.ENSP00000311083->ENSP00000266970->ENSP00000227507->ENSP00000206249->ENSP00000254227->ENSP00000342470->ENSP00000363868->ENSP00000372703 124

2914.ENSP00000311083->ENSP00000266970->ENSP00000274026->ENSP00000306043->ENSP00000342307->ENSP00000230538->ENSP00000222399->ENSP00000374309 24

2915.ENSP00000311083->ENSP00000274255->ENSP00000228872->ENSP00000270202->ENSP00000348986->ENSP00000375892 9

2916.ENSP00000311083->ENSP00000266970->ENSP00000227507->ENSP00000264657->ENSP00000376765 7

2917.ENSP00000311083->ENSP00000266970->ENSP00000303706->ENSP00000278916->ENSP00000321656->ENSP00000300093->ENSP00000263753->ENSP00000418447->ENSP00000348784->ENSP00000379866 43

2918.ENSP00000311083->ENSP00000266970->ENSP00000269305->ENSP00000324897->ENSP00000381648 4

2919.ENSP00000311083->ENSP00000266970->ENSP00000269305->ENSP00000263253->ENSP00000384273 4

2920.ENSP00000311083->ENSP00000266970->ENSP00000269305->ENSP00000361021->ENSP00000341189->ENSP00000228307->ENSP00000380227->ENSP00000364094->ENSP00000386896 9

2921.ENSP00000311083->ENSP00000266970->ENSP00000227507->ENSP00000206249->ENSP00000254227->ENSP00000342470->ENSP00000363868->ENSP00000393286 124

2922.ENSP00000311083->ENSP00000266970->ENSP00000227507->ENSP00000206249->ENSP00000254227->ENSP00000342470->ENSP00000363868->ENSP00000393870 124

2923.ENSP00000311083->ENSP00000266970->ENSP00000269305->ENSP00000329357->ENSP00000332973->ENSP00000287727->ENSP00000400104 105

2924.ENSP00000311083->ENSP00000266970->ENSP00000274026->ENSP00000306043->ENSP00000342307->ENSP00000230538->ENSP00000354360->ENSP00000400365 17

2925.ENSP00000311083->ENSP00000266970->ENSP00000227507->ENSP00000206249->ENSP00000254227->ENSP00000342470->ENSP00000363868->ENSP00000402590 124

2926.ENSP00000311083->ENSP00000266970->ENSP00000269305->ENSP00000212015->ENSP00000264867->ENSP00000287820->ENSP00000419692 6

2927.ENSP00000324532->ENSP00000348384->ENSP00000340937->ENSP00000200181->ENSP00000386896->ENSP00000364094->ENSP00000367316->ENSP00000303242->ENSP00000264832->ENSP00000226574->ENSP00000327251 38

2928.ENSP00000324532->ENSP00000348384->ENSP00000340937->ENSP00000200181->ENSP00000386896->ENSP00000364094->ENSP00000380227->ENSP00000228307->ENSP00000341189->ENSP00000361021->ENSP00000269305->ENSP00000329623 18

2929.ENSP00000324532->ENSP00000348384->ENSP00000340937->ENSP00000200181->ENSP00000386896->ENSP00000364094->ENSP00000380227->ENSP00000228307->ENSP00000341189->ENSP00000361021->ENSP00000269305->ENSP00000353059->ENSP00000330237 20

2930.ENSP00000324532->ENSP00000348384->ENSP00000340937->ENSP00000200181->ENSP00000386896->ENSP00000364094->ENSP00000380227->ENSP00000228307->ENSP00000341189->ENSP00000350941->ENSP00000206249->ENSP00000399968->ENSP00000419692->ENSP00000332296 34

2931.ENSP00000324532->ENSP00000348384->ENSP00000340937->ENSP00000200181->ENSP00000386896->ENSP00000364094->ENSP00000380227->ENSP00000228307->ENSP00000300574->ENSP00000275493->ENSP00000344818->ENSP00000316840->ENSP00000361359->ENSP00000332468 20

2932.ENSP00000324532->ENSP00000348384->ENSP00000340937->ENSP00000200181->ENSP00000386896->ENSP00000364094->ENSP00000346839->ENSP00000221930->ENSP00000262160->ENSP00000263253->ENSP00000384273->ENSP00000339151 19

2933.ENSP00000324532->ENSP00000348384->ENSP00000340937->ENSP00000200181->ENSP00000386896->ENSP00000364094->ENSP00000380227->ENSP00000228307->ENSP00000341189 14

2934.ENSP00000324532->ENSP00000348384->ENSP00000340937->ENSP00000200181->ENSP00000386896->ENSP00000364094->ENSP00000380227->ENSP00000228307->ENSP00000341189->ENSP00000361021->ENSP00000269305->ENSP00000293195->ENSP00000342087 51

2935.ENSP00000324532->ENSP00000348384->ENSP00000340937->ENSP00000200181->ENSP00000386896->ENSP00000364094->ENSP00000380227->ENSP00000228307->ENSP00000341189->ENSP00000350941->ENSP00000206249->ENSP00000329357->ENSP00000345571 18

2936.ENSP00000324532->ENSP00000348384->ENSP00000340937->ENSP00000200181->ENSP00000386896->ENSP00000364094->ENSP00000346839 12

2937.ENSP00000324532->ENSP00000348384->ENSP00000340937->ENSP00000200181->ENSP00000386896->ENSP00000364094->ENSP00000380227->ENSP00000228307->ENSP00000300574->ENSP00000275493->ENSP00000344818->ENSP00000316840->ENSP00000216160->ENSP00000347858 20

2938.ENSP00000324532->ENSP00000348384 1

2939.ENSP00000324532->ENSP00000348384->ENSP00000340937->ENSP00000200181->ENSP00000386896->ENSP00000364094->ENSP00000380227->ENSP00000228307->ENSP00000341189->ENSP00000339007->ENSP00000215832->ENSP00000367207->ENSP00000351490 19

2940.ENSP00000324532->ENSP00000348384->ENSP00000340937->ENSP00000200181->ENSP00000386896->ENSP00000364094->ENSP00000380227->ENSP00000228307->ENSP00000299421->ENSP00000270202->ENSP00000352121 22

2941.ENSP00000324532->ENSP00000348384->ENSP00000340937->ENSP00000200181->ENSP00000386896->ENSP00000364094->ENSP00000380227->ENSP00000228307->ENSP00000341189->ENSP00000350941->ENSP00000206249->ENSP00000327213->ENSP00000352900 31

2942.ENSP00000324532->ENSP00000348384->ENSP00000340937->ENSP00000200181->ENSP00000386896->ENSP00000364094->ENSP00000380227->ENSP00000228307->ENSP00000341189->ENSP00000361021->ENSP00000269305->ENSP00000353059 19

2943.ENSP00000324532->ENSP00000348384->ENSP00000340937->ENSP00000200181->ENSP00000386896->ENSP00000364094->ENSP00000282588->ENSP00000364979->ENSP00000353654 24

2944.ENSP00000324532->ENSP00000264144->ENSP00000252999->ENSP00000354360 22

2945.ENSP00000324532->ENSP00000348384->ENSP00000340937->ENSP00000200181->ENSP00000386896->ENSP00000364094->ENSP00000380227->ENSP00000228307->ENSP00000299421->ENSP00000270202->ENSP00000417281->ENSP00000267163->ENSP00000355249 19

2946.ENSP00000324532->ENSP00000348384->ENSP00000340937->ENSP00000200181->ENSP00000386896->ENSP00000364094->ENSP00000380227->ENSP00000228307->ENSP00000341189->ENSP00000350941->ENSP00000264657->ENSP00000258743->ENSP00000356438 21

2947.ENSP00000324532->ENSP00000348384->ENSP00000340937->ENSP00000200181->ENSP00000386896->ENSP00000364094->ENSP00000380227->ENSP00000228307->ENSP00000300574->ENSP00000275493->ENSP00000344818->ENSP00000316840->ENSP00000358622 19

2948.ENSP00000324532->ENSP00000348384->ENSP00000340937->ENSP00000200181->ENSP00000386896->ENSP00000364094->ENSP00000346839->ENSP00000221930->ENSP00000262160->ENSP00000263253->ENSP00000384273->ENSP00000359424 19

2949.ENSP00000324532->ENSP00000348384->ENSP00000340937->ENSP00000200181->ENSP00000386896->ENSP00000364094->ENSP00000380227->ENSP00000228307->ENSP00000341189->ENSP00000361021 16

2950.ENSP00000324532->ENSP00000348384->ENSP00000340937->ENSP00000200181->ENSP00000386896->ENSP00000364094->ENSP00000380227->ENSP00000228307->ENSP00000341189->ENSP00000361021->ENSP00000269305->ENSP00000278916->ENSP00000312995->ENSP00000300093->ENSP00000263753->ENSP00000418447->ENSP00000348784->ENSP00000379866->ENSP00000379823->ENSP00000331902->ENSP00000361290 61

2951.ENSP00000324532->ENSP00000348384->ENSP00000340937->ENSP00000200181->ENSP00000386896->ENSP00000364094->ENSP00000380227->ENSP00000228307->ENSP00000300574->ENSP00000275493->ENSP00000344818->ENSP00000316840->ENSP00000361359->ENSP00000362994 20

2952.ENSP00000324532->ENSP00000348384->ENSP00000340937->ENSP00000200181->ENSP00000386896->ENSP00000364094->ENSP00000380227->ENSP00000228307->ENSP00000341189->ENSP00000350941->ENSP00000206249->ENSP00000327213->ENSP00000363812 43

2953.ENSP00000324532->ENSP00000348384->ENSP00000340937->ENSP00000200181->ENSP00000386896->ENSP00000364094 11

2954.ENSP00000324532->ENSP00000348384->ENSP00000340937->ENSP00000200181->ENSP00000386896->ENSP00000364094->ENSP00000282588->ENSP00000364979 23

2955.ENSP00000324532->ENSP00000348384->ENSP00000340937->ENSP00000200181->ENSP00000386896->ENSP00000364094->ENSP00000380227->ENSP00000228307->ENSP00000341189->ENSP00000361021->ENSP00000366563 28

2956.ENSP00000324532->ENSP00000348384->ENSP00000340937->ENSP00000200181->ENSP00000386896->ENSP00000364094->ENSP00000380227->ENSP00000228307->ENSP00000341189->ENSP00000339007->ENSP00000215832->ENSP00000367207 18

2957.ENSP00000324532->ENSP00000348384->ENSP00000340937->ENSP00000200181->ENSP00000386896->ENSP00000364094->ENSP00000380227->ENSP00000228307->ENSP00000341189->ENSP00000350941->ENSP00000206249->ENSP00000254227->ENSP00000342470->ENSP00000363868->ENSP00000372703 136

2958.ENSP00000324532->ENSP00000264144->ENSP00000252999->ENSP00000258341->ENSP00000374309 24

2959.ENSP00000324532->ENSP00000348384->ENSP00000340937->ENSP00000200181->ENSP00000386896->ENSP00000364094->ENSP00000380227->ENSP00000228307->ENSP00000341189->ENSP00000339007->ENSP00000304895->ENSP00000348986->ENSP00000375892 22

2960.ENSP00000324532->ENSP00000348384->ENSP00000340937->ENSP00000200181->ENSP00000386896->ENSP00000364094->ENSP00000380227->ENSP00000228307->ENSP00000341189->ENSP00000350941->ENSP00000264657->ENSP00000376765 19

2961.ENSP00000324532->ENSP00000348384->ENSP00000340937->ENSP00000200181->ENSP00000386896->ENSP00000364094->ENSP00000380227->ENSP00000228307->ENSP00000341189->ENSP00000361021->ENSP00000269305->ENSP00000278916->ENSP00000312995->ENSP00000300093->ENSP00000263753->ENSP00000418447->ENSP00000348784->ENSP00000379866 58

2962.ENSP00000324532->ENSP00000348384->ENSP00000340937->ENSP00000200181->ENSP00000386896->ENSP00000364094->ENSP00000380227->ENSP00000228307->ENSP00000341189->ENSP00000361021->ENSP00000269305->ENSP00000324897->ENSP00000381648 19

2963.ENSP00000324532->ENSP00000348384->ENSP00000340937->ENSP00000200181->ENSP00000386896->ENSP00000364094->ENSP00000346839->ENSP00000221930->ENSP00000262160->ENSP00000263253->ENSP00000384273 18

2964.ENSP00000324532->ENSP00000348384->ENSP00000340937->ENSP00000200181->ENSP00000386896 10

2965.ENSP00000324532->ENSP00000348384->ENSP00000340937->ENSP00000200181->ENSP00000386896->ENSP00000364094->ENSP00000380227->ENSP00000228307->ENSP00000341189->ENSP00000350941->ENSP00000206249->ENSP00000254227->ENSP00000342470->ENSP00000363868->ENSP00000393286 136

2966.ENSP00000324532->ENSP00000348384->ENSP00000340937->ENSP00000200181->ENSP00000386896->ENSP00000364094->ENSP00000380227->ENSP00000228307->ENSP00000341189->ENSP00000350941->ENSP00000206249->ENSP00000254227->ENSP00000342470->ENSP00000363868->ENSP00000393870 136

2967.ENSP00000324532->ENSP00000348384->ENSP00000340937->ENSP00000200181->ENSP00000386896->ENSP00000364094->ENSP00000346839->ENSP00000221930->ENSP00000262160->ENSP00000287727->ENSP00000400104 117

2968.ENSP00000324532->ENSP00000264144->ENSP00000252999->ENSP00000354360->ENSP00000400365 23

2969.ENSP00000324532->ENSP00000348384->ENSP00000340937->ENSP00000200181->ENSP00000386896->ENSP00000364094->ENSP00000380227->ENSP00000228307->ENSP00000341189->ENSP00000350941->ENSP00000206249->ENSP00000254227->ENSP00000342470->ENSP00000363868->ENSP00000402590 136

2970.ENSP00000324532->ENSP00000348384->ENSP00000340937->ENSP00000200181->ENSP00000386896->ENSP00000364094->ENSP00000380227->ENSP00000228307->ENSP00000341189->ENSP00000350941->ENSP00000206249->ENSP00000399968->ENSP00000419692 18

2971.ENSP00000327251->ENSP00000226574->ENSP00000384273->ENSP00000262367->ENSP00000269305->ENSP00000329623 23

2972.ENSP00000327251->ENSP00000226574->ENSP00000384273->ENSP00000262367->ENSP00000269305->ENSP00000353059->ENSP00000330237 25

2973.ENSP00000327251->ENSP00000226574->ENSP00000231509->ENSP00000320940->ENSP00000348827->ENSP00000419692->ENSP00000332296 40

2974.ENSP00000327251->ENSP00000226574->ENSP00000359424->ENSP00000247668->ENSP00000227758->ENSP00000332468 23

2975.ENSP00000327251->ENSP00000226574->ENSP00000339151 20

2976.ENSP00000327251->ENSP00000226574->ENSP00000384273->ENSP00000263253->ENSP00000264657->ENSP00000350941->ENSP00000341189 24

2977.ENSP00000327251->ENSP00000226574->ENSP00000384273->ENSP00000262367->ENSP00000269305->ENSP00000293195->ENSP00000342087 56

2978.ENSP00000327251->ENSP00000226574->ENSP00000384273->ENSP00000362649->ENSP00000267163->ENSP00000345571 23

2979.ENSP00000327251->ENSP00000226574->ENSP00000384273->ENSP00000263253->ENSP00000262160->ENSP00000221930->ENSP00000346839 26

2980.ENSP00000327251->ENSP00000226574->ENSP00000359424->ENSP00000247668->ENSP00000227758->ENSP00000267169->ENSP00000347858 24

2981.ENSP00000327251->ENSP00000226574->ENSP00000264832->ENSP00000303242->ENSP00000367316->ENSP00000364094->ENSP00000386896->ENSP00000200181->ENSP00000340937->ENSP00000348384 37

2982.ENSP00000327251->ENSP00000226574->ENSP00000384273->ENSP00000263253->ENSP00000367207->ENSP00000351490 23

2983.ENSP00000327251->ENSP00000226574->ENSP00000384273->ENSP00000262367->ENSP00000387699->ENSP00000270202->ENSP00000352121 30

2984.ENSP00000327251->ENSP00000226574->ENSP00000384273->ENSP00000263253->ENSP00000206249->ENSP00000327213->ENSP00000352900 37

2985.ENSP00000327251->ENSP00000226574->ENSP00000384273->ENSP00000262367->ENSP00000269305->ENSP00000353059 24

2986.ENSP00000327251->ENSP00000226574->ENSP00000264832->ENSP00000303242->ENSP00000367316->ENSP00000364094->ENSP00000282588->ENSP00000364979->ENSP00000353654 40

2987.ENSP00000327251->ENSP00000226574->ENSP00000384273->ENSP00000262367->ENSP00000269305->ENSP00000278916->ENSP00000321656->ENSP00000256442->ENSP00000342307->ENSP00000230538->ENSP00000354360 37

2988.ENSP00000327251->ENSP00000226574->ENSP00000384273->ENSP00000362649->ENSP00000267163->ENSP00000355249 23

2989.ENSP00000327251->ENSP00000226574->ENSP00000263341->ENSP00000356438 25

2990.ENSP00000327251->ENSP00000226574->ENSP00000359424->ENSP00000358622 21

2991.ENSP00000327251->ENSP00000226574->ENSP00000359424 20

2992.ENSP00000327251->ENSP00000226574->ENSP00000384273->ENSP00000262367->ENSP00000269305->ENSP00000361021 23

2993.ENSP00000327251->ENSP00000226574->ENSP00000384273->ENSP00000262367->ENSP00000269305->ENSP00000278916->ENSP00000321656->ENSP00000300093->ENSP00000263753->ENSP00000418447->ENSP00000348784->ENSP00000379866->ENSP00000379823->ENSP00000331902->ENSP00000361290 66

2994.ENSP00000327251->ENSP00000226574->ENSP00000362994 22

2995.ENSP00000327251->ENSP00000226574->ENSP00000384273->ENSP00000263253->ENSP00000206249->ENSP00000327213->ENSP00000363812 49

2996.ENSP00000327251->ENSP00000226574->ENSP00000264832->ENSP00000303242->ENSP00000367316->ENSP00000364094 27

2997.ENSP00000327251->ENSP00000226574->ENSP00000264832->ENSP00000303242->ENSP00000367316->ENSP00000364094->ENSP00000282588->ENSP00000364979 39

2998.ENSP00000327251->ENSP00000226574->ENSP00000384273->ENSP00000262367->ENSP00000269305->ENSP00000361021->ENSP00000366563 35

2999.ENSP00000327251->ENSP00000226574->ENSP00000384273->ENSP00000263253->ENSP00000367207 22

3000.ENSP00000327251->ENSP00000226574->ENSP00000384273->ENSP00000263253->ENSP00000206249->ENSP00000254227->ENSP00000342470->ENSP00000363868->ENSP00000372703 142

3001.ENSP00000327251->ENSP00000226574->ENSP00000384273->ENSP00000262367->ENSP00000269305->ENSP00000278916->ENSP00000321656->ENSP00000256442->ENSP00000342307->ENSP00000230538->ENSP00000222399->ENSP00000374309 45

3002.ENSP00000327251->ENSP00000226574->ENSP00000384273->ENSP00000262367->ENSP00000387699->ENSP00000270202->ENSP00000348986->ENSP00000375892 30

3003.ENSP00000327251->ENSP00000226574->ENSP00000384273->ENSP00000263253->ENSP00000264657->ENSP00000376765 25

3004.ENSP00000327251->ENSP00000226574->ENSP00000384273->ENSP00000262367->ENSP00000269305->ENSP00000278916->ENSP00000321656->ENSP00000300093->ENSP00000263753->ENSP00000418447->ENSP00000348784->ENSP00000379866 63

3005.ENSP00000327251->ENSP00000226574->ENSP00000384273->ENSP00000262367->ENSP00000269305->ENSP00000324897->ENSP00000381648 24

3006.ENSP00000327251->ENSP00000226574->ENSP00000384273 20

3007.ENSP00000327251->ENSP00000226574->ENSP00000264832->ENSP00000303242->ENSP00000367316->ENSP00000364094->ENSP00000386896 28

3008.ENSP00000327251->ENSP00000226574->ENSP00000384273->ENSP00000263253->ENSP00000206249->ENSP00000254227->ENSP00000342470->ENSP00000363868->ENSP00000393286 142

3009.ENSP00000327251->ENSP00000226574->ENSP00000384273->ENSP00000263253->ENSP00000206249->ENSP00000254227->ENSP00000342470->ENSP00000363868->ENSP00000393870 142

3010.ENSP00000327251->ENSP00000226574->ENSP00000384273->ENSP00000263253->ENSP00000262160->ENSP00000287727->ENSP00000400104 123

3011.ENSP00000327251->ENSP00000226574->ENSP00000384273->ENSP00000262367->ENSP00000269305->ENSP00000278916->ENSP00000321656->ENSP00000256442->ENSP00000342307->ENSP00000230538->ENSP00000354360->ENSP00000400365 38

3012.ENSP00000327251->ENSP00000226574->ENSP00000384273->ENSP00000263253->ENSP00000206249->ENSP00000254227->ENSP00000342470->ENSP00000363868->ENSP00000402590 142

3013.ENSP00000327251->ENSP00000226574->ENSP00000231509->ENSP00000320940->ENSP00000348827->ENSP00000419692 24

3014.ENSP00000329623->ENSP00000269305->ENSP00000353059->ENSP00000330237 4

3015.ENSP00000329623->ENSP00000269305->ENSP00000263253->ENSP00000325690->ENSP00000399968->ENSP00000419692->ENSP00000332296 21

3016.ENSP00000329623->ENSP00000269305->ENSP00000344818->ENSP00000316840->ENSP00000361359->ENSP00000332468 5

3017.ENSP00000329623->ENSP00000269305->ENSP00000362649->ENSP00000384273->ENSP00000339151 4

3018.ENSP00000329623->ENSP00000269305->ENSP00000361021->ENSP00000341189 4

3019.ENSP00000329623->ENSP00000269305->ENSP00000293195->ENSP00000342087 35

3020.ENSP00000329623->ENSP00000269305->ENSP00000267163->ENSP00000345571 3

3021.ENSP00000329623->ENSP00000269305->ENSP00000263253->ENSP00000262160->ENSP00000221930->ENSP00000346839 7

3022.ENSP00000329623->ENSP00000311032->ENSP00000347858 3

3023.ENSP00000329623->ENSP00000269305->ENSP00000361021->ENSP00000341189->ENSP00000228307->ENSP00000380227->ENSP00000364094->ENSP00000386896->ENSP00000200181->ENSP00000340937->ENSP00000348384 17

3024.ENSP00000329623->ENSP00000367207->ENSP00000351490 3

3025.ENSP00000329623->ENSP00000309103->ENSP00000270202->ENSP00000352121 8

3026.ENSP00000329623->ENSP00000269305->ENSP00000263253->ENSP00000206249->ENSP00000327213->ENSP00000352900 18

3027.ENSP00000329623->ENSP00000269305->ENSP00000353059 3

3028.ENSP00000329623->ENSP00000269305->ENSP00000361021->ENSP00000341189->ENSP00000228307->ENSP00000380227->ENSP00000364094->ENSP00000282588->ENSP00000364979->ENSP00000353654 20

3029.ENSP00000329623->ENSP00000269305->ENSP00000361275->ENSP00000321656->ENSP00000256442->ENSP00000342307->ENSP00000230538->ENSP00000354360 16

3030.ENSP00000329623->ENSP00000269305->ENSP00000267163->ENSP00000355249 3

3031.ENSP00000329623->ENSP00000269305->ENSP00000344352->ENSP00000360266->ENSP00000356438 8

3032.ENSP00000329623->ENSP00000269305->ENSP00000344818->ENSP00000316840->ENSP00000358622 4

3033.ENSP00000329623->ENSP00000269305->ENSP00000362649->ENSP00000384273->ENSP00000359424 4

3034.ENSP00000329623->ENSP00000269305->ENSP00000361021 2

3035.ENSP00000329623->ENSP00000269305->ENSP00000278916->ENSP00000312995->ENSP00000300093->ENSP00000263753->ENSP00000418447->ENSP00000348784->ENSP00000379866->ENSP00000379823->ENSP00000331902->ENSP00000361290 45

3036.ENSP00000329623->ENSP00000269305->ENSP00000344818->ENSP00000316840->ENSP00000361359->ENSP00000362994 5

3037.ENSP00000329623->ENSP00000269305->ENSP00000263253->ENSP00000206249->ENSP00000327213->ENSP00000363812 30

3038.ENSP00000329623->ENSP00000269305->ENSP00000361021->ENSP00000341189->ENSP00000228307->ENSP00000380227->ENSP00000364094 7

3039.ENSP00000329623->ENSP00000269305->ENSP00000361021->ENSP00000341189->ENSP00000228307->ENSP00000380227->ENSP00000364094->ENSP00000282588->ENSP00000364979 19

3040.ENSP00000329623->ENSP00000269305->ENSP00000361021->ENSP00000366563 14

3041.ENSP00000329623->ENSP00000367207 2

3042.ENSP00000329623->ENSP00000269305->ENSP00000263253->ENSP00000206249->ENSP00000254227->ENSP00000342470->ENSP00000363868->ENSP00000372703 123

3043.ENSP00000329623->ENSP00000269305->ENSP00000361275->ENSP00000321656->ENSP00000256442->ENSP00000342307->ENSP00000230538->ENSP00000222399->ENSP00000374309 24

3044.ENSP00000329623->ENSP00000309103->ENSP00000270202->ENSP00000348986->ENSP00000375892 8

3045.ENSP00000329623->ENSP00000269305->ENSP00000263253->ENSP00000264657->ENSP00000376765 6

3046.ENSP00000329623->ENSP00000269305->ENSP00000278916->ENSP00000312995->ENSP00000300093->ENSP00000263753->ENSP00000418447->ENSP00000348784->ENSP00000379866 42

3047.ENSP00000329623->ENSP00000269305->ENSP00000324897->ENSP00000381648 3

3048.ENSP00000329623->ENSP00000269305->ENSP00000362649->ENSP00000384273 3

3049.ENSP00000329623->ENSP00000269305->ENSP00000361021->ENSP00000341189->ENSP00000228307->ENSP00000380227->ENSP00000364094->ENSP00000386896 8

3050.ENSP00000329623->ENSP00000269305->ENSP00000263253->ENSP00000206249->ENSP00000254227->ENSP00000342470->ENSP00000363868->ENSP00000393286 123

3051.ENSP00000329623->ENSP00000269305->ENSP00000263253->ENSP00000206249->ENSP00000254227->ENSP00000342470->ENSP00000363868->ENSP00000393870 123

3052.ENSP00000329623->ENSP00000269305->ENSP00000263253->ENSP00000262160->ENSP00000287727->ENSP00000400104 104

3053.ENSP00000329623->ENSP00000309103->ENSP00000270202->ENSP00000297494->ENSP00000341940->ENSP00000312435->ENSP00000400365 17

3054.ENSP00000329623->ENSP00000269305->ENSP00000263253->ENSP00000206249->ENSP00000254227->ENSP00000342470->ENSP00000363868->ENSP00000402590 123

3055.ENSP00000329623->ENSP00000269305->ENSP00000263253->ENSP00000325690->ENSP00000399968->ENSP00000419692 5

3056.ENSP00000330237->ENSP00000353059->ENSP00000269305->ENSP00000212015->ENSP00000264867->ENSP00000287820->ENSP00000419692->ENSP00000332296 23

3057.ENSP00000330237->ENSP00000347858->ENSP00000267169->ENSP00000227758->ENSP00000332468 4

3058.ENSP00000330237->ENSP00000347858->ENSP00000216160->ENSP00000316840->ENSP00000358622->ENSP00000339151 5

3059.ENSP00000330237->ENSP00000353059->ENSP00000269305->ENSP00000361021->ENSP00000341189 6

3060.ENSP00000330237->ENSP00000353059->ENSP00000269305->ENSP00000293195->ENSP00000342087 37

3061.ENSP00000330237->ENSP00000353059->ENSP00000345571 5

3062.ENSP00000330237->ENSP00000353059->ENSP00000269305->ENSP00000263253->ENSP00000262160->ENSP00000221930->ENSP00000346839 9

3063.ENSP00000330237->ENSP00000347858 1

3064.ENSP00000330237->ENSP00000353059->ENSP00000269305->ENSP00000361021->ENSP00000341189->ENSP00000228307->ENSP00000380227->ENSP00000364094->ENSP00000386896->ENSP00000200181->ENSP00000340937->ENSP00000348384 19

3065.ENSP00000330237->ENSP00000353059->ENSP00000269305->ENSP00000263253->ENSP00000367207->ENSP00000351490 6

3066.ENSP00000330237->ENSP00000353059->ENSP00000269305->ENSP00000417281->ENSP00000270202->ENSP00000352121 11

3067.ENSP00000330237->ENSP00000353059->ENSP00000269305->ENSP00000263253->ENSP00000206249->ENSP00000327213->ENSP00000352900 20

3068.ENSP00000330237->ENSP00000353059 1

3069.ENSP00000330237->ENSP00000353059->ENSP00000269305->ENSP00000361021->ENSP00000341189->ENSP00000228307->ENSP00000380227->ENSP00000364094->ENSP00000282588->ENSP00000364979->ENSP00000353654 22

3070.ENSP00000330237->ENSP00000353059->ENSP00000269305->ENSP00000278916->ENSP00000321656->ENSP00000256442->ENSP00000342307->ENSP00000230538->ENSP00000354360 18

3071.ENSP00000330237->ENSP00000353059->ENSP00000269305->ENSP00000267163->ENSP00000355249 5

3072.ENSP00000330237->ENSP00000347858->ENSP00000216160->ENSP00000316840->ENSP00000358997->ENSP00000263341->ENSP00000356438 8

3073.ENSP00000330237->ENSP00000347858->ENSP00000216160->ENSP00000316840->ENSP00000358622 4

3074.ENSP00000330237->ENSP00000347858->ENSP00000216160->ENSP00000316840->ENSP00000358622->ENSP00000359424 5

3075.ENSP00000330237->ENSP00000353059->ENSP00000269305->ENSP00000361021 4

3076.ENSP00000330237->ENSP00000353059->ENSP00000269305->ENSP00000278916->ENSP00000321656->ENSP00000300093->ENSP00000263753->ENSP00000418447->ENSP00000348784->ENSP00000379866->ENSP00000379823->ENSP00000331902->ENSP00000361290 47

3077.ENSP00000330237->ENSP00000347858->ENSP00000267169->ENSP00000263464->ENSP00000362994 4

3078.ENSP00000330237->ENSP00000353059->ENSP00000269305->ENSP00000263253->ENSP00000206249->ENSP00000327213->ENSP00000363812 32

3079.ENSP00000330237->ENSP00000353059->ENSP00000269305->ENSP00000361021->ENSP00000341189->ENSP00000228307->ENSP00000380227->ENSP00000364094 9

3080.ENSP00000330237->ENSP00000353059->ENSP00000269305->ENSP00000361021->ENSP00000341189->ENSP00000228307->ENSP00000380227->ENSP00000364094->ENSP00000282588->ENSP00000364979 21

3081.ENSP00000330237->ENSP00000353059->ENSP00000269305->ENSP00000361021->ENSP00000366563 16

3082.ENSP00000330237->ENSP00000353059->ENSP00000269305->ENSP00000263253->ENSP00000367207 5

3083.ENSP00000330237->ENSP00000353059->ENSP00000269305->ENSP00000263253->ENSP00000206249->ENSP00000254227->ENSP00000342470->ENSP00000363868->ENSP00000372703 125

3084.ENSP00000330237->ENSP00000353059->ENSP00000269305->ENSP00000278916->ENSP00000321656->ENSP00000256442->ENSP00000342307->ENSP00000230538->ENSP00000222399->ENSP00000374309 26

3085.ENSP00000330237->ENSP00000353059->ENSP00000269305->ENSP00000353483->ENSP00000304895->ENSP00000348986->ENSP00000375892 11

3086.ENSP00000330237->ENSP00000353059->ENSP00000269305->ENSP00000263253->ENSP00000264657->ENSP00000376765 8

3087.ENSP00000330237->ENSP00000353059->ENSP00000269305->ENSP00000278916->ENSP00000321656->ENSP00000300093->ENSP00000263753->ENSP00000418447->ENSP00000348784->ENSP00000379866 44

3088.ENSP00000330237->ENSP00000353059->ENSP00000269305->ENSP00000324897->ENSP00000381648 5

3089.ENSP00000330237->ENSP00000353059->ENSP00000269305->ENSP00000263253->ENSP00000384273 5

3090.ENSP00000330237->ENSP00000353059->ENSP00000269305->ENSP00000361021->ENSP00000341189->ENSP00000228307->ENSP00000380227->ENSP00000364094->ENSP00000386896 10

3091.ENSP00000330237->ENSP00000353059->ENSP00000269305->ENSP00000263253->ENSP00000206249->ENSP00000254227->ENSP00000342470->ENSP00000363868->ENSP00000393286 125

3092.ENSP00000330237->ENSP00000353059->ENSP00000269305->ENSP00000263253->ENSP00000206249->ENSP00000254227->ENSP00000342470->ENSP00000363868->ENSP00000393870 125

3093.ENSP00000330237->ENSP00000353059->ENSP00000269305->ENSP00000263253->ENSP00000262160->ENSP00000287727->ENSP00000400104 106

3094.ENSP00000330237->ENSP00000353059->ENSP00000269305->ENSP00000278916->ENSP00000321656->ENSP00000256442->ENSP00000342307->ENSP00000230538->ENSP00000354360->ENSP00000400365 19

3095.ENSP00000330237->ENSP00000353059->ENSP00000269305->ENSP00000263253->ENSP00000206249->ENSP00000254227->ENSP00000342470->ENSP00000363868->ENSP00000402590 125

3096.ENSP00000330237->ENSP00000353059->ENSP00000269305->ENSP00000212015->ENSP00000264867->ENSP00000287820->ENSP00000419692 7

3097.ENSP00000332296->ENSP00000419692->ENSP00000399968->ENSP00000206249->ENSP00000350941->ENSP00000275493->ENSP00000344818->ENSP00000316840->ENSP00000361359->ENSP00000332468 24

3098.ENSP00000332296->ENSP00000419692->ENSP00000399968->ENSP00000325690->ENSP00000263253->ENSP00000384273->ENSP00000339151 21

3099.ENSP00000332296->ENSP00000419692->ENSP00000399968->ENSP00000206249->ENSP00000350941->ENSP00000341189 20

3100.ENSP00000332296->ENSP00000419692->ENSP00000399968->ENSP00000325690->ENSP00000263253->ENSP00000269305->ENSP00000293195->ENSP00000342087 54

3101.ENSP00000332296->ENSP00000419692->ENSP00000399968->ENSP00000206249->ENSP00000329357->ENSP00000345571 20

3102.ENSP00000332296->ENSP00000419692->ENSP00000399968->ENSP00000206249->ENSP00000350941->ENSP00000341189->ENSP00000228307->ENSP00000380227->ENSP00000346839 23

3103.ENSP00000332296->ENSP00000419692->ENSP00000399968->ENSP00000325690->ENSP00000263253->ENSP00000269305->ENSP00000329623->ENSP00000311032->ENSP00000347858 24

3104.ENSP00000332296->ENSP00000419692->ENSP00000399968->ENSP00000206249->ENSP00000350941->ENSP00000341189->ENSP00000228307->ENSP00000380227->ENSP00000364094->ENSP00000386896->ENSP00000200181->ENSP00000340937->ENSP00000348384 33

3105.ENSP00000332296->ENSP00000419692->ENSP00000399968->ENSP00000206249->ENSP00000367207->ENSP00000351490 21

3106.ENSP00000332296->ENSP00000419692->ENSP00000399968->ENSP00000206249->ENSP00000270202->ENSP00000352121 26

3107.ENSP00000332296->ENSP00000419692->ENSP00000327213->ENSP00000352900 33

3108.ENSP00000332296->ENSP00000419692->ENSP00000399968->ENSP00000325690->ENSP00000263253->ENSP00000269305->ENSP00000353059 22

3109.ENSP00000332296->ENSP00000419692->ENSP00000399968->ENSP00000206249->ENSP00000350941->ENSP00000341189->ENSP00000228307->ENSP00000380227->ENSP00000364094->ENSP00000282588->ENSP00000364979->ENSP00000353654 36

3110.ENSP00000332296->ENSP00000419692->ENSP00000399968->ENSP00000206249->ENSP00000350283->ENSP00000372023->ENSP00000321656->ENSP00000256442->ENSP00000342307->ENSP00000230538->ENSP00000354360 34

3111.ENSP00000332296->ENSP00000419692->ENSP00000399968->ENSP00000206249->ENSP00000227507->ENSP00000267163->ENSP00000355249 22

3112.ENSP00000332296->ENSP00000419692->ENSP00000399968->ENSP00000325690->ENSP00000263253->ENSP00000264657->ENSP00000258743->ENSP00000356438 25

3113.ENSP00000332296->ENSP00000419692->ENSP00000399968->ENSP00000325690->ENSP00000263253->ENSP00000384273->ENSP00000359424->ENSP00000358622 22

3114.ENSP00000332296->ENSP00000419692->ENSP00000399968->ENSP00000325690->ENSP00000263253->ENSP00000384273->ENSP00000359424 21

3115.ENSP00000332296->ENSP00000419692->ENSP00000399968->ENSP00000325690->ENSP00000263253->ENSP00000269305->ENSP00000361021 21

3116.ENSP00000332296->ENSP00000419692->ENSP00000399968->ENSP00000206249->ENSP00000350283->ENSP00000372023->ENSP00000321656->ENSP00000300093->ENSP00000263753->ENSP00000418447->ENSP00000348784->ENSP00000379866->ENSP00000379823->ENSP00000331902->ENSP00000361290 63

3117.ENSP00000332296->ENSP00000419692->ENSP00000348827->ENSP00000320940->ENSP00000231509->ENSP00000226574->ENSP00000362994 24

3118.ENSP00000332296->ENSP00000419692->ENSP00000327213->ENSP00000363812 45

3119.ENSP00000332296->ENSP00000419692->ENSP00000399968->ENSP00000206249->ENSP00000350941->ENSP00000341189->ENSP00000228307->ENSP00000380227->ENSP00000364094 23

3120.ENSP00000332296->ENSP00000419692->ENSP00000399968->ENSP00000206249->ENSP00000350941->ENSP00000341189->ENSP00000228307->ENSP00000380227->ENSP00000364094->ENSP00000282588->ENSP00000364979 35

3121.ENSP00000332296->ENSP00000419692->ENSP00000399968->ENSP00000325690->ENSP00000263253->ENSP00000269305->ENSP00000361021->ENSP00000366563 33

3122.ENSP00000332296->ENSP00000419692->ENSP00000399968->ENSP00000206249->ENSP00000367207 20

3123.ENSP00000332296->ENSP00000419692->ENSP00000342470->ENSP00000363868->ENSP00000372703 134

3124.ENSP00000332296->ENSP00000419692->ENSP00000399968->ENSP00000206249->ENSP00000350283->ENSP00000372023->ENSP00000321656->ENSP00000256442->ENSP00000342307->ENSP00000230538->ENSP00000222399->ENSP00000374309 42

3125.ENSP00000332296->ENSP00000419692->ENSP00000399968->ENSP00000206249->ENSP00000270202->ENSP00000348986->ENSP00000375892 26

3126.ENSP00000332296->ENSP00000419692->ENSP00000399968->ENSP00000325690->ENSP00000263253->ENSP00000264657->ENSP00000376765 23
[truncated: 289,463 more chars]
